# Mixture Effects of Estrogenic Pesticides at the Human Estrogen Receptor α and β

**DOI:** 10.1371/journal.pone.0147490

**Published:** 2016-01-26

**Authors:** Bettina Seeger, Frank Klawonn, Boris Nguema Bekale, Pablo Steinberg

**Affiliations:** 1 Institute for Food Toxicology and Analytical Chemistry, University of Veterinary Medicine Hannover, Foundation, Bischofsholer Damm 15, 30173, Hannover, Germany; 2 Biostatistics Group, Helmholtz Centre for Infection Research, Inhoffenstr. 7, 38124, Braunschweig, Germany; 3 Department of Computer Science, Ostfalia University of Applied Sciences, Salzdahlumerstr. 46/48, 38302, Wolfenbüttel, Germany; University of Salerno, Faculty of Medicine and Surgery, ITALY

## Abstract

Consumers of fruits and vegetables are frequently exposed to small amounts of hormonally active pesticides, some of them sharing a common mode of action such as the activation of the human estrogen receptor α (hERα) or β (hERβ). Therefore, it is of particular importance to evaluate risks emanating from chemical mixtures, in which the individual pesticides are present at human-relevant concentrations, below their corresponding maximum residue levels. Binary and ternary iso-effective mixtures of estrogenic pesticides at effect concentrations eliciting a 1 or 10% effect in the presence or absence of 17β-estradiol were tested experimentally at the hERα in the yeast-based estrogen screen (YES) assay as well as in the human U2-OS cell-based ERα chemical-activated luciferase gene expression (ERα CALUX) assay and at the hERβ in the ERβ CALUX assay. The outcome was then compared to predictions calculated by means of concentration addition. In most cases, additive effects were observed with the tested combinations in all three test systems, an observation that supports the need to expand the risk assessment of pesticides and consider cumulative risk assessment. An additional testing of mixture effects at the hERβ showed that most test substances being active at the hERα could also elicit additive effects at the hERβ, but the hERβ was less sensitive. In conclusion, effects of the same ligands at the hERα and the hERβ could influence the estrogenic outcome under physiological conditions.

## Introduction

Many substances used as crop protection products possess hormonal activity, which may influence human health by imitating or disrupting endogenous hormones [1]. Exposure is barely avoidable considering the widespread occurrence in conventionally grown fruits, vegetables and other crops. Up to 2013 there were about 800 substances with known hormonal activity [2]; their total number still remains unknown, since many substances have not been tested for that type of activity [2]. Nowadays, high-throughput bioassays for screening purposes, which are suited to evaluate the potential of pesticides with endocrine activity, are needed. Besides the identification of effects emanating from single substances there is scientific evidence that chemical mixtures of substances sharing the same mode of action elicit predominately additive effects *in vitro* as well as *in vivo* [3–6]. Pesticide residues of substances acting in a similar way on the same cellular targets are found in/on one food sample caused by simultaneous application of various pesticides, by cross-contamination due to common storage or by application of pesticide formulations containing mixtures of pesticides sharing the same mode of action [7]. The individual residues are usually present in low concentrations, mostly below their individual maximum residue levels, but have been shown to act additively, thereby eliciting remarkable effects, even when applied in combination with the individual compounds at concentrations below their individual No Observed Adverse Effect Levels (NOAELs) [4,5,8]. A recent cumulative risk assessment approach considers evaluating pesticides in mixtures, grouped by organ-specific toxicity, in addition to evaluating individual substances [9]. The tested pesticides (pirimicarb, propamocarb, fenhexamid, fludioxonil, chlorpyrifos, fenarimol) were selected based on their occurrence as residues listed in the 2013 European Union report on pesticide residues in food [10] and their estrogenic activity known from the literature [1,11–14]. We included pesticides frequently used, like fenhexamid and fludioxonil, as well as 2,4’-DDT and 4,4’-DDT, which were banned a number of years ago and are not detected in plant-derived foodstuffs anymore [10], but are well-characterized estrogenic substances. Therefore, they were used to test whether the test systems are suited to detect compounds capable of activating the hERα and hERβ, but were not included in the mixture studies, since their occurrence in plant-derived foodstuffs, even in low concentrations, is unlikely. Unfortunately, data on human exposure to hormonally active pesticides is rarely available [15,16]. In this context, an analysis by Kortenkamp et al. [16] showed that anti-androgenic environmental contaminants are present in human serum in picomolar to nanomolar concentrations. At such concentration levels one would not expect a significant effect by individual chemicals, but mixtures of substances being present at low concentrations and sharing the same mode of action could influence the human endocrine system [4,5,8].

We investigated the effects of single pesticides (pirimicarb, propamocarb, fenhexamid, fludioxonil, chlorpyrifos, fenarimol, 2,4’-DDT and 4,4’-DDT) as well as selected binary and ternary mixtures of them at low effect concentrations in a β-galactosidase reporter gene assay, the broadly used Yeast-based Estrogen Screen (YES) assay, as well as in the human U2-OS cell-based ERα chemical-activated luciferase gene expression (ERα CALUX) assay. Full concentration-response curves were evaluated for the mathematical modeling, but the assessment of additivity was restricted to low effect concentrations (EC01 and EC10) in the range of human-relevant concentrations. Furthermore, the substances were screened in combination with a saturating concentration of 17β-estradiol (E2) to test for an E2 potentiating or an anti-estrogenic activity in the YES assay, and the anti-estrogenic substances were also tested for anti-estrogenic activity in the ERα CALUX assay.

Most studies have analyzed the mixture effects of pesticides at the hERα, while only a few reports have dealt with the effects of individual pesticides on the human estrogen receptor β (hERβ) and to our knowledge no study has investigated pesticide mixture effects at the hERβ. While the hERα frequently occurs in tissues related to reproductive activity (uterus, mammary gland), the hERβ is more widely distributed, and the ligand binding domains of the two isoforms slightly differ (59% homology) (reviewed by Gustafsson [17]), thereby indicating differing effects of the substances at the receptor isoforms. Since the hERβ is mostly regarded as a negative regulator of the hERα [18] and the risk emanating from an estrogenic substance depends on its activity at both receptor subtypes, we additionally investigated the effects of the individual test substances as well as combinations of them on the hERβ in the ERβ CALUX assay.

The well-known concept of concentration addition (CA), based on the work of Loewe and Muischnek [19], was used for the prediction of the outcome of the mixture experiments, supposing that additive effects of the pesticides occurred at the hERα and hERβ level.

The aim of this study was to evaluate the suitability of the established YES assay and the more recent ERα CALUX assay to identify estrogenic or anti-estrogenic effects at the hERα and to investigate mixture effects of estrogenic pesticides at low concentrations via CA. Additionally, the effects of the pesticides and mixtures at the hERβ, mostly acting as a counterpart of the hERα *in vivo* [18], were analyzed. The data generated for pesticide residue mixtures at the level of the hERα and hERβ support the assumption of additive effects of pesticides sharing the same mode of action, again emphasizing the importance of a cumulative risk assessment of pesticides.

## Material and Methods

### Tested chemicals

17ß-estradiol (E2; CAS# 521-18-6; ≥ 98% purity), 17α-methyltestosterone (CAS# 58-18-4; 99.5% purity), 2,4’-dichlorodiphenyltrichloroethane (2,4’-DDT CAS# 789-02-6; 99.5% purity), 4,4’- dichlorodiphenyltrichloroethane (4,4’-DDT CAS# 50-29-3; 99.8% purity), 4-hydroxytamoxifen (4-HT; CAS# 68047-06-3; ≥ 98% purity), chlorpyrifos (CAS# 2921-88-2; 99,7% purity), corticosterone (CAS# 50-22-6; ≥ 98.5% purity), fenarimol (CAS# 60168-88-9; 99.9% purity), fenhexamid (CAS# 126833-17-8; 99,7% purity), fludioxonil (CAS# 131341-86-1; 99.9% purity), ICI 182,780 (ICI; CAS# 129453-61-8; > 98% purity), pirimicarb (CAS# 23103-98-2; 98.5% purity), propamocarb (CAS# 24579-73-5; 99.3% purity), resveratrol (CAS# 501-36-0, ≥ 99% purity), and tamoxifen (CAS# 10540-29-1; ≥99% purity) were purchased from Sigma Aldrich (Schnelldorf, Germany). By dissolving the chemicals in dimethyl sulfoxide (DMSO; CAS# 67-68-5; ≥ 99.5% purity; Carl Roth, Karlsruhe, Germany), dilution series were stored at -20°C in glass vials freeze-thawed for each experiment or batched in polypropylene vials and only thawed once.

### Yeast (anti)-estrogenicity screen (YES) assay

The YES assay developed by Routledge and Sumpter [14] was used as described by Kolle et al. [20] with slight modifications: The maximal concentration of DMSO in the medium was 3% v/v and the E2 concentrations ranged from 0.01 pM to 10 nM. Each pesticide and pesticide mixture was tested in quadruplicate in at least five experiments. To asses anti-estrogenic, estrogenic or potentiating effects in combination with E2, the substances were initially tested with and without a saturating concentration of E2 (1 nM) in increasing concentrations. 4-HT at a concentration of 1 μM combined with 1 nM E2 was used as anti-estrogenic control in a complete concentration-response curve in each plate.

A cytotoxic effect was defined as a decrease in optical density of the cell suspension at 690 nm by more than 30% when compared to the solvent control [20], and such concentrations were excluded from the tests.

Substances that could not be fully solubilized in the YES assay were tested in a cell-free control plate, and the turbidity, as a benchmark for insolubility, was measured at a wavelength of 690 nm. This was necessary to distinguish between a real growth induction of the yeast cells by the tested substance and an artifact resulting from the insolubility of the compound at higher concentrations, since in both cases an increase of turbidity in the cell-containing assay would be observed. It should be taken into account that an increased turbidity could also mask cytotoxicity.

### ERα/ERβ CALUX

The ERα and ERβ CALUX assay were performed to evaluate the effect of the test substances on the hERα and hERβ as described by van der Burg et al. [21] with slight modifications. The pesticides were tested in three independent experiments, individually and as mixtures, in triplicates in 96-well plates. DMSO was used as solvent and was present in the test medium at a maximal concentration of 0.2% v/v. Solvent controls were included in each plate. Controls were selected based on the Organisation for Economic Co-operation and Development (OECD) Test Guideline 455 [22]. A full dose-response curve for E2 was included in one 96-well plate per experiment (E2 concentration range: 0.1 pM to 0.1 nM in the ERα CALUX assay, 0.3 pM to 30 nM in the ERβ CALUX assay). The same plate also contained 3 μM 17α-methyltestosterone as additional estrogenic control as well as 10 nM corticosterone as negative control. On each subsequent plate per experiment a middle concentration of E2 (3 pM in the ERα CALUX assay, 0.1 nM in the ERβ CALUX assay) and a high concentration of E2 (0.1 nM in the ERα CALUX assay, 30 nM in the ERβ CALUX assay) were tested, and the effects were compared to the respective controls on the first plate. A prescreen, in which all test substances were tested for estrogenicity between 0.01 μM and 60 μM, was performed. The three highest test substance concentrations and controls were checked for their ability to affect cell membrane integrity by quantification of the lactate dehydrogenase leakage (LDH) with the CytoTox-ONE™ Homogeneous Membrane Integrity Assay (Promega, Madison, WI, USA), which was performed as recommended by the manufacturer. Those test substance concentrations leading to a decline of the effect after a maximum was reached as well as those leading to insolubility were excluded from further experiments.

In the case of anti-estrogenicity assessment in the ERα CALUX assay, test substances that showed an anti-estrogenic activity in the YES assay, fenhexamid and fludioxonil, were tested in combination with 3 pM E2 (~ EC50). One plate per experiment included a full dose-response curve of the competitive hERα antagonist tamoxifen (3 nM to 10 μM), 1 nM 4-HT as additional anti-estrogenic control and 10 μM resveratrol as negative control, all also combined with 3 pM E2.

### Data normalization and analysis

The raw optical density values obtained at 690 nm in the YES assay were subtracted as background from the values obtained at 540 nm and thereafter the corresponding solvent control values were also subtracted. All data points were normalized to the effect of 1 nM E2 (~ EC100), and mean values were generated in the YES assay. The data from the ERα and ERβ CALUX assays were evaluated in a similar manner: Solvent controls were subtracted from the raw luminescence values and all mean values were normalized to 0.1 nM E2 (~ EC100) in the ERα CALUX assay and 30 nM (~ EC100) in the ERβ CALUX assay, with one exception: When fludioxonil and fenhexamid were tested for anti-estrogenic effects in the ERα CALUX assay, the values were normalized to 3 pM E2.

### Application of nonlinear regression in the evaluation of single substances and mixtures

Normalized concentration-response data from the YES, ERα and ERβ CALUX assays were analyzed by nonlinear regression after the x-axis with the test substance concentrations was log-transformed. The best-fit approach established by Scholze et al. [23] was used with few modifications: The regression model selection was performed using the Akaike Information Criterion with a correction for finite sample sizes, whereby logit, probit, Weibull, generalized logit I and II, as well as Aranda-Ordanz were the applicable models, while the 95% confidence intervals (CIs) were determined by application of the bootstrap method with 1,000 bootstrap simulations. All mathematical/statistical analyses were performed using R version 3.1.2 (R Foundation for Statistical Computing, Vienna, Austria).

### Mixture ratios and analysis of mixture effects by use of concentration addition (CA)

Fixed mixtures ratios were calculated to be iso-effective [24], i.e. in proportion to their individual concentration that caused a 1 or 10% effect, as calculated from the concentration-response curve of the individual compounds (effect concentrations EC01 and EC10, as listed in Table 1 for the corresponding test systems). The calculated mixture ratios are listed in the **S1 Table**. CA was applied as described by Rajapakse et al. [25]. A worked out example of the mixture ratio calculation, production of the mixtures and interpretation of the data can be found in **S1 File**.

**Table 1:** EC01 and EC10 values of the single substances in the corresponding test systems.

	YES EC01		ERα CALUX EC01		ERβ CALUX EC01		YES EC10		ERα CALUX EC10		ERβ CALUX EC10	
substance	M	[CI]	M	[CI]	M	[CI]	M	[CI]	M	[CI]	M	[CI]
propamocarb	-	-	2.09E-07	[1.66E-07-3.72E-07]	1.15E-05	[3.55E-06-2.69E-05]	-	-	7.24E-07	[6.31E-07-9.77E-07]	2.63E-05	[1.41E-05-2.95E-05]
chlorpyrifos	7.94E-08	[3.09E-08-4.57E-06]	6.17E-07	[2.14E-07-1.07E-06]	-	-	-	-	3.47E-06	[2.63E-06-4.27E-06]	-	-
fenarimol	1.17E-05	[3.89E-06-2.95E-05]	7.94E-07	[6.61E-07-1.15E-06]	3.63E-06	[1.35E-06-7.94E-06]	2.19E-05	[1.17E-05-3.72E-05]	2.82E-06	[2.63E-06-3.31E-06]	1.35E-05	[9.12E-06-2.57E-05]
fludioxonil	8.32E-07	[4.42E-08-9.22E-07]	3.63E-07	[3.16E-07-5.50E-07]	8.91E-07	[1.00E-07-1.23E-06]	2.63E-05	[6.92E-06-3.80E-05]	1.26E-06	[1.12E-06-1.48E-06]	2.09E-06	[5.89E-07-2.40E-06]
fenhexamid	2.19E-07	[1.51E-08-6.92E-07]	6.31E-07	[2.95E-07-8.71E-07]	1.17E-06	[5.89E-07-2.88E-06]	2.14E-05	[2.04E-06-2.63E-05]	2.63E-06	[1.82E-06-3.02E-06]	5.01E-06	[3.98E-06-5.62E-06]
4,4‘-DDT	3.47E-07	[1.51E-07-6.76E-07]	4.57E-07	[3.63E-07-5.13E-07]	1.02E-06	[5.50E-07-1.82E-06]	8.32E-06	[6.61E-06-1.12E-05]	1.07E-06	[9.55E-07-1.15E-06]	-	-
2,4‘-DDT	3.31E-08	[1.82E-08-6.76E-08]	4.07E-08	[2.45E-08-6.46E-08]	5.50E-08	[8.32E-09-1.86E-07]	4.68E-07	[3.55E-07-6.92E-07]	1.02E-07	[7.41E-08-1.45E-07]	6.03E-07	[4.27E-07-1.23E-06]

Comparison of the EC01 and EC10 values, the effect concentration needed to elicit a 1 or 10% effect of 1 nM E2 with the approximate 95% confidence interval [CI] of the single substances in the corresponding test systems. Pirimicarb was excluded, since it did not show an effect in any test system.

## Results

### Effect of E2 in the YES, ERα CALUX and ERβ CALUX assays

**Fig 1:**
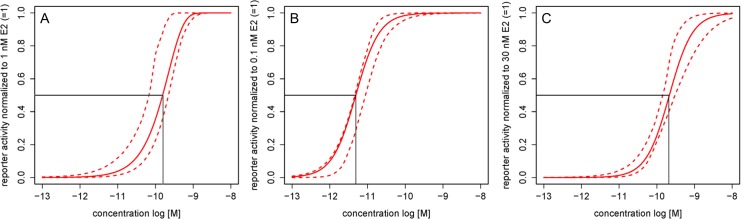
E2 in the YES, ERα CALUX and ERβ CALUX assays. Regression models with the indicated EC50 concentrations and the 95% confidence bands for E2 in the (A) YES, (B) ERα CALUX and (C) ERβ CALUX assays.

The hERα was trans-activated concentration-dependently by E2 in the YES assay (Fig 1A) and the ERα CALUX assay (Fig 1B). The maximally induced hERα activity was reached at 1 nM E2 in the YES assay and at 0.1 nM in the ERα CALUX assay, while the EC50 values were 0.16 nM and 4.9 pM, respectively. Trans-activation of the hERβ in the ERβ CALUX assay (Fig 1C) was at its maximum at 30 nM E2 with an EC50 of 0.21 nM.

### Dose-response analysis of single substances in the presence or absence of E2

**Fig 2:**
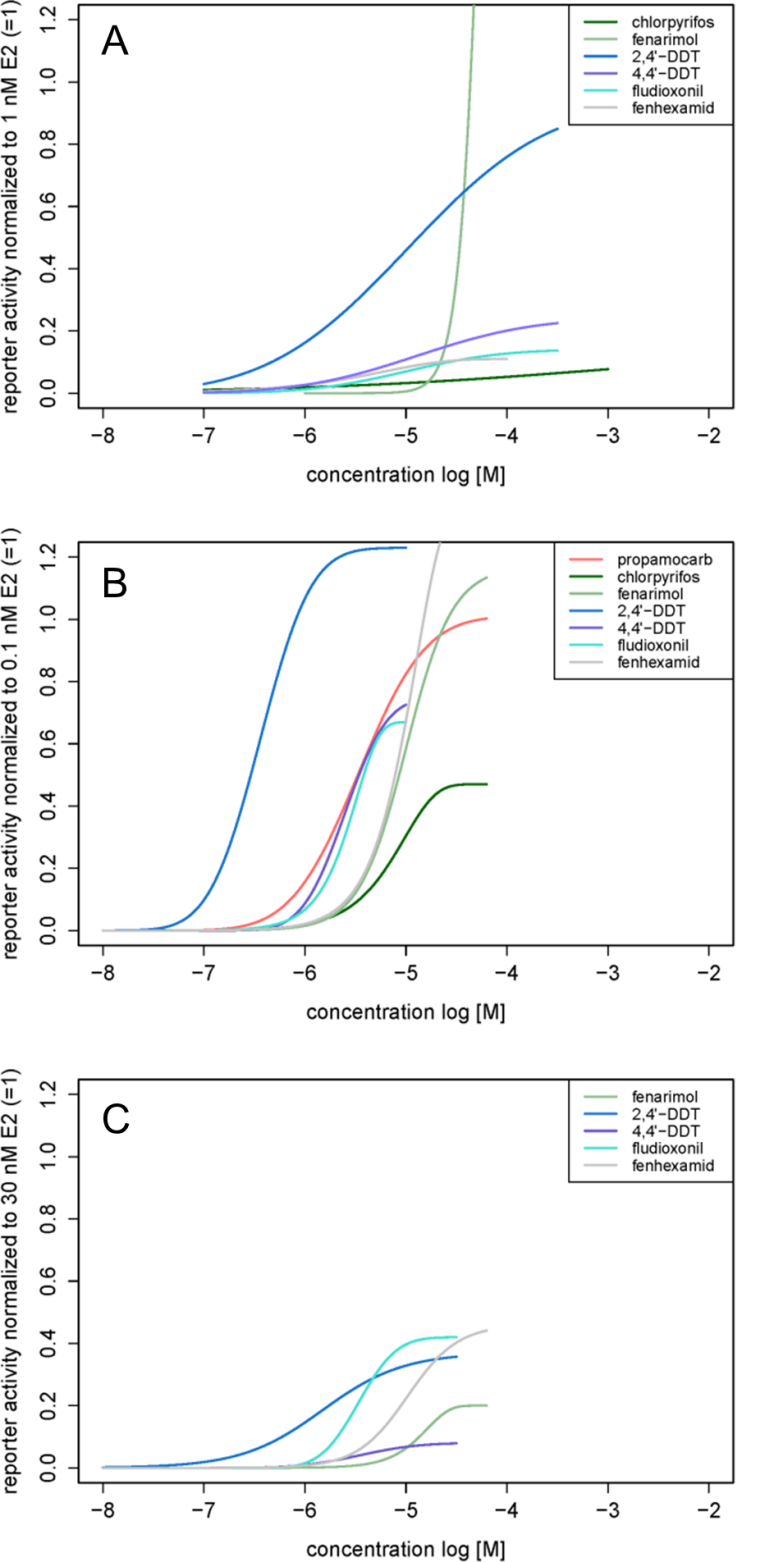
Regression models of the test substances. Regression models of the test substances in the (A) YES, (B) ERα CALUX and (C) ERβ CALUX assays. Substances not eliciting an effect are not shown.

**Fig 3:**
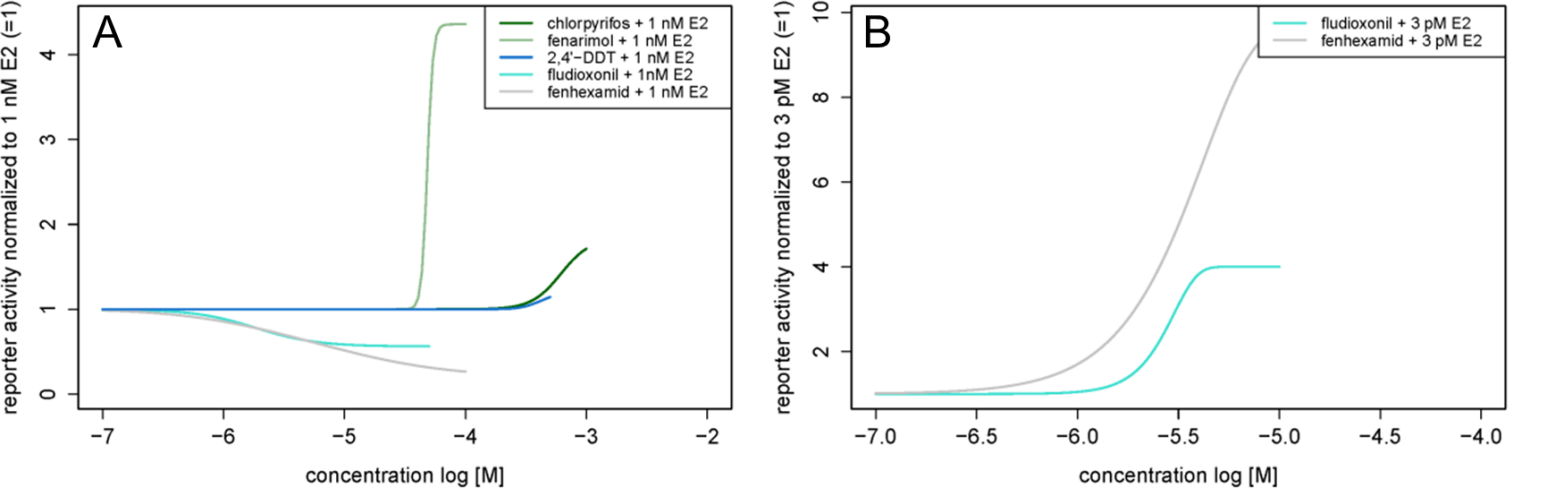
Regression models of the test substances applied in combination with E2. Regression models of the test substances in the (A) YES (with 1 nM E2) and the (B) ERα CALUX assay (with 3 pM E2). Substances not eliciting an effect are not shown.

All tested pesticides showed estrogenic activity with full concentration-response curves in the YES and ERα CALUX assays with the exception of pirimicarb, which was negative in both assays, and propamocarb, which was only active in the ERα CALUX assay (Fig 2A and 2B, Table 1, **S2 and S3 Tables**). Most substances were in the effect range of E2 in the YES assay, except for fenarimol with a maximum of 2.69 normalized to the effect of 1 nM E2 (Fig 2A and **S2 Table**). Fenarimol (maximum of 1.17) also exceeded the effect of 0.1 nM E2 in the ERα CALUX assays, as did fenhexamid (maximum of 1.37) and 2,4’-DDT (maximum of 1.23) (Fig 2B and **S3 Table**). Fenarimol, chlorpyrifos and 2,4’-DDT enhanced the effect of 1 nM E2 in the YES assay to a maximum of 4.36, 1.84 and 1.22, respectively (Fig 3A, **S5 Table**).

Fenhexamid and fludioxonil showed concentration-dependent anti-estrogenic effects when combined with 1 nM E2 in the YES assay (Fig 3A, **S5 Table**). The two above-mentioned compounds were also tested in combination with 3 pM E2 in the ERα CALUX assay, but in both cases an anti-estrogenic effect was not observed (Fig 3B, **S6 Table**).

In the ERβ CALUX propamocarb, fenarimol, fludioxonil, fenhexamid, 4,4’-DDT and 2,4’-DDT showed a dose-dependent estrogenic activity at the hERβ (Fig 2C, Table 1, **S4 Table**).

The concentrations of the test substances to be tested were limited depending on their solubility. A decreased solubility led to an increase of turbidity/optical density measured at 690 nm in the yeast-based assay or was determined by microscopic examination in the CALUX assays. Concentrations leading to insolubility were excluded in the CALUX assays, but not in the YES assay. In the CALUX assays it was possible to generate full concentration-response curves at lower concentrations. It should be pointed out that fenarimol and chlorpyrifos only showed reporter gene activity in the YES assay when applied in concentrations leading to insolubility; nevertheless, these were tested in the YES assay (100 μM fenarimol, 100–1000 μM chlorpyrifos, 100–500 μM fludioxonil, 100 μM fenhexamid, 50–500 μM 2,4’- and 4,4’-DDT). Cytotoxicity, measured by performing the LDH leakage assay, was not observed in the case of the U2-OS cells (data not shown). A decrease of turbidity in the YES assay was assumed to be equivalent to an inhibition of yeast cell growth. Fenhexamid decreased turbidity in the YES assay by more than 30% in comparison to the solvent control at concentrations ≥ 500 μM and the concentrations were therefore excluded (data not shown).

### Concentration-response analysis of mixtures

Iso-effective combinations of different estrogenic pesticides resulted in a concentration-dependent increase of reporter gene activity in the YES assay (**S1 Fig**), the ERα CALUX assay (**S2 Fig**) as well as in the ERβ CALUX assay (**S3 Fig**). In the case of the ERα CALUX assay, 95% CIs of the EC01 and EC10 were very narrow (Table 2); therefore, deviations from additivity were identified, mostly towards subadditivity (seven out of twelve predicted EC01/EC10 values were below the experimentally observed 95% CIs. Five out of ten comparisons between the experimental and the predicted data sets showed slight synergism in the YES assay, the predictions being higher than the experimentally obtained 95% CIs; the others were clearly additive (Table 3). The predicted values for the EC01 and EC10 mostly correlated with the 95% CIs of the experiments in the ERβ CALUX assay (Table 4).

**Table 2:** Regression model parameters of the mixtures in the ERα CALUX assay and comparison of the observed and predicted EC01 and EC10 values.

	Concentration-response function					EC01		EC10	
mixture	RM	${\hat{\theta }}_{1}$	${\hat{\theta }}_{2}$	${\hat{\theta }}_{\text{min}}$	${\hat{\theta }}_{\text{max}}$	predicted M	observed M [CI]	predicted M	observed M [CI]
**fludioxonil fenhexamid EC01**	Weibull	23.39	4.46	0	0.81	4.90E-07	5.89E-07 [5.01E-07-7.41E-07]	1.86E-06	2.00E-06[1.86E-06-2.19E-06]
**fludioxonil fenhexamid EC10**	Weibull	23.18	4.41	0	0.84	5.01E-07	5.50E-07 [4.90E-07-7.76E-07]	1.95E-06	1.86E-06 [1.86E-06-2.24E-06]
**chlorpyrifos fludioxonil fenhexamid EC01**	Weibull	20.96	3.99	0	0.68	5.37E-07	4.90E-07 [4.07E-07-6.31E-07]	2.29E-06	1.95E-06 [1.82E-06-2.14E-06]
**chlorpyrifos fludioxonil fenhexamid EC10**	Weibull	27.65	5.30	0	0.43	5.50E-07	1.20E-06 [8.51E-07-1.86E-06]	2.45E-06	3.39E-06 [3.09E-06-4.17E-06]
**propamocarb fludioxonil fenhexamid EC01**	Weibull	24.47	4.66	0	0.76	3.98E-07	6.76E-07 [5.25E-07-7.76E-07]	1.45E-06	2.14E-06 [1.95E-06-2.29E-06]
**propamocarb fludioxonil fenhexamid EC10**	Weibull	26.43	5.09	0	0.76	4.07E-07	9.12E-07 [7.94E-07-1.10E-06]	1.51E-06	2.63E-06 [2.51E-06-2.82E-06]

**RM**, the selected regression model; ${\hat{\theta }}_{1}$, ${\hat{\theta }}_{2}$ the estimated model parameters, ${\hat{\theta }}_{\min}$, set 0; ${\hat{\theta }}_{\max}$, the mean of the highest effect gained in the assay; **EC01/EC10** the effect concentration needed to elicit a 1 or 10% effect of 0.1 nM E2; **[CI]**, the approximate 95% confidence interval.

**Table 3:** Regression model parameters of the mixtures in the YES assay and comparison of the observed and predicted EC01/10values.

	Concentration-response function						EC01		EC10	
mixture	RM	${\hat{\theta }}_{1}$	${\hat{\theta }}_{2}$	${\hat{\theta }}_{3}$	${\hat{\theta }}_{\text{min}}$	${\hat{\theta }}_{\text{max}}$	predicted M	observed M [CI]	predicted M	observed M [CI]
**fludioxonil fenhexamid EC01**	logit	18.07	3.42	-	0	0.15	2.69E-07	8.71E-07 [1.12E-08-7.24E-06]	2.69E-05	8.32E-06 [2.63E-06-7.08E-05]
**fludioxonil fenhexamid EC10**	probit	11.05	2.14	-	0	0.15	3.89E-07	1.35E-06 [2.57E-08-5.89E-06]	3.89E-05	1.07E-05 [3.80E-06-1.10E-04]
**chlorpyrifos fludioxonil fenhexamid EC01**	logit	16.80	3.19	-	0	0.15	5.62E-07	7.94E-07 [2.63E-09-5.75E-06]	1.10E-04	8.91E-06 [3.98E-06-3.72E-05]

**RM**, the selected regression model, **glogitI**, generalized logit I; ${\hat{\theta }}_{1}$, ${\hat{\theta }}_{2}$, ${\hat{\theta }}_{3}$, the estimated model parameters, ${\hat{\theta }}_{\min}$, set 0; ${\hat{\theta }}_{\max}$, the mean of the highest effect gained in the assay; **EC01/EC10**, the effect concentration needed to elicit a 1 or 10% effect of 1 nM E2; **[CI]**, the approximate 95% confidence interval.

**Table 4:** Regression model parameters of the mixtures in the ERβ CALUX assay and comparison of the observed and predicted EC01 and EC10 values.

	Concentration-response function					EC01		EC10	
mixture	RM	${\hat{\theta }}_{1}$	${\hat{\theta }}_{2}$	${\hat{\theta }}_{\text{min}}$	${\hat{\theta }}_{\text{max}}$	Predicted M	Observed M [CI]	Predicted M	Observed M [CI]
**fludioxonil fenhexamid EC01**	logit	19.33	3.80	0	0.59	1.02E-06	6.92E-07 [5.37E-07-1.74E-06]	3.09E-06	3.09E-06 [2.75E-06-5.62E-06]
**fludioxonil fenhexamid EC10**	logit	16.88	3.36	0	0.58	1.07E-06	5.89E-07 [3.31E-07-1.62E-06]	3.55E-06	3.09E-06 [2.45E-06-5.62E-06]
**propamocarb fludioxonil fenhexamid EC01**	Weibull	26.92	5.72	0	0.19	4.47E-06	6.03E-06 [3.80E-06-8.13E-06]	1.23E-05	1.74E-05 [1.17E-05-2.29E-05]
**propamocarb fludioxonil fenhexamid EC10**	Weibull	79.24	16.21	0	0.22	3.72E-06	8.32E-06 [4.37E-06-9.12E-06]	1.12E-05	1.20E-05 [1.15E-05-1.91E-05]

**RM**, the selected regression model; ${\hat{\theta }}_{1}$, ${\hat{\theta }}_{2}$ the estimated model parameters, ${\hat{\theta }}_{\min}$, set 0; ${\hat{\theta }}_{\max}$, the mean of the highest effect gained in the assay; **EC01/EC10** the effect concentration needed to elicit a 1 or 10% effect of 30 nM E2; **[CI]**, the approximate 95% confidence interval.

## Discussion

In the present study mostly additive effects of pesticide mixtures at low effect concentrations in the YES, the ERα CALUX as well as the ERβ CALUX assays were observed. The predicted EC01/10 values not lying in the range of the 95% CIs of the experiments in the ERα CALUX (Table 2) can be explained by the fact that the 95% CIs were very narrow. The concentrations of the prediction are maximally 1.32-fold higher or 0.45-fold lower than the experimentally obtained data (Table 2), which does not represent a biologically relevant deviation from additivity. Those findings underpin the applicability of CA to predict mixture effects of pesticides sharing a common mode of action in the human cell-based ERα CALUX and ERβ CALUX assays as well as in the yeast-based YES assay. This additive behavior of estrogenic substances was previously shown in studies with hERα reporter gene bioassays [4] as well as in a MCF-7 proliferation assay [13] and *in vivo* in the immature mouse uterotrophic assay [26]. To our knowledge, this is the first study that has evaluated mixture effects of pesticides at the hERα and hERβ. Since the hERβ may acts as counterpart of the hERα, it could be of great relevance for the risk assessment process not to limit the analysis to the mixture effects of low concentrations of pesticides to the hERα level, since most hERα ligands also bind to the hERβ, which is also valid for the test substances in this study. The extent of the estrogenic effect in the different tissues largely depends on their hERα:hERβ ratio (reviewed by Böttner et al. [18]). In the present study, it has been shown that the pesticides did not elicit the same maximal response at the hERβ as compared to the effects at the hERα (e.g. Fig 2), but the potencies of the substances to elicit a 1 or 10% effect (Table 1, EC01 and EC10 in comparison in the corresponding test systems) are in the same order of magnitude when compared to the ECs in the ERα CALUX. These results, in turn, lead to the conclusion that most pesticides tested at low concentrations as well as their mixtures induce stronger effects at the hERα when directly compared to those at the hERβ, but the same substances elicit estrogenic effects in an additive manner at the hERβ. Additive effects of pesticides at the hERα activating the corresponding receptors should be considered in a cumulative risk assessment, and it is an important finding that the substances eliciting additive effects at the hERα in most cases also influence the hERβ additively, albeit with a lower efficacy.

There was a high level of concordance between the ERα CALUX and the YES assay regarding the identification of estrogenic effects of single substances. With the exception of pirimicarb and propamocarb, all tested pesticides trans-activated the hERα in both test systems. Pirimicarb was negative in both bioassays. There were certain differences in the identification of estrogenic and anti-estrogenic activity in the selected reporter gene assays: Propamocarb was identified as an estrogenic compound with an effect at the hERα in the ERα CALUX assay but not in the YES assay. Both substances were applied alone in the YES and in the ERα CALUX assay and in combination with a saturating concentration of 1 nM E2 in the YES assay. As known from the scientific literature, pirimicarb [11,27] and propamocarb [11] did not stimulate the proliferation of MCF-7 cells in the E-Screen with or without E2, but they induced a stronger effect in a β-galactosidase and luciferase-based transactivation assay performed with MCF-7 BOS cells, when added in combination with a saturating concentration of E2 [11]. In this case, it was suggested that the substances could influence the interaction of E2 or cofactors with the estrogen receptor or the binding of the ligand-receptor-complex to its response element [11]. Bonefeld-Jorgensen et al. [28] also showed that propamocarb did not elicit an effect alone and in combination with an E2 concentration at the EC50 level in a stably transfected reporter gene assay based on MVLN cells and speculated that propamocarb might interact with the reporter vector and/or internal standard vector in the MCF-7 BOS cell-based assay used in the study of Andersen et al. [11], therefore leading to an artifact. Pirimicarb was negative in both reporter gene assays used in this study, even when combined with 1 nM E2 (saturating effect) in the YES assay. Interestingly, propamocarb, which did not induce any effects in the YES assay, was able to elicit concentration-dependent effects in the ERα and ERβ CALUX assays when applied alone. As far as we know, there is no *in vivo* data concerning the estrogenic activity of these two substances, and it is not possible to assess their estrogenicity based on the outcome of the above-mentioned studies, but it is most likely that the described potentiation the E2 effect in the transiently transfected MCF-7 BOS cell assay used in the study of Andersen et al. [11] depends on a particular interaction in this assay. An alternative explanation for the positive outcome in the CALUX assays could be that propamocarb is unable to cross the yeast cell wall or that it could be metabolized to compounds with a certain estrogenicity in the U2-OS cells used in the CALUX assays. Although U2-OS cells do not express any cytochrome P450 enzymes, they possess a certain enzymatic capacity, as shown in a proteomic analysis by Niforou et al. [29], which could impact the estrogenicity of propamocarb in the ERα CALUX assay, but not in the YES assay.

We detected an anti-estrogenic activity of fenhexamid and fludioxonil when applied in combination with E2 in the YES assay (Fig 3A), which could not be confirmed in the ERα CALUX assay (Fig 3B). Fenhexamid and fludioxonil led to an inhibition of the E2 effect in the YES assay by 79 and 44%, which is in line with a study by Mertl et al. [30] on food contact materials: Some of these substances showed an anti-estrogenic effect in the YES assay, but not in the ERα CALUX assay. Teng et al. [31] reported an increase of miR-21 expression in breast cancer cells and an inhibition of E2-induced cell proliferation in MCF-7 cells when applying fenhexamid and fludioxonil, which are associated with downstream anti-estrogenic effects.

Supramaximal estrogenic effects of substances eliciting higher putative effects than E2 itself were observed (Fig 2A and 2B, **S2 and S3 Tables**) and were also shown to act additively as a mixture in the YES assay (**S9 Table**, **Figure C and D in S1 Fig**). As shown in the Supplemental Material section, those supramaximal effects are receptor-dependent, mostly occur at high concentrations not relevant for the risk assessment of pesticide residues at low concentrations and are most likely to be a post-transcriptional artifact in hERα reporter gene bioassays [32–34] and do not deviate from the assumption of additivity, consequently not influencing the results of the present mixture study. Furthermore, fenarimol and chlorpyrifos were tested in a MCF-7 proliferation assay by Vinggaard et al. [1] without exceeding the effect of E2, thereby underlining that the biological relevance of the supramaximal effects in the YES assay is questionable.

## Conclusions

To our knowledge, this is the first study to test mixtures of pesticides for additive effects not only at the hERα but also at the hERβ level. Most pesticides being active at the hERα were also active at the hERβ, and additive effects were observed when applied as mixtures. Although the pesticide mixtures were less efficient at the hERβ, one should analyze their effects at both receptor subtypes, since after an exposure one would expect that the compounds interact with both receptors in humans, the receptor subtype ratio influencing the estrogenic outcome. Additive effects of the tested pesticides at the hERα were mostly observed in the YES assay as well as in the ERα CALUX assay, an observation that supports the assumption of additivity of pesticides sharing a mode of action. The used *in vitro* assays are suitable for the identification of estrogenic effects at the hERα and hERβ level and, moreover, for the hazard identification step in the risk assessment process. The shown additive effects implicate that pesticide mixtures sharing a mode of action should be taken into account, besides a single substance assessment, in a cumulative risk assessment approach.

## Supporting Information

S1 Fig Regression models for mixtures in the YES assay. **Red line**, regression models of the mixture experiments with 95% confidence belts; **blue line,** CA model prediction; (A) EC01 and (B) EC10 mixture of fludioxonil and fenhexamid; (C) EC101 and (D) EC110 mixture of chlorpyrifos, fenarimol and 1 nM E2; (E) EC01 and (F) EC10 mixture of chlorpyrifos, fludioxonil and fenhexamid.
(TIFF)

S2 Fig Regression models for mixtures in the ERα CALUX assay. **Red line**, regression models of the mixture experiments with 95% confidence belts; **blue line,** CA model prediction; (A) EC01 and (B) EC10 mixture of fludioxonil and fenhexamid; (C) EC101 and (D) EC110 mixture of propamocarb, fludioxonil and fenhexamid; (E) EC01 and (F) EC10 mixture of chlorpyrifos, fludioxonil and fenhexamid.
(TIFF)

S3 Fig Regression models for mixtures in the ERβ CALUX assay. **Red line**, regression models of the mixture experiments with 95% confidence belts; **blue line,** CA model prediction; (A) EC01 and (B) EC10 mixture of fludioxonil and fenhexamid; (C) EC101 and (D) EC110 mixture of propamocarb, fludioxonil and fenhexamid.
(TIFF)

S4 Fig Regression models of pesticides applied together with competitive inhibitors of the hERα. Regression models with 95% confidence bands; dashed end of the regression model line stands for concentrations at which the turbidity of the yeast suspension was reduced; S4A–S4F Fig show experiments in the YES assay with (A) 1 mM chlorpyrifos applied together with 1 nM E2 and increasing concentrations of 4-hydroxytamoxifen; (B) 1 mM chlorpyrifos applied together with 1 nM E2 and increasing concentrations of ICI 184,780; (C) 100 μM fenarimol applied together with 1 nM E2 and increasing concentrations of 4-hydroxytamoxifen; (D) 100 μM fenarimol applied together with 1 nM E2 and increasing concentrations of ICI 184,780; (E) 100 μM fenarimol applied together with increasing concentrations of 4-hydroxytamoxifen; (F) 100 μM fenarimol applied together with increasing concentrations of ICI 184,780; (G) 60 μM fenhexamid applied together with increasing concentrations of tamoxifen were tested in the ERα CALUX assay.
(TIFF)

S1 File Calculation scenario. for an iso-effective binary mixture of fludioxonil and fenhexamid in the ERα CALUX assay, based on their individual EC10 values.
(PDF)

S2 File Raw Data. (PDF)

S1 Table Mixture components and ratios. Iso-effective mixtures based on EC01/EC10 or EC101/EC110 values of the single compounds.
(PDF)

S2 TableRegression models of single substances in the YES assay.**RM**, the selected regression model; ${\hat{\theta }}_{1}$, ${\hat{\theta }}_{2}$ the estimated model parameters; ${\hat{\theta }}_{\min}$, set 0; ${\hat{\theta }}_{\max}$, the mean of the highest effect observed in the assay, corresponding to the effect induced by 1 nM E2.(PDF)

S3 TableRegression models of single substances in the ERα CALUX assay.**RM**, the selected regression model; ${\hat{\theta }}_{1}$, ${\hat{\theta }}_{2}$ the estimated model parameters; ${\hat{\theta }}_{\min}$, set 0; ${\hat{\theta }}_{\max}$, the mean of the highest effect observed in the assay, corresponding to the effect induced by 0.1 nM E2.(PDF)

S4 TableRegression models of single substances in the ERβ CALUX assay.**RM**, the selected regression model; ${\hat{\theta }}_{1}$, ${\hat{\theta }}_{2}$ the estimated model parameters; ${\hat{\theta }}_{\min}$, set 0; ${\hat{\theta }}_{\max}$, the mean of the highest effect observed in the assay, corresponding to the effect induced by 30 nM E2.(PDF)

S5 TableEstrogenic effects of single substances in the YES assay when combined with 1 nM E2.**RM**, the selected regression model; glogitII, generalized logit II; ${\hat{\theta }}_{1}$, ${\hat{\theta }}_{2}$, ${\hat{\theta }}_{3}$ the estimated model parameters; ${\hat{\theta }}_{\min}$, set 1 if the effect is enhanced by the test compound in comparison to 1 nM E2 or the smallest mean value if the substance inhibits the effect of E2; ${\hat{\theta }}_{\max}$, the mean of the highest effect observed in the assay, corresponding to the effect induced by 1 nM E2 or set to 1 if the substance inhibits the effect of E2; EC101/EC110, the effect concentration needed to elicit a 101% or 110% effect of 1 nM E2; [CI], the approximate 95% confidence interval.(PDF)

S6 TableRegression models of single substances in the ERα CALUX assay when combined with 3 pM E2.**RM**, the selected regression model; ${\hat{\theta }}_{1}$, ${\hat{\theta }}_{2}$, the estimated model parameters; ${\hat{\theta }}_{\min}$, set 1; ${\hat{\theta }}_{\max}$, the mean of the highest effect observed in the assay, corresponding to the effect induced by 3 pM E2.(PDF)

S7 TableCombination of pesticides with competitive hERα inhibitors in the YES assayRM, the selected regression model; glogitI, generalized logit I; ${\hat{\theta }}_{1}$, ${\hat{\theta }}_{2}$, ${\hat{\theta }}_{3}$ the estimated model parameters; ${\hat{\theta }}_{\min}$, the mean of the highest anti-estrogenic effect observed in the assay, corresponding to the effect induced by 1 nM E2; ${\hat{\theta }}_{\max}$, the mean of the highest effect observed in the assay, corresponding to the effect induced by 1 nM E2.(PDF)

S8 TableCombination of pesticides with competitive hERα inhibitors in the ERα CALUX assayRM, the selected regression model; ${\hat{\theta }}_{1}$, ${\hat{\theta }}_{2}$, the estimated model parameters; ${\hat{\theta }}_{\min}$, the mean of the highest anti-estrogenic effect observed in the assay, corresponding to the effect induced by 0.1 nM E2; ${\hat{\theta }}_{\max}$, the mean of the highest effect observed in the assay, corresponding to the effect induced by 0.1 nM E2.(PDF)

S9 TableMixtures of pesticides with supramaximal effects in the YES assayRM, the selected regression model, glogitI, generalized logit I; ${\hat{\theta }}_{1}$, ${\hat{\theta }}_{2}$, ${\hat{\theta }}_{3}$, the estimated model parameters, ${\hat{\theta }}_{\min}$, set 1; ${\hat{\theta }}_{\max}$, the mean of the highest effect gained in the assay; EC101/EC110, the effect concentration needed to elicit a 101or 110% effect of 1 nM E2; [CI], the approximate 95% confidence interval.(PDF)
