# Supplementary figures and images for: Mixture Effects of Estrogenic Pesticides at the Human Estrogen Receptor α and β
Source: PLoS One. 2016 Jan 26;11(1):e0147490. doi: 10.1371/journal.pone.0147490 (PMC4728068; doi:10.1371/journal.pone.0147490)

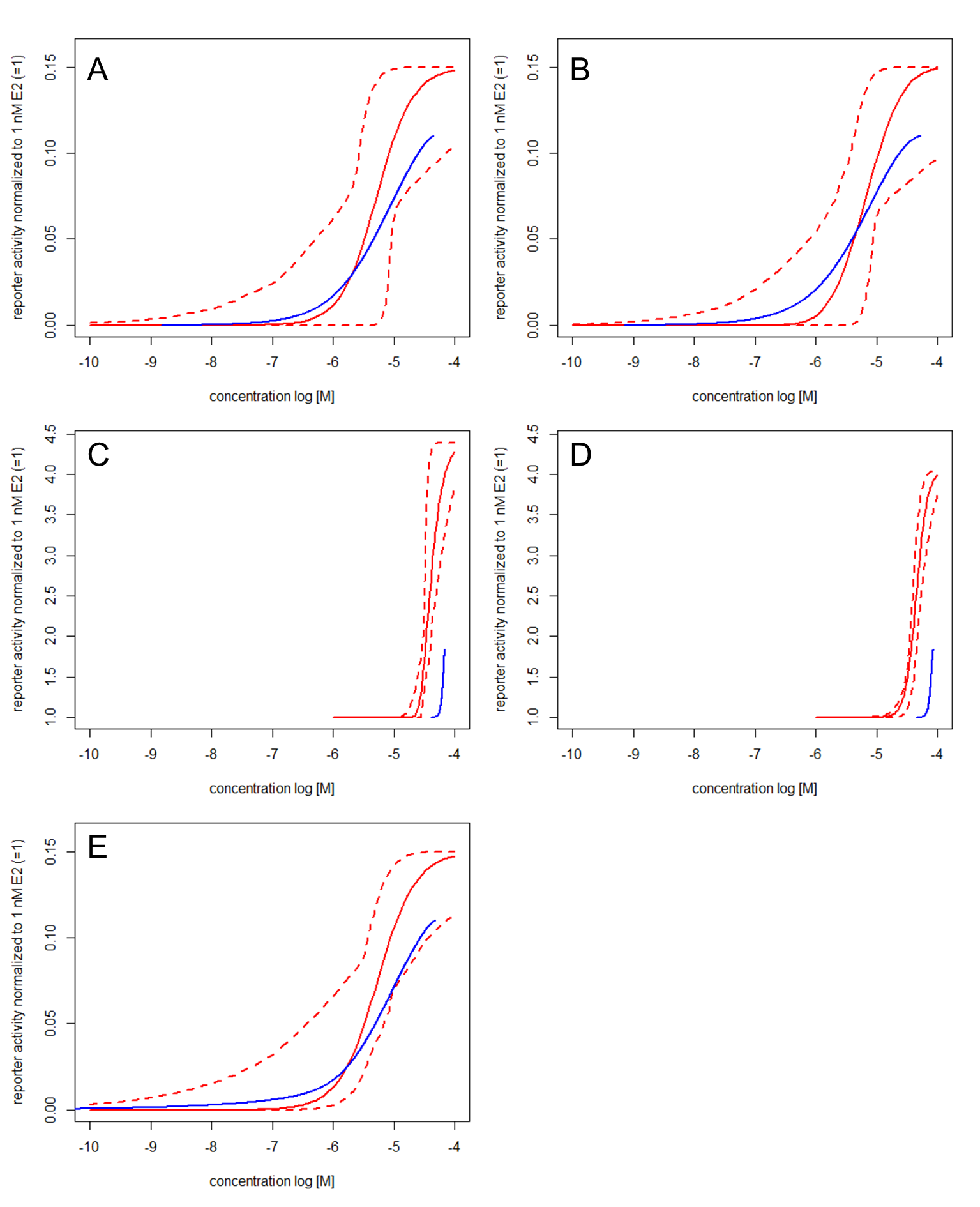

Supplement: S1 Fig — Red line, regression models of the mixture experiments with 95% confidence belts; blue line, CA model prediction; (A) EC01 and (B) EC10 mixture of fludioxonil and fenhexamid; (C) EC101 and (D) EC110 mixture of chlorpyrifos, fenarimol and 1 nM E2; (E) EC01 and (F) EC10 mixture of chlorpyrifos, fludioxonil and fenhexamid. (TIFF) [file pone.0147490.s001.tiff]

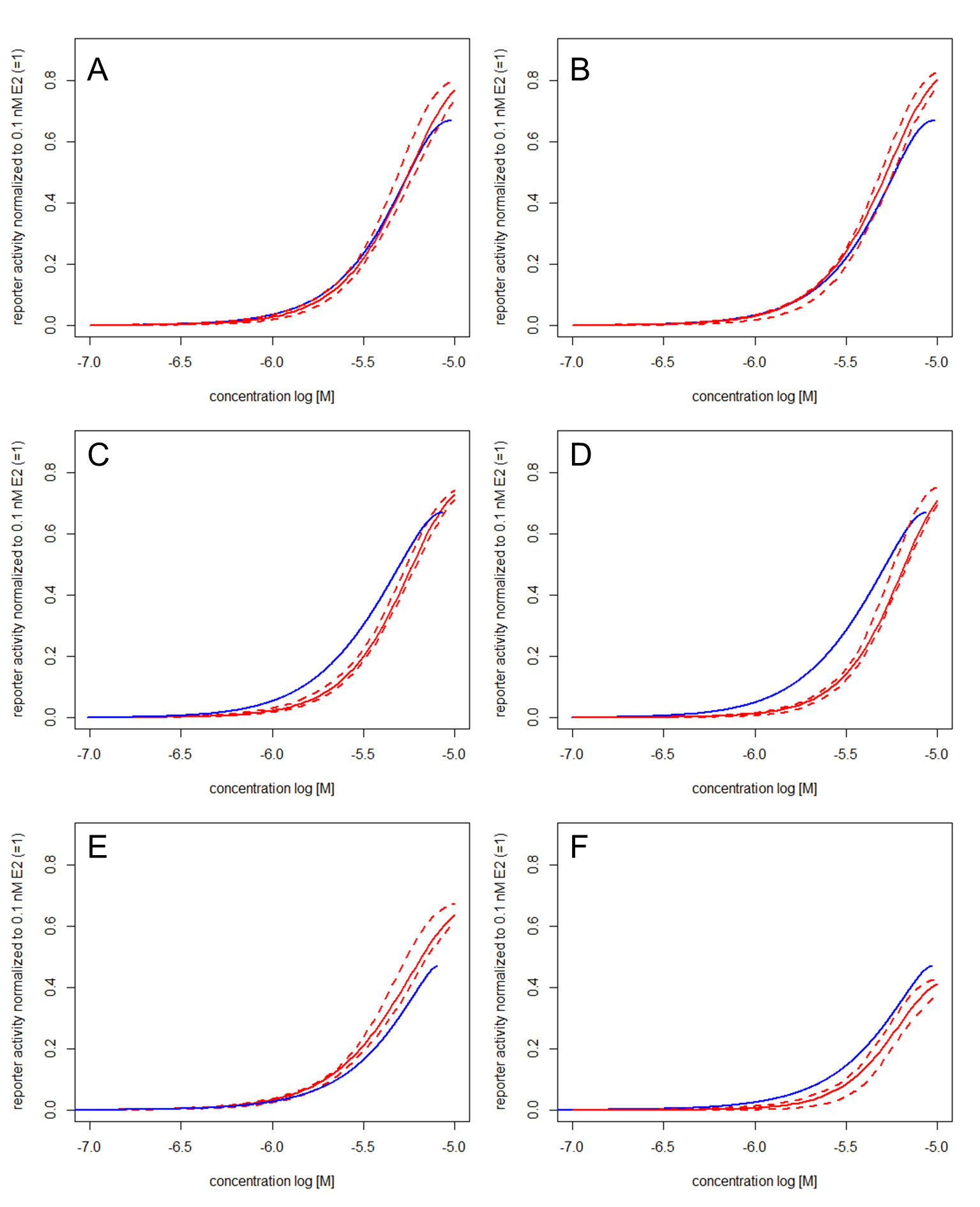

Supplement: S2 Fig — Red line, regression models of the mixture experiments with 95% confidence belts; blue line, CA model prediction; (A) EC01 and (B) EC10 mixture of fludioxonil and fenhexamid; (C) EC101 and (D) EC110 mixture of propamocarb, fludioxonil and fenhexamid; (E) EC01 and (F) EC10 mixture of chlorpyrifos, fludioxonil and fenhexamid. (TIFF) [file pone.0147490.s002.tiff]

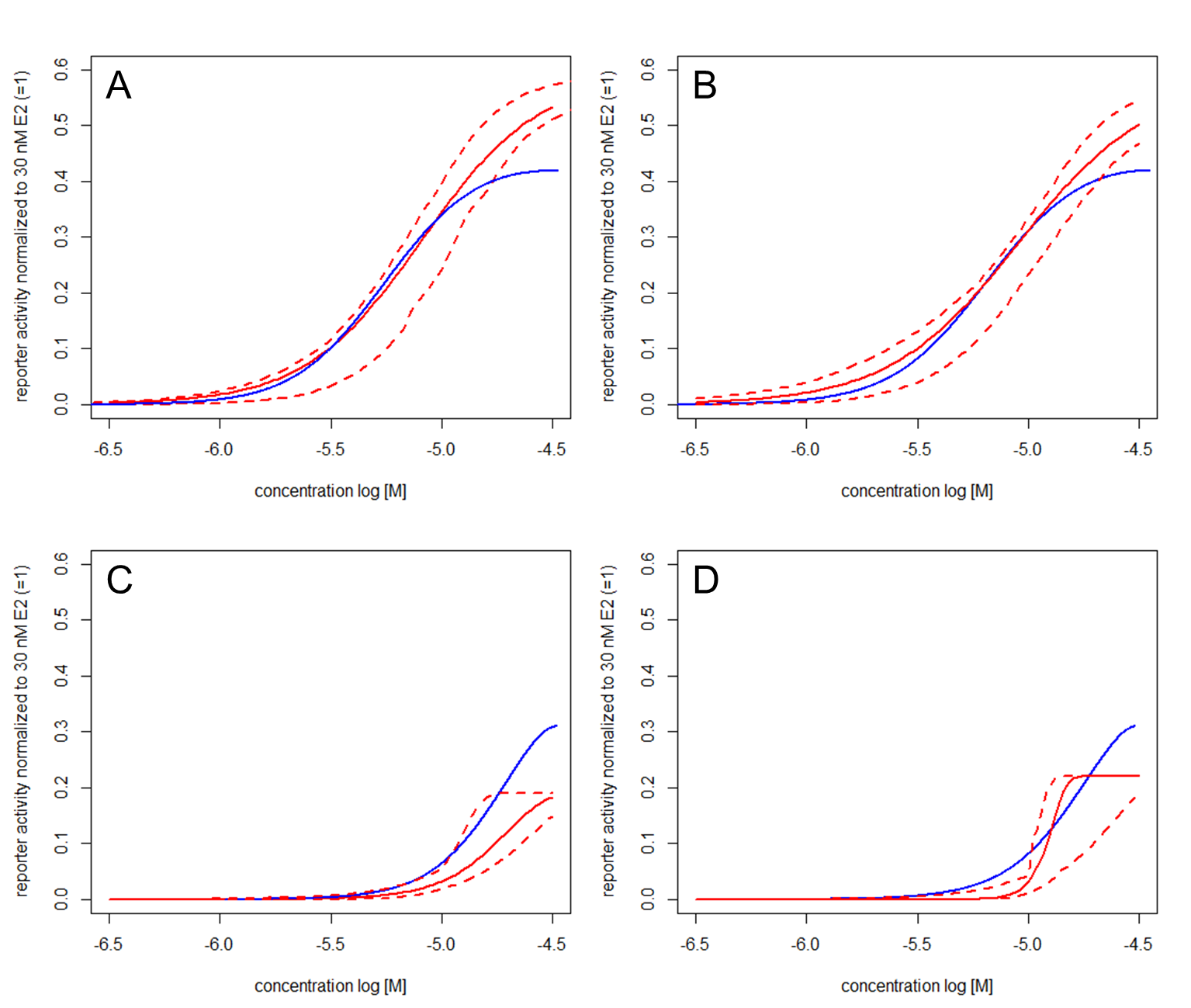

Supplement: S3 Fig — Red line, regression models of the mixture experiments with 95% confidence belts; blue line, CA model prediction; (A) EC01 and (B) EC10 mixture of fludioxonil and fenhexamid; (C) EC101 and (D) EC110 mixture of propamocarb, fludioxonil and fenhexamid. (TIFF) [file pone.0147490.s003.tiff]

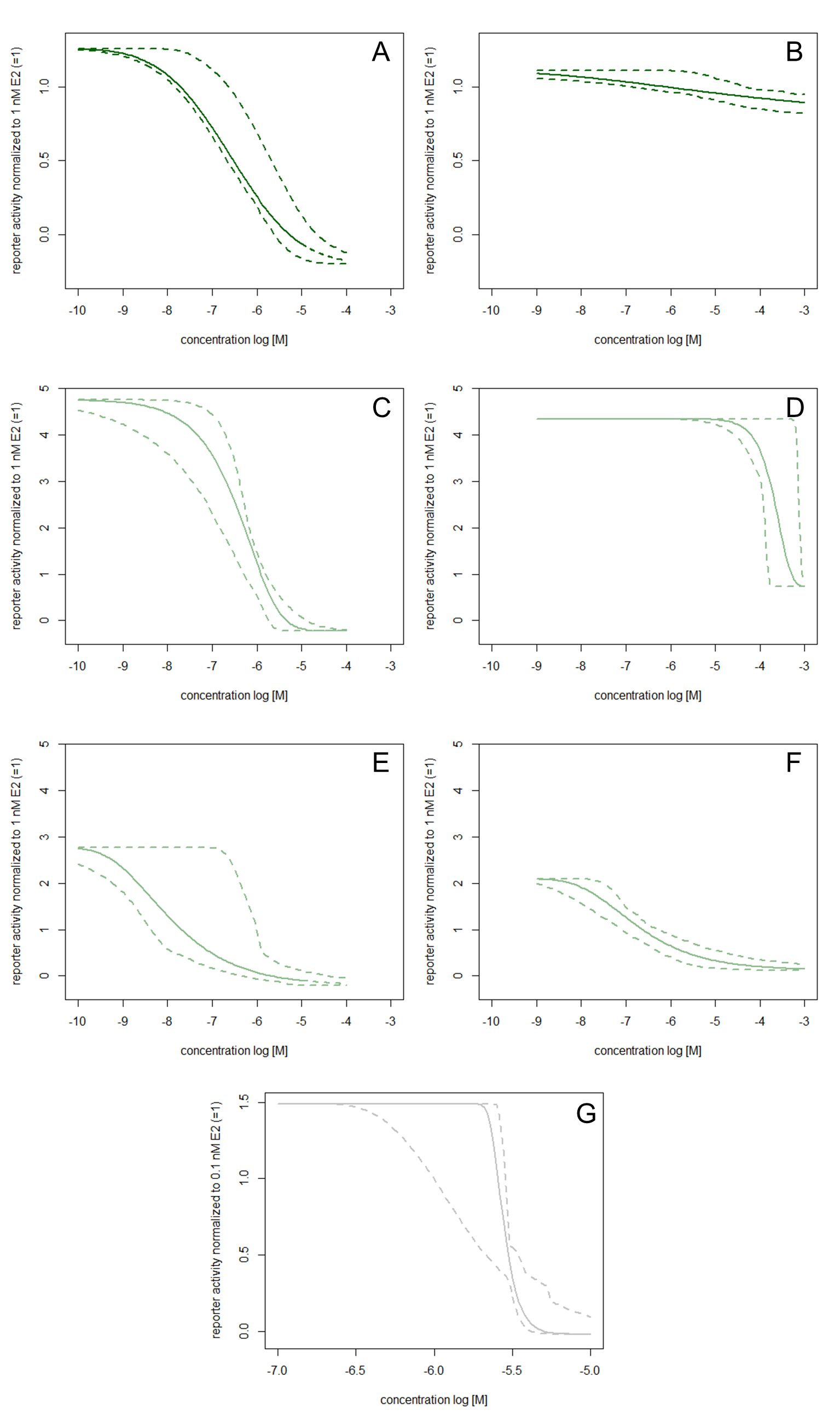

Supplement: S4 Fig — Regression models with 95% confidence bands; dashed end of the regression model line stands for concentrations at which the turbidity of the yeast suspension was reduced; S4A–S4F Fig show experiments in the YES assay with (A) 1 mM chlorpyrifos applied together with 1 nM E2 and increasing concentrations of 4-hydroxytamoxifen; (B) 1 mM chlorpyrifos applied together with 1 nM E2 and increasing concentrations of ICI 184,780; (C) 100 μM fenarimol applied together with 1 nM E2 and increasing concentrations of 4-hydroxytamoxifen; (D) 100 μM fenarimol applied together with 1 nM E2 and increasing concentrations of ICI 184,780; (E) 100 μM fenarimol applied together with increasing concentrations of 4-hydroxytamoxifen; (F) 100 μM fenarimol applied together with increasing concentrations of ICI 184,780; (G) 60 μM fenhexamid applied together with increasing concentrations of tamoxifen were tested in the ERα CALUX assay. (TIFF) [file pone.0147490.s004.tiff]
