# Supplementary material for: Mixture Effects of Estrogenic Pesticides at the Human Estrogen Receptor α and β
Source: PLoS One. 2016 Jan 26;11(1):e0147490. doi: 10.1371/journal.pone.0147490 (PMC4728068; doi:10.1371/journal.pone.0147490)
Supplement: S1 File — for an iso-effective binary mixture of fludioxonil and fenhexamid in the ERα CALUX assay, based on their individual EC10 values. (PDF) [file pone.0147490.s005.pdf]

substance	**EC10 for the individual substance (Table 1)**	**mixture ratio (S1 Table)**
fludioxonil	1.26E-06	32.39%
fenhexamid	2.63E-06	67.61%
	**3.89E-06**	**100%**

1. EC10 is calculated for each individual substance.
2. The concentrations are summed up and set at 100%.
3. The ratio in the mixture is calculated for each substance as an iso-effective mixture.
4. The stock solution is prepared and dilution series are made: In a binary mixture, in our example a 10 mM stock consisting of 32.39% fludioxonil and 67.61% fenhexamid (i.e. 3.24 mM fludioxonil and 6.76 mM fenhexamid), a double concentrated stock of the individual substances, dissolved in DMSO, is prepared, and the two single substance stocks are mixed at a ratio of 1:1. The same scheme is applicable for ternary mixtures, but in this case the three times concentrated stocks for the individual substances are mixed at a ratio of 1:1:1.
5. The experiments are performed, and regression models are calculated. Then, the EC01 and EC10 values with the 95% CI are calculated.
6. Mixtures are calculated by concentration addition (CA) from the experiments with the individual substances. Thereafter, the EC01 and EC10 values of the predicted mixture are calculated.
7. The outcome of the prediction (CA) is compared to the experimentally gained data. If the predicted mean (1.95 µM) is within the 95% CI [1.86-2.24] of the experimental data, the effect is statistically classified to be additive (see below).

Mixture potency (Table 2)

Predicted EC10: 1.95 µM

Observed EC10: 1.86 µM [1.86-2.24]
