# Supplementary material for: Mixture Effects of Estrogenic Pesticides at the Human Estrogen Receptor α and β
Source: PLoS One. 2016 Jan 26;11(1):e0147490. doi: 10.1371/journal.pone.0147490 (PMC4728068; doi:10.1371/journal.pone.0147490)
Supplement: S2 File — (PDF) [file pone.0147490.s006.pdf]

YES pirimicarb + 1nM E2

1st experient

540nm

cocentration [M]	Replicate 1	Replicate 2	Replicate 3	Replicate 4
1,00E-10	1,014899969	0,987600029	1,023800015	1,042999983
1,00E-09	1,038400054	1,01820004	1,029099941	1,050899982
1,00E-08	1,044499993	1,064599991	1,032600045	1,01730001
1,00E-07	0,990700006	1,029399991	1,039399981	1,01030004
1,00E-06	1,006700039	1,010599971	1,048599958	1,042400002
1,00E-05	1,033699989	1,040699959	1,0273	1,029000044
1,00E-04	0,999499977	1,06219995	1,064100027	1,089300036

690nm

cocentration [M]	Replicate 1	Replicate 2	Replicate 3	Replicate 4
1,00E-10	0,609899998	0,585799992	0,612999976	0,623600006
1,00E-09	0,628199995	0,609700024	0,631200016	0,643000007
1,00E-08	0,636799991	0,657999992	0,625400007	0,615100026
1,00E-07	0,595300019	0,622699976	0,626699984	0,607200027
1,00E-06	0,600000024	0,601300001	0,63319999	0,629599988
1,00E-05	0,634599984	0,635999978	0,626999974	0,621999979
1,00E-04	0,57160002	0,620500028	0,626800001	0,645600021

solvent control 690nm

	Replicate 1	Replicate 2	Replicate 3	Replicate 4
	0,700800002	0,688000023	0,677600026	0,650300026

540-690nm

cocentration [M]	Replicate 1	Replicate 2	Replicate 3	Replicate 4
1,00E-10	0,405000001	0,401800007	0,41080001	0,419400007
1,00E-09	0,410299987	0,408499986	0,397799999	0,407900006
1,00E-08	0,407700002	0,406699985	0,407200009	0,402200013
1,00E-07	0,395300001	0,406699985	0,412699997	0,403100014
1,00E-06	0,406599998	0,409399986	0,415399998	0,412800014
1,00E-05	0,399100006	0,404700011	0,400299996	0,407099992
1,00E-04	0,4278	0,441799998	0,437299997	0,443699986

solvent control 540nm-690nm

	Replicate 1	Replicate 2	Replicate 3	Replicate 4
	0,193800002	0,144600004	0,144199997	0,144199997

(540nm-690nm)-solvent control (540nm-690nm)

cocentration [M]	Replicate 1	Replicate 2	Replicate 3	Replicate 4
1,00E-10	0,211199999	0,257200003	0,266600013	0,275200009
1,00E-09	0,216499984	0,263899982	0,253600001	0,263700008
1,00E-08	0,2139	0,262099981	0,263000011	0,258000016
1,00E-07	0,201499999	0,262099981	0,2685	0,258900017
1,00E-06	0,212799996	0,264799982	0,271200001	0,268600017
1,00E-05	0,205300003	0,260100007	0,256099999	0,262899995
1,00E-04	0,233999997	0,297199994	0,293099999	0,299499989

(540nm-690nm)-solvent control E2 reference

cocentration [M]	Replicate 1	Replicate 2	Replicate 3	Replicate 4
1,00E-09	0,288800001	0,275399998	0,277500004	0,264999986

Normalization (substance/1 nM E2)

cocentration [M]	Replicate 1	Replicate 2	Replicate 3	Replicate 4	Mean
1,00E-10	73,13%	93,39%	96,07%	103,85%	**91,61%**
1,00E-09	74,97%	95,82%	91,39%	99,51%	**90,42%**
1,00E-08	74,07%	95,17%	94,77%	97,36%	**90,34%**
1,00E-07	69,77%	95,17%	96,76%	97,70%	**89,85%**
1,00E-06	73,68%	96,15%	97,73%	101,36%	**92,23%**
1,00E-05	71,09%	94,44%	92,29%	99,21%	**89,26%**
1,00E-04	81,02%	107,92%	105,62%	113,02%	**101,90%**

yeast-growth/solubility 690nm/solvent control 690nm

cocentration [M]	Replicate 1	Replicate 2	Replicate 3	Replicate 4	Mean
1,00E-10	87,03%	85,15%	90,47%	95,89%	**89,63%**
1,00E-09	89,64%	88,62%	93,15%	98,88%	**92,57%**
1,00E-08	90,87%	95,64%	92,30%	94,59%	**93,35%**
1,00E-07	84,95%	90,51%	92,49%	93,37%	**90,33%**
1,00E-06	90,55%	92,44%	92,53%	95,65%	**92,79%**
1,00E-05	85,62%	87,40%	93,45%	96,82%	**90,82%**
1,00E-04	81,56%	90,19%	92,50%	99,28%	**90,88%**

4-HT (540nm-690nm) anti-estrogenic control

cocentration [M]	Replicate 1	Replicate 2	Replicate 3	Replicate 4
1,00E-06	0,014200002	0,063599989	0,059699997	0,063900009

4-HT (540nm-690nm)/1 nM E2 anti-estrogenic control

cocentration [M]	Replicate 1	Replicate 2	Replicate 3	Replicate 4	Mean
1,00E-06	4,92%	23,09%	21,51%	24,11%	**18,41%**

2nd experient

540nm

cocentration [M]	Replicate 1	Replicate 2	Replicate 3	Replicate 4
1,00E-10	1,082499981	1,109899998	1,150200009	1,184000015
1,00E-09	1,154700041	1,170699954	1,210999966	1,101899981
1,00E-08	1,144799948	1,096899986	1,214699984	1,215800047
1,00E-07	1,218199968	1,103000045	1,10800004	1,159999967
1,00E-06	1,063899994	1,180600047	1,193099976	1,16900003
1,00E-05	1,094000012	1,167299986	1,193600059	1,333699942
1,00E-04	1,118999958	1,069700003	1,059599996	1,006500006

690nm

cocentration [M]	Replicate 1	Replicate 2	Replicate 3	Replicate 4
1,00E-10	0,66930002	0,680499971	0,753400028	0,749199986
1,00E-09	0,76789999	0,722800016	0,788200021	0,677200019
1,00E-08	0,718900025	0,672800004	0,781599998	0,79519999
1,00E-07	0,800800025	0,676100016	0,689400017	0,749899983
1,00E-06	0,630599976	0,747600019	0,75819999	0,717199981
1,00E-05	0,658999979	0,707400024	0,778199971	0,840799987
1,00E-04	0,662800014	0,65079999	0,703800023	0,639400005

solvent control 690nm

	Replicate 1	Replicate 2	Replicate 3	Replicate 4
	0,649800003	0,749599993	0,73180002	0,812099993

540-690nm

cocentration [M]	Replicate 1	Replicate 2	Replicate 3	Replicate 4
1,00E-10	0,413100004	0,429399997	0,396800011	0,434799999
1,00E-09	0,386799991	0,447899997	0,422899991	0,424699992
1,00E-08	0,425900012	0,424100012	0,433099985	0,420599997
1,00E-07	0,417400002	0,426899999	0,418599993	0,410100013
1,00E-06	0,433299989	0,433099985	0,434899986	0,451799989
1,00E-05	0,435900003	0,459899992	0,415499985	0,492900014
1,00E-04	0,456200004	0,418900013	0,355800003	0,3671

solvent control 540nm-690nm

	Replicate 1	Replicate 2	Replicate 3	Replicate 4
	0,125300005	0,146200001	0,137500003	0,141100004

(540nm-690nm)-solvent control (540nm-690nm)

cocentration [M]	Replicate 1	Replicate 2	Replicate 3	Replicate 4
1,00E-10	0,287799999	0,283199996	0,259300008	0,293699995
1,00E-09	0,261499986	0,301699996	0,285399988	0,283599988
1,00E-08	0,300600007	0,27790001	0,295599982	0,279499993
1,00E-07	0,292099997	0,280699998	0,28109999	0,269000009
1,00E-06	0,307999983	0,286899984	0,297399983	0,310699984
1,00E-05	0,310599998	0,313699991	0,277999982	0,35180001
1,00E-04	0,330899999	0,272700012	0,2183	0,225999996

(540nm-690nm)-solvent control E2 reference

cocentration [M]	Replicate 1	Replicate 2	Replicate 3	Replicate 4
1,00E-09	0,28109999	0,352200016	0,36119999	0,35270001

Normalization (substance/1 nM E2)

cocentration [M]	Replicate 1	Replicate 2	Replicate 3	Replicate 4	Mean
1,00E-10	102,38%	80,41%	71,79%	83,27%	**84,46%**
1,00E-09	93,03%	85,66%	79,01%	80,41%	**84,53%**
1,00E-08	106,94%	78,90%	81,84%	79,25%	**86,73%**
1,00E-07	103,91%	79,70%	77,82%	76,27%	**84,43%**
1,00E-06	109,57%	81,46%	82,34%	88,09%	**90,36%**
1,00E-05	110,49%	89,07%	76,97%	99,74%	**94,07%**
1,00E-04	117,72%	77,43%	60,44%	64,08%	**79,91%**

yeast-growth/solubility 690nm/solvent control 690nm

cocentration [M]	Replicate 1	Replicate 2	Replicate 3	Replicate 4	Mean
1,00E-10	103,00%	90,78%	102,95%	92,25%	**97,25%**
1,00E-09	118,17%	96,42%	107,71%	83,39%	**101,42%**
1,00E-08	110,63%	89,75%	106,81%	97,92%	**101,28%**
1,00E-07	123,24%	90,19%	94,21%	92,34%	**99,99%**
1,00E-06	97,05%	99,73%	103,61%	88,31%	**97,18%**
1,00E-05	101,42%	94,37%	106,34%	103,53%	**101,42%**
1,00E-04	102,00%	86,82%	96,17%	78,73%	**90,93%**

4-HT (540nm-690nm) anti-estrogenic control

cocentration [M]	Replicate 1	Replicate 2	Replicate 3	Replicate 4
1,00E-06	0,067599997	0,059599996	0,029399991	0,048999995

4-HT (540nm-690nm)/1 nM E2 anti-estrogenic control

cocentration [M]	Replicate 1	Replicate 2	Replicate 3	Replicate 4	Mean
1,00E-06	24,05%	16,92%	8,14%	13,89%	**15,75%**

3rd experiment

540nm

cocentration [M	Replicate 1	Replicate 2	Replicate 3	Replicate 4
1,00E-10	1,064900041	1,038300037	1,091899991	1,078099966
1,00E-09	1,002599955	1,080800056	1,018599987	1,064299941
1,00E-08	1,079100013	1,058799982	1,064200044	1,016499996
1,00E-07	1,012699962	1,031399965	0,971000016	1,033699989
1,00E-06	1,002200007	1,013200045	1,033499956	1,03489995
1,00E-05	1,00909996	0,957099974	1,060299993	1,00880003
1,00E-04	0,955900013	0,935500026	0,973699987	0,9727

690nm

cocentration [M	Replicate 1	Replicate 2	Replicate 3	Replicate 4
1,00E-10	0,660600007	0,634500027	0,680400014	0,666700006
1,00E-09	0,596499979	0,692300022	0,648999989	0,679499984
1,00E-08	0,677600026	0,672800004	0,679700017	0,643700004
1,00E-07	0,613699973	0,642700016	0,60680002	0,666700006
1,00E-06	0,564400017	0,628799975	0,654200017	0,648999989
1,00E-05	0,639400005	0,59009999	0,665700018	0,633000016
1,00E-04	0,571399987	0,574400008	0,601100028	0,583199978

solvent control 690nm

	Replicate 1	Replicate 2	Replicate 3	Replicate 4
	0,458999991	0,599300027	0,644500017	0,652700007

540-690nm

cocentration [M	Replicate 1	Replicate 2	Replicate 3	Replicate 4
1,00E-10	0,404300004	0,403800011	0,411500007	0,41139999
1,00E-09	0,406100005	0,388500005	0,369700015	0,384799987
1,00E-08	0,401499987	0,386000007	0,384499997	0,372799993
1,00E-07	0,398999989	0,388700008	0,364199996	0,367000014
1,00E-06	0,43779999	0,38440001	0,379299998	0,385899991
1,00E-05	0,369700015	0,366899997	0,394600004	0,375800014
1,00E-04	0,384499997	0,361000001	0,372599989	0,389499992

solvent control 540nm-690nm

	Replicate 1	Replicate 2	Replicate 3	Replicate 4
	0,125499994	0,140400007	0,151700005	0,151299998

(540nm-690nm)-solvent control (540nm-690nm)

cocentration [M	Replicate 1	Replicate 2	Replicate 3	Replicate 4
1,00E-10	0,278800011	0,263400003	0,259800002	0,260099992
1,00E-09	0,280600011	0,248099998	0,21800001	0,233499989
1,00E-08	0,275999993	0,2456	0,232799992	0,221499994
1,00E-07	0,273499995	0,248300001	0,212499991	0,215700015
1,00E-06	0,312299997	0,244000003	0,227599993	0,234599993
1,00E-05	0,244200021	0,22649999	0,242899999	0,224500015
1,00E-04	0,259000003	0,220599994	0,220899984	0,238199994

(540nm-690nm)-solvent control E2 reference

cocentration [M	Replicate 1	Replicate 2	Replicate 3	Replicate 4
1,00E-09	0,304000005	0,229799986	0,252800018	0,23560001

Normalization (substance/1 nM E2)

cocentration [M	Replicate 1	Replicate 2	Replicate 3	Replicate 4	Mean
1,00E-10	91,71%	114,62%	102,77%	110,40%	**104,87%**
1,00E-09	92,30%	107,96%	86,23%	99,11%	**96,40%**
1,00E-08	90,79%	106,88%	92,09%	94,02%	**95,94%**
1,00E-07	89,97%	108,05%	84,06%	91,55%	**93,41%**
1,00E-06	102,73%	106,18%	90,03%	99,58%	**99,63%**
1,00E-05	80,33%	98,56%	96,08%	95,29%	**92,57%**
1,00E-04	85,20%	96,00%	87,38%	101,10%	**92,42%**

yeast-growth/solubility 690nm/solvent control 690nm

cocentration [M	Replicate 1	Replicate 2	Replicate 3	Replicate 4	Mean
1,00E-10	143,92%	105,87%	105,57%	102,14%	**114,38%**
1,00E-09	129,96%	115,52%	100,70%	104,11%	**112,57%**
1,00E-08	147,63%	112,26%	105,46%	98,62%	**115,99%**
1,00E-07	133,70%	107,24%	94,15%	102,14%	**109,31%**
1,00E-06	122,96%	104,92%	101,51%	99,43%	**107,21%**
1,00E-05	139,30%	98,46%	103,29%	96,98%	**109,51%**
1,00E-04	124,49%	95,85%	93,27%	89,35%	**100,74%**

4-HT (540nm-690nm) anti-estrogenic control

cocentration [M	Replicate 1	Replicate 2	Replicate 3	Replicate 4
1,00E-06	0,088800013	0,083499998	0,076700002	0,1303

4-HT (540nm-690nm)/1 nM E2 anti-estrogenic control

cocentration [M	Replicate 1	Replicate 2	Replicate 3	Replicate 4	Mean
1,00E-06	29,21%	36,34%	30,34%	55,31%	**37,80%**

4th experiment

540nm

cocentration [M	Replicate 1	Replicate 2	Replicate 3	Replicate 4
1,00E-10	1,286499977	1,26789999	1,234599948	1,33010006
1,00E-09	1,210100055	1,242799997	1,204300046	1,303500056
1,00E-08	1,246399999	1,285400033	1,220900059	1,313699961
1,00E-07	1,298599958	1,293900013	1,284500023	1,219599962
1,00E-06	1,3398	1,302399993	1,284700036	1,268100023
1,00E-05	1,330199957	1,308300018	1,287799954	1,329900026
1,00E-04	1,43840003	1,3204	1,217399955	1,289800048

690nm

cocentration [M	Replicate 1	Replicate 2	Replicate 3	Replicate 4
1,00E-10	0,717100024	0,699699998	0,686500013	0,745299995
1,00E-09	0,653199971	0,677200019	0,688799977	0,760900021
1,00E-08	0,676900029	0,665899992	0,700800002	0,774500012
1,00E-07	0,690599978	0,710600019	0,661899984	0,68809998
1,00E-06	0,680599988	0,701399982	0,707499981	0,733699977
1,00E-05	0,681299984	0,687099993	0,698400021	0,755100012
1,00E-04	0,64289999	0,67839998	0,648999989	0,708800018

solvent control 690nm

	Replicate 1	Replicate 2	Replicate 3	Replicate 4
	0,874100029	0,876600027	0,713400006	0,852699995

540-690nm

cocentration [M	Replicate 1	Replicate 2	Replicate 3	Replicate 4
1,00E-10	0,569500029	0,568199992	0,548099995	0,584800005
1,00E-09	0,556999981	0,565599978	0,515500009	0,542500019
1,00E-08	0,569500029	0,619499981	0,520099998	0,539200008
1,00E-07	0,60799998	0,583299994	0,551599979	0,531499982
1,00E-06	0,659099996	0,601000011	0,577199996	0,534300029
1,00E-05	0,648999989	0,621200025	0,589399993	0,574899971
1,00E-04	0,79549998	0,64200002	0,568400025	0,58099997

solvent control 540nm-690nm

	Replicate 1	Replicate 2	Replicate 3	Replicate 4
	0,157199994	0,159400001	0,149700001	0,157900006

(540nm-690nm)-solvent control (540nm-690nm)

cocentration [M	Replicate 1	Replicate 2	Replicate 3	Replicate 4
1,00E-10	0,412300035	0,408799991	0,398399994	0,426899999
1,00E-09	0,399799988	0,406199977	0,365800008	0,384600013
1,00E-08	0,412300035	0,46009998	0,370399997	0,381300002
1,00E-07	0,450799987	0,423899993	0,401899979	0,373599976
1,00E-06	0,501900002	0,44160001	0,427499995	0,376400024
1,00E-05	0,491799995	0,461800024	0,439699993	0,416999966
1,00E-04	0,638299987	0,482600018	0,418700024	0,423099965

(540nm-690nm)-solvent control E2 reference

cocentration [M	Replicate 1	Replicate 2	Replicate 3	Replicate 4
1,00E-09	0,547500014	0,585800022	0,578599989	0,581199989

Normalization (substance/1 nM E2)

cocentration [M	Replicate 1	Replicate 2	Replicate 3	Replicate 4	Mean
1,00E-10	75,31%	69,78%	68,86%	73,45%	**71,85%**
1,00E-09	73,02%	69,34%	63,22%	66,17%	**67,94%**
1,00E-08	75,31%	78,54%	64,02%	65,61%	**70,87%**
1,00E-07	82,34%	72,36%	69,46%	64,28%	**72,11%**
1,00E-06	91,67%	75,38%	73,89%	64,76%	**76,43%**
1,00E-05	89,83%	78,83%	75,99%	71,75%	**79,10%**
1,00E-04	116,58%	82,38%	72,36%	72,80%	**86,03%**

yeast-growth/solubility 690nm/solvent control 690nm

cocentration [M	Replicate 1	Replicate 2	Replicate 3	Replicate 4	Mean
1,00E-10	82,04%	79,82%	96,23%	87,40%	**86,37%**
1,00E-09	74,73%	77,25%	96,55%	89,23%	**84,44%**
1,00E-08	77,44%	75,96%	98,23%	90,83%	**85,62%**
1,00E-07	79,01%	81,06%	92,78%	80,70%	**83,39%**
1,00E-06	77,86%	80,01%	99,17%	86,04%	**85,77%**
1,00E-05	77,94%	78,38%	97,90%	88,55%	**85,69%**
1,00E-04	73,55%	77,39%	90,97%	83,12%	**81,26%**

4-HT (540nm-690nm) anti-estrogenic control

cocentration [M	Replicate 1	Replicate 2	Replicate 3	Replicate 4
1,00E-06	0,097500011	0,11940001	0,123199984	0,12379998

4-HT (540nm-690nm)/1 nM E2 anti-estrogenic control

cocentration [M	Replicate 1	Replicate 2	Replicate 3	Replicate 4	Mean
1,00E-06	17,81%	20,38%	21,29%	21,30%	**20,20%**

5th experiment

540nm

cocentration [M	Replicate 1	Replicate 2	Replicate 3	Replicate 4
1,00E-10	1,23029995	1,248600006	1,29429996	1,215999961
1,00E-09	1,23150003	1,205800056	1,264999986	1,266800046
1,00E-08	1,156499982	1,226400018	1,241000056	1,268599987
1,00E-07	1,17840004	1,220700026	1,219900012	1,180799961
1,00E-06	1,206400037	1,219799995	1,212499976	1,194100022
1,00E-05	1,218999982	1,174999952	1,203500032	1,203699946
1,00E-04	1,165099978	1,179399967	1,175400019	1,194499969

690nm

cocentration [M	Replicate 1	Replicate 2	Replicate 3	Replicate 4
1,00E-10	0,714399993	0,720899999	0,749199986	0,690400004
1,00E-09	0,711799979	0,69569999	0,742200017	0,736299992
1,00E-08	0,659399986	0,715499997	0,717100024	0,744300008
1,00E-07	0,677299976	0,707300007	0,707300007	0,676999986
1,00E-06	0,697700024	0,700399995	0,708299994	0,690999985
1,00E-05	0,694299996	0,677200019	0,684400022	0,693799973
1,00E-04	0,669799984	0,678200006	0,678799987	0,685299993

solvent control 690nm

	Replicate 1	Replicate 2	Replicate 3	Replicate 4
	0,909699976	0,92900002	0,960300028	0,660899997

540-690nm

cocentration [M	Replicate 1	Replicate 2	Replicate 3	Replicate 4
1,00E-10	0,515900016	0,527800024	0,545099974	0,525600016
1,00E-09	0,519699991	0,510200024	0,522800028	0,530499995
1,00E-08	0,497099996	0,510900021	0,523999989	0,524299979
1,00E-07	0,501100004	0,513400018	0,512600005	0,503799975
1,00E-06	0,508700013	0,519400001	0,504199982	0,503099978
1,00E-05	0,524699986	0,497799993	0,51910001	0,509899974
1,00E-04	0,495299995	0,50120002	0,496600002	0,509199977

solvent control 540nm-690nm

	Replicate 1	Replicate 2	Replicate 3	Replicate 4
	0,146799996	0,154899999	0,149900004	0,132300004

(540nm-690nm)-solvent control (540nm-690nm)

cocentration [M	Replicate 1	Replicate 2	Replicate 3	Replicate 4
1,00E-10	0,369100019	0,372900024	0,395199969	0,393300012
1,00E-09	0,372899994	0,355300024	0,372900024	0,39819999
1,00E-08	0,350299999	0,356000021	0,374099985	0,391999975
1,00E-07	0,354300007	0,358500019	0,3627	0,371499971
1,00E-06	0,361900017	0,364500001	0,354299977	0,370799974
1,00E-05	0,377899989	0,342899993	0,369200006	0,37759997
1,00E-04	0,348499998	0,346300021	0,346699998	0,376899973

(540nm-690nm)-solvent control E2 reference

cocentration [M	Replicate 1	Replicate 2	Replicate 3	Replicate 4
1,00E-09	0,498999983	0,503600016	0,50999999	0,501399994

Normalization (substance/1 nM E2)

cocentration [M	Replicate 1	Replicate 2	Replicate 3	Replicate 4	Mean
1,00E-10	73,97%	74,05%	77,49%	78,44%	**75,99%**
1,00E-09	74,73%	70,55%	73,12%	79,42%	**74,45%**
1,00E-08	70,20%	70,69%	73,35%	78,18%	**73,11%**
1,00E-07	71,00%	71,19%	71,12%	74,09%	**71,85%**
1,00E-06	72,53%	72,38%	69,47%	73,95%	**72,08%**
1,00E-05	75,73%	68,09%	72,39%	75,31%	**72,88%**
1,00E-04	69,84%	68,76%	67,98%	75,17%	**70,44%**

yeast-growth/solubility 690nm/solvent control 690nm

cocentration [M	Replicate 1	Replicate 2	Replicate 3	Replicate 4	Mean
1,00E-10	78,53%	77,60%	78,02%	104,46%	**84,65%**
1,00E-09	78,25%	74,89%	77,29%	111,41%	**85,46%**
1,00E-08	72,49%	77,02%	74,67%	112,62%	**84,20%**
1,00E-07	74,45%	76,14%	73,65%	102,44%	**81,67%**
1,00E-06	76,70%	75,39%	73,76%	104,55%	**82,60%**
1,00E-05	76,32%	72,90%	71,27%	104,98%	**81,37%**
1,00E-04	73,63%	73,00%	70,69%	103,69%	**80,25%**

4-HT (540nm-690nm) anti-estrogenic control

cocentration [M	Replicate 1	Replicate 2	Replicate 3	Replicate 4
1,00E-06	0,091200009	0,095300004	0,096199989	0,111399993

4-HT (540nm-690nm)/1 nM E2 anti-estrogenic control

cocentration [M	Replicate 1	Replicate 2	Replicate 3	Replicate 4	Mean
1,00E-06	18,28%	18,92%	18,86%	22,22%	**19,57%**

6th experiment

540nm

cocentration [M	Replicate 1	Replicate 2	Replicate 3	Replicate 4
1,00E-10	1,263700008	1,280300021	1,258900046	1,303500056
1,00E-09	1,291599989	1,315000057	1,307899952	1,239199996
1,00E-08	1,306800008	1,266899943	1,268900037	1,295300007
1,00E-07	1,298200011	1,243399978	1,273699999	1,255399942
1,00E-06	1,261299968	1,326400042	1,274700046	1,271299958
1,00E-05	1,34070003	1,366899967	1,321200013	1,29610002
1,00E-04	1,218600035	1,241400003	1,173099995	1,25909996

690nm

cocentration [M	Replicate 1	Replicate 2	Replicate 3	Replicate 4
1,00E-10	0,640500009	0,648000002	0,638700008	0,657299995
1,00E-09	0,658900023	0,664399981	0,660399973	0,603299975
1,00E-08	0,655399978	0,617600024	0,634199977	0,648599982
1,00E-07	0,648599982	0,602699995	0,630599976	0,618700027
1,00E-06	0,611999989	0,662299991	0,616100013	0,623899996
1,00E-05	0,663500011	0,701499999	0,685299993	0,636900008
1,00E-04	0,602500021	0,613600016	0,570299983	0,62620002

solvent control 690nm

	Replicate 1	Replicate 2	Replicate 3	Replicate 4
	0,641200006	0,639500022	0,598500013	0,620800018

540-690nm

cocentration [M	Replicate 1	Replicate 2	Replicate 3	Replicate 4
1,00E-10	0,623199999	0,632300019	0,620100021	0,646200001
1,00E-09	0,632700026	0,650600016	0,647499979	0,635900021
1,00E-08	0,651499987	0,649299979	0,6347	0,646700025
1,00E-07	0,649600029	0,640699983	0,643100023	0,636699975
1,00E-06	0,649299979	0,664099991	0,658599973	0,647400022
1,00E-05	0,677200019	0,665499985	0,635900021	0,659099996
1,00E-04	0,615999997	0,627799988	0,602900028	0,632799983

solvent control 540nm-690nm

	Replicate 1	Replicate 2	Replicate 3	Replicate 4
	0,136000007	0,138699993	0,128999993	0,133599997

(540nm-690nm)-solvent control (540nm-690nm)

cocentration [M	Replicate 1	Replicate 2	Replicate 3	Replicate 4
1,00E-10	0,487199992	0,493600026	0,491100028	0,512600005
1,00E-09	0,496700019	0,511900023	0,518499985	0,502300024
1,00E-08	0,515499979	0,510599986	0,505700007	0,513100028
1,00E-07	0,513600022	0,501999989	0,51410003	0,503099978
1,00E-06	0,513299972	0,525399998	0,52959998	0,513800025
1,00E-05	0,541200012	0,526799992	0,506900027	0,5255
1,00E-04	0,479999989	0,489099994	0,473900035	0,499199986

(540nm-690nm)-solvent control E2 reference

cocentration [M	Replicate 1	Replicate 2	Replicate 3	Replicate 4
1,00E-09	0,683299989	0,639200017	0,670399994	0,671300024

Normalization (substance/1 nM E2)

cocentration [M	Replicate 1	Replicate 2	Replicate 3	Replicate 4	Mean
1,00E-10	71,30%	77,22%	73,25%	76,36%	**74,53%**
1,00E-09	72,69%	80,08%	77,34%	74,82%	**76,24%**
1,00E-08	75,44%	79,88%	75,43%	76,43%	**76,80%**
1,00E-07	75,16%	78,54%	76,69%	74,94%	**76,33%**
1,00E-06	75,12%	82,20%	79,00%	76,54%	**78,21%**
1,00E-05	79,20%	82,42%	75,61%	78,28%	**78,88%**
1,00E-04	70,25%	76,52%	70,69%	74,36%	**72,95%**

yeast-growth/solubility 690nm/solvent control 690nm

cocentration [M	Replicate 1	Replicate 2	Replicate 3	Replicate 4	Mean
1,00E-10	99,89%	101,33%	106,72%	105,88%	**103,45%**
1,00E-09	102,76%	103,89%	110,34%	97,18%	**103,54%**
1,00E-08	102,21%	96,58%	105,96%	104,48%	**102,31%**
1,00E-07	101,15%	94,25%	105,36%	99,66%	**100,11%**
1,00E-06	95,45%	103,57%	102,94%	100,50%	**100,61%**
1,00E-05	103,48%	109,70%	114,50%	102,59%	**107,57%**
1,00E-04	93,96%	95,95%	95,29%	100,87%	**96,52%**

4-HT (540nm-690nm) anti-estrogenic control

cocentration [M	Replicate 1	Replicate 2	Replicate 3	Replicate 4
1,00E-06	0,147199988	0,128200009	0,156000003	0,147300005

4-HT (540nm-690nm)/1 nM E2 anti-estrogenic control

cocentration [M	Replicate 1	Replicate 2	Replicate 3	Replicate 4	Mean
1,00E-06	21,54%	20,06%	23,27%	21,94%	**21,70%**

7th experiment

540nm

cocentration [M	Replicate 1	Replicate 2	Replicate 3	Replicate 4
1,00E-10	1,334200025	1,303300023	1,327000022	1,296800017
1,00E-09	1,33130002	1,29279995	1,326300025	1,331900001
1,00E-08	1,404099941	1,400999993	1,421200037	1,364199996
1,00E-07	1,382200003	1,391499996	1,368299961	1,380100012
1,00E-06	1,346199989	1,363499999	1,422799945	1,363700032
1,00E-05	1,385800004	1,396299958	1,380599976	1,386700034
1,00E-04	1,495800018	1,34010005	1,42900002	1,400099993

690nm

cocentration [M	Replicate 1	Replicate 2	Replicate 3	Replicate 4
1,00E-10	0,69569999	0,661199987	0,677299976	0,654500008
1,00E-09	0,695299983	0,652800024	0,66839999	0,672500014
1,00E-08	0,730599999	0,721000016	0,731299996	0,693700016
1,00E-07	0,695599973	0,692600012	0,682799995	0,691100001
1,00E-06	0,654399991	0,664900005	0,698400021	0,656799972
1,00E-05	0,667299986	0,675199986	0,66049999	0,658299983
1,00E-04	0,781300008	0,627200007	0,689100027	0,675000012

solvent control 690nm

	Replicate 1	Replicate 2	Replicate 3	Replicate 4
	0,719200015	0,784500003	0,787699997	0,712000012

540-690nm

cocentration [M	Replicate 1	Replicate 2	Replicate 3	Replicate 4
1,00E-10	0,638599992	0,642099977	0,649699986	0,64230001
1,00E-09	0,635900021	0,639999986	0,657899976	0,659399986
1,00E-08	0,673500001	0,67900002	0,689899981	0,67049998
1,00E-07	0,686600029	0,698899984	0,685400009	0,68900001
1,00E-06	0,691799998	0,698599994	0,724399987	0,706900001
1,00E-05	0,718400002	0,721199989	0,720099986	0,728500009
1,00E-04	0,71450001	0,712899983	0,739899993	0,725099981

solvent control 540nm-690nm

	Replicate 1	Replicate 2	Replicate 3	Replicate 4
	0,161200002	0,160999998	0,160899997	0,155000001

(540nm-690nm)-solvent control (540nm-690nm)

cocentration [M	Replicate 1	Replicate 2	Replicate 3	Replicate 4
1,00E-10	0,47739999	0,481099978	0,488799989	0,487300009
1,00E-09	0,474700019	0,478999987	0,496999979	0,504399985
1,00E-08	0,5123	0,518000022	0,528999984	0,515499979
1,00E-07	0,525400028	0,537899986	0,524500012	0,534000009
1,00E-06	0,530599996	0,537599996	0,563499987	0,551899999
1,00E-05	0,5572	0,560199991	0,559199989	0,573500007
1,00E-04	0,553300008	0,551899984	0,578999996	0,57009998

(540nm-690nm)-solvent control E2 reference

cocentration [M	Replicate 1	Replicate 2	Replicate 3	Replicate 4
1,00E-09	0,700200014	0,651600003	0,658099979	0,669500023

Normalization (substance/1 nM E2)

cocentration [M	Replicate 1	Replicate 2	Replicate 3	Replicate 4	Mean
1,00E-10	68,18%	73,83%	74,27%	72,79%	**72,27%**
1,00E-09	67,79%	73,51%	75,52%	75,34%	**73,04%**
1,00E-08	73,16%	79,50%	80,38%	77,00%	**77,51%**
1,00E-07	75,04%	82,55%	79,70%	79,76%	**79,26%**
1,00E-06	75,78%	82,50%	85,63%	82,43%	**81,59%**
1,00E-05	79,58%	85,97%	84,97%	85,66%	**84,05%**
1,00E-04	79,02%	84,70%	87,98%	85,15%	**84,21%**

yeast-growth/solubility 690nm/solvent control 690nm

cocentration [M	Replicate 1	Replicate 2	Replicate 3	Replicate 4	Mean
1,00E-10	96,73%	84,28%	85,98%	91,92%	**89,73%**
1,00E-09	96,68%	83,21%	84,85%	94,45%	**89,80%**
1,00E-08	101,59%	91,91%	92,84%	97,43%	**95,94%**
1,00E-07	96,72%	88,29%	86,68%	97,06%	**92,19%**
1,00E-06	90,99%	84,75%	88,66%	92,25%	**89,16%**
1,00E-05	92,78%	86,07%	83,85%	92,46%	**88,79%**
1,00E-04	108,63%	79,95%	87,48%	94,80%	**92,72%**

4-HT (540nm-690nm) anti-estrogenic control

cocentration [M	Replicate 1	Replicate 2	Replicate 3	Replicate 4
1,00E-06	0,141100004	0,139599994	0,137199998	0,1514

4-HT (540nm-690nm)/1 nM E2 anti-estrogenic control

cocentration [M	Replicate 1	Replicate 2	Replicate 3	Replicate 4	Mean
1,00E-06	20,15%	21,42%	20,85%	22,61%	**21,26%**

8th experiment

540nm

cocentration [M	Replicate 1	Replicate 2	Replicate 3	Replicate 4
1,00E-10	1,105599999	1,100900054	1,081200004	1,06099999
1,00E-09	1,070899963	1,065400004	1,080199957	1,087599993
1,00E-08	1,042199969	1,075399995	1,047799945	1,029600024
1,00E-07	1,09739995	1,084200025	1,031599998	1,089599967
1,00E-06	1,067800045	1,079200029	1,029299974	1,034500003
1,00E-05	1,086400032	1,040699959	1,049700022	1,050400019
1,00E-04	1,068799973	1,081500053	1,071499944	1,036200047

690nm

cocentration [M	Replicate 1	Replicate 2	Replicate 3	Replicate 4
1,00E-10	0,646799982	0,646099985	0,629000008	0,613399982
1,00E-09	0,634000003	0,639800012	0,632700026	0,629899979
1,00E-08	0,622399986	0,638700008	0,608299971	0,590499997
1,00E-07	0,637700021	0,62620002	0,600600004	0,630699992
1,00E-06	0,626699984	0,633400023	0,588999987	0,608500004
1,00E-05	0,629000008	0,589600027	0,611999989	0,617600024
1,00E-04	0,592800021	0,617799997	0,627799988	0,613399982

solvent control 690nm

	Replicate 1	Replicate 2	Replicate 3	Replicate 4
	0,727299988	0,739700019	0,721000016	0,732800007

540-690nm

cocentration [M	Replicate 1	Replicate 2	Replicate 3	Replicate 4
1,00E-10	0,458700001	0,45480001	0,452199996	0,447600007
1,00E-09	0,43689999	0,425500005	0,44749999	0,457700014
1,00E-08	0,419800013	0,436699986	0,439599991	0,439200014
1,00E-07	0,459699988	0,458000004	0,430999994	0,458900005
1,00E-06	0,441000015	0,445800006	0,440400004	0,425900012
1,00E-05	0,457399994	0,451099992	0,43779999	0,432799995
1,00E-04	0,476000011	0,463699996	0,443699986	0,422899991

solvent control 540nm-690nm

	Replicate 1	Replicate 2	Replicate 3	Replicate 4
	0,1479	0,153099999	0,148200005	0,150700003

(540nm-690nm)-solvent control (540nm-690nm)

cocentration [M	Replicate 1	Replicate 2	Replicate 3	Replicate 4
1,00E-10	0,310800001	0,301700011	0,30399999	0,296900004
1,00E-09	0,2889999	0,272400007	0,299299985	0,307000011
1,00E-08	0,271900013	0,283599988	0,291399986	0,288500011
1,00E-07	0,311799988	0,304900005	0,282799989	0,308200002
1,00E-06	0,293100014	0,292700008	0,292199999	0,275200009
1,00E-05	0,309499994	0,297999993	0,289599985	0,282099992
1,00E-04	0,328100011	0,310599998	0,29549998	0,272199988

(540nm-690nm)-solvent control E2 reference

cocentration [M	Replicate 1	Replicate 2	Replicate 3	Replicate 4
1,00E-09	0,323899999	0,312899992	0,313000008	0,324100003

Normalization (substance/1 nM E2)

cocentration [M	Replicate 1	Replicate 2	Replicate 3	Replicate 4	Mean
1,00E-10	95,96%	96,42%	97,12%	91,61%	**95,28%**
1,00E-09	89,23%	87,06%	95,62%	94,72%	**91,66%**
1,00E-08	83,95%	90,64%	93,10%	89,02%	**89,17%**
1,00E-07	96,26%	97,44%	90,35%	95,09%	**94,79%**
1,00E-06	90,49%	93,54%	93,35%	84,91%	**90,58%**
1,00E-05	95,55%	95,24%	92,52%	87,04%	**92,59%**
1,00E-04	101,30%	99,26%	94,41%	83,99%	**94,74%**

yeast-growth/solubility 690nm/solvent control 690nm

cocentration [M	Replicate 1	Replicate 2	Replicate 3	Replicate 4	Mean
1,00E-10	88,93%	87,35%	87,24%	83,71%	**86,81%**
1,00E-09	87,17%	86,49%	87,75%	85,96%	**86,84%**
1,00E-08	85,58%	86,35%	84,37%	80,58%	**84,22%**
1,00E-07	87,68%	84,66%	83,30%	86,07%	**85,43%**
1,00E-06	86,17%	85,63%	81,69%	83,04%	**84,13%**
1,00E-05	86,48%	79,71%	84,88%	84,28%	**83,84%**
1,00E-04	81,51%	83,52%	87,07%	83,71%	**83,95%**

4-HT (540nm-690nm) anti-estrogenic control

cocentration [M	Replicate 1	Replicate 2	Replicate 3	Replicate 4
1,00E-06	0,086600006	0,089100003	0,098799989	0,088499993

4-HT (540nm-690nm)/1 nM E2 anti-estrogenic control

cocentration [M	Replicate 1	Replicate 2	Replicate 3	Replicate 4	Mean
1,00E-06	26,74%	28,48%	31,57%	27,31%	**28,52%**

YES propamocarb + 1nM E2

1st experiment

540nm

cocentration [M]	Replicate 1	Replicate 2	Replicate 3	Replicate 4
1,00E-10	1,030099988	1,038200021	0,964299977	0,993900001
1,00E-09	1,058099985	1,034999967	1,004899979	1,078999996
1,00E-08	1,081799984	1,09829998	1,034100056	1,081400037
1,00E-07	1,01820004	1,048400044	1,020699978	1,014000058
1,00E-06	1,03579998	1,056699991	1,00940001	1,102499962
1,00E-05	1,026900053	1,089300036	1,019999981	0,990999997
1,00E-04	1,101400018	1,059299946	1,054999948	1,065600038

690nm

cocentration [M]	Replicate 1	Replicate 2	Replicate 3	Replicate 4
1,00E-10	0,631200016	0,641499996	0,582099974	0,608500004
1,00E-09	0,650099993	0,631699979	0,613799989	0,665199995
1,00E-08	0,684000015	0,68900001	0,629800022	0,678799987
1,00E-07	0,611899972	0,639699996	0,611299992	0,610800028
1,00E-06	0,632200003	0,631799996	0,602500021	0,680000007
1,00E-05	0,577499986	0,667400002	0,591000021	0,563499987
1,00E-04	0,644400001	0,612800002	0,609799981	0,6153

solvent control 690nm

	Replicate 1	Replicate 2	Replicate 3	Replicate 4
	0,642700016	0,629999995	0,611800015	0,60769999

540-690nm

cocentration [M]	Replicate 1	Replicate 2	Replicate 3	Replicate 4
1,00E-10	0,398900002	0,396699995	0,382099986	0,385399997
1,00E-09	0,407999992	0,403299987	0,391200006	0,413800001
1,00E-08	0,397799999	0,409200013	0,404300004	0,40259999
1,00E-07	0,406300008	0,408800006	0,409399986	0,403200001
1,00E-06	0,403600007	0,425000012	0,406800002	0,422600001
1,00E-05	0,449400008	0,421900004	0,429100007	0,427599996
1,00E-04	0,457100004	0,446500003	0,445199996	0,450300008

solvent control 540nm-690nm

	Replicate 1	Replicate 2	Replicate 3	Replicate 4
	0,141200006	0,1373	0,140300006	0,143399999

(540nm-690nm)-solvent control (540nm-690nm)

cocentration [M]	Replicate 1	Replicate 2	Replicate 3	Replicate 4
1,00E-10	0,257699996	0,259399995	0,24179998	0,241999999
1,00E-09	0,266799996	0,265999988	0,2509	0,270400003
1,00E-08	0,256599993	0,271900013	0,263999999	0,259199992
1,00E-07	0,265100002	0,271500006	0,269099981	0,259800002
1,00E-06	0,262400001	0,287700012	0,266499996	0,279200003
1,00E-05	0,308200002	0,284600005	0,288800001	0,284199998
1,00E-04	0,315899998	0,309200004	0,304899991	0,30690001

(540nm-690nm)-solvent control E2 reference

cocentration [M]	Replicate 1	Replicate 2	Replicate 3	Replicate 4
1,00E-09	0,3398	0,291799992	0,272400007	0,287199989

Normalization (substance/1 nM E2)

cocentration [M]	Replicate 1	Replicate 2	Replicate 3	Replicate 4	Mean
1,00E-10	75,84%	88,90%	88,77%	84,26%	**84,44%**
1,00E-09	78,52%	91,16%	92,11%	94,15%	**88,98%**
1,00E-08	75,52%	93,18%	96,92%	90,25%	**88,97%**
1,00E-07	78,02%	93,04%	98,79%	90,46%	**90,08%**
1,00E-06	77,22%	98,59%	97,83%	97,21%	**92,72%**
1,00E-05	90,70%	97,53%	106,02%	98,96%	**98,30%**
1,00E-04	92,97%	105,96%	111,93%	106,86%	**104,43%**

yeast-growth/solubility 690nm/solvent control 690nm

cocentration [M]	Replicate 1	Replicate 2	Replicate 3	Replicate 4	Mean
1,00E-10	98,21%	101,83%	95,15%	100,13%	**98,83%**
1,00E-09	101,15%	100,27%	100,33%	109,46%	**102,80%**
1,00E-08	106,43%	109,37%	102,94%	111,70%	**107,61%**
1,00E-07	95,21%	101,54%	99,92%	100,51%	**99,29%**
1,00E-06	89,86%	105,94%	96,60%	92,73%	**96,28%**
1,00E-05	98,37%	100,29%	98,48%	111,90%	**102,26%**
1,00E-04	100,26%	97,27%	99,67%	101,25%	**99,61%**

4-HT (540nm-690nm) anti-estrogenic control

cocentration [M]	Replicate 1	Replicate 2	Replicate 3	Replicate 4
1,00E-06	0,054899991	0,066699997	0,065399989	0,063600004

4-HT (540nm-690nm)/1 nM E2 anti-estrogenic control

cocentration [M]	Replicate 1	Replicate 2	Replicate 3	Replicate 4	Mean
1,00E-06	16,16%	22,86%	24,01%	22,14%	**21,29%**

2nd experient

540nm

cocentration [M]	Replicate 1	Replicate 2	Replicate 3	Replicate 4
1,00E-10	1,094200015	1,039399981	1,10710001	0,996699989
1,00E-09	1,11500001	1,163200021	1,080199957	1,093799949
1,00E-08	1,170099974	1,193799973	1,166900039	1,147099972
1,00E-07	1,178900003	1,116299987	1,179399967	1,162700057
1,00E-06	1,159800053	1,137400031	1,162099957	1,102900028
1,00E-05	1,147500038	1,09920001	1,078400016	1,073099971
1,00E-04	1,079300046	1,132099986	0,997900009	1,228899956

690nm

cocentration [M]	Replicate 1	Replicate 2	Replicate 3	Replicate 4
1,00E-10	0,645099998	0,606400013	0,663299978	0,576799989
1,00E-09	0,667500019	0,692600012	0,636799991	0,647000015
1,00E-08	0,695900023	0,704299986	0,693700016	0,675700009
1,00E-07	0,692799985	0,661300004	0,690400004	0,696500003
1,00E-06	0,64319998	0,665099978	0,694400012	0,638899982
1,00E-05	0,680599988	0,630999982	0,618300021	0,624899983
1,00E-04	0,582099974	0,636300027	0,536300004	0,735899985

solvent control 690nm

	Replicate 1	Replicate 2	Replicate 3	Replicate 4
	0,531199992	0,46540001	0,494700015	0,508599997

540-690nm

cocentration [M]	Replicate 1	Replicate 2	Replicate 3	Replicate 4
1,00E-10	0,449099988	0,432999998	0,443800002	0,4199
1,00E-09	0,44749999	0,470699996	0,443399996	0,446799994
1,00E-08	0,47420001	0,489499986	0,473199993	0,471300006
1,00E-07	0,486099988	0,455000013	0,488999993	0,466199994
1,00E-06	0,516600013	0,472299993	0,467799991	0,463999987
1,00E-05	0,466899991	0,468199998	0,460099995	0,448199987
1,00E-04	0,497099996	0,495799989	0,461600006	0,493099988

solvent control 540nm-690nm

	Replicate 1	Replicate 2	Replicate 3	Replicate 4
	0,127499998	0,118600003	0,122599997	0,125300005

(540nm-690nm)-solvent control (540nm-690nm)

cocentration [M]	Replicate 1	Replicate 2	Replicate 3	Replicate 4
1,00E-10	0,32159999	0,314399995	0,321200006	0,294599995
1,00E-09	0,319999993	0,352099992	0,320799999	0,321499988
1,00E-08	0,346700013	0,370899983	0,350599997	0,346000001
1,00E-07	0,358599991	0,33640001	0,366399996	0,340899989
1,00E-06	0,389100015	0,35369999	0,345199995	0,338699982
1,00E-05	0,339399993	0,349599995	0,337499999	0,322899982
1,00E-04	0,369599998	0,377199985	0,339000009	0,367799982

(540nm-690nm)-solvent control E2 reference

cocentration [M]	Replicate 1	Replicate 2	Replicate 3	Replicate 4
1,00E-09	0,273800001	0,321899995	0,327999994	0,322899997

Normalization (substance/1 nM E2)

cocentration [M]	Replicate 1	Replicate 2	Replicate 3	Replicate 4	Mean
1,00E-10	117,46%	97,67%	97,93%	91,24%	**101,07%**
1,00E-09	116,87%	109,38%	97,80%	99,57%	**105,91%**
1,00E-08	126,63%	115,22%	106,89%	107,15%	**113,97%**
1,00E-07	130,97%	104,50%	111,71%	105,57%	**113,19%**
1,00E-06	142,11%	109,88%	105,24%	104,89%	**115,53%**
1,00E-05	123,96%	108,61%	102,90%	100,00%	**108,87%**
1,00E-04	134,99%	117,18%	103,35%	113,91%	**117,36%**

yeast-growth/solubility 690nm/solvent control 690nm

cocentration [M]	Replicate 1	Replicate 2	Replicate 3	Replicate 4	Mean
1,00E-10	121,44%	130,30%	134,08%	113,41%	**124,81%**
1,00E-09	125,66%	148,82%	128,72%	127,21%	**132,60%**
1,00E-08	131,01%	151,33%	140,23%	132,85%	**138,85%**
1,00E-07	130,42%	142,09%	139,56%	136,94%	**137,25%**
1,00E-06	121,08%	142,91%	140,37%	125,62%	**132,50%**
1,00E-05	128,12%	135,58%	124,98%	122,87%	**127,89%**
1,00E-04	109,58%	136,72%	108,41%	144,69%	**124,85%**

4-HT (540nm-690nm) anti-estrogenic control

cocentration [M]	Replicate 1	Replicate 2	Replicate 3	Replicate 4
1,00E-06	0,075900003	0,071000002	0,072900005	0,062299997

4-HT (540nm-690nm)/1 nM E2 anti-estrogenic control

cocentration [M]	Replicate 1	Replicate 2	Replicate 3	Replicate 4	Mean
1,00E-06	27,72%	22,06%	22,23%	19,29%	**22,82%**

3rd experiment

540nm

cocentration [M	Replicate 1	Replicate 2	Replicate 3	Replicate 4
1,00E-10	1,081099987	1,074599981	1,055199981	1,068600059
1,00E-09	1,041300058	1,108999968	1,03610003	1,024199963
1,00E-08	0,985800028	1,122499943	1,071699977	1,061800003
1,00E-07	1,062700033	1,114400029	1,094099998	1,103199959
1,00E-06	1,052899957	1,072299957	1,078699946	1,114899993
1,00E-05	1,037799954	0,967000008	1,088099957	1,07249999
1,00E-04	0,997900009	0,861999989	1,017400026	0,970499992

690nm

cocentration [M	Replicate 1	Replicate 2	Replicate 3	Replicate 4
1,00E-10	0,652100027	0,662999988	0,644500017	0,652999997
1,00E-09	0,638400018	0,706099987	0,628400028	0,627499998
1,00E-08	0,602699995	0,704900026	0,662899971	0,666999996
1,00E-07	0,66170001	0,713999987	0,689599991	0,701699972
1,00E-06	0,646399975	0,671999991	0,683000028	0,720499992
1,00E-05	0,638899982	0,568000019	0,670099974	0,659399986
1,00E-04	0,578299999	0,461699992	0,565900028	0,555499971

solvent control 690nm

	Replicate 1	Replicate 2	Replicate 3	Replicate 4
	0,602900028	0,600799978	0,518299997	0,623199999

540-690nm

cocentration [M	Replicate 1	Replicate 2	Replicate 3	Replicate 4
1,00E-10	0,42899999	0,411599994	0,410699993	0,415600002
1,00E-09	0,40290001	0,40290001	0,407700002	0,396699995
1,00E-08	0,383100003	0,417600006	0,408800006	0,394899994
1,00E-07	0,400999993	0,400400013	0,404500008	0,401499987
1,00E-06	0,406500012	0,400200009	0,395700008	0,394499987
1,00E-05	0,398900002	0,399100006	0,418000013	0,413199991
1,00E-04	0,41960001	0,400299996	0,451499999	0,414999992

solvent control 540nm-690nm

	Replicate 1	Replicate 2	Replicate 3	Replicate 4
	0,143000007	0,138699993	0,1241	0,140599996

(540nm-690nm)-solvent control (540nm-690nm)

cocentration [M	Replicate 1	Replicate 2	Replicate 3	Replicate 4
1,00E-10	0,285999984	0,2729	0,286599994	0,275000006
1,00E-09	0,259900004	0,264200017	0,283600003	0,256099999
1,00E-08	0,240099996	0,278900012	0,284700006	0,254299998
1,00E-07	0,257999986	0,261700019	0,280400008	0,260899991
1,00E-06	0,263500005	0,261500016	0,271600008	0,253899992
1,00E-05	0,255899996	0,260400012	0,293900013	0,272599995
1,00E-04	0,276600003	0,261600003	0,327399999	0,274399996

(540nm-690nm)-solvent control E2 reference

cocentration [M	Replicate 1	Replicate 2	Replicate 3	Replicate 4
1,00E-09	0,272799984	0,2377	0,280699998	0,259600013

Normalization (substance/1 nM E2)

cocentration [M	Replicate 1	Replicate 2	Replicate 3	Replicate 4	Mean
1,00E-10	104,84%	114,81%	102,10%	105,93%	**106,92%**
1,00E-09	95,27%	111,15%	101,03%	98,65%	**101,53%**
1,00E-08	88,01%	117,33%	101,43%	97,96%	**101,18%**
1,00E-07	94,57%	110,10%	99,89%	100,50%	**101,27%**
1,00E-06	96,59%	110,01%	96,76%	97,80%	**100,29%**
1,00E-05	93,80%	109,55%	104,70%	105,01%	**103,27%**
1,00E-04	101,39%	110,05%	116,64%	105,70%	**108,45%**

yeast-growth/solubility 690nm/solvent control 690nm

cocentration [M	Replicate 1	Replicate 2	Replicate 3	Replicate 4	Mean
1,00E-10	108,16%	110,35%	124,35%	104,78%	**111,91%**
1,00E-09	105,89%	117,53%	121,24%	100,69%	**111,34%**
1,00E-08	99,97%	117,33%	127,90%	107,03%	**113,06%**
1,00E-07	109,75%	118,84%	133,05%	112,60%	**118,56%**
1,00E-06	107,22%	111,85%	131,78%	115,61%	**116,61%**
1,00E-05	105,97%	94,54%	129,29%	105,81%	**108,90%**
1,00E-04	95,92%	76,85%	109,18%	89,14%	**92,77%**

4-HT (540nm-690nm) anti-estrogenic control

cocentration [M	Replicate 1	Replicate 2	Replicate 3	Replicate 4
1,00E-06	0,075299993	0,094400004	0,096799999	0,078500003

4-HT (540nm-690nm)/1 nM E2 anti-estrogenic control

cocentration [M	Replicate 1	Replicate 2	Replicate 3	Replicate 4	Mean
1,00E-06	27,60%	39,71%	34,49%	30,24%	**33,01%**

4th experiment

540nm

cocentration [M	Replicate 1	Replicate 2	Replicate 3	Replicate 4
1,00E-10	1,343299985	1,3398	1,341799974	1,309700012
1,00E-09	1,319800019	1,405500054	1,307000041	1,317600012
1,00E-08	1,355399966	1,321499944	1,368399978	1,297600031
1,00E-07	1,390200019	1,281499982	1,370599985	1,361099958
1,00E-06	1,395499945	1,323899984	1,374500036	1,320199966
1,00E-05	1,329599977	1,369600058	1,368299961	1,356899977
1,00E-04	1,42750001	1,349300027	1,299499989	1,294399977

690nm

cocentration [M	Replicate 1	Replicate 2	Replicate 3	Replicate 4
1,00E-10	0,717800021	0,728200018	0,752300024	0,732500017
1,00E-09	0,699999988	0,760299981	0,693799973	0,731999993
1,00E-08	0,719399989	0,708899975	0,760599971	0,707499981
1,00E-07	0,767799973	0,674899995	0,746100008	0,739000022
1,00E-06	0,721400023	0,684199989	0,739499986	0,696399987
1,00E-05	0,637099981	0,70539999	0,712100029	0,702400029
1,00E-04	0,694800019	0,69599998	0,660399973	0,645099998

solvent control 690nm

	Replicate 1	Replicate 2	Replicate 3	Replicate 4
	0,721899986	0,768899977	0,748399973	0,86680001

540-690nm

cocentration [M	Replicate 1	Replicate 2	Replicate 3	Replicate 4
1,00E-10	0,625500023	0,611599982	0,589399993	0,577199996
1,00E-09	0,619799972	0,645200014	0,613200009	0,585600019
1,00E-08	0,635999978	0,612600029	0,607800007	0,59009999
1,00E-07	0,622399986	0,606700003	0,624499977	0,622099996
1,00E-06	0,674099982	0,639599979	0,63499999	0,62379998
1,00E-05	0,692499995	0,664200008	0,656099975	0,654500008
1,00E-04	0,73269999	0,653299987	0,639100015	0,649299979

solvent control 540nm-690nm

	Replicate 1	Replicate 2	Replicate 3	Replicate 4
	0,149700001	0,150000006	0,132400006	0,164000005

(540nm-690nm)-solvent control (540nm-690nm)

cocentration [M	Replicate 1	Replicate 2	Replicate 3	Replicate 4
1,00E-10	0,475800022	0,461599976	0,456999987	0,413199991
1,00E-09	0,470099971	0,495200008	0,480800003	0,421600014
1,00E-08	0,486299977	0,462600023	0,475400001	0,426099986
1,00E-07	0,472699985	0,456699997	0,492099971	0,458099991
1,00E-06	0,524399981	0,489599973	0,502599984	0,459799975
1,00E-05	0,542799994	0,514200002	0,523699969	0,490500003
1,00E-04	0,582999989	0,503299981	0,506700009	0,485299975

(540nm-690nm)-solvent control E2 reference

cocentration [M	Replicate 1	Replicate 2	Replicate 3	Replicate 4
1,00E-09	0,560300022	0,562099993	0,587200001	0,591399997

Normalization (substance/1 nM E2)

cocentration [M	Replicate 1	Replicate 2	Replicate 3	Replicate 4	Mean
1,00E-10	84,92%	82,12%	77,83%	69,87%	**78,68%**
1,00E-09	83,90%	88,10%	81,88%	71,29%	**81,29%**
1,00E-08	86,79%	82,30%	80,96%	72,05%	**80,53%**
1,00E-07	84,37%	81,25%	83,80%	77,46%	**81,72%**
1,00E-06	93,59%	87,10%	85,59%	77,75%	**86,01%**
1,00E-05	96,88%	91,48%	89,19%	82,94%	**90,12%**
1,00E-04	104,05%	89,54%	86,29%	82,06%	**90,49%**

yeast-growth/solubility 690nm/solvent control 690nm

cocentration [M	Replicate 1	Replicate 2	Replicate 3	Replicate 4	Mean
1,00E-10	99,43%	94,71%	100,52%	84,51%	**94,79%**
1,00E-09	96,97%	98,88%	92,70%	84,45%	**93,25%**
1,00E-08	99,65%	92,20%	101,63%	81,62%	**93,78%**
1,00E-07	106,36%	87,77%	99,69%	85,26%	**94,77%**
1,00E-06	99,93%	88,98%	98,81%	80,34%	**92,02%**
1,00E-05	88,25%	91,74%	95,15%	81,03%	**89,04%**
1,00E-04	96,25%	90,52%	88,24%	74,42%	**87,36%**

4-HT (540nm-690nm) anti-estrogenic control

cocentration [M	Replicate 1	Replicate 2	Replicate 3	Replicate 4
1,00E-06	0,115400001	0,132099986	0,144499987	0,113299996

4-HT (540nm-690nm)/1 nM E2 anti-estrogenic control

cocentration [M	Replicate 1	Replicate 2	Replicate 3	Replicate 4	Mean
1,00E-06	20,60%	23,50%	24,61%	19,16%	**21,97%**

5th experiment

540nm

cocentration [M	Replicate 1	Replicate 2	Replicate 3	Replicate 4
1,00E-10	1,259899974	1,279800057	1,286000013	1,309499979
1,00E-09	1,277400017	1,298099995	1,281900048	1,261100054
1,00E-08	1,284500003	1,251099944	1,261399984	1,23969996
1,00E-07	1,225399971	1,236299992	1,257300019	1,311699986
1,00E-06	1,194499969	1,221600056	1,220499992	1,281200051
1,00E-05	1,225000024	1,19599998	1,193199992	1,32099998
1,00E-04	1,247799993	1,158200026	1,152099967	1,263299942

690nm

cocentration [M	Replicate 1	Replicate 2	Replicate 3	Replicate 4
1,00E-10	0,698199987	0,700900018	0,701399982	0,717899978
1,00E-09	0,720700026	0,755900025	0,718800008	0,708299994
1,00E-08	0,742999971	0,718599975	0,711199999	0,690999985
1,00E-07	0,692499995	0,70630002	0,713500023	0,759899974
1,00E-06	0,676900029	0,69749999	0,692399979	0,738200009
1,00E-05	0,6972	0,659200013	0,660300016	0,765699983
1,00E-04	0,683399975	0,613200009	0,596599996	0,693700016

solvent control 690nm

	Replicate 1	Replicate 2	Replicate 3	Replicate 4
	0,877699971	0,93809998	0,85680002	0,790000021

540-690nm

cocentration [M	Replicate 1	Replicate 2	Replicate 3	Replicate 4
1,00E-10	0,561699986	0,57889998	0,584500015	0,591600001
1,00E-09	0,556599975	0,542200029	0,563199997	0,5528
1,00E-08	0,541499972	0,532500029	0,550100029	0,548699975
1,00E-07	0,532800019	0,529999971	0,543799996	0,551699996
1,00E-06	0,517499983	0,524100006	0,528100014	0,542999983
1,00E-05	0,527800024	0,53670001	0,532899976	0,555299997
1,00E-04	0,564499974	0,545000017	0,555499971	0,569599986

solvent control 540nm-690nm

	Replicate 1	Replicate 2	Replicate 3	Replicate 4
	0,150800005	0,159299999	0,150299996	0,153200001

(540nm-690nm)-solvent control (540nm-690nm)

cocentration [M	Replicate 1	Replicate 2	Replicate 3	Replicate 4
1,00E-10	0,410899982	0,41959998	0,434200019	0,4384
1,00E-09	0,40579997	0,382900029	0,412900001	0,399599999
1,00E-08	0,390699968	0,373200029	0,399800032	0,395499974
1,00E-07	0,382000014	0,370699972	0,3935	0,398499995
1,00E-06	0,366699979	0,364800006	0,377800018	0,389799982
1,00E-05	0,377000019	0,377400011	0,38259998	0,402099997
1,00E-04	0,41369997	0,385700017	0,405199975	0,416399986

(540nm-690nm)-solvent control E2 reference

cocentration [M	Replicate 1	Replicate 2	Replicate 3	Replicate 4
1,00E-09	0,474000022	0,47420001	0,477100015	0,466400012

Normalization (substance/1 nM E2)

cocentration [M	Replicate 1	Replicate 2	Replicate 3	Replicate 4	Mean
1,00E-10	86,69%	88,49%	91,01%	94,00%	**90,04%**
1,00E-09	85,61%	80,75%	86,54%	85,68%	**84,64%**
1,00E-08	82,43%	78,70%	83,80%	84,80%	**82,43%**
1,00E-07	80,59%	78,17%	82,48%	85,44%	**81,67%**
1,00E-06	77,36%	76,93%	79,19%	83,58%	**79,26%**
1,00E-05	79,54%	79,59%	80,19%	86,21%	**81,38%**
1,00E-04	87,28%	81,34%	84,93%	89,28%	**85,71%**

yeast-growth/solubility 690nm/solvent control 690nm

cocentration [M	Replicate 1	Replicate 2	Replicate 3	Replicate 4	Mean
1,00E-10	79,55%	74,71%	81,86%	90,87%	**81,75%**
1,00E-09	82,11%	80,58%	83,89%	89,66%	**84,06%**
1,00E-08	84,65%	76,60%	83,01%	87,47%	**82,93%**
1,00E-07	78,90%	75,29%	83,27%	96,19%	**83,41%**
1,00E-06	77,12%	74,35%	80,81%	93,44%	**81,43%**
1,00E-05	79,43%	70,27%	77,07%	96,92%	**80,92%**
1,00E-04	77,86%	65,37%	69,63%	87,81%	**75,17%**

4-HT (540nm-690nm) anti-estrogenic control

cocentration [M	Replicate 1	Replicate 2	Replicate 3	Replicate 4
1,00E-06	0,106800005	0,100199997	0,132499993	0,069999993

4-HT (540nm-690nm)/1 nM E2 anti-estrogenic control

cocentration [M	Replicate 1	Replicate 2	Replicate 3	Replicate 4	Mean
1,00E-06	22,53%	21,13%	27,77%	15,01%	**21,61%**

6th experiment

540nm

cocentration [M	Replicate 1	Replicate 2	Replicate 3	Replicate 4
1,00E-10	1,29400003	1,325799942	1,317399979	1,339300036
1,00E-09	1,277899981	1,318600059	1,255200028	1,269099951
1,00E-08	1,25849998	1,326200008	1,319399953	1,328199983
1,00E-07	1,273900032	1,240700006	1,288699985	1,384899974
1,00E-06	1,33130002	1,312700033	1,315299988	1,295799971
1,00E-05	1,268300056	1,319399953	1,255699992	1,245900035
1,00E-04	1,326400042	1,299399972	1,269400001	1,212299943

690nm

cocentration [M	Replicate 1	Replicate 2	Replicate 3	Replicate 4
1,00E-10	0,632000029	0,641700029	0,646700025	0,671299994
1,00E-09	0,622900009	0,643299997	0,610000014	0,610000014
1,00E-08	0,602800012	0,639999986	0,637199998	0,671299994
1,00E-07	0,613799989	0,62529999	0,612900019	0,704999983
1,00E-06	0,65109998	0,635399997	0,638199985	0,618300021
1,00E-05	0,599399984	0,648000002	0,593200028	0,584399998
1,00E-04	0,627200007	0,619000018	0,601300001	0,556200027

solvent control 690nm

	Replicate 1	Replicate 2	Replicate 3	Replicate 4
	0,581499994	0,554099977	0,603999972	0,605599999

540-690nm

cocentration [M	Replicate 1	Replicate 2	Replicate 3	Replicate 4
1,00E-10	0,662100017	0,684099972	0,670799971	0,668099999
1,00E-09	0,654900014	0,675300002	0,645200014	0,659200013
1,00E-08	0,655799985	0,686200023	0,682200015	0,656899989
1,00E-07	0,660099983	0,615400016	0,675800025	0,679799974
1,00E-06	0,680100024	0,677399993	0,676999986	0,67750001
1,00E-05	0,668900013	0,671400011	0,662599981	0,661499977
1,00E-04	0,699199975	0,680400014	0,668200016	0,656099975

solvent control 540nm-690nm

	Replicate 1	Replicate 2	Replicate 3	Replicate 4
	0,131999999	0,127100006	0,134800002	0,134200007

(540nm-690nm)-solvent control (540nm-690nm)

cocentration [M	Replicate 1	Replicate 2	Replicate 3	Replicate 4
1,00E-10	0,530100018	0,556999967	0,535999969	0,533899993
1,00E-09	0,522900015	0,548199996	0,510400012	0,525000006
1,00E-08	0,523799986	0,559100017	0,547400013	0,522699982
1,00E-07	0,528099984	0,488300011	0,541000023	0,545599967
1,00E-06	0,548100024	0,550299987	0,542199984	0,543300003
1,00E-05	0,536900014	0,544300005	0,527799979	0,52729997
1,00E-04	0,567199975	0,553300008	0,533400014	0,521899968

(540nm-690nm)-solvent control E2 reference

cocentration [M	Replicate 1	Replicate 2	Replicate 3	Replicate 4
1,00E-09	0,674400017	0,68690002	0,68490003	0,693200007

Normalization (substance/1 nM E2)

cocentration [M	Replicate 1	Replicate 2	Replicate 3	Replicate 4	Mean
1,00E-10	78,60%	81,09%	78,26%	77,02%	**78,74%**
1,00E-09	77,54%	79,81%	74,52%	75,74%	**76,90%**
1,00E-08	77,67%	81,39%	79,92%	75,40%	**78,60%**
1,00E-07	78,31%	71,09%	78,99%	78,71%	**76,77%**
1,00E-06	81,27%	80,11%	79,16%	78,38%	**79,73%**
1,00E-05	79,61%	79,24%	77,06%	76,07%	**78,00%**
1,00E-04	84,10%	80,55%	77,88%	75,29%	**79,46%**

yeast-growth/solubility 690nm/solvent control 690nm

cocentration [M	Replicate 1	Replicate 2	Replicate 3	Replicate 4	Mean
1,00E-10	108,68%	115,81%	107,07%	110,85%	**110,60%**
1,00E-09	107,12%	116,10%	100,99%	100,73%	**106,23%**
1,00E-08	103,66%	115,50%	105,50%	110,85%	**108,88%**
1,00E-07	105,55%	112,85%	101,47%	116,41%	**109,07%**
1,00E-06	111,97%	114,67%	105,66%	102,10%	**108,60%**
1,00E-05	103,08%	116,95%	98,21%	96,50%	**103,68%**
1,00E-04	107,86%	111,71%	99,55%	91,84%	**102,74%**

4-HT (540nm-690nm) anti-estrogenic control

cocentration [M	Replicate 1	Replicate 2	Replicate 3	Replicate 4
1,00E-06	0,177899987	0,170000002	0,175299987	0,170899987

4-HT (540nm-690nm)/1 nM E2 anti-estrogenic control

cocentration [M	Replicate 1	Replicate 2	Replicate 3	Replicate 4	Mean
1,00E-06	26,38%	24,75%	25,59%	24,65%	**25,34%**

7th experiment

540nm

cocentration [M	Replicate 1	Replicate 2	Replicate 3	Replicate 4
1,00E-10	1,387400031	1,319200039	1,319499969	1,376000047
1,00E-09	1,298799992	1,301599979	1,274199963	1,303599954
1,00E-08	1,339300036	1,33920002	1,320700049	1,300500035
1,00E-07	1,398100019	1,361400008	1,329200029	1,340800047
1,00E-06	1,398699999	1,337499976	1,351300001	1,344099998
1,00E-05	1,358299971	1,35710001	1,387300014	1,391199946
1,00E-04	1,425099969	1,417400002	1,418599963	1,389799953

690nm

cocentration [M	Replicate 1	Replicate 2	Replicate 3	Replicate 4
1,00E-10	0,726800025	0,653800011	0,673300028	0,708599985
1,00E-09	0,653999984	0,638999999	0,624199986	0,645500004
1,00E-08	0,660399973	0,658999979	0,645099998	0,642700016
1,00E-07	0,700399995	0,668500006	0,662999988	0,654900014
1,00E-06	0,699100018	0,64289999	0,647599995	0,643899977
1,00E-05	0,6426	0,64349997	0,665300012	0,655300021
1,00E-04	0,655200005	0,641700029	0,656899989	0,630299985

solvent control 690nm

	Replicate 1	Replicate 2	Replicate 3	Replicate 4
	0,720099986	0,71509999	0,72240001	0,627499998

540-690nm

cocentration [M	Replicate 1	Replicate 2	Replicate 3	Replicate 4
1,00E-10	0,660600007	0,665400028	0,646200001	0,667400002
1,00E-09	0,644900024	0,662500024	0,649999976	0,658100009
1,00E-08	0,67900002	0,680199981	0,675599992	0,657800019
1,00E-07	0,697700024	0,692799985	0,666199982	0,685899973
1,00E-06	0,699599981	0,694599986	0,703700006	0,700200021
1,00E-05	0,715799987	0,71359998	0,722000003	0,735899985
1,00E-04	0,769900024	0,775699973	0,761699975	0,759500027

solvent control 540nm-690nm

	Replicate 1	Replicate 2	Replicate 3	Replicate 4
	0,154799998	0,154699996	0,157700002	0,149100006

(540nm-690nm)-solvent control (540nm-690nm)

cocentration [M	Replicate 1	Replicate 2	Replicate 3	Replicate 4
1,00E-10	0,505800009	0,510700032	0,488499999	0,518299997
1,00E-09	0,490100026	0,507800028	0,492299974	0,509000003
1,00E-08	0,524200022	0,525499985	0,51789999	0,508700013
1,00E-07	0,542900026	0,538099989	0,50849998	0,536799967
1,00E-06	0,544799984	0,53989999	0,546000004	0,551100016
1,00E-05	0,56099999	0,558899984	0,564300001	0,586799979
1,00E-04	0,615100026	0,620999977	0,603999972	0,610400021

(540nm-690nm)-solvent control E2 reference

cocentration [M	Replicate 1	Replicate 2	Replicate 3	Replicate 4
1,00E-09	0,626299992	0,650099993	0,643300012	0,647899985

Normalization (substance/1 nM E2)

cocentration [M	Replicate 1	Replicate 2	Replicate 3	Replicate 4	Mean
1,00E-10	80,76%	78,56%	75,94%	80,00%	**78,81%**
1,00E-09	78,25%	78,11%	76,53%	78,56%	**77,86%**
1,00E-08	83,70%	80,83%	80,51%	78,52%	**80,89%**
1,00E-07	86,68%	82,77%	79,05%	82,85%	**82,84%**
1,00E-06	86,99%	83,05%	84,87%	85,06%	**84,99%**
1,00E-05	89,57%	85,97%	87,72%	90,57%	**88,46%**
1,00E-04	98,21%	95,52%	93,89%	94,21%	**95,46%**

yeast-growth/solubility 690nm/solvent control 690nm

cocentration [M	Replicate 1	Replicate 2	Replicate 3	Replicate 4	Mean
1,00E-10	100,93%	91,43%	93,20%	112,92%	**99,62%**
1,00E-09	90,82%	89,36%	86,41%	102,87%	**92,36%**
1,00E-08	91,71%	92,15%	89,30%	102,42%	**93,90%**
1,00E-07	97,26%	93,48%	91,78%	104,37%	**96,72%**
1,00E-06	97,08%	89,90%	89,65%	102,61%	**94,81%**
1,00E-05	89,24%	89,99%	92,10%	104,43%	**93,94%**
1,00E-04	90,99%	89,74%	90,93%	100,45%	**93,03%**

4-HT (540nm-690nm) anti-estrogenic control

cocentration [M	Replicate 1	Replicate 2	Replicate 3	Replicate 4
1,00E-06	0,159299999	0,163900003	0,156800002	0,1708

4-HT (540nm-690nm)/1 nM E2 anti-estrogenic control

cocentration [M	Replicate 1	Replicate 2	Replicate 3	Replicate 4	Mean
1,00E-06	25,44%	25,21%	24,37%	26,36%	**25,35%**

8th experiment

540nm

cocentration [M	Replicate 1	Replicate 2	Replicate 3	Replicate 4
1,00E-10	1,115100026	1,073199987	1,078699946	1,121199965
1,00E-09	1,074699998	1,098500013	1,108899951	1,10650003
1,00E-08	1,069499969	1,078500032	1,092599988	1,045699954
1,00E-07	1,08070004	1,068099976	1,058200002	1,085500002
1,00E-06	1,066900015	1,077800035	1,107900023	1,097599983
1,00E-05	1,159600019	1,120300055	1,100499988	1,090199947
1,00E-04	1,162299991	1,110599995	1,118999958	1,046900034

690nm

cocentration [M	Replicate 1	Replicate 2	Replicate 3	Replicate 4
1,00E-10	0,607500017	0,620599985	0,617699981	0,643100023
1,00E-09	0,625899971	0,652700007	0,60650003	0,64289999
1,00E-08	0,618200004	0,620800018	0,638100028	0,604600012
1,00E-07	0,626999974	0,613699973	0,611500025	0,533200026
1,00E-06	0,602500021	0,605300009	0,615400016	0,63410002
1,00E-05	0,609499991	0,634599984	0,619400024	0,613600016
1,00E-04	0,620299995	0,601999998	0,619400024	0,608799994

solvent control 690nm

	Replicate 1	Replicate 2	Replicate 3	Replicate 4
	0,734799981	0,705600023	0,703599989	0,720399976

540-690nm

cocentration [M	Replicate 1	Replicate 2	Replicate 3	Replicate 4
1,00E-10	0,507600009	0,452600002	0,460999995	0,478100002
1,00E-09	0,448799998	0,445800006	0,502399981	0,46360001
1,00E-08	0,451200008	0,457800001	0,454600006	0,441100001
1,00E-07	0,453700006	0,454400003	0,446700007	0,552399993
1,00E-06	0,464399993	0,472499996	0,492599994	0,46360001
1,00E-05	0,550100029	0,485700011	0,481099993	0,476700008
1,00E-04	0,541899979	0,508599997	0,499599993	0,43810001

solvent control 540nm-690nm

	Replicate 1	Replicate 2	Replicate 3	Replicate 4
	0,147200003	0,145600006	0,145600006	0,148200005

(540nm-690nm)-solvent control (540nm-690nm)

cocentration [M	Replicate 1	Replicate 2	Replicate 3	Replicate 4
1,00E-10	0,360400006	0,306999996	0,315399989	0,329899997
1,00E-09	0,301599994	0,3002	0,356799975	0,315400004
1,00E-08	0,304000005	0,312199995	0,309	0,292899996
1,00E-07	0,306500003	0,308799997	0,301100001	0,404199988
1,00E-06	0,31719999	0,32689999	0,346999988	0,315400004
1,00E-05	0,402900025	0,340100005	0,335499987	0,328500003
1,00E-04	0,394699976	0,362999991	0,353999987	0,289900005

(540nm-690nm)-solvent control E2 reference

cocentration [M	Replicate 1	Replicate 2	Replicate 3	Replicate 4
1,00E-09	0,339200005	0,327099994	0,320900008	0,316100001

Normalization (substance/1 nM E2)

cocentration [M	Replicate 1	Replicate 2	Replicate 3	Replicate 4	Mean
1,00E-10	106,25%	93,86%	98,29%	104,37%	**100,69%**
1,00E-09	88,92%	91,78%	111,19%	99,78%	**97,91%**
1,00E-08	89,62%	95,44%	96,29%	92,66%	**93,50%**
1,00E-07	90,36%	94,41%	93,83%	127,87%	**101,62%**
1,00E-06	93,51%	99,94%	108,13%	99,78%	**100,34%**
1,00E-05	118,78%	103,97%	104,55%	103,92%	**107,81%**
1,00E-04	116,36%	110,98%	110,31%	91,71%	**107,34%**

yeast-growth/solubility 690nm/solvent control 690nm

cocentration [M	Replicate 1	Replicate 2	Replicate 3	Replicate 4	Mean
1,00E-10	82,68%	87,95%	87,79%	89,27%	**86,92%**
1,00E-09	85,18%	92,50%	86,20%	89,24%	**88,28%**
1,00E-08	84,13%	87,98%	90,69%	83,93%	**86,68%**
1,00E-07	85,33%	86,98%	86,91%	74,01%	**83,31%**
1,00E-06	82,00%	85,79%	87,46%	88,02%	**85,82%**
1,00E-05	82,95%	89,94%	88,03%	85,17%	**86,52%**
1,00E-04	84,42%	85,32%	88,03%	84,51%	**85,57%**

4-HT (540nm-690nm) anti-estrogenic control

cocentration [M	Replicate 1	Replicate 2	Replicate 3	Replicate 4
1,00E-06	0,093899995	0,099299997	0,085099995	0,110199988

4-HT (540nm-690nm)/1 nM E2 anti-estrogenic control

cocentration [M	Replicate 1	Replicate 2	Replicate 3	Replicate 4	Mean
1,00E-06	27,68%	30,36%	26,52%	34,86%	**29,86%**

YES chlorpyrifos + E2

1st experiment

540nm

cocentration [M]	Replicate 1	Replicate 2	Replicate 3	Replicate 4
1,00E-07	0,901000023	0,831700027	0,903299987	0,890799999
1,00E-06	0,892099977	0,867299974	1,006899953	0,905200005
1,00E-05	1,450600028	1,06219995	1,098099947	1,034700036
5,00E-05	0,977400005	1,005900025	0,985599995	0,98360002
1,00E-04	1,040599942	1,065199971	1,0546	1,050300002
5,00E-04	1,261999965	1,112499952	1,122599959	1,100999951
1,00E-03	1,477699995	1,486099958	1,390300035	1,546599984

690nm

cocentration [M]	Replicate 1	Replicate 2	Replicate 3	Replicate 4
1,00E-07	0,423200011	0,351000011	0,418099999	0,410699993
1,00E-06	0,40290001	0,393400013	0,494599998	0,430799991
1,00E-05	0,696200013	0,554400027	0,596700013	0,5528
5,00E-05	0,532299995	0,546500027	0,547200024	0,535899997
1,00E-04	0,566600025	0,546000004	0,559899986	0,566999972
5,00E-04	0,63499999	0,56129998	0,555299997	0,583199978
1,00E-03	0,696799994	0,685800016	0,661199987	0,743799984

solvent control 690nm

	Replicate 1	Replicate 2	Replicate 3	Replicate 4
	0,479099989	0,528800011	0,54549998	0,538999975

540-690nm

cocentration [M]	Replicate 1	Replicate 2	Replicate 3	Replicate 4
1,00E-07	0,477800012	0,480800003	0,485300004	0,479999989
1,00E-06	0,489199996	0,47389999	0,512300014	0,474400014
1,00E-05	0,754299998	0,507799983	0,501399994	0,481900007
5,00E-05	0,445100009	0,459300011	0,4384	0,44780001
1,00E-04	0,474000007	0,519200027	0,494700015	0,4833
5,00E-04	0,626999974	0,551199973	0,567300022	0,517799973
1,00E-03	0,781000018	0,800300002	0,729099989	0,8028

solvent control 540nm-690nm

	Replicate 1	Replicate 2	Replicate 3	Replicate 4
	0,119900003	0,123000003	0,122299999	0,123000003

(540nm-690nm)-solvent control (540nm-690nm)

cocentration [M]	Replicate 1	Replicate 2	Replicate 3	Replicate 4
1,00E-07	0,357900009	0,357799999	0,363000005	0,356999986
1,00E-06	0,369299993	0,350899987	0,390000015	0,35140001
1,00E-05	0,634399995	0,38479998	0,379099995	0,358900003
5,00E-05	0,325200006	0,336300008	0,316100001	0,324800007
1,00E-04	0,354100004	0,396200024	0,372400016	0,360299997
5,00E-04	0,507099971	0,428199969	0,445000023	0,39479997
1,00E-03	0,661100015	0,677299999	0,60679999	0,679799996

(540nm-690nm)-solvent control E2 reference

cocentration [M]	Replicate 1	Replicate 2	Replicate 3	Replicate 4
1,00E-09	0,432500005	0,401799969	0,412900023	0,42240002

Normalization (substance/1 nM E2)

cocentration [M]	Replicate 1	Replicate 2	Replicate 3	Replicate 4	Mean
1,00E-07	82,75%	89,05%	87,91%	84,52%	**86,06%**
1,00E-06	85,39%	87,33%	94,45%	83,19%	**87,59%**
1,00E-05	146,68%	95,77%	91,81%	84,97%	**104,81%**
5,00E-05	75,19%	83,70%	76,56%	76,89%	**78,08%**
1,00E-04	81,87%	98,61%	90,19%	85,30%	**88,99%**
5,00E-04	117,25%	106,57%	107,77%	93,47%	**106,26%**
1,00E-03	152,86%	168,57%	146,96%	160,94%	**157,33%**

yeast-growth/solubility 690nm/solvent control 690nm

cocentration [M]	Replicate 1	Replicate 2	Replicate 3	Replicate 4	Mean
1,00E-07	88,33%	66,38%	76,65%	76,20%	**76,89%**
1,00E-06	84,10%	74,39%	90,67%	79,93%	**82,27%**
1,00E-05	145,31%	104,84%	109,39%	102,56%	**115,53%**
5,00E-05	111,10%	103,35%	100,31%	99,42%	**103,55%**
1,00E-04	132,54%	106,15%	101,80%	108,20%	**112,17%**
5,00E-04	118,26%	103,25%	102,64%	105,19%	**107,34%**
1,00E-03	145,44%	129,69%	121,21%	138,00%	**133,58%**

4-HT (540nm-690nm) anti-estrogenic control

cocentration [M]	Replicate 1	Replicate 2	Replicate 3	Replicate 4
1,00E-06	0,0546	0,055599995	0,051100001	0,049900003

4-HT (540nm-690nm)/1 nM E2 anti-estrogenic control

cocentration [M]	Replicate 1	Replicate 2	Replicate 3	Replicate 4	Mean
1,00E-06	12,62%	13,84%	12,38%	11,81%	**12,66%**

2nd experient

540nm

cocentration [M]	Replicate 1	Replicate 2	Replicate 3	Replicate 4
1,00E-07	0,867399991	0,87620002	0,892400026	0,838999987
1,00E-06	0,842800021	0,881799996	0,8829	0,841499984
1,00E-05	0,979200006	0,95539999	0,941900015	0,937300026
5,00E-05	0,998099983	0,979600012	0,992500007	0,992500007
1,00E-04	1,024199963	0,979399979	0,992299974	1,041200042
5,00E-04	1,104200006	1,171900034	1,14349997	1,183400035
1,00E-03	1,728999972	1,656200051	1,632799983	1,700199962

690nm

cocentration [M]	Replicate 1	Replicate 2	Replicate 3	Replicate 4
1,00E-07	0,423799992	0,438499987	0,451799989	0,403200001
1,00E-06	0,405000001	0,438699991	0,436399996	0,401199996
1,00E-05	0,532800019	0,518299997	0,5079	0,51789999
5,00E-05	0,547699988	0,541299999	0,562900007	0,546000004
1,00E-04	0,567099988	0,538500011	0,557500005	0,57889998
5,00E-04	0,610899985	0,644699991	0,621999979	0,625199974
1,00E-03	0,783900023	0,795400023	0,759500027	0,752200007

solvent control 690nm

	Replicate 1	Replicate 2	Replicate 3	Replicate 4
	0,479099989	0,5097	0,504599988	0,44600001

540-690nm

cocentration [M]	Replicate 1	Replicate 2	Replicate 3	Replicate 4
1,00E-07	0,443599999	0,437700003	0,440600008	0,435799986
1,00E-06	0,437900007	0,443100005	0,446500003	0,440299988
1,00E-05	0,446399987	0,437099993	0,433999985	0,419400007
5,00E-05	0,450399995	0,438300014	0,4296	0,44659999
1,00E-04	0,457100004	0,440800011	0,434799999	0,462300003
5,00E-04	0,493299991	0,527199984	0,521399975	0,558200002
1,00E-03	0,945100009	0,860800028	0,873300016	0,948000014

solvent control 540nm-690nm

	Replicate 1	Replicate 2	Replicate 3	Replicate 4
	0,121399999	0,121299997	0,124300003	0,1149

(540nm-690nm)-solvent control (540nm-690nm)

cocentration [M]	Replicate 1	Replicate 2	Replicate 3	Replicate 4
1,00E-07	0,3222	0,316400006	0,316300005	0,320899986
1,00E-06	0,316500008	0,321800008	0,3222	0,325399987
1,00E-05	0,324999988	0,315799996	0,309699982	0,304500006
5,00E-05	0,328999996	0,317000017	0,305299997	0,33169999
1,00E-04	0,335700005	0,319500014	0,310499996	0,347400002
5,00E-04	0,371899992	0,405899987	0,397099972	0,443300001
1,00E-03	0,823700011	0,739500031	0,749000013	0,833100013

(540nm-690nm)-solvent control E2 reference

cocentration [M]	Replicate 1	Replicate 2	Replicate 3	Replicate 4
1,00E-09	0,323400006	0,306000009	0,330699988	0,313099988

Normalization (substance/1 nM E2)

cocentration [M]	Replicate 1	Replicate 2	Replicate 3	Replicate 4	Mean
1,00E-07	99,63%	103,40%	95,65%	102,49%	**100,29%**
1,00E-06	97,87%	105,16%	97,43%	103,93%	**101,10%**
1,00E-05	100,49%	103,20%	93,65%	97,25%	**98,65%**
5,00E-05	101,73%	103,59%	92,32%	105,94%	**100,90%**
1,00E-04	103,80%	104,41%	93,89%	110,95%	**103,27%**
5,00E-04	115,00%	132,65%	120,08%	141,58%	**127,33%**
1,00E-03	254,70%	241,67%	226,49%	266,08%	**247,23%**

yeast-growth/solubility 690nm/solvent control 690nm

cocentration [M]	Replicate 1	Replicate 2	Replicate 3	Replicate 4	Mean
1,00E-07	88,46%	86,03%	89,54%	90,40%	**88,61%**
1,00E-06	84,53%	86,07%	86,48%	89,96%	**86,76%**
1,00E-05	111,21%	101,69%	100,65%	116,12%	**107,42%**
5,00E-05	114,32%	106,20%	111,55%	122,42%	**113,62%**
1,00E-04	118,37%	105,65%	110,48%	129,80%	**116,07%**
5,00E-04	127,51%	126,49%	123,27%	140,18%	**129,36%**
1,00E-03	163,62%	156,05%	150,52%	168,65%	**159,71%**

4-HT (540nm-690nm) anti-estrogenic control

cocentration [M]	Replicate 1	Replicate 2	Replicate 3	Replicate 4
1,00E-06	0,055199996	0,050500005	0,051100001	0,0691

4-HT (540nm-690nm)/1 nM E2 anti-estrogenic control

cocentration [M]	Replicate 1	Replicate 2	Replicate 3	Replicate 4	Mean
1,00E-06	17,07%	16,50%	15,45%	22,07%	**17,77%**

3rd experiment

540nm

cocentration [M	Replicate 1	Replicate 2	Replicate 3	Replicate 4
1,00E-07	1,015499949	1,06099999	0,976800025	0,989600003
1,00E-06	0,983399987	1,266499996	0,994199991	0,990199983
1,00E-05	1,140799999	1,138000011	1,047500014	1,262199998
5,00E-05	1,113399982	1,151000023	1,150099993	1,181200027
1,00E-04	1,165699959	1,225800037	1,202999949	1,19630003
5,00E-04	1,382500052	1,311300039	1,289899945	1,293599963
1,00E-03	1,594799995	1,480800033	1,526000023	1,694900036

690nm

cocentration [M	Replicate 1	Replicate 2	Replicate 3	Replicate 4
1,00E-07	0,501600027	0,540700018	0,473800004	0,480300009
1,00E-06	0,478799999	0,628000021	0,485300004	0,489300013
1,00E-05	0,62559998	0,581700027	0,544399977	0,655700028
5,00E-05	0,602800012	0,629599988	0,624000013	0,639999986
1,00E-04	0,626399994	0,660300016	0,633899987	0,636799991
5,00E-04	0,7227	0,691299975	0,679199994	0,668200016
1,00E-03	0,703299999	0,681599975	0,661800027	0,723999977

solvent control 690nm

	Replicate 1	Replicate 2	Replicate 3	Replicate 4
	0,561600029	0,570800006	0,621599972	0,614199996

540-690nm

cocentration [M	Replicate 1	Replicate 2	Replicate 3	Replicate 4
1,00E-07	0,513800025	0,520299971	0,503000021	0,509299994
1,00E-06	0,504499972	0,638499975	0,508899987	0,500800014
1,00E-05	0,515200019	0,556299984	0,503099978	0,60650003
5,00E-05	0,510599971	0,521399975	0,526199996	0,541199982
1,00E-04	0,539300025	0,565500021	0,569100022	0,559599996
5,00E-04	0,659799993	0,620000005	0,610700011	0,625400007
1,00E-03	0,891499996	0,799199998	0,864199996	0,970899999

solvent control 540nm-690nm

	Replicate 1	Replicate 2	Replicate 3	Replicate 4
	0,1052	0,108800001	0,1109	0,111500002

(540nm-690nm)-solvent control (540nm-690nm)

cocentration [M	Replicate 1	Replicate 2	Replicate 3	Replicate 4
1,00E-07	0,408600025	0,41149997	0,392100021	0,397799991
1,00E-06	0,399299972	0,529699974	0,397999987	0,389300011
1,00E-05	0,410000019	0,447499983	0,392199978	0,495000027
5,00E-05	0,405399971	0,412599973	0,415299997	0,42969998
1,00E-04	0,434100025	0,45670002	0,458200023	0,448099993
5,00E-04	0,554599993	0,511200003	0,499800012	0,513900004
1,00E-03	0,786299996	0,690399997	0,753299996	0,859399997

(540nm-690nm)-solvent control E2 reference

cocentration [M	Replicate 1	Replicate 2	Replicate 3	Replicate 4
1,00E-09	0,587700002	0,401399985	0,414099991	0,434400015

Normalization (substance/1 nM E2)

cocentration [M	Replicate 1	Replicate 2	Replicate 3	Replicate 4	Mean
1,00E-07	69,53%	102,52%	94,69%	91,57%	**89,58%**
1,00E-06	67,94%	131,96%	96,11%	89,62%	**96,41%**
1,00E-05	69,76%	111,48%	94,71%	113,95%	**97,48%**
5,00E-05	68,98%	102,79%	100,29%	98,92%	**92,74%**
1,00E-04	73,86%	113,78%	110,65%	103,15%	**100,36%**
5,00E-04	94,37%	127,35%	120,70%	118,30%	**115,18%**
1,00E-03	133,79%	172,00%	181,91%	197,84%	**171,38%**

yeast-growth/solubility 690nm/solvent control 690nm

cocentration [M	Replicate 1	Replicate 2	Replicate 3	Replicate 4	Mean
1,00E-07	89,32%	94,73%	76,22%	78,20%	**84,62%**
1,00E-06	85,26%	110,02%	78,07%	79,66%	**88,25%**
1,00E-05	111,40%	101,91%	87,58%	106,76%	**101,91%**
5,00E-05	107,34%	110,30%	100,39%	104,20%	**105,56%**
1,00E-04	111,54%	115,68%	101,98%	103,68%	**108,22%**
5,00E-04	128,69%	121,11%	109,27%	108,79%	**116,96%**
1,00E-03	125,23%	119,41%	106,47%	117,88%	**117,25%**

4-HT (540nm-690nm) anti-estrogenic control

cocentration [M	Replicate 1	Replicate 2	Replicate 3	Replicate 4
1,00E-06	0,058600001	0,060999997	0,059200004	0,067699991

4-HT (540nm-690nm)/1 nM E2 anti-estrogenic control

cocentration [M	Replicate 1	Replicate 2	Replicate 3	Replicate 4	Mean
1,00E-06	9,97%	15,20%	14,30%	15,58%	**13,76%**

4th experiment

540nm

cocentration [M	Replicate 1	Replicate 2	Replicate 3	Replicate 4
1,00E-07	1,1796	1,237699986	1,244199991	1,22510004
1,00E-06	1,39199996	1,276600003	1,215299964	1,282199979
1,00E-05	1,336300015	1,400200009	1,381500006	1,379899979
5,00E-05	1,369600058	1,487699986	1,326900005	1,348999977
1,00E-04	1,386600018	1,451799989	1,415199995	1,415799975
5,00E-04	1,544499993	1,702900052	1,452999949	1,443600059
1,00E-03	1,574399948	1,581900001	1,603000045	1,663100004

690nm

cocentration [M	Replicate 1	Replicate 2	Replicate 3	Replicate 4
1,00E-07	0,585699975	0,631500006	0,639999986	0,626299977
1,00E-06	0,649999976	0,606400013	0,587199986	0,600300014
1,00E-05	0,707300007	0,70539999	0,700999975	0,698700011
5,00E-05	0,739000022	0,772000015	0,692199975	0,710500002
1,00E-04	0,736599982	0,763199985	0,745400012	0,725399971
5,00E-04	0,771000028	0,715699971	0,75819999	0,764199972
1,00E-03	0,719500005	0,738200009	0,716400027	0,763899982

solvent control 690nm

	Replicate 1	Replicate 2	Replicate 3	Replicate 4
	0,704599977	0,635800004	0,7051	0,584999979

540-690nm

cocentration [M	Replicate 1	Replicate 2	Replicate 3	Replicate 4
1,00E-07	0,593900025	0,60619998	0,604200006	0,598800004
1,00E-06	0,741999984	0,67019999	0,628099978	0,681800008
1,00E-05	0,629000008	0,694800019	0,680499971	0,681200027
5,00E-05	0,630599976	0,715699971	0,635599971	0,638499975
1,00E-04	0,649999976	0,688600004	0,669799984	0,690299988
5,00E-04	0,773500025	0,987200022	0,694800019	0,679400027
1,00E-03	0,854900002	0,843699992	0,886600018	0,899200022

solvent control 540nm-690nm

	Replicate 1	Replicate 2	Replicate 3	Replicate 4
	0,104199998	0,100599997	0,103799999	0,092500001

(540nm-690nm)-solvent control (540nm-690nm)

cocentration [M	Replicate 1	Replicate 2	Replicate 3	Replicate 4
1,00E-07	0,489700027	0,505599983	0,500400007	0,506300002
1,00E-06	0,637799986	0,569599994	0,524299979	0,589300007
1,00E-05	0,52480001	0,594200023	0,576699972	0,588700026
5,00E-05	0,526399978	0,615099974	0,531799972	0,545999974
1,00E-04	0,545799978	0,588000007	0,565999985	0,597799987
5,00E-04	0,669300027	0,886600025	0,591000021	0,586900026
1,00E-03	0,750700004	0,743099995	0,782800019	0,806700021

(540nm-690nm)-solvent control E2 reference

cocentration [M	Replicate 1	Replicate 2	Replicate 3	Replicate 4
1,00E-09	0,51979997	0,531299993	0,54239998	0,55270002

Normalization (substance/1 nM E2)

cocentration [M	Replicate 1	Replicate 2	Replicate 3	Replicate 4	Mean
1,00E-07	94,21%	95,16%	92,26%	91,60%	**93,31%**
1,00E-06	122,70%	107,21%	96,66%	106,62%	**108,30%**
1,00E-05	100,96%	111,84%	106,32%	106,51%	**106,41%**
5,00E-05	101,27%	115,77%	98,05%	98,79%	**103,47%**
1,00E-04	105,00%	110,67%	104,35%	108,16%	**107,05%**
5,00E-04	128,76%	166,87%	108,96%	106,19%	**127,70%**
1,00E-03	144,42%	139,86%	144,32%	145,96%	**143,64%**

yeast-growth/solubility 690nm/solvent control 690nm

cocentration [M	Replicate 1	Replicate 2	Replicate 3	Replicate 4	Mean
1,00E-07	83,13%	99,32%	90,77%	107,06%	**95,07%**
1,00E-06	92,25%	95,38%	83,28%	102,62%	**93,38%**
1,00E-05	100,38%	110,95%	99,42%	119,44%	**107,55%**
5,00E-05	104,88%	121,42%	98,04%	121,45%	**111,45%**
1,00E-04	104,54%	120,04%	105,72%	124,00%	**113,57%**
5,00E-04	109,42%	112,57%	107,53%	130,63%	**115,04%**
1,00E-03	102,11%	116,11%	101,60%	130,58%	**112,60%**

4-HT (540nm-690nm) anti-estrogenic control

cocentration [M	Replicate 1	Replicate 2	Replicate 3	Replicate 4
1,00E-06	0,067600004	0,078400008	0,072500005	0,085099995

4-HT (540nm-690nm)/1 nM E2 anti-estrogenic control

cocentration [M	Replicate 1	Replicate 2	Replicate 3	Replicate 4	Mean
1,00E-06	13,01%	14,76%	13,37%	15,40%	**14,13%**

5th experiment

540nm

cocentration [M	Replicate 1	Replicate 2	Replicate 3	Replicate 4
1,00E-07	1,07980001	1,119500041	1,113000035	1,253000021
1,00E-06	1,162400007	1,100600004	1,071799994	1,12650001
1,00E-05	1,192600012	1,152699947	1,110800028	1,093000054
5,00E-05	1,168900013	1,143000007	2,248899937	1,118399978
1,00E-04	1,1796	1,202900052	1,197100043	1,158599973
5,00E-04	1,399399996	1,406299949	1,409500003	1,439900041
1,00E-03	1,846699953	1,905300021	1,857499957	1,834800005

690nm

cocentration [M	Replicate 1	Replicate 2	Replicate 3	Replicate 4
1,00E-07	0,509199977	0,516700029	0,514400005	0,59009999
1,00E-06	0,556299984	0,515900016	0,495900005	0,52759999
1,00E-05	0,563799977	0,545000017	0,510299981	0,498100013
5,00E-05	0,582099974	0,551299989	0,674300015	0,541800022
1,00E-04	0,564400017	0,582899988	0,578199983	0,54610002
5,00E-04	0,619499981	0,62440002	0,608900011	0,659399986
1,00E-03	0,677399993	0,680199981	0,672599971	0,659799993

solvent control 690nm

	Replicate 1	Replicate 2	Replicate 3	Replicate 4
	0,639800012	0,614000022	0,620800018	0,603999972

540-690nm

cocentration [M	Replicate 1	Replicate 2	Replicate 3	Replicate 4
1,00E-07	0,570599973	0,602699995	0,59859997	0,662899971
1,00E-06	0,606100023	0,584800005	0,575900018	0,59890002
1,00E-05	0,628899992	0,60769999	0,600600004	0,594799995
5,00E-05	0,586799979	0,591700017	1,574699998	0,576600015
1,00E-04	0,615199983	0,619899988	0,618900001	0,612500012
5,00E-04	0,779900014	0,781799972	0,800599992	0,780499995
1,00E-03	1,16929996	1,225000024	1,184900045	1,174999952

solvent control 540nm-690nm

	Replicate 1	Replicate 2	Replicate 3	Replicate 4
	0,108900003	0,106899999	0,1074	0,106600001

(540nm-690nm)-solvent control (540nm-690nm)

cocentration [M	Replicate 1	Replicate 2	Replicate 3	Replicate 4
1,00E-07	0,46169997	0,495799996	0,49119997	0,55629997
1,00E-06	0,49720002	0,477900006	0,468500018	0,492300019
1,00E-05	0,519999988	0,500799991	0,493200004	0,488199994
5,00E-05	0,477899976	0,484800018	1,467299998	0,470000014
1,00E-04	0,50629998	0,512999989	0,511500001	0,50590001
5,00E-04	0,671000011	0,674899973	0,693199992	0,673899993
1,00E-03	1,060399957	1,118100025	1,077500045	1,068399951

(540nm-690nm)-solvent control E2 reference

cocentration [M	Replicate 1	Replicate 2	Replicate 3	Replicate 4
1,00E-09	0,512500018	0,546300001	0,527200021	0,555800028

Normalization (substance/1 nM E2)

cocentration [M	Replicate 1	Replicate 2	Replicate 3	Replicate 4	Mean
1,00E-07	90,09%	90,76%	93,17%	100,09%	**93,53%**
1,00E-06	97,01%	87,48%	88,87%	88,58%	**90,48%**
1,00E-05	101,46%	91,67%	93,55%	87,84%	**93,63%**
5,00E-05	93,25%	88,74%	278,32%	84,56%	**136,22%**
1,00E-04	98,79%	93,90%	97,02%	91,02%	**95,18%**
5,00E-04	130,93%	123,54%	131,49%	121,25%	**126,80%**
1,00E-03	206,91%	204,67%	204,38%	192,23%	**202,05%**

yeast-growth/solubility 690nm/solvent control 690nm

cocentration [M	Replicate 1	Replicate 2	Replicate 3	Replicate 4	Mean
1,00E-07	79,59%	84,15%	82,86%	97,70%	**86,07%**
1,00E-06	86,95%	84,02%	79,88%	87,35%	**84,55%**
1,00E-05	88,12%	88,76%	82,20%	82,47%	**85,39%**
5,00E-05	90,98%	89,79%	108,62%	89,70%	**94,77%**
1,00E-04	88,22%	94,93%	93,14%	90,41%	**91,68%**
5,00E-04	96,83%	101,69%	98,08%	109,17%	**101,44%**
1,00E-03	105,88%	110,78%	108,34%	109,24%	**108,56%**

4-HT (540nm-690nm) anti-estrogenic control

cocentration [M	Replicate 1	Replicate 2	Replicate 3	Replicate 4
1,00E-06	0,057700001	0,0568	0,060200006	0,0625

4-HT (540nm-690nm)/1 nM E2 anti-estrogenic control

cocentration [M	Replicate 1	Replicate 2	Replicate 3	Replicate 4	Mean
1,00E-06	11,26%	10,40%	11,42%	11,25%	**11,08%**

cell-free 690nm

cocentration [M	Replicate 1	Replicate 2	Replicate 3	Replicate 4
1,00E-07	0,037900001	0,038600001	0,0372	0,0372
1,00E-06	0,037599999	0,0381	0,036600001	0,0372
1,00E-05	0,0372	0,037300002	0,0363	0,037300002
5,00E-05	0,043200001	0,042100001	0,0425	0,042100001
1,00E-04	0,052000001	0,051899999	0,050299998	0,051899999
5,00E-04	0,1558	0,144899994	0,1391	0,1426
1,00E-03	0,236499995	0,237399995	0,248600006	0,273000002

cell-free solvent control 690nm

cocentration [M	Replicate 1	Replicate 2	Replicate 3	Replicate 4
	0,037900001	0,036600001	0,036699999	0,036699999

cell-free (substance/solvent control)

cocentration [M	Replicate 1	Replicate 2	Replicate 3	Replicate 4	Mean
1,00E-07	100,00%	105,46%	101,36%	101,36%	**102,05%**
1,00E-06	99,21%	104,10%	99,73%	101,36%	**101,10%**
1,00E-05	98,15%	101,91%	98,91%	101,63%	**100,15%**
5,00E-05	113,98%	115,03%	115,80%	114,71%	**114,88%**
1,00E-04	137,20%	141,80%	137,06%	141,42%	**139,37%**
5,00E-04	411,08%	395,90%	379,02%	388,56%	**393,64%**
1,00E-03	624,01%	648,63%	677,38%	743,87%	**673,47%**

YES fenarimol + E2

1st experiment

540nm

cocentration [M]	Replicate 1	Replicate 2	Replicate 3	Replicate 4
1,00E-06	0,90109998	0,879499972	0,842599988	0,911499977
5,00E-06	0,91900003	0,866100013	0,875400007	0,867699981
1,00E-05	0,908200026	0,797800004	0,83069998	0,851400018
5,00E-05	1,887400031	1,694399953	1,645599961	1,761399984
1,00E-04	2,367899895	2,246599913	2,225500107	2,228100061
5,00E-04	1,293099999	1,197100043	1,267400026	1,306300044
1,00E-03	1,430799961	1,280200005	1,190999985	1,301499963

690nm

cocentration [M]	Replicate 1	Replicate 2	Replicate 3	Replicate 4
1,00E-06	0,420700014	0,417199999	0,398900002	0,446099997
5,00E-06	0,453200012	0,42930001	0,439099997	0,436300009
1,00E-05	0,468199998	0,390799999	0,422699988	0,423000008
5,00E-05	0,461499989	0,429699987	0,398299992	0,459699988
1,00E-04	0,398600012	0,413500011	0,375	0,393700004
5,00E-04	0,395200014	0,368900001	0,39320001	0,407799989
1,00E-03	0,553799987	0,483700007	0,403499991	0,512199998

solvent control 690nm

	Replicate 1	Replicate 2	Replicate 3	Replicate 4
	0,470800012	0,460000008	0,497599989	0,468499988

540-690nm

cocentration [M]	Replicate 1	Replicate 2	Replicate 3	Replicate 4
1,00E-06	0,480399996	0,462300003	0,443800002	0,46540001
5,00E-06	0,465700001	0,436800003	0,436399996	0,431400001
1,00E-05	0,439999998	0,407000005	0,408100009	0,42840001
5,00E-05	1,425899982	1,264799953	1,247300029	1,301800013
1,00E-04	1,969300032	1,833099961	1,850499988	1,834400058
5,00E-04	0,898000002	0,828100026	0,874199986	0,898500025
1,00E-03	0,876999974	0,796500027	0,787500024	0,789300025

solvent control 540nm-690nm

	Replicate 1	Replicate 2	Replicate 3	Replicate 4
	0,119800001	0,120999999	0,120700002	0,115099996

(540nm-690nm)-solvent control (540nm-690nm)

cocentration [M]	Replicate 1	Replicate 2	Replicate 3	Replicate 4
1,00E-06	0,360599995	0,341300003	0,323100001	0,350300014
5,00E-06	0,345899999	0,315800004	0,315699995	0,316300005
1,00E-05	0,320199996	0,286000006	0,287400007	0,313300014
5,00E-05	1,306099981	1,143799953	1,12660027	1,186700016
1,00E-04	1,84950003	1,712099962	1,729799986	1,719300061
5,00E-04	0,778200001	0,707100026	0,753499985	0,783400029
1,00E-03	0,757199973	0,675500028	0,666800022	0,674200028

(540nm-690nm)-solvent control E2 reference

cocentration [M]	Replicate 1	Replicate 2	Replicate 3	Replicate 4
1,00E-09	0,408400007	0,401200011	0,419999994	0,417299978

Normalization (substance/1 nM E2)

cocentration [M]	Replicate 1	Replicate 2	Replicate 3	Replicate 4	Mean
1,00E-06	88,30%	85,07%	76,93%	83,94%	**83,56%**
5,00E-06	84,70%	78,71%	75,17%	75,80%	**78,59%**
1,00E-05	78,40%	71,29%	68,43%	75,08%	**73,30%**
5,00E-05	319,81%	285,09%	268,24%	284,38%	**289,38%**
1,00E-04	452,86%	426,74%	411,86%	412,01%	**425,87%**
5,00E-04	190,55%	176,25%	179,40%	187,73%	**183,48%**
1,00E-03	185,41%	168,37%	158,76%	161,56%	**168,53%**

yeast-growth/solubility 690nm/solvent control 690nm

cocentration [M]	Replicate 1	Replicate 2	Replicate 3	Replicate 4	Mean
1,00E-06	89,36%	90,70%	80,16%	95,22%	**88,86%**
5,00E-06	96,26%	93,33%	88,24%	93,13%	**92,74%**
1,00E-05	99,45%	84,96%	84,95%	90,29%	**89,91%**
5,00E-05	98,02%	93,41%	80,04%	98,12%	**92,40%**
1,00E-04	83,94%	80,20%	79,02%	87,04%	**82,55%**
5,00E-04	84,66%	89,89%	75,36%	84,03%	**83,49%**
1,00E-03	117,63%	105,15%	81,09%	109,33%	**103,30%**

4-HT (540nm-690nm) anti-estrogenic control

cocentration [M]	Replicate 1	Replicate 2	Replicate 3	Replicate 4
1,00E-06	0,059699997	0,062799998	0,042899996	0,054100007

4-HT (540nm-690nm)/1 nM E2 anti-estrogenic control

cocentration [M]	Replicate 1	Replicate 2	Replicate 3	Replicate 4	Mean
1,00E-06	14,62%	15,65%	10,21%	12,96%	**13,36%**

excluded

2nd experiment

540nm

cocentration [M]	Replicate 1	Replicate 2	Replicate 3	Replicate 4
1,00E-06	0,876299977	0,851100028	0,871800005	0,935100019
5,00E-06	0,799099982	0,839699984	0,818599999	0,786300004
1,00E-05	0,762099981	0,800199986	0,778100014	0,762499988
5,00E-05	2,011499882	2,029500008	1,974900007	2,088099957
1,00E-04	2,21329999	2,326100111	2,23300044	2,180599928
5,00E-04	0,940599978	0,925100029	0,939199984	0,967999995
1,00E-03	1,072399974	1,0352	1,065199971	1,09070003

690nm

cocentration [M]	Replicate 1	Replicate 2	Replicate 3	Replicate 4
1,00E-06	0,449000001	0,422699988	0,448199987	0,489600003
5,00E-06	0,406100005	0,43720001	0,425900012	0,399599999
1,00E-05	0,379900008	0,412499994	0,395399988	0,371399999
5,00E-05	0,414099991	0,441199988	0,391099989	0,435699999
1,00E-04	0,382499993	0,451200008	0,372900009	0,356599987
5,00E-04	0,275599986	0,266200006	0,262499988	0,286799997
1,00E-03	0,425199986	0,424199998	0,432300001	0,418799996

solvent control 690nm

	Replicate 1	Replicate 2	Replicate 3	Replicate 4
	0,503499985	0,542699993	0,501399994	0,505400002

540-690nm

cocentration [M]	Replicate 1	Replicate 2	Replicate 3	Replicate 4
1,00E-06	0,427300006	0,42840001	0,423599988	0,445499986
5,00E-06	0,393000007	0,402500004	0,392800003	0,386799991
1,00E-05	0,382299989	0,387699991	0,382699996	0,391099989
5,00E-05	1,59739995	1,58829999	1,583799958	1,6523
1,00E-04	1,830800056	1,874899983	1,860100031	1,824000001
5,00E-04	0,665000021	0,658999979	0,676699996	0,681200027
1,00E-03	0,647199988	0,611000001	0,6329	0,671899974

solvent control 540nm-690nm

	Replicate 1	Replicate 2	Replicate 3	Replicate 4
	0,129600003	0,131300002	0,125499994	0,125

(540nm-690nm)-solvent control (540nm-690nm)

cocentration [M]	Replicate 1	Replicate 2	Replicate 3	Replicate 4
1,00E-06	0,297700003	0,297100008	0,298099995	0,320499986
5,00E-06	0,263400003	0,271200001	0,26730001	0,261799991
1,00E-05	0,252699986	0,256399989	0,257200003	0,266099989
5,00E-05	1,467799947	1,456999987	1,458299965	1,5273
1,00E-04	1,701200053	1,743599981	1,734600037	1,699000001
5,00E-04	0,535400018	0,527699977	0,551200002	0,556200027
1,00E-03	0,517599985	0,479699999	0,507400006	0,546899974

(540nm-690nm)-solvent control E2 reference

cocentration [M]	Replicate 1	Replicate 2	Replicate 3	Replicate 4
1,00E-09	0,348099984	0,324999988	0,328999996	0,350099996

Normalization (substance/1 nM E2)

cocentration [M]	Replicate 1	Replicate 2	Replicate 3	Replicate 4	Mean
1,00E-06	85,52%	91,42%	90,61%	91,55%	**89,77%**
5,00E-06	75,67%	83,45%	81,25%	74,78%	**78,78%**
1,00E-05	72,59%	78,89%	78,18%	76,01%	**76,42%**
5,00E-05	421,66%	448,31%	443,25%	436,25%	**437,37%**
1,00E-04	488,71%	536,49%	527,23%	485,29%	**509,43%**
5,00E-04	153,81%	162,37%	167,54%	158,87%	**160,65%**
1,00E-03	148,69%	147,60%	154,22%	156,21%	**151,68%**

yeast-growth/solubility 690nm/solvent control 690nm

cocentration [M]	Replicate 1	Replicate 2	Replicate 3	Replicate 4	Mean
1,00E-06	89,18%	77,89%	89,39%	96,87%	**88,33%**
5,00E-06	80,66%	80,56%	84,94%	79,07%	**81,31%**
1,00E-05	75,45%	76,01%	78,86%	73,49%	**75,95%**
5,00E-05	82,24%	81,30%	78,00%	86,21%	**81,94%**
1,00E-04	75,97%	83,14%	74,37%	70,56%	**76,01%**
5,00E-04	54,74%	49,05%	52,35%	56,75%	**53,22%**
1,00E-03	84,45%	78,16%	86,22%	82,87%	**82,92%**

4-HT (540nm-690nm) anti-estrogenic control

cocentration [M]	Replicate 1	Replicate 2	Replicate 3	Replicate 4
1,00E-06	0,050499991	0,057300001	0,044800013	0,054100007

4-HT (540nm-690nm)/1 nM E2 anti-estrogenic control

cocentration [M]	Replicate 1	Replicate 2	Replicate 3	Replicate 4	Mean
1,00E-06	14,51%	17,63%	13,62%	15,45%	**15,30%**

3rd experiment

540nm

cocentration [M	Replicate 1	Replicate 2	Replicate 3	Replicate 4
1,00E-06	1,042000055	1,114899993	0,982299984	1,059700012
5,00E-06	1,001700044	1,001600027	1,048599958	1,019199967
1,00E-05	0,924300015	0,930899978	0,897700012	1,030300021
5,00E-05	1,924200058	2,081099987	2,023000002	2,042399883
1,00E-04	2,608400106	2,593100071	2,540999889	2,606100082
5,00E-04	1,1329	1,163599968	1,161200047	1,127900004
1,00E-03	1,230399966	1,26789999	1,263000011	1,265799999

690nm

cocentration [M	Replicate 1	Replicate 2	Replicate 3	Replicate 4
1,00E-06	0,524800003	0,562300026	0,480599999	0,528100014
5,00E-06	0,523800015	0,531099975	0,575100005	0,55369997
1,00E-05	0,495299995	0,499199986	0,470099986	0,580500007
5,00E-05	0,530499995	0,478799999	0,432700008	0,477200001
1,00E-04	0,472000003	0,466399997	0,431400001	0,459699988
5,00E-04	0,2412	0,273600012	0,29550001	0,236599997
1,00E-03	0,388900012	0,426600009	0,433099985	0,417199999

solvent control 690nm

	Replicate 1	Replicate 2	Replicate 3	Replicate 4
	0,537	0,568000019	0,574500024	0,584299982

540-690nm

cocentration [M	Replicate 1	Replicate 2	Replicate 3	Replicate 4
1,00E-06	0,517199993	0,552600026	0,501699984	0,531599998
5,00E-06	0,477899998	0,470499992	0,473500013	0,465499997
1,00E-05	0,42899999	0,431699991	0,427599996	0,449800014
5,00E-05	1,39380002	1,602300048	1,590299964	1,565299988
1,00E-04	2,136499882	2,12680006	2,109600067	2,146399975
5,00E-04	0,891700029	0,889999986	0,865700006	0,89139998
1,00E-03	0,841499984	0,841300011	0,829900026	0,84859997

solvent control 540nm-690nm

	Replicate 1	Replicate 2	Replicate 3	Replicate 4
	0,105700001	0,108999997	0,108199999	0,109800003

(540nm-690nm)-solvent control (540nm-690nm)

cocentration [M	Replicate 1	Replicate 2	Replicate 3	Replicate 4
1,00E-06	0,411499992	0,443600029	0,393499985	0,421799995
5,00E-06	0,372199997	0,361499995	0,365300015	0,355699994
1,00E-05	0,323299989	0,322699994	0,319399998	0,340000011
5,00E-05	1,288100019	1,49330005	1,482099965	1,455499984
1,00E-04	2,030799881	2,017800063	2,001400068	2,036599971
5,00E-04	0,786000028	0,780999988	0,757500008	0,781599976
1,00E-03	0,735799983	0,732300013	0,721700028	0,738799967

(540nm-690nm)-solvent control E2 reference

cocentration [M	Replicate 1	Replicate 2	Replicate 3	Replicate 4
1,00E-09	0,482899994	0,438600004	0,426799983	0,468699992

Normalization (substance/1 nM E2)

cocentration [M	Replicate 1	Replicate 2	Replicate 3	Replicate 4	Mean
1,00E-06	85,21%	101,14%	92,20%	89,99%	**92,14%**
5,00E-06	77,08%	82,42%	85,59%	75,89%	**80,24%**
1,00E-05	66,95%	73,58%	74,84%	72,54%	**71,98%**
5,00E-05	266,74%	340,47%	347,26%	310,54%	**316,25%**
1,00E-04	420,54%	460,05%	468,93%	434,52%	**446,01%**
5,00E-04	162,77%	178,07%	177,48%	166,76%	**171,27%**
1,00E-03	152,37%	166,96%	169,10%	157,63%	**161,51%**

yeast-growth/solubility 690nm/solvent control 690nm

cocentration [M	Replicate 1	Replicate 2	Replicate 3	Replicate 4	Mean
1,00E-06	97,73%	99,00%	83,66%	90,38%	**92,69%**
5,00E-06	97,54%	93,50%	100,10%	94,76%	**96,48%**
1,00E-05	92,23%	87,89%	81,83%	99,35%	**90,32%**
5,00E-05	98,79%	84,30%	75,32%	81,67%	**85,02%**
1,00E-04	87,90%	82,11%	75,09%	78,68%	**80,94%**
5,00E-04	44,92%	48,17%	51,44%	40,49%	**46,25%**
1,00E-03	72,42%	75,11%	75,39%	71,40%	**73,58%**

4-HT (540nm-690nm) anti-estrogenic control

cocentration [M	Replicate 1	Replicate 2	Replicate 3	Replicate 4
1,00E-06	0,077700004	0,073200002	0,077900007	0,079599999

4-HT (540nm-690nm)/1 nM E2 anti-estrogenic control

cocentration [M	Replicate 1	Replicate 2	Replicate 3	Replicate 4	Mean
1,00E-06	16,09%	16,69%	18,25%	16,98%	**17,00%**

4th experiment

540nm

cocentration [M	Replicate 1	Replicate 2	Replicate 3	Replicate 4
1,00E-06	1,195600033	1,165099978	1,233199954	1,298799992
5,00E-06	1,096500039	1,201799989	1,223700047	1,212000012
1,00E-05	1,046599984	1,20510006	1,162500024	1,142699957
5,00E-05	2,16989994	2,162199974	2,299900055	2,280800104
1,00E-04	2,44569993	2,483500004	2,480400085	2,455800056
5,00E-04	1,091899991	1,105800033	1,133299947	1,15320003
1,00E-03	1,263599992	1,27489996	1,199599981	1,29400003

690nm

cocentration [M	Replicate 1	Replicate 2	Replicate 3	Replicate 4
1,00E-06	0,606400013	0,574100018	0,644599974	0,647800028
5,00E-06	0,541700006	0,630200028	0,646899998	0,639400005
1,00E-05	0,550199986	0,655300021	0,620800018	0,610400021
5,00E-05	0,486799985	0,516300023	0,561800003	0,566299975
1,00E-04	0,471899986	0,495999992	0,497599989	0,488799989
5,00E-04	0,196099997	0,200900003	0,186199993	0,238199994
1,00E-03	0,399500012	0,393599987	0,308099985	0,426099986

solvent control 690nm

	Replicate 1	Replicate 2	Replicate 3	Replicate 4
	0,649100006	0,713999987	0,659399986	0,601300001

540-690nm

cocentration [M	Replicate 1	Replicate 2	Replicate 3	Replicate 4
1,00E-06	0,58920002	0,591000021	0,588699996	0,651000023
5,00E-06	0,554799974	0,57160002	0,576799989	0,572600007
1,00E-05	0,496399999	0,549799979	0,541700006	0,532199979
5,00E-05	1,683099985	1,645900011	1,738100052	1,71449995
1,00E-04	1,973799944	1,987499952	1,982800007	1,967000008
5,00E-04	0,895799994	0,904900014	0,947000027	0,915000021
1,00E-03	0,864099979	0,881299973	0,891499996	0,867999971

solvent control 540nm-690nm

	Replicate 1	Replicate 2	Replicate 3	Replicate 4
	0,098700002	0,105800003	0,100500003	0,097499996

(540nm-690nm)-solvent control (540nm-690nm)

cocentration [M	Replicate 1	Replicate 2	Replicate 3	Replicate 4
1,00E-06	0,490500018	0,485200018	0,488199994	0,553500026
5,00E-06	0,456099972	0,465800017	0,476299986	0,475100011
1,00E-05	0,397699997	0,443999976	0,441200003	0,434699982
5,00E-05	1,584399983	1,540100008	1,637600049	1,616999954
1,00E-04	1,875099942	1,88169995	1,882300004	1,869500011
5,00E-04	0,797099993	0,799100012	0,846500024	0,817500025
1,00E-03	0,765399978	0,77549997	0,790999994	0,770499974

(540nm-690nm)-solvent control E2 reference

cocentration [M	Replicate 1	Replicate 2	Replicate 3	Replicate 4
1,00E-09	0,53770002	0,519900024	0,550800011	0,51920002

Normalization (substance/1 nM E2)

cocentration [M	Replicate 1	Replicate 2	Replicate 3	Replicate 4	Mean
1,00E-06	91,22%	93,33%	88,63%	106,61%	**94,95%**
5,00E-06	84,82%	89,59%	86,47%	91,51%	**88,10%**
1,00E-05	73,96%	85,40%	80,10%	83,72%	**80,80%**
5,00E-05	294,66%	296,23%	297,31%	311,44%	**299,91%**
1,00E-04	348,73%	361,93%	341,74%	360,07%	**353,12%**
5,00E-04	148,24%	153,70%	153,69%	157,45%	**153,27%**
1,00E-03	142,35%	149,16%	143,61%	148,40%	**145,88%**

yeast-growth/solubility 690nm/solvent control 690nm

cocentration [M	Replicate 1	Replicate 2	Replicate 3	Replicate 4	Mean
1,00E-06	93,42%	80,41%	97,76%	107,73%	**94,83%**
5,00E-06	83,45%	88,26%	98,10%	106,34%	**94,04%**
1,00E-05	84,76%	91,78%	94,15%	101,51%	**93,05%**
5,00E-05	75,00%	72,31%	85,20%	94,18%	**81,67%**
1,00E-04	72,70%	69,47%	75,46%	81,29%	**74,73%**
5,00E-04	30,21%	28,14%	28,24%	39,61%	**31,55%**
1,00E-03	61,55%	55,13%	46,72%	70,86%	**58,57%**

4-HT (540nm-690nm) anti-estrogenic control

cocentration [M	Replicate 1	Replicate 2	Replicate 3	Replicate 4
1,00E-06	0,075900003	0,065099999	0,0854	0,079700008

4-HT (540nm-690nm)/1 nM E2 anti-estrogenic control

cocentration [M	Replicate 1	Replicate 2	Replicate 3	Replicate 4	Mean
1,00E-06	14,12%	12,52%	15,50%	15,35%	**14,37%**

5th experiment

540nm

cocentration [M	Replicate 1	Replicate 2	Replicate 3	Replicate 4
1,00E-06	1,089900017	1,023200035	1,019400001	1,001700044
5,00E-06	0,999199986	1,026600003	0,958000004	1,014000058
1,00E-05	0,981700003	1,006999969	1,005499959	0,958000004
5,00E-05	2,3756001	2,371400118	2,384399891	2,357199907
1,00E-04	2,66230011	2,648299932	2,713399887	2,638000011
5,00E-04	1,122499943	1,201200008	1,213099957	1,239599943
1,00E-03	1,345299959	1,353800058	1,347900033	1,340399981

690nm

cocentration [M	Replicate 1	Replicate 2	Replicate 3	Replicate 4
1,00E-06	0,51730001	0,479699999	0,473399997	0,46540001
5,00E-06	0,491400003	0,505200028	0,467200011	0,493900001
1,00E-05	0,487399995	0,501299977	0,501399994	0,475300014
5,00E-05	0,449699998	0,454899997	0,443899989	0,415699989
1,00E-04	0,397300005	0,383599997	0,445199996	0,358900011
5,00E-04	0,205699995	0,227300003	0,227599993	0,232999995
1,00E-03	0,433699995	0,398699999	0,418300003	0,397500008

solvent control 690nm

	Replicate 1	Replicate 2	Replicate 3	Replicate 4
	0,615499973	0,605599999	0,624599993	0,570500016

540-690nm

cocentration [M	Replicate 1	Replicate 2	Replicate 3	Replicate 4
1,00E-06	0,57249999	0,543500006	0,54610002	0,536300004
5,00E-06	0,507799983	0,521399975	0,490799993	0,520099998
1,00E-05	0,494300008	0,505699992	0,504100025	0,48269999
5,00E-05	1,925899982	1,916499972	1,940500021	1,941499949
1,00E-04	2,264899969	2,264699936	2,268199921	2,279099941
5,00E-04	0,916700006	0,973999977	0,985499978	1,006600022
1,00E-03	0,911599994	0,9551	0,9296	0,942799985

solvent control 540nm-690nm

	Replicate 1	Replicate 2	Replicate 3	Replicate 4
	0,107000001	0,109999999	0,110699996	0,104400001

(540nm-690nm)-solvent control (540nm-690nm)

cocentration [M	Replicate 1	Replicate 2	Replicate 3	Replicate 4
1,00E-06	0,46549999	0,433500007	0,435400024	0,431900002
5,00E-06	0,400799982	0,411399975	0,380099997	0,415699996
1,00E-05	0,387300007	0,395699993	0,393400028	0,378299989
5,00E-05	1,818899982	1,806499973	1,829800025	1,837099947
1,00E-04	2,157899968	2,154699937	2,157499924	2,17469994
5,00E-04	0,809700005	0,863999978	0,874799982	0,902200021
1,00E-03	0,804599993	0,845100001	0,818900004	0,838399984

(540nm-690nm)-solvent control E2 reference

cocentration [M	Replicate 1	Replicate 2	Replicate 3	Replicate 4
1,00E-09	0,486399978	0,481599972	0,496199995	0,475299977

Normalization (substance/1 nM E2)

cocentration [M	Replicate 1	Replicate 2	Replicate 3	Replicate 4	**Mean**
1,00E-06	95,70%	90,01%	87,75%	90,87%	**91,08%**
5,00E-06	82,40%	85,42%	76,60%	87,46%	**82,97%**
1,00E-05	79,63%	82,16%	79,28%	79,59%	**80,17%**
5,00E-05	373,95%	375,10%	368,76%	386,51%	**376,08%**
1,00E-04	443,65%	447,40%	434,80%	457,54%	**445,85%**
5,00E-04	166,47%	179,40%	176,30%	189,82%	**178,00%**
1,00E-03	165,42%	175,48%	165,03%	176,39%	**170,58%**

yeast-growth/solubility 690nm/solvent control 690nm

cocentration [M	Replicate 1	Replicate 2	Replicate 3	Replicate 4	**Mean**
1,00E-06	84,05%	79,21%	75,79%	81,58%	**80,16%**
5,00E-06	79,84%	83,42%	74,80%	86,57%	**81,16%**
1,00E-05	79,19%	82,78%	80,28%	83,31%	**81,39%**
5,00E-05	73,06%	75,12%	71,07%	72,87%	**73,03%**
1,00E-04	64,55%	63,34%	71,28%	62,91%	**65,52%**
5,00E-04	33,42%	37,53%	36,44%	40,84%	**37,06%**
1,00E-03	70,46%	65,84%	66,97%	69,68%	**68,24%**

4-HT (540nm-690nm) anti-estrogenic control

cocentration [M	Replicate 1	Replicate 2	Replicate 3	Replicate 4
1,00E-06	0,052099995	0,057699993	0,050400004	0,064900003

4-HT (540nm-690nm)/1 nM E2 anti-estrogenic control

cocentration [M	Replicate 1	Replicate 2	Replicate 3	Replicate 4	**Mean**
1,00E-06	10,71%	11,98%	10,16%	13,65%	**11,63%**

cell-free 690nm

cocentration [M	Replicate 1	Replicate 2	Replicate 3	Replicate 4
1,00E-06	0,037799999	0,037799999	0,037	0,037500001
5,00E-06	0,0374	0,0374	0,0374	0,0372
1,00E-05	0,037500001	0,038199998	0,037300002	0,036899999
5,00E-05	0,0381	0,0458	0,037500001	0,0374
1,00E-04	0,0381	0,045499999	0,041000001	0,037799999
5,00E-04	0,286199987	0,247700006	0,210299999	0,2377
1,00E-03	0,832599998	0,578999996	0,703000009	0,666599989

cell-free solvent control 690nm

cocentration [M	Replicate 1	Replicate 2	Replicate 3	Replicate 4
	0,0372	0,039700001	0,037599999	0,038400002

cell-free (substance/solvent control)

cocentration [M	Replicate 1	Replicate 2	Replicate 3	Replicate 4	**Mean**
1,00E-06	101,61%	95,21%	98,40%	97,66%	**98,22%**
5,00E-06	100,54%	94,21%	99,47%	96,87%	**97,77%**
1,00E-05	100,81%	96,22%	99,20%	96,09%	**98,08%**
5,00E-05	102,42%	115,37%	99,73%	97,40%	**103,73%**
1,00E-04	102,42%	114,61%	109,04%	98,44%	**106,13%**
5,00E-04	769,35%	623,93%	559,31%	619,01%	**642,90%**
1,00E-03	2238,17%	1458,44%	1869,68%	1735,94%	**1825,56%**

YES fludioxonil + E2

1st experiment

540nm

cocentration [M]	Replicate 1	Replicate 2	Replicate 3	Replicate 4
1,00E-07	0,9454	0,952499986	0,942700028	0,936800003
1,00E-06	0,9454	0,979700029	0,919200003	0,936999977
1,00E-05	0,688899994	0,732200027	0,729700029	0,693300009
5,00E-05	0,685400009	0,673900008	0,741900027	0,709900022
1,00E-04	0,701099992	0,710900009	0,761900008	0,746800005
5,00E-04	0,913900018	0,988300025	1,00849998	1,102800012
1,00E-03	1,267199993	1,345999956	1,365100026	1,310899973

690nm

cocentration [M]	Replicate 1	Replicate 2	Replicate 3	Replicate 4
1,00E-07	0,438300014	0,431600004	0,420700014	0,416500002
1,00E-06	0,439500004	0,471799999	0,415199995	0,427700013
1,00E-05	0,34040001	0,379000008	0,362899995	0,321700007
5,00E-05	0,372999996	0,334300011	0,370299995	0,360399991
1,00E-04	0,346199989	0,352499992	0,363700002	0,353199989
5,00E-04	0,470800012	0,492399991	0,523699999	0,515399992
1,00E-03	0,662199974	0,707000017	0,573000014	0,666400015

solvent control 690nm

	Replicate 1	Replicate 2	Replicate 3	Replicate 4
	0,495200008	0,476999998	0,495099992	0,480300009

540-690nm

cocentration [M]	Replicate 1	Replicate 2	Replicate 3	Replicate 4
1,00E-07	0,507099986	0,520900011	0,521899998	0,520200014
1,00E-06	0,505800009	0,5079	0,503899992	0,509199977
1,00E-05	0,348500013	0,353300005	0,366699994	0,371600002
5,00E-05	0,3125	0,339599997	0,371499985	0,349400014
1,00E-04	0,354900002	0,358399987	0,398200005	0,3935
5,00E-04	0,443199992	0,495900005	0,484800011	0,587400019
1,00E-03	0,605000019	0,638999999	0,792100012	0,644400001

solvent control 540nm-690nm

	Replicate 1	Replicate 2	Replicate 3	Replicate 4
	0,128900006	0,126200005	0,1294	0,121399999

(540nm-690nm)-solvent control (540nm-690nm)

cocentration [M]	Replicate 1	Replicate 2	Replicate 3	Replicate 4
1,00E-07	0,37819998	0,394700006	0,392499998	0,398800015
1,00E-06	0,376900002	0,381699994	0,374499992	0,387799978
1,00E-05	0,219600007	0,2271	0,237299994	0,250200003
5,00E-05	0,183599994	0,213399991	0,242099985	0,228000015
1,00E-04	0,225999996	0,232199982	0,268800005	0,272100002
5,00E-04	0,314299986	0,3697	0,355400011	0,466000021
1,00E-03	0,476100013	0,512799993	0,662700012	0,523000002

(540nm-690nm)-solvent control E2 reference

cocentration [M]	Replicate 1	Replicate 2	Replicate 3	Replicate 4
1,00E-09	0,394699976	0,422400028	0,38970001	0,397199973

Normalization (substance/1 nM E2)

cocentration [M]	Replicate 1	Replicate 2	Replicate 3	Replicate 4	Mean
1,00E-07	95,82%	93,44%	100,72%	100,40%	**97,60%**
1,00E-06	95,49%	90,36%	96,10%	97,63%	**94,90%**
1,00E-05	55,64%	53,76%	60,89%	62,99%	**58,32%**
5,00E-05	46,52%	50,52%	62,12%	57,40%	**54,14%**
1,00E-04	57,26%	54,97%	68,98%	68,50%	**62,43%**
5,00E-04	79,63%	87,52%	91,20%	117,32%	**93,92%**
1,00E-03	120,62%	121,40%	170,05%	131,67%	**135,94%**

yeast-growth/solubility 690nm/solvent control 690nm

cocentration [M]	Replicate 1	Replicate 2	Replicate 3	Replicate 4	Mean
1,00E-07	88,51%	90,48%	84,97%	86,72%	**87,67%**
1,00E-06	88,75%	98,91%	83,86%	89,05%	**90,14%**
1,00E-05	68,74%	79,45%	73,30%	66,98%	**72,12%**
5,00E-05	75,32%	70,08%	74,79%	75,04%	**73,81%**
1,00E-04	95,07%	103,23%	105,78%	107,31%	**102,85%**
5,00E-04	69,91%	73,90%	73,46%	73,54%	**72,70%**
1,00E-03	133,72%	148,22%	115,73%	138,75%	**134,11%**

4-HT (540nm-690nm) anti-estrogenic control

cocentration [M]	Replicate 1	Replicate 2	Replicate 3	Replicate 4
1,00E-06	0,056099996	0,061299995	0,0528	0,066699997

4-HT (540nm-690nm)/1 nM E2 anti-estrogenic control

cocentration [M]	Replicate 1	Replicate 2	Replicate 3	Replicate 4	Mean
1,00E-06	14,21%	14,51%	13,55%	16,79%	**14,77%**

excluded

2nd experiment

540nm

cocentration [M]	Replicate 1	Replicate 2	Replicate 3	Replicate 4
1,00E-07	0,855599999	0,848800004	0,826600015	0,840399981
1,00E-06	0,795899987	1,036299944	0,828499973	0,80309999
1,00E-05	0,632200003	0,635699987	0,646200001	0,625
5,00E-05	0,655399978	0,645299971	0,666199982	0,616599977
1,00E-04	0,680999994	0,695500016	0,699400008	0,638400018
5,00E-04	0,785399973	0,720300019	0,78640002	0,771200001
1,00E-03	0,939400017	0,999499977	0,99150002	0,913100004

690nm

cocentration [M]	Replicate 1	Replicate 2	Replicate 3	Replicate 4
1,00E-07	0,42809999	0,399300009	0,411599994	0,419400007
1,00E-06	0,403600007	0,544700027	0,414799988	0,399899989
1,00E-05	0,317499995	0,313800007	0,330000013	0,319799995
5,00E-05	0,336299986	0,344700009	0,363599986	0,325300008
1,00E-04	0,371800005	0,379500002	0,396200001	0,351399988
5,00E-04	0,458999991	0,413399994	0,417899996	0,445100009
1,00E-03	0,573700011	0,620800018	0,605099976	0,550999999

solvent control 690nm

	Replicate 1	Replicate 2	Replicate 3	Replicate 4
	0,493200004	0,502900004	0,472799987	0,513000011

540-690nm

cocentration [M]	Replicate 1	Replicate 2	Replicate 3	Replicate 4
1,00E-07	0,42750001	0,449499995	0,415100008	0,421000004
1,00E-06	0,39230001	0,491600007	0,413599998	0,403200001
1,00E-05	0,314799994	0,32190001	0,316100001	0,305200011
5,00E-05	0,319000006	0,300599992	0,302599996	0,291299999
1,00E-04	0,309199989	0,316000015	0,303200006	0,287
5,00E-04	0,326299995	0,306800008	0,368499994	0,326099992
1,00E-03	0,365700006	0,378699988	0,386400014	0,362199992

solvent control 540nm-690nm

	Replicate 1	Replicate 2	Replicate 3	Replicate 4
	0,128800005	0,125400007	0,123599999	0,118299998

(540nm-690nm)-solvent control (540nm-690nm)

cocentration [M]	Replicate 1	Replicate 2	Replicate 3	Replicate 4
1,00E-07	0,357900009	0,357799999	0,363000005	0,356999986
1,00E-06	0,369299993	0,350899987	0,390000015	0,35140001
1,00E-05	0,634399995	0,38479998	0,379099995	0,358900003
5,00E-05	0,325200006	0,336300008	0,316100001	0,324800007
1,00E-04	0,354100004	0,396200024	0,372400016	0,360299997
5,00E-04	0,507099971	0,428199969	0,445000023	0,39479997
1,00E-03	0,661100015	0,677299999	0,60679999	0,679799996

(540nm-690nm)-solvent control E2 reference

cocentration [M]	Replicate 1	Replicate 2	Replicate 3	Replicate 4
1,00E-09	0,432500005	0,401799969	0,412900023	0,42240002

Normalization (substance/1 nM E2)

cocentration [M]	Replicate 1	Replicate 2	Replicate 3	Replicate 4	Mean
1,00E-07	82,75%	89,05%	87,91%	84,52%	**86,06%**
1,00E-06	85,39%	87,33%	94,45%	83,19%	**87,59%**
1,00E-05	146,68%	95,77%	91,81%	84,97%	**104,81%**
5,00E-05	75,19%	83,70%	76,56%	76,89%	**78,08%**
1,00E-04	81,87%	98,61%	90,19%	85,30%	**88,99%**
5,00E-04	117,25%	106,57%	107,77%	93,47%	**106,26%**
1,00E-03	152,86%	168,57%	146,96%	160,94%	**157,33%**

yeast-growth/solubility 690nm/solvent control 690nm

cocentration [M]	Replicate 1	Replicate 2	Replicate 3	Replicate 4	Mean
1,00E-07	86,80%	79,40%	87,06%	81,75%	**83,75%**
1,00E-06	81,83%	108,31%	87,73%	77,95%	**88,96%**
1,00E-05	64,38%	62,40%	69,80%	62,34%	**64,73%**
5,00E-05	68,19%	68,54%	76,90%	63,41%	**69,26%**
1,00E-04	75,39%	75,46%	83,80%	68,50%	**75,79%**
5,00E-04	93,07%	82,20%	88,39%	86,76%	**87,61%**
1,00E-03	116,32%	123,44%	127,98%	107,41%	**118,79%**

4-HT (540nm-690nm) anti-estrogenic control

cocentration [M]	Replicate 1	Replicate 2	Replicate 3	Replicate 4
1,00E-06	0,0546	0,055599995	0,051100001	0,049900003

4-HT (540nm-690nm)/1 nM E2 anti-estrogenic control

cocentration [M]	Replicate 1	Replicate 2	Replicate 3	Replicate 4	Mean
1,00E-06	12,62%	13,84%	12,38%	11,81%	**12,66%**

3rd experiment

540nm

cocentration [M	Replicate 1	Replicate 2	Replicate 3	Replicate 4
1,00E-07	1,09679997	1,07249999	1,087100029	1,056499958
1,00E-06	1,043400049	1,044800043	1,056100011	1,053599954
1,00E-05	0,797599971	0,827199996	0,765699983	0,814899981
5,00E-05	0,857800007	0,754599988	0,732599974	0,76910001
1,00E-04	0,828400016	0,783100009	0,848800004	0,783399999
5,00E-04	0,905900002	0,886900008	0,868399978	0,8671
1,00E-03	1,058300018	0,923900008	1,130300045	1,091799974

690nm

cocentration [M	Replicate 1	Replicate 2	Replicate 3	Replicate 4
1,00E-07	0,566999972	0,538900018	0,568700016	0,506799996
1,00E-06	0,53670001	0,513599992	0,537299991	0,543799996
1,00E-05	0,485700011	0,452199996	0,438699991	0,467500001
5,00E-05	0,526499987	0,443899989	0,416599989	0,4463
1,00E-04	0,463200003	0,448100001	0,512300014	0,459600002
5,00E-04	0,555299997	0,511799991	0,521899998	0,52700001
1,00E-03	0,610800028	0,416200012	0,645099998	0,669799984

solvent control 690nm

	Replicate 1	Replicate 2	Replicate 3	Replicate 4
	0,531599998	0,583199978	0,567600012	0,568099976

540-690nm

cocentration [M	Replicate 1	Replicate 2	Replicate 3	Replicate 4
1,00E-07	0,529900014	0,533599973	0,518400013	0,549700022
1,00E-06	0,506699979	0,531199992	0,51880002	0,509800017
1,00E-05	0,31189999	0,375	0,327100009	0,34740001
5,00E-05	0,33129999	0,310600013	0,316000015	0,32280001
1,00E-04	0,365200013	0,335000008	0,336400002	0,323799998
5,00E-04	0,350600004	0,375099987	0,346500009	0,34009999
1,00E-03	0,44749999	0,507700026	0,485100001	0,422100008

solvent control 540nm-690nm

	Replicate 1	Replicate 2	Replicate 3	Replicate 4
	0,104599997	0,112000003	0,1083	0,108000003

(540nm-690nm)-solvent control (540nm-690nm)

cocentration [M	Replicate 1	Replicate 2	Replicate 3	Replicate 4
1,00E-07	0,425300017	0,421599969	0,410100013	0,441700019
1,00E-06	0,402099982	0,419199988	0,41050002	0,401800014
1,00E-05	0,207299992	0,262999997	0,218800008	0,239400007
5,00E-05	0,226699993	0,198600009	0,207700014	0,214800008
1,00E-04	0,260600016	0,223000005	0,228100002	0,215799995
5,00E-04	0,246000007	0,263099983	0,238200009	0,232099988
1,00E-03	0,342899993	0,395700023	0,376800001	0,314100005

(540nm-690nm)-solvent control E2 reference

cocentration [M	Replicate 1	Replicate 2	Replicate 3	Replicate 4
1,00E-09	0,434100002	0,46790003	0,459899977	0,454300001

Normalization (substance/1 nM E2)

cocentration [M	Replicate 1	Replicate 2	Replicate 3	Replicate 4	Mean
1,00E-07	97,97%	90,10%	89,17%	97,23%	**93,62%**
1,00E-06	92,63%	89,59%	89,26%	88,44%	**89,98%**
1,00E-05	47,75%	56,21%	47,58%	52,70%	**51,06%**
5,00E-05	52,22%	42,44%	45,16%	47,28%	**46,78%**
1,00E-04	60,03%	47,66%	49,60%	47,50%	**51,20%**
5,00E-04	56,67%	56,23%	51,79%	51,09%	**53,95%**
1,00E-03	78,99%	84,57%	81,93%	69,14%	**78,66%**

yeast-growth/solubility 690nm/solvent control 690nm

cocentration [M	Replicate 1	Replicate 2	Replicate 3	Replicate 4	Mean
1,00E-07	106,66%	92,40%	100,19%	89,21%	**97,12%**
1,00E-06	100,96%	88,07%	94,66%	95,72%	**94,85%**
1,00E-05	91,37%	77,54%	77,29%	82,29%	**82,12%**
5,00E-05	99,04%	76,11%	73,40%	78,56%	**81,78%**
1,00E-04	87,13%	76,83%	90,26%	80,90%	**83,78%**
5,00E-04	104,46%	87,76%	91,95%	92,77%	**94,23%**
1,00E-03	114,90%	71,36%	113,65%	117,90%	**104,45%**

4-HT (540nm-690nm) anti-estrogenic control

cocentration [M	Replicate 1	Replicate 2	Replicate 3	Replicate 4
1,00E-06	0,078099996	0,064499989	0,074499995	0,084799998

4-HT (540nm-690nm)/1 nM E2 anti-estrogenic control

cocentration [M	Replicate 1	Replicate 2	Replicate 3	Replicate 4	Mean
1,00E-06	17,99%	13,78%	16,20%	18,67%	**16,66%**

4th experiment

540nm

cocentration [M	Replicate 1	Replicate 2	Replicate 3	Replicate 4
1,00E-07	1,065500021	1,155300021	1,131800056	1,143700004
1,00E-06	1,060299993	1,066200018	1,021499991	1,101400018
1,00E-05	0,77759999	0,877699971	0,816699982	0,92019999
5,00E-05	0,827199996	0,827199996	0,842000008	0,807699978
1,00E-04	0,869400024	0,907400012	0,915600002	0,836000025
5,00E-04	0,999199986	0,960900009	1,026600003	0,997600019
1,00E-03	1,122900009	1,186400056	1,118999958	1,083600044

690nm

cocentration [M	Replicate 1	Replicate 2	Replicate 3	Replicate 4
1,00E-07	0,496100008	0,513700008	0,563399971	0,539499998
1,00E-06	0,514900029	0,496699989	0,470600009	0,529100001
1,00E-05	0,411199987	0,479900002	0,423099995	0,519299984
5,00E-05	0,473500013	0,460299999	0,491100013	0,455199987
1,00E-04	0,497900009	0,551199973	0,555599988	0,474000007
5,00E-04	0,585600019	0,5528	0,602299988	0,580600023
1,00E-03	0,683499992	0,746200025	0,684300005	0,648899972

solvent control 690nm

	Replicate 1	Replicate 2	Replicate 3	Replicate 4
	0,631900012	0,578299999	0,625100017	0,606999993

540-690nm

cocentration [M	Replicate 1	Replicate 2	Replicate 3	Replicate 4
1,00E-07	0,569500029	0,641600013	0,568400025	0,604099989
1,00E-06	0,545300007	0,569500029	0,550800025	0,572300017
1,00E-05	0,366400003	0,397799999	0,3935	0,400900006
5,00E-05	0,353599995	0,366899997	0,350899994	0,352499992
1,00E-04	0,371499985	0,35620001	0,359899998	0,361999989
5,00E-04	0,413599998	0,407999992	0,424299985	0,416999996
1,00E-03	0,439399987	0,440200001	0,434700012	0,434700012

solvent control 540nm-690nm

	Replicate 1	Replicate 2	Replicate 3	Replicate 4
	0,0942	0,091300003	0,093500003	0,097999997

(540nm-690nm)-solvent control (540nm-690nm)

cocentration [M	Replicate 1	Replicate 2	Replicate 3	Replicate 4
1,00E-07	0,475300029	0,55030001	0,474900022	0,506099992
1,00E-06	0,451100007	0,478200026	0,457300022	0,474300019
1,00E-05	0,272200003	0,306499995	0,299999997	0,302900009
5,00E-05	0,259399995	0,275599994	0,257399991	0,254499994
1,00E-04	0,277299985	0,264900006	0,266399994	0,263999991
5,00E-04	0,319399998	0,316699989	0,330799982	0,318999998
1,00E-03	0,345199987	0,348899998	0,341200009	0,336700015

(540nm-690nm)-solvent control E2 reference

cocentration [M	Replicate 1	Replicate 2	Replicate 3	Replicate 4
1,00E-09	0,531699978	0,511200018	0,53269998	0,548800014

Normalization (substance/1 nM E2)

cocentration [M	Replicate 1	Replicate 2	Replicate 3	Replicate 4	Mean
1,00E-07	89,39%	107,65%	89,15%	92,22%	**94,60%**
1,00E-06	84,84%	93,54%	85,85%	86,42%	**87,66%**
1,00E-05	51,19%	59,96%	56,32%	55,19%	**55,67%**
5,00E-05	48,79%	53,91%	48,32%	46,37%	**49,35%**
1,00E-04	52,15%	51,82%	50,01%	48,10%	**50,52%**
5,00E-04	60,07%	61,95%	62,10%	58,13%	**60,56%**
1,00E-03	64,92%	68,25%	64,05%	61,35%	**64,64%**

yeast-growth/solubility 690nm/solvent control 690nm

cocentration [M	Replicate 1	Replicate 2	Replicate 3	Replicate 4	Mean
1,00E-07	78,51%	88,83%	90,13%	88,88%	**86,59%**
1,00E-06	81,48%	85,89%	75,28%	87,17%	**82,46%**
1,00E-05	65,07%	82,98%	67,69%	85,55%	**75,32%**
5,00E-05	74,93%	79,60%	78,56%	74,99%	**77,02%**
1,00E-04	78,79%	95,31%	88,88%	78,09%	**85,27%**
5,00E-04	92,67%	95,59%	96,35%	95,65%	**95,07%**
1,00E-03	108,17%	129,03%	109,47%	106,90%	**113,39%**

4-HT (540nm-690nm) anti-estrogenic control

cocentration [M	Replicate 1	Replicate 2	Replicate 3	Replicate 4
1,00E-06	0,075800002	0,081999995	0,084999993	0,078599997

4-HT (540nm-690nm)/1 nM E2 anti-estrogenic control

cocentration [M	Replicate 1	Replicate 2	Replicate 3	Replicate 4	Mean
1,00E-06	14,26%	16,04%	15,96%	14,32%	**15,14%**

5th experiment

540nm

cocentration [M	Replicate 1	Replicate 2	Replicate 3	Replicate 4
1,00E-07	0,600600004	1,478800058	1,051300049	1,042099953
1,00E-06	1,068699956	1,046000004	1,004600048	1,011199951
1,00E-05	0,823300004	0,815199971	0,833800018	0,808399975
5,00E-05	0,827899992	0,84740001	0,820299983	0,788800001
1,00E-04	0,843200028	0,851800025	0,814700007	0,833700001
5,00E-04	0,965300024	0,973100007	0,968900025	1,022300005
1,00E-03	1,133399963	1,193600059	1,110900044	1,161499977

690nm

cocentration [M	Replicate 1	Replicate 2	Replicate 3	Replicate 4
1,00E-07	0,0515	0,665899992	0,495599985	0,483099997
1,00E-06	0,529699981	0,504499972	0,476900011	0,486000001
1,00E-05	0,435200006	0,41870001	0,435200006	0,420100003
5,00E-05	0,454299986	0,469799995	0,457300007	0,432099998
1,00E-04	0,46329999	0,459600002	0,440299988	0,458499998
5,00E-04	0,538699985	0,534099996	0,539099991	0,593699992
1,00E-03	0,654699981	0,698099971	0,634000003	0,676400006

solvent control 690nm

	Replicate 1	Replicate 2	Replicate 3	Replicate 4
	0,633899987	0,6426	0,634800017	0,583999991

540-690nm

cocentration [M	Replicate 1	Replicate 2	Replicate 3	Replicate 4
1,00E-07	0,549099982	0,812900007	0,555700004	0,559000015
1,00E-06	0,538999975	0,541499972	0,527700007	0,525200009
1,00E-05	0,388099998	0,396400005	0,398600012	0,388300002
5,00E-05	0,373699993	0,377600014	0,363000005	0,356700003
1,00E-04	0,379799992	0,392100006	0,374500006	0,375200003
5,00E-04	0,426600009	0,43900001	0,429800004	0,428600013
1,00E-03	0,478799999	0,495499998	0,476799995	0,485000014

solvent control 540nm-690nm

	Replicate 1	Replicate 2	Replicate 3	Replicate 4
	0,112499997	0,112599999	0,112499997	0,107799999

(540nm-690nm)-solvent control (540nm-690nm)

cocentration [M	Replicate 1	Replicate 2	Replicate 3	Replicate 4
1,00E-07	0,436599985	0,700300008	0,443200007	0,451200016
1,00E-06	0,426499978	0,428899974	0,41520001	0,41740001
1,00E-05	0,275600001	0,283800006	0,286100015	0,280500002
5,00E-05	0,261199996	0,265000015	0,250500008	0,248900004
1,00E-04	0,267299995	0,279500008	0,262000009	0,267400004
5,00E-04	0,314100012	0,326400012	0,317300007	0,320800014
1,00E-03	0,366300002	0,3829	0,364299998	0,377200015

(540nm-690nm)-solvent control E2 reference

cocentration [M	Replicate 1	Replicate 2	Replicate 3	Replicate 4
1,00E-09	0,467500024	0,534699976	0,461499989	0,449799977

Normalization (substance/1 nM E2)

cocentration [M	Replicate 1	Replicate 2	Replicate 3	Replicate 4	Mean
1,00E-07	93,39%	130,97%	96,03%	100,31%	**105,18%**
1,00E-06	91,23%	80,21%	89,97%	92,80%	**88,55%**
1,00E-05	58,95%	53,08%	61,99%	62,36%	**59,10%**
5,00E-05	55,87%	49,56%	54,28%	55,34%	**53,76%**
1,00E-04	57,18%	52,27%	56,77%	59,45%	**56,42%**
5,00E-04	67,19%	61,04%	68,75%	71,32%	**67,08%**
1,00E-03	78,35%	71,61%	78,94%	83,86%	**78,19%**

yeast-growth/solubility 690nm/solvent control 690nm

cocentration [M	Replicate 1	Replicate 2	Replicate 3	Replicate 4	Mean
1,00E-07	8,12%	103,63%	78,07%	82,72%	**68,14%**
1,00E-06	83,56%	78,51%	75,13%	83,22%	**80,10%**
1,00E-05	68,65%	65,16%	68,56%	71,93%	**68,58%**
5,00E-05	71,67%	73,11%	72,04%	73,99%	**72,70%**
1,00E-04	73,09%	71,52%	69,36%	78,51%	**73,12%**
5,00E-04	84,98%	83,12%	84,92%	101,66%	**88,67%**
1,00E-03	103,28%	108,64%	99,87%	115,82%	**106,90%**

4-HT (540nm-690nm) anti-estrogenic control

cocentration [M	Replicate 1	Replicate 2	Replicate 3	Replicate 4
1,00E-06	0,055700004	0,050500005	0,053200006	0,059800006

4-HT (540nm-690nm)/1 nM E2 anti-estrogenic control

cocentration [M	Replicate 1	Replicate 2	Replicate 3	Replicate 4	Mean
1,00E-06	11,91%	9,44%	11,53%	13,29%	**11,55%**

cell-free 690nm

cocentration [M	Replicate 1	Replicate 2	Replicate 3	Replicate 4
1,00E-07	0,0383	0,0385	0,0374	0,037599999
1,00E-06	0,037599999	0,037599999	0,037	0,037599999
1,00E-05	0,037999999	0,037799999	0,036899999	0,0374
5,00E-05	0,0427	0,040100001	0,041000001	0,043099999
1,00E-04	0,064199999	0,067299999	0,063100003	0,065700002
5,00E-04	0,270700008	0,238499999	0,242899999	0,229800001
1,00E-03	0,455300003	0,470299989	0,490999997	0,441700011

cell-free solvent control 690nm

cocentration [M	Replicate 1	Replicate 2	Replicate 3	Replicate 4
	0,0372	0,0374	0,0374	0,037999999

cell-free (substance/solvent control)

cocentration [M	Replicate 1	Replicate 2	Replicate 3	Replicate 4	Mean
1,00E-07	102,96%	102,94%	100,00%	98,95%	**101,21%**
1,00E-06	101,08%	100,53%	98,93%	98,95%	**99,87%**
1,00E-05	102,15%	101,07%	98,66%	98,42%	**100,08%**
5,00E-05	114,78%	107,22%	109,63%	113,42%	**111,26%**
1,00E-04	172,58%	179,95%	168,72%	172,89%	**173,53%**
5,00E-04	727,69%	637,70%	649,47%	604,74%	**654,90%**
1,00E-03	1223,92%	1257,49%	1312,83%	1162,37%	**1239,15%**

YES fenhexamid + E2

1st experiment

540nm

cocentration [M]	Replicate 1	Replicate 2	Replicate 3	Replicate 4
1,00E-07	0,964100003	1,010200024	0,917800009	0,957199991
1,00E-06	0,967800021	0,92869997	0,90259999	0,924600005
1,00E-05	0,769800007	0,725499988	0,743799984	0,761799991
5,00E-05	0,689999998	0,691699982	0,673399985	0,669499993
1,00E-04	0,674799979	0,684700012	0,716300011	0,708999991
5,00E-04	0,271899998	0,287200004	0,244800001	0,25999999
1,00E-03	0,2861	0,258899987	0,239199996	0,2755

690nm

cocentration [M]	Replicate 1	Replicate 2	Replicate 3	Replicate 4
1,00E-07	0,437900007	0,461100012	0,40110001	0,416999996
1,00E-06	0,469999999	0,420599997	0,412299991	0,425000012
1,00E-05	0,446799994	0,416500002	0,446399987	0,450500011
5,00E-05	0,465299994	0,466699988	0,455599993	0,444200009
1,00E-04	0,447400004	0,467299998	0,498800009	0,476799995
5,00E-04	0,167500004	0,178299993	0,149599999	0,160099998
1,00E-03	0,175300002	0,161699995	0,146200001	0,169499993

solvent control 690nm

	Replicate 1	Replicate 2	Replicate 3	Replicate 4
	0,464100003	0,486000001	0,473199993	0,437099993

540-690nm

cocentration [M]	Replicate 1	Replicate 2	Replicate 3	Replicate 4
1,00E-07	0,526199996	0,549099982	0,516700029	0,540199995
1,00E-06	0,497799993	0,50819999	0,4903	0,499599993
1,00E-05	0,322899997	0,308899999	0,297399998	0,311199993
5,00E-05	0,224700004	0,224999994	0,217800006	0,225199997
1,00E-04	0,227500007	0,217399999	0,217500001	0,232199997
5,00E-04	0,104400001	0,108900003	0,095100001	0,100000001
1,00E-03	0,110799998	0,097199999	0,093099996	0,105999999

solvent control 540nm-690nm

	Replicate 1	Replicate 2	Replicate 3	Replicate 4
	0,120700002	0,1228	0,121600002	0,116800003

(540nm-690nm)-solvent control (540nm-690nm)

cocentration [M]	Replicate 1	Replicate 2	Replicate 3	Replicate 4
1,00E-07	0,405499995	0,426299982	0,395100027	0,423399992
1,00E-06	0,377099991	0,38539999	0,368699998	0,38279999
1,00E-05	0,202199996	0,186099999	0,175799996	0,19439999
5,00E-05	0,104000002	0,102199994	0,096200004	0,108399995
1,00E-04	0,106800005	0,094599999	0,095899999	0,115399994
5,00E-04	-0,0163	-0,013899997	-0,026500002	-0,016800001
1,00E-03	-0,009900004	-0,025600001	-0,028500006	-0,010800004

(540nm-690nm)-solvent control E2 reference

cocentration [M]	Replicate 1	Replicate 2	Replicate 3	Replicate 4
1,00E-09	0,412499979	0,377599984	0,430899993	0,533399969

Normalization (substance/1 nM E2)

cocentration [M]	Replicate 1	Replicate 2	Replicate 3	Replicate 4	Mean
1,00E-07	98,30%	112,90%	91,69%	79,38%	**95,57%**
1,00E-06	91,42%	102,07%	85,57%	71,77%	**87,70%**
1,00E-05	49,02%	49,28%	40,80%	36,45%	**43,89%**
5,00E-05	25,21%	27,07%	22,33%	20,32%	**23,73%**
1,00E-04	25,89%	25,05%	22,26%	21,63%	**23,71%**
5,00E-04	-3,95%	-3,68%	-6,15%	-3,15%	**-4,23%**
1,00E-03	-2,40%	-6,78%	-6,61%	-2,02%	**-4,45%**

yeast-growth/solubility 690nm/solvent control 690nm

cocentration [M]	Replicate 1	Replicate 2	Replicate 3	Replicate 4	Mean
1,00E-07	94,35%	94,88%	84,76%	95,40%	**92,35%**
1,00E-06	101,27%	86,54%	87,13%	97,23%	**93,04%**
1,00E-05	96,27%	85,70%	94,34%	103,07%	**94,84%**
5,00E-05	100,26%	96,03%	96,28%	101,62%	**98,55%**
1,00E-04	36,09%	36,69%	31,61%	36,63%	**35,26%**
5,00E-04	96,40%	96,15%	105,41%	109,08%	**101,76%**
1,00E-03	37,77%	33,27%	30,90%	38,78%	**35,18%**

4-HT (540nm-690nm) anti-estrogenic control

cocentration [M]	Replicate 1	Replicate 2	Replicate 3	Replicate 4
1,00E-06	0,076499999	0,0682	0,077700004	0,071399994

4-HT (540nm-690nm)/1 nM E2 anti-estrogenic control

cocentration [M]	Replicate 1	Replicate 2	Replicate 3	Replicate 4	Mean
1,00E-06	18,55%	18,06%	18,03%	13,39%	**17,01%**

excluded

2nd experiment

540nm

cocentration [M]	Replicate 1	Replicate 2	Replicate 3	Replicate 4
1,00E-07	0,840900004	0,838999987	0,871399999	0,885500014
1,00E-06	0,812099993	0,822899997	0,82069999	0,845799983
1,00E-05	0,743300021	0,723200023	0,730499983	0,756900012
5,00E-05	0,675499976	0,702099979	0,666299999	0,648999989
1,00E-04	0,734200001	0,763599992	0,703700006	0,696500003
5,00E-04	0,194299996	0,214499995	0,220799997	0,203199998
1,00E-03	0,208700001	0,200800002	0,207800001	0,215700001

690nm

cocentration [M]	Replicate 1	Replicate 2	Replicate 3	Replicate 4
1,00E-07	0,407299995	0,397399992	0,42899999	0,428900003
1,00E-06	0,403800011	0,400200009	0,416399986	0,422699988
1,00E-05	0,425799996	0,410100013	0,425999999	0,451099992
5,00E-05	0,422899991	0,450100005	0,41870001	0,40959999
1,00E-04	0,464100003	0,508099973	0,434300005	0,439900011
5,00E-04	0,095399998	0,108199999	0,108499996	0,1017
1,00E-03	0,1096	0,099799998	0,106700003	0,112999998

solvent control 690nm

	Replicate 1	Replicate 2	Replicate 3	Replicate 4
	0,504700005	0,520900011	0,497700006	0,466100007

540-690nm

cocentration [M]	Replicate 1	Replicate 2	Replicate 3	Replicate 4
1,00E-07	0,433499992	0,441599995	0,442400008	0,45660001
1,00E-06	0,408199996	0,422699988	0,404300004	0,423000008
1,00E-05	0,317499995	0,31310001	0,304500014	0,305799991
5,00E-05	0,252600014	0,252000004	0,247600004	0,239399996
1,00E-04	0,270099998	0,255600005	0,269400001	0,256599993
5,00E-04	0,098899998	0,106399998	0,112300001	0,101499997
1,00E-03	0,099100001	0,101099998	0,101099998	0,102799997

solvent control 540nm-690nm

	Replicate 1	Replicate 2	Replicate 3	Replicate 4
	0,132100001	0,129600003	0,1303	0,1162

(540nm-690nm)-solvent control (540nm-690nm)

cocentration [M]	Replicate 1	Replicate 2	Replicate 3	Replicate 4
1,00E-07	0,301399991	0,311999992	0,312100008	0,34040001
1,00E-06	0,276099995	0,293099985	0,274000004	0,306800008
1,00E-05	0,185399994	0,183500007	0,174200013	0,189599991
5,00E-05	0,120500013	0,122400001	0,117300004	0,123199999
1,00E-04	0,137999997	0,126000002	0,1391	0,140399992
5,00E-04	-0,033200003	-0,023200005	-0,017999999	-0,014700003
1,00E-03	-0,033	-0,028500006	-0,029200003	-0,013400003

(540nm-690nm)-solvent control E2 reference

cocentration [M]	Replicate 1	Replicate 2	Replicate 3	Replicate 4
1,00E-09	0,318199992	0,327400014	0,32600002	0,377300024

Normalization (substance/1 nM E2)

cocentration [M]	Replicate 1	Replicate 2	Replicate 3	Replicate 4	Mean
1,00E-07	94,72%	95,30%	95,74%	90,22%	**93,99%**
1,00E-06	86,77%	89,52%	84,05%	81,31%	**85,41%**
1,00E-05	58,27%	56,05%	53,44%	50,25%	**54,50%**
5,00E-05	37,87%	37,39%	35,98%	32,65%	**35,97%**
1,00E-04	43,37%	38,49%	42,67%	37,21%	**40,43%**
5,00E-04	-10,43%	-7,09%	-5,52%	-3,90%	**-6,73%**
1,00E-03	-10,37%	-8,70%	-8,96%	-3,55%	**-7,90%**

yeast-growth/solubility 690nm/solvent control 690nm

cocentration [M]	Replicate 1	Replicate 2	Replicate 3	Replicate 4	Mean
1,00E-07	80,70%	76,29%	86,20%	92,02%	**83,80%**
1,00E-06	80,01%	76,83%	83,66%	90,69%	**82,80%**
1,00E-05	84,37%	78,73%	85,59%	96,78%	**86,37%**
5,00E-05	83,79%	86,41%	84,13%	87,88%	**85,55%**
1,00E-04	91,96%	97,54%	87,26%	94,38%	**92,78%**
5,00E-04	18,90%	20,77%	21,80%	21,82%	**20,82%**
1,00E-03	21,72%	19,16%	21,44%	24,24%	**21,64%**

4-HT (540nm-690nm) anti-estrogenic control

cocentration [M]	Replicate 1	Replicate 2	Replicate 3	Replicate 4
1,00E-06	0,059699997	0,05659999	0,058200002	0,071600005

4-HT (540nm-690nm)/1 nM E2 anti-estrogenic control

cocentration [M]	Replicate 1	Replicate 2	Replicate 3	Replicate 4	Mean
1,00E-06	18,76%	17,29%	17,85%	18,98%	**18,22%**

3rd experiment

540nm

cocentration [M	Replicate 1	Replicate 2	Replicate 3	Replicate 4
1,00E-07	1,184900045	1,212000012	1,177999973	1,165799975
1,00E-06	1,144400001	1,175199986	1,12469995	1,111999989
1,00E-05	0,966499984	0,937699974	0,941799998	1,060199976
5,00E-05	0,734300017	0,757799983	0,792200029	0,753000021
1,00E-04	0,730599999	0,683499992	0,745299995	0,729799986
5,00E-04	0,114600003	0,1153	0,108199999	0,317299992
1,00E-03	0,148399994	0,129600003	0,1237	0,137199998

690nm

cocentration [M	Replicate 1	Replicate 2	Replicate 3	Replicate 4
1,00E-07	0,584100008	0,616100013	0,605000019	0,579900026
1,00E-06	0,603900015	0,611800015	0,5898	0,574199975
1,00E-05	0,599900007	0,593400002	0,592400014	0,686800003
5,00E-05	0,512700021	0,531099975	0,564999998	0,52640003
1,00E-04	0,515399992	0,473800004	0,518299997	0,514299989
5,00E-04	0,049699999	0,051199999	0,048799999	0,0995
1,00E-03	0,075300001	0,052200001	0,0515	0,062899999

solvent control 690nm

	Replicate 1	Replicate 2	Replicate 3	Replicate 4
	0,626600027	0,609200001	0,589900017	0,593699992

540-690nm

cocentration [M	Replicate 1	Replicate 2	Replicate 3	Replicate 4
1,00E-07	0,600700021	0,595799983	0,573000014	0,586000025
1,00E-06	0,540600002	0,563399971	0,53490001	0,537800014
1,00E-05	0,366600007	0,344300002	0,349400014	0,373400003
5,00E-05	0,221699998	0,226699993	0,227200001	0,226600006
1,00E-04	0,215200007	0,209800005	0,226999998	0,215499997
5,00E-04	0,064999998	0,064099997	0,0594	0,217800006
1,00E-03	0,073100001	0,077399999	0,072099999	0,074299999

solvent control 540nm-690nm

	Replicate 1	Replicate 2	Replicate 3	Replicate 4
	0,116999999	0,117799997	0,117299996	0,112099998

(540nm-690nm)-solvent control (540nm-690nm)

cocentration [M	Replicate 1	Replicate 2	Replicate 3	Replicate 4
1,00E-07	0,600700021	0,595799983	0,573000014	0,586000025
1,00E-06	0,540600002	0,563399971	0,53490001	0,537800014
1,00E-05	0,366600007	0,344300002	0,349400014	0,373400003
5,00E-05	0,221699998	0,226699993	0,227200001	0,226600006
1,00E-04	0,215200007	0,209800005	0,226999998	0,215499997
5,00E-04	0,064999998	0,064099997	0,0594	0,217800006
1,00E-03	0,073100001	0,077399999	0,072099999	0,074299999

(540nm-690nm)-solvent control E2 reference

cocentration [M	Replicate 1	Replicate 2	Replicate 3	Replicate 4
1,00E-09	0,474199988	0,476600021	0,456499994	0,480800025

Normalization (substance/1 nM E2)

cocentration [M	Replicate 1	Replicate 2	Replicate 3	Replicate 4	Mean
1,00E-07	126,68%	125,01%	125,52%	121,88%	**124,77%**
1,00E-06	114,00%	118,21%	117,17%	111,86%	**115,31%**
1,00E-05	77,31%	72,24%	76,54%	77,66%	**75,94%**
5,00E-05	46,75%	47,57%	49,77%	47,13%	**47,80%**
1,00E-04	45,38%	44,02%	49,73%	44,82%	**45,99%**
5,00E-04	13,71%	13,45%	13,01%	45,30%	**21,37%**
1,00E-03	15,42%	16,24%	15,79%	15,45%	**15,73%**

yeast-growth/solubility 690nm/solvent control 690nm

cocentration [M	Replicate 1	Replicate 2	Replicate 3	Replicate 4	Mean
1,00E-07	93,22%	101,13%	102,56%	97,68%	**98,65%**
1,00E-06	96,38%	100,43%	99,98%	96,72%	**98,38%**
1,00E-05	95,74%	97,41%	100,42%	115,68%	**102,31%**
5,00E-05	81,82%	87,18%	95,78%	88,66%	**88,36%**
1,00E-04	82,25%	77,77%	87,86%	86,63%	**83,63%**
5,00E-04	7,93%	8,40%	8,27%	16,76%	**10,34%**
1,00E-03	12,02%	8,57%	8,73%	10,59%	**9,98%**

4-HT (540nm-690nm) anti-estrogenic control

cocentration [M	Replicate 1	Replicate 2	Replicate 3	Replicate 4
1,00E-06	0,194700003	0,194800004	0,1928	0,187800005

4-HT (540nm-690nm)/1 nM E2 anti-estrogenic control

cocentration [M	Replicate 1	Replicate 2	Replicate 3	Replicate 4	Mean
1,00E-06	41,06%	40,87%	42,23%	39,06%	**40,81%**

4th experiment

540nm

cocentration [M	Replicate 1	Replicate 2	Replicate 3	Replicate 4
1,00E-07	1,081799984	1,110900044	1,113999963	1,179399967
1,00E-06	1,102100015	1,08070004	1,083199978	1,165300012
1,00E-05	0,896300018	1,041700006	0,940800011	0,939300001
5,00E-05	0,717499971	0,816100001	0,779100001	0,78490001
1,00E-04	0,75819999	0,70539999	0,803799987	0,757200003
5,00E-04	0,098899998	0,093599997	0,0898	0,097199999
1,00E-03	0,096799999	0,105999999	0,101599999	0,094499998

690nm

cocentration [M	Replicate 1	Replicate 2	Replicate 3	Replicate 4
1,00E-07	0,54339999	0,546899974	0,558799982	0,586099982
1,00E-06	0,572000027	0,558499992	0,560299993	0,604200006
1,00E-05	0,547100008	0,648500025	0,565800011	0,573800027
5,00E-05	0,484800011	0,564300001	0,519699991	0,531599998
1,00E-04	0,521600008	0,474599987	0,576099992	0,521399975
5,00E-04	0,054499999	0,050700001	0,047600001	0,0495
1,00E-03	0,059300002	0,0616	0,062700003	0,056000002

solvent control 690nm

	Replicate 1	Replicate 2	Replicate 3	Replicate 4
	0,658900023	0,700800002	0,635399997	0,636300027

540-690nm

cocentration [M	Replicate 1	Replicate 2	Replicate 3	Replicate 4
1,00E-07	0,538500011	0,563899994	0,555199981	0,593299985
1,00E-06	0,530099988	0,522199985	0,522899985	0,56099999
1,00E-05	0,349099994	0,39320001	0,375	0,365500003
5,00E-05	0,232700005	0,251800001	0,25940001	0,253300011
1,00E-04	0,236599997	0,230800003	0,227699995	0,235799998
5,00E-04	0,044399999	0,042800002	0,042199999	0,047699999
1,00E-03	0,037500001	0,044500001	0,038899999	0,0385

solvent control 540nm-690nm

	Replicate 1	Replicate 2	Replicate 3	Replicate 4
	0,100299999	0,1043	0,098499998	0,097499996

(540nm-690nm)-solvent control (540nm-690nm)

cocentration [M	Replicate 1	Replicate 2	Replicate 3	Replicate 4
1,00E-07	0,429799989	0,417899989	0,424399987	0,463499993
1,00E-06	0,248799995	0,28890001	0,276500002	0,268000007
1,00E-05	0,132400006	0,147500001	0,160900012	0,155800015
5,00E-05	0,136299998	0,126500003	0,129199997	0,138300002
1,00E-04	-0,0559	-0,061499998	-0,056299999	-0,049799997
5,00E-04	-0,062799998	-0,059799999	-0,059599999	-0,058999997
1,00E-03	-0,100299999	-0,1043	-0,098499998	-0,097499996

(540nm-690nm)-solvent control E2 reference

cocentration [M	Replicate 1	Replicate 2	Replicate 3	Replicate 4
1,00E-09	0,51669997	0,391900003	0,3957	0,394299991

Normalization (substance/1 nM E2)

cocentration [M	Replicate 1	Replicate 2	Replicate 3	Replicate 4	Mean
1,00E-07	83,18%	106,63%	107,25%	117,55%	**103,65%**
1,00E-06	48,15%	73,72%	69,88%	67,97%	**64,93%**
1,00E-05	25,62%	37,64%	40,66%	39,51%	**35,86%**
5,00E-05	26,38%	32,28%	32,65%	35,07%	**31,60%**
1,00E-04	-10,82%	-15,69%	-14,23%	-12,63%	**-13,34%**
5,00E-04	-12,15%	-15,26%	-15,06%	-14,96%	**-14,36%**
1,00E-03	-19,41%	-26,61%	-24,89%	-24,73%	**-23,91%**

yeast-growth/solubility 690nm/solvent control 690nm

cocentration [M	Replicate 1	Replicate 2	Replicate 3	Replicate 4	Mean
1,00E-07	82,47%	78,04%	87,94%	92,11%	**85,14%**
1,00E-06	86,81%	79,69%	88,18%	94,96%	**87,41%**
1,00E-05	83,03%	92,54%	89,05%	90,18%	**88,70%**
5,00E-05	73,58%	80,52%	81,79%	83,55%	**79,86%**
1,00E-04	79,16%	67,72%	90,67%	81,94%	**79,87%**
5,00E-04	8,27%	7,23%	7,49%	7,78%	**7,69%**
1,00E-03	9,00%	8,79%	9,87%	8,80%	**9,11%**

4-HT (540nm-690nm) anti-estrogenic control

cocentration [M	Replicate 1	Replicate 2	Replicate 3	Replicate 4	
1,00E-06	0,438200012	0,459599994	0,456699982	0,495799989	**pipetting error**

4-HT (540nm-690nm)/1 nM E2 anti-estrogenic control

cocentration [M	Replicate 1	Replicate 2	Replicate 3	Replicate 4	Mean
1,00E-06	84,81%	117,27%	115,42%	125,74%	**110,81%**

5th experiment

540nm

cocentration [M	Replicate 1	Replicate 2	Replicate 3	Replicate 4
1,00E-07	1,065400004	1,03550005	1,080500007	1,23029995
1,00E-06	1,04279995	1,037400007	1,063500047	0,978600025
1,00E-05	0,897300005	0,961799979	0,907999992	0,951200008
5,00E-05	0,771399975	0,76789999	0,760100007	0,919700027
1,00E-04	0,757200003	0,742999971	0,753700018	0,725199997
5,00E-04	0,098099999	0,094599999	0,092600003	0,091799997
1,00E-03	0,098200001	0,105999999	0,098899998	0,108499996

690nm

cocentration [M	Replicate 1	Replicate 2	Replicate 3	Replicate 4
1,00E-07	0,486000001	0,482199997	0,523699999	0,558000028
1,00E-06	0,493699998	0,514900029	0,549600005	0,530600011
1,00E-05	0,514400005	0,581499994	0,538699985	0,567399979
5,00E-05	0,492399991	0,502600014	0,490999997	0,629899979
1,00E-04	0,475499988	0,488000005	0,477600008	0,436800003
5,00E-04	0,049199998	0,0495	0,048099998	0,048300002
1,00E-03	0,0515	0,058800001	0,050999999	0,060199998

solvent control 690nm

	Replicate 1	Replicate 2	Replicate 3	Replicate 4
	0,605499983	0,5625	0,576799989	0,54460001

540-690nm

cocentration [M	Replicate 1	Replicate 2	Replicate 3	Replicate 4
1,00E-07	0,579400003	0,553300023	0,556800008	0,672299981
1,00E-06	0,549099982	0,522499979	0,513899982	0,448100001
1,00E-05	0,3829	0,380299985	0,369399995	0,3838
5,00E-05	0,279000014	0,265300006	0,269199997	0,289799988
1,00E-04	0,281800002	0,254999995	0,276199996	0,288500011
5,00E-04	0,048999999	0,045200001	0,044500001	0,043499999
1,00E-03	0,046700001	0,047200002	0,047899999	0,0484

solvent control 540nm-690nm

	Replicate 1	Replicate 2	Replicate 3	Replicate 4
	0,109899998	0,105099998	0,107699998	0,101400003

(540nm-690nm)-solvent control (540nm-690nm)

cocentration [M	Replicate 1	Replicate 2	Replicate 3	Replicate 4
1,00E-07	0,469500005	0,448200025	0,44910001	0,570899978
1,00E-06	0,439199984	0,41739998	0,406199984	0,346699998
1,00E-05	0,273000002	0,275199987	0,261699997	0,282399997
5,00E-05	0,169100016	0,160200007	0,161499999	0,188399985
1,00E-04	0,171900004	0,149899997	0,168499999	0,187100008
5,00E-04	-0,060899999	-0,059899997	-0,063199997	-0,057900004
1,00E-03	-0,063199997	-0,057899997	-0,059799999	-0,053000003

(540nm-690nm)-solvent control E2 reference

cocentration [M	Replicate 1	Replicate 2	Replicate 3	Replicate 4
1,00E-09	0,508200012	0,533000022	0,477800012	0,47639998

Normalization (substance/1 nM E2)

cocentration [M	Replicate 1	Replicate 2	Replicate 3	Replicate 4	Mean
1,00E-07	92,38%	84,09%	93,99%	119,84%	**97,58%**
1,00E-06	86,42%	78,31%	85,01%	72,77%	**80,63%**
1,00E-05	53,72%	51,63%	54,77%	59,28%	**54,85%**
5,00E-05	33,27%	30,06%	33,80%	39,55%	**34,17%**
1,00E-04	33,83%	28,12%	35,27%	39,27%	**34,12%**
5,00E-04	-11,98%	-11,24%	-13,23%	-12,15%	**-12,15%**
1,00E-03	-12,44%	-10,86%	-12,52%	-11,13%	**-11,73%**

yeast-growth/solubility 690nm/solvent control 690nm

cocentration [M	Replicate 1	Replicate 2	Replicate 3	Replicate 4	Mean
1,00E-07	80,26%	85,72%	90,79%	102,46%	**89,81%**
1,00E-06	81,54%	91,54%	95,28%	97,43%	**91,45%**
1,00E-05	84,95%	103,38%	93,39%	104,19%	**96,48%**
5,00E-05	81,32%	89,35%	85,12%	115,66%	**92,87%**
1,00E-04	78,53%	86,76%	82,80%	80,21%	**82,07%**
5,00E-04	8,13%	8,80%	8,34%	8,87%	**8,53%**
1,00E-03	8,51%	10,45%	8,84%	11,05%	**9,71%**

4-HT (540nm-690nm) anti-estrogenic control

	cocentration [M	Replicate 1	Replicate 2	Replicate 3	Replicate 4
!	1,00E-06	0,055000007	0,058100007	0,103199996	0,067100003

4-HT (540nm-690nm)/1 nM E2 anti-estrogenic control

cocentration [M	Replicate 1	Replicate 2	Replicate 3	Replicate 4	Mean
1,00E-06	10,82%	10,90%	21,60%	14,08%	**14,35%**

cell-free 690nm

cocentration [M	Replicate 1	Replicate 2	Replicate 3	Replicate 4
1,00E-07	0,037900001	0,038400002	0,037500001	0,037300002
1,00E-06	0,0372	0,0372	0,036699999	0,037300002
1,00E-05	0,037900001	0,037900001	0,036800001	0,037300002
5,00E-05	0,0374	0,038600001	0,0374	0,038199998
1,00E-04	0,0385	0,038400002	0,0396	0,038199998
5,00E-04	0,038800001	0,040800001	0,0381	0,038899999
1,00E-03	0,0506	0,051800001	0,050299998	0,0495

cell-free solvent control 690nm

cocentration [M	Replicate 1	Replicate 2	Replicate 3	Replicate 4
	0,039099999	0,040199999	0,0372	0,0381

cell-free (substance/solvent control)

cocentration [M	Replicate 1	Replicate 2	Replicate 3	Replicate 4	Mean
1,00E-07	96,93%	95,52%	100,81%	97,90%	**97,79%**
1,00E-06	95,14%	92,54%	98,66%	97,90%	**96,06%**
1,00E-05	96,93%	94,28%	98,92%	97,90%	**97,01%**
5,00E-05	95,65%	96,02%	100,54%	100,26%	**98,12%**
1,00E-04	98,47%	95,52%	106,45%	100,26%	**100,18%**
5,00E-04	99,23%	101,49%	102,42%	102,10%	**101,31%**
1,00E-03	129,41%	128,86%	135,22%	129,92%	**130,85%**

YES 4,4'-DDT + E2

1st experiment

540nm

cocentration [M]	Replicate 1	Replicate 2	Replicate 3	Replicate 4
1,00E-07	1,002500057	0,928900003	1,025400043	0,977100015
1,00E-06	1,02670002	1,005800009	1,020200014	0,923300028
5,00E-06	0,964600027	0,883099973	0,943199992	1,004999995
1,00E-05	0,901600003	0,897199988	0,895099998	0,960399985
5,00E-05	0,85650003	0,872099996	0,891499996	0,858699977
1,00E-04	0,860899985	0,867799997	0,821799994	0,839299977
5,00E-04	0,89200002	0,910399973	1,148300052	0,889699996

690nm

cocentration [M]	Replicate 1	Replicate 2	Replicate 3	Replicate 4
1,00E-07	0,460999995	0,422399998	0,48269999	0,45660001
1,00E-06	0,498800009	0,477299988	0,499000013	0,430900007
5,00E-06	0,46419999	0,399300009	0,466100007	0,498100013
1,00E-05	0,438699991	0,430400014	0,429500014	0,480599999
5,00E-05	0,399599999	0,423099995	0,435600013	0,41139999
1,00E-04	0,41139999	0,433999985	0,393000007	0,404599994
5,00E-04	0,412900001	0,461699992	0,602699995	0,453900009

solvent control 690nm

	Replicate 1	Replicate 2	Replicate 3	Replicate 4
	0,517099977	0,444999993	0,453500003	0,439900011

540-690nm

cocentration [M]	Replicate 1	Replicate 2	Replicate 3	Replicate 4
1,00E-07	0,541499972	0,506500006	0,542599976	0,520500004
1,00E-06	0,527899981	0,528500021	0,521099985	0,492300004
5,00E-06	0,500400007	0,483799994	0,477100015	0,506900012
1,00E-05	0,462900013	0,466800004	0,465600014	0,479799986
5,00E-05	0,456999987	0,449000001	0,455799997	0,447400004
1,00E-04	0,449499995	0,433899999	0,428799987	0,434700012
5,00E-04	0,479000002	0,448599994	0,545700014	0,435799986

solvent control 540nm-690nm

	Replicate 1	Replicate 2	Replicate 3	Replicate 4
	0,129800007	0,118600003	0,122100003	0,113399997

(540nm-690nm)-solvent control (540nm-690nm)

cocentration [M]	Replicate 1	Replicate 2	Replicate 3	Replicate 4
1,00E-07	0,411699966	0,387900002	0,420499973	0,407100007
1,00E-06	0,398099974	0,409900017	0,398999982	0,378900006
5,00E-06	0,3706	0,365199991	0,355000012	0,393500015
1,00E-05	0,333100006	0,348200001	0,343500011	0,366399989
5,00E-05	0,327199981	0,330399998	0,333699994	0,334000006
1,00E-04	0,319699988	0,315299995	0,306699984	0,321300015
5,00E-04	0,349199995	0,329999991	0,423600011	0,322399989

(540nm-690nm)-solvent control E2 reference

cocentration [M]	Replicate 1	Replicate 2	Replicate 3	Replicate 4
1,00E-09	0,47860001	0,408799991	0,405900031	0,414599992

Normalization (substance/1 nM E2)

cocentration [M]	Replicate 1	Replicate 2	Replicate 3	Replicate 4	Mean
1,00E-07	86,02%	94,89%	103,60%	98,19%	**95,67%**
1,00E-06	83,18%	100,27%	98,30%	91,39%	**93,28%**
5,00E-06	77,43%	89,33%	87,46%	94,91%	**87,28%**
1,00E-05	69,60%	85,18%	84,63%	88,37%	**81,94%**
5,00E-05	68,37%	80,82%	82,21%	80,56%	**77,99%**
1,00E-04	66,80%	77,13%	75,56%	77,50%	**74,25%**
5,00E-04	72,96%	80,72%	104,36%	77,76%	**83,95%**

yeast-growth/solubility 690nm/solvent control 690nm

cocentration [M]	Replicate 1	Replicate 2	Replicate 3	Replicate 4	Mean
1,00E-07	89,15%	94,92%	106,44%	103,80%	**98,58%**
1,00E-06	96,46%	107,26%	110,03%	97,95%	**102,93%**
5,00E-06	89,77%	89,73%	102,78%	113,23%	**98,88%**
1,00E-05	84,84%	96,72%	94,71%	109,25%	**96,38%**
5,00E-05	79,56%	97,53%	86,66%	91,98%	**88,93%**
1,00E-04	77,28%	95,08%	96,05%	93,52%	**90,48%**
5,00E-04	79,85%	103,75%	132,90%	103,18%	**104,92%**

4-HT (540nm-690nm) anti-estrogenic control

cocentration [M]	Replicate 1	Replicate 2	Replicate 3	Replicate 4
1,00E-06	0,060699999	0,066500001	0,060899995	0,075800002

4-HT (540nm-690nm)/1 nM E2 anti-estrogenic control

cocentration [M]	Replicate 1	Replicate 2	Replicate 3	Replicate 4	Mean
1,00E-06	12,68%	16,27%	15,00%	18,28%	**15,56%**

2nd experiment

540nm

cocentration [M]	Replicate 1	Replicate 2	Replicate 3	Replicate 4
1,00E-07	0,853900015	0,897899985	0,844500005	0,882399976
1,00E-06	0,90109998	0,905399978	0,839299977	0,832799971
5,00E-06	0,831799984	0,888599992	0,833500028	0,892499983
1,00E-05	0,842999995	0,835300028	0,834100008	0,805199981
5,00E-05	0,853399992	0,890600026	0,806900024	0,823800027
1,00E-04	0,847599983	0,841899991	0,732299984	0,810100019
5,00E-04	0,764100015	0,824299991	0,807900012	0,810899973

690nm

cocentration [M]	Replicate 1	Replicate 2	Replicate 3	Replicate 4
1,00E-07	0,407400012	0,433499992	0,393299997	0,431899995
1,00E-06	0,438699991	0,454299986	0,40110001	0,403800011
5,00E-06	0,409000009	0,435200006	0,412800014	0,461299986
1,00E-05	0,406100005	0,369300008	0,412	0,394600004
5,00E-05	0,407000005	0,4454	0,353199989	0,38530001
1,00E-04	0,39199999	0,411599994	0,278299987	0,373299986
5,00E-04	0,32249999	0,39230001	0,339300007	0,364899993

solvent control 690nm

	Replicate 1	Replicate 2	Replicate 3	Replicate 4
	0,497999996	0,451200008	0,460599989	0,479699999

540-690nm

cocentration [M]	Replicate 1	Replicate 2	Replicate 3	Replicate 4
1,00E-07	0,446500003	0,464399993	0,451200008	0,450500011
1,00E-06	0,462399989	0,451099992	0,43810001	0,42899999
5,00E-06	0,422800004	0,453399986	0,420700014	0,431300014
1,00E-05	0,43689999	0,465999991	0,421999991	0,410600007
5,00E-05	0,446399987	0,445199996	0,453700006	0,438499987
1,00E-04	0,455599993	0,430299997	0,453999996	0,436699986
5,00E-04	0,441599995	0,431899995	0,468499988	0,44600001

solvent control 540nm-690nm

	Replicate 1	Replicate 2	Replicate 3	Replicate 4
	0,130500004	0,1237	0,125400007	0,1237

(540nm-690nm)-solvent control (540nm-690nm)

cocentration [M]	Replicate 1	Replicate 2	Replicate 3	Replicate 4
1,00E-07	0,37819998	0,394700006	0,392499998	0,398800015
1,00E-06	0,376900002	0,381699994	0,374499992	0,387799978
5,00E-06	0,219600007	0,2271	0,237299994	0,250200003
1,00E-05	0,183599994	0,213399991	0,242099985	0,228000015
5,00E-05	0,225999996	0,232199982	0,268800005	0,272100002
1,00E-04	0,314299986	0,3697	0,355400011	0,466000021
5,00E-04	0,476100013	0,512799993	0,662700012	0,523000002

(540nm-690nm)-solvent control E2 reference

cocentration [M]	Replicate 1	Replicate 2	Replicate 3	Replicate 4
1,00E-09	0,394699976	0,422400028	0,38970001	0,397199973

Normalization (substance/1 nM E2)

cocentration [M]	Replicate 1	Replicate 2	Replicate 3	Replicate 4	Mean
1,00E-07	95,82%	93,44%	100,72%	100,40%	**97,60%**
1,00E-06	95,49%	90,36%	96,10%	97,63%	**94,90%**
5,00E-06	55,64%	53,76%	60,89%	62,99%	**58,32%**
1,00E-05	46,52%	50,52%	62,12%	57,40%	**54,14%**
5,00E-05	57,26%	54,97%	68,98%	68,50%	**62,43%**
1,00E-04	79,63%	87,52%	91,20%	117,32%	**93,92%**
5,00E-04	120,62%	121,40%	170,05%	131,67%	**135,94%**

yeast-growth/solubility 690nm/solvent control 690nm

cocentration [M]	Replicate 1	Replicate 2	Replicate 3	Replicate 4	Mean
1,00E-07	81,81%	96,08%	85,39%	90,04%	**88,33%**
1,00E-06	88,09%	100,69%	87,08%	84,18%	**90,01%**
5,00E-06	82,13%	96,45%	89,62%	96,16%	**91,09%**
1,00E-05	81,55%	81,85%	89,45%	82,26%	**83,78%**
5,00E-05	81,73%	98,71%	76,68%	80,32%	**84,36%**
1,00E-04	78,71%	91,22%	60,42%	77,82%	**77,04%**
5,00E-04	64,76%	86,95%	73,66%	76,07%	**75,36%**

4-HT (540nm-690nm) anti-estrogenic control

cocentration [M]	Replicate 1	Replicate 2	Replicate 3	Replicate 4
1,00E-06	0,056099996	0,061299995	0,0528	0,066699997

4-HT (540nm-690nm)/1 nM E2 anti-estrogenic control

cocentration [M]	Replicate 1	Replicate 2	Replicate 3	Replicate 4	Mean
1,00E-06	14,21%	14,51%	13,55%	16,79%	**14,77%**

3rd experiment

540nm

cocentration [M	Replicate 1	Replicate 2	Replicate 3	Replicate 4
1,00E-07	1,151900053	1,222000003	1,200700045	1,185299993
1,00E-06	1,093700051	1,174100041	1,153399944	1,19690001
5,00E-06	1,108899951	1,210199952	1,187000036	1,128299952
1,00E-05	1,140499949	1,189900041	1,428599954	1,150400043
5,00E-05	1,101199985	1,207900047	1,206599951	1,156399965
1,00E-04	1,233100057	1,228500009	1,126099944	1,179200053
5,00E-04	1,250200033	1,202900052	1,203400016	1,143300056

690nm

cocentration [M	Replicate 1	Replicate 2	Replicate 3	Replicate 4
1,00E-07	0,548600018	0,599799991	0,583599985	0,575299978
1,00E-06	0,518100023	0,577499986	0,555499971	0,566999972
5,00E-06	0,523800015	0,593299985	0,583400011	0,538200021
1,00E-05	0,559800029	0,577099979	0,719600022	0,569700003
5,00E-05	0,521799982	0,597800016	0,604200006	0,56400001
1,00E-04	0,625100017	0,604900002	0,531899989	0,583700001
5,00E-04	0,608699977	0,567499995	0,553200006	0,539399981

solvent control 690nm

	Replicate 1	Replicate 2	Replicate 3	Replicate 4
	0,619599998	0,640399992	0,619700015	0,606700003

540-690nm

cocentration [M	Replicate 1	Replicate 2	Replicate 3	Replicate 4
1,00E-07	0,603299975	0,622099996	0,6171	0,610000014
1,00E-06	0,575600028	0,596700013	0,59799999	0,629899979
5,00E-06	0,585200012	0,616900027	0,603600025	0,59009999
1,00E-05	0,58069998	0,612699986	0,708999991	0,58069998
5,00E-05	0,579400003	0,610099971	0,602400005	0,592400014
1,00E-04	0,60799998	0,623700023	0,594200015	0,595399976
5,00E-04	0,641499996	0,635399997	0,650099993	0,603900015

solvent control 540nm-690nm

	Replicate 1	Replicate 2	Replicate 3	Replicate 4
	0,115199998	0,117799997	0,115199998	0,115599997

(540nm-690nm)-solvent control (540nm-690nm)

cocentration [M	Replicate 1	Replicate 2	Replicate 3	Replicate 4
1,00E-07	0,488099977	0,504299998	0,501900002	0,494400017
1,00E-06	0,46040003	0,478900015	0,482799992	0,514299981
5,00E-06	0,470000014	0,499100029	0,488400027	0,474499993
1,00E-05	0,465499982	0,494899988	0,593799993	0,465099983
5,00E-05	0,464200005	0,492299974	0,487200007	0,476800017
1,00E-04	0,492799982	0,505900025	0,479000017	0,479799978
5,00E-04	0,526299998	0,5176	0,534899995	0,488300018

(540nm-690nm)-solvent control E2 reference

cocentration [M	Replicate 1	Replicate 2	Replicate 3	Replicate 4
1,00E-09	0,500000007	0,438200012	0,495200031	0,553300016

Normalization (substance/1 nM E2)

cocentration [M	Replicate 1	Replicate 2	Replicate 3	Replicate 4	Mean
1,00E-07	97,62%	115,08%	101,35%	89,35%	**100,85%**
1,00E-06	92,08%	109,29%	97,50%	92,95%	**97,95%**
5,00E-06	94,00%	113,90%	98,63%	85,76%	**98,07%**
1,00E-05	93,10%	112,94%	119,91%	84,06%	**102,50%**
5,00E-05	92,84%	112,35%	98,38%	86,17%	**97,44%**
1,00E-04	98,56%	115,45%	96,73%	86,72%	**99,36%**
5,00E-04	105,26%	118,12%	108,02%	88,25%	**104,91%**

yeast-growth/solubility 690nm/solvent control 690nm

cocentration [M	Replicate 1	Replicate 2	Replicate 3	Replicate 4	Mean
1,00E-07	88,54%	93,66%	94,17%	94,82%	**92,80%**
1,00E-06	83,62%	90,18%	89,64%	93,46%	**89,22%**
5,00E-06	84,54%	92,65%	94,14%	88,71%	**90,01%**
1,00E-05	90,35%	90,12%	116,12%	93,90%	**97,62%**
5,00E-05	84,22%	93,35%	97,50%	92,96%	**92,01%**
1,00E-04	100,89%	94,46%	85,83%	96,21%	**94,35%**
5,00E-04	98,24%	88,62%	89,27%	88,91%	**91,26%**

4-HT (540nm-690nm) anti-estrogenic control

cocentration [M	Replicate 1	Replicate 2	Replicate 3	Replicate 4
1,00E-06	0,087799996	0,087200001	0,110500008	0,08640001

4-HT (540nm-690nm)/1 nM E2 anti-estrogenic control

cocentration [M	Replicate 1	Replicate 2	Replicate 3	Replicate 4	Mean
1,00E-06	17,56%	19,90%	22,31%	15,62%	**18,85%**

4th experiment

540nm

cocentration [M	Replicate 1	Replicate 2	Replicate 3	Replicate 4
1,00E-07	1,091699958	1,10739994	1,17900002	1,300799966
1,00E-06	1,085399985	1,099699974	1,126000047	1,118100047
5,00E-06	1,084400058	1,148200035	1,069100022	1,137099981
1,00E-05	1,036000013	1,01789999	1,079399943	1,087499976
5,00E-05	0,993799984	1,100999951	1,04429996	1,127300024
1,00E-04	1,099400043	1,043200016	1,070500016	1,064900041
5,00E-04	1,198699951	1,231899977	1,191499949	1,248900056

690nm

cocentration [M	Replicate 1	Replicate 2	Replicate 3	Replicate 4
1,00E-07	0,548900008	0,557900012	0,571099997	0,628799975
1,00E-06	0,533999979	0,55309999	0,553900003	0,566999972
5,00E-06	0,557299972	0,574400008	0,538699985	0,58950001
1,00E-05	0,538200021	0,512000024	0,540499985	0,558200002
5,00E-05	0,502399981	0,578299999	0,529000014	0,591799974
1,00E-04	0,576200008	0,528900027	0,555800021	0,540600002
5,00E-04	0,633000016	0,642700016	0,635299981	0,673200011

solvent control 690nm

	Replicate 1	Replicate 2	Replicate 3	Replicate 4
	0,718900025	0,682500005	0,639199972	0,624499977

540-690nm

cocentration [M	Replicate 1	Replicate 2	Replicate 3	Replicate 4
1,00E-07	0,542900026	0,549499989	0,60799998	0,671999991
1,00E-06	0,551400006	0,546599984	0,572099984	0,551100016
5,00E-06	0,527100027	0,573800027	0,530300021	0,547599971
1,00E-05	0,497799993	0,505800009	0,538900018	0,529299974
5,00E-05	0,491400003	0,522700012	0,514299989	0,53549999
1,00E-04	0,523100019	0,514299989	0,514699996	0,524299979
5,00E-04	0,565699995	0,58920002	0,556100011	0,575699985

solvent control 540nm-690nm

	Replicate 1	Replicate 2	Replicate 3	Replicate 4
	0,103699997	0,102399997	0,098099999	0,098200001

(540nm-690nm)-solvent control (540nm-690nm)

cocentration [M	Replicate 1	Replicate 2	Replicate 3	Replicate 4
1,00E-07	0,439200029	0,447099991	0,509899981	0,57379999
1,00E-06	0,447700009	0,444199987	0,473999985	0,452900015
5,00E-06	0,42340003	0,47140003	0,432200022	0,44939997
1,00E-05	0,394099995	0,403400011	0,440800019	0,431099974
5,00E-05	0,387700006	0,420300014	0,41619999	0,437299989
1,00E-04	0,419400021	0,411899991	0,416599996	0,426099978
5,00E-04	0,461999997	0,486800022	0,458000012	0,477499984

(540nm-690nm)-solvent control E2 reference

cocentration [M	Replicate 1	Replicate 2	Replicate 3	Replicate 4
1,00E-09	0,509299986	0,480200022	0,531100027	0,515799977

Normalization (substance/1 nM E2)

cocentration [M	Replicate 1	Replicate 2	Replicate 3	Replicate 4	Mean
1,00E-07	86,24%	93,11%	96,01%	111,24%	**96,65%**
1,00E-06	87,90%	92,50%	89,25%	87,81%	**89,37%**
5,00E-06	83,13%	98,17%	81,38%	87,13%	**87,45%**
1,00E-05	77,38%	84,01%	83,00%	83,58%	**81,99%**
5,00E-05	76,12%	87,53%	78,37%	84,78%	**81,70%**
1,00E-04	82,35%	85,78%	78,44%	82,61%	**82,29%**
5,00E-04	90,71%	101,37%	86,24%	92,57%	**92,72%**

yeast-growth/solubility 690nm/solvent control 690nm

cocentration [M	Replicate 1	Replicate 2	Replicate 3	Replicate 4	Mean
1,00E-07	76,35%	81,74%	89,35%	100,69%	**87,03%**
1,00E-06	74,28%	81,04%	86,66%	90,79%	**83,19%**
5,00E-06	77,52%	84,16%	84,28%	94,40%	**85,09%**
1,00E-05	74,86%	75,02%	84,56%	89,38%	**80,96%**
5,00E-05	69,88%	84,73%	82,90%	94,76%	**83,07%**
1,00E-04	80,15%	77,49%	86,95%	86,57%	**82,79%**
5,00E-04	88,05%	94,17%	99,39%	107,80%	**97,35%**

4-HT (540nm-690nm) anti-estrogenic control

cocentration [M	Replicate 1	Replicate 2	Replicate 3	Replicate 4
1,00E-06	0,0625	0,077100001	0,065800004	0,077

4-HT (540nm-690nm)/1 nM E2 anti-estrogenic control

cocentration [M	Replicate 1	Replicate 2	Replicate 3	Replicate 4	Mean
1,00E-06	12,27%	16,06%	12,39%	14,93%	**13,91%**

5th experiment

540nm

cocentration [M	Replicate 1	Replicate 2	Replicate 3	Replicate 4
1,00E-07	1,072199941	1,132099986	1,178900003	1,10619998
1,00E-06	1,010900021	1,087499976	1,081200004	1,053599954
5,00E-06	1,036399961	1,100700021	1,039000034	1,074800014
1,00E-05	1,024000049	1,094599962	1,04550004	1,005599976
5,00E-05	1,049600005	1,092399955	1,092900038	1,034000039
1,00E-04	1,031499982	1,056100011	0,969799995	1,030500054
5,00E-04	1,063699961	1,155699968	1,055699944	1,038300037

690nm

cocentration [M	Replicate 1	Replicate 2	Replicate 3	Replicate 4
1,00E-07	0,487100005	0,540199995	0,562300026	0,512799978
1,00E-06	0,461299986	0,516300023	0,517099977	0,498800009
5,00E-06	0,483200014	0,531700015	0,495400012	0,515200019
1,00E-05	0,47299999	0,54400003	0,513599992	0,467200011
5,00E-05	0,530200005	0,540300012	0,543900013	0,493200004
1,00E-04	0,483799994	0,493900001	0,4111	0,472499996
5,00E-04	0,4542	0,523299992	0,459300011	0,431400001

solvent control 690nm

	Replicate 1	Replicate 2	Replicate 3	Replicate 4
	0,703299999	0,577499986	0,546700001	0,566299975

540-690nm

cocentration [M	Replicate 1	Replicate 2	Replicate 3	Replicate 4
1,00E-07	0,585200012	0,591799974	0,616599977	0,593400002
1,00E-06	0,549600005	0,571200013	0,564100027	0,554799974
5,00E-06	0,553200006	0,569000006	0,543600023	0,559599996
1,00E-05	0,550999999	0,550599992	0,531899989	0,538500011
5,00E-05	0,519400001	0,552100003	0,549000025	0,540899992
1,00E-04	0,547699988	0,56220001	0,558700025	0,558000028
5,00E-04	0,609499991	0,632399976	0,596400023	0,606999993

solvent control 540nm-690nm

	Replicate 1	Replicate 2	Replicate 3	Replicate 4
	0,1171	0,1131	0,114200003	0,111000001

(540nm-690nm)-solvent control (540nm-690nm)

cocentration [M	Replicate 1	Replicate 2	Replicate 3	Replicate 4
1,00E-07	0,468100011	0,478699975	0,502399974	0,4824
1,00E-06	0,432500005	0,458100013	0,449900024	0,443799973
5,00E-06	0,436100006	0,455900006	0,429400019	0,448599994
1,00E-05	0,433899999	0,437499993	0,417699985	0,42750001
5,00E-05	0,4023	0,439000003	0,434800021	0,429899991
1,00E-04	0,430599988	0,44910001	0,444500022	0,447000027
5,00E-04	0,492399991	0,519299977	0,482200019	0,495999992

(540nm-690nm)-solvent control E2 reference

cocentration [M	Replicate 1	Replicate 2	Replicate 3	Replicate 4
1,00E-09	0,44319997	0,4608	0,457400031	0,45190002

Normalization (substance/1 nM E2)

cocentration [M	Replicate 1	Replicate 2	Replicate 3	Replicate 4	Mean
1,00E-07	105,62%	103,88%	109,84%	106,75%	**106,52%**
1,00E-06	97,59%	99,41%	98,36%	98,21%	**98,39%**
5,00E-06	98,40%	98,94%	93,88%	99,27%	**97,62%**
1,00E-05	97,90%	94,94%	91,32%	94,60%	**94,69%**
5,00E-05	90,77%	95,27%	95,06%	95,13%	**94,06%**
1,00E-04	97,16%	97,46%	97,18%	98,92%	**97,68%**
5,00E-04	111,10%	112,70%	105,42%	109,76%	**109,74%**

yeast-growth/solubility 690nm/solvent control 690nm

cocentration [M	Replicate 1	Replicate 2	Replicate 3	Replicate 4	Mean
1,00E-07	69,26%	93,54%	102,85%	90,55%	**89,05%**
1,00E-06	65,59%	89,40%	94,59%	88,08%	**84,41%**
5,00E-06	68,70%	92,07%	90,62%	90,98%	**85,59%**
1,00E-05	67,25%	94,20%	93,95%	82,50%	**84,47%**
5,00E-05	75,39%	93,56%	99,49%	87,09%	**88,88%**
1,00E-04	68,79%	85,52%	75,20%	83,44%	**78,24%**
5,00E-04	64,58%	90,61%	84,01%	76,18%	**78,85%**

4-HT (540nm-690nm) anti-estrogenic control

cocentration [M	Replicate 1	Replicate 2	Replicate 3	Replicate 4
1,00E-06	0,045499995	0,052500002	0,049299993	0,057300001

4-HT (540nm-690nm)/1 nM E2 anti-estrogenic control

cocentration [M	Replicate 1	Replicate 2	Replicate 3	Replicate 4	Mean
1,00E-06	10,27%	11,39%	10,78%	12,68%	**11,28%**

cell-free 690nm

cocentration [M	Replicate 1	Replicate 2	Replicate 3	Replicate 4
1,00E-07	0,037999999	0,038699999	0,037300002	0,0372
1,00E-06	0,0374	0,0381	0,036899999	0,037099998
5,00E-06	0,037999999	0,038400002	0,037099998	0,037599999
1,00E-05	0,038600001	0,038199998	0,037999999	0,037999999
5,00E-05	0,0427	0,042399999	0,041200001	0,044
1,00E-04	0,059799999	0,060699999	0,056200001	0,0579
5,00E-04	0,199000001	0,148599997	0,190799996	0,211999997

cell-free solvent control 690nm

cocentration [M	Replicate 1	Replicate 2	Replicate 3	Replicate 4
	0,037099998	0,037099998	0,037500001	0,039299998

cell-free (substance/solvent control)

cocentration [M	Replicate 1	Replicate 2	Replicate 3	Replicate 4	Mean
1,00E-07	102,43%	104,31%	99,47%	94,66%	**100,22%**
1,00E-06	100,81%	102,70%	98,40%	94,40%	**99,08%**
5,00E-06	102,43%	103,50%	98,93%	95,67%	**100,13%**
1,00E-05	104,04%	102,96%	101,33%	96,69%	**101,26%**
5,00E-05	115,09%	114,29%	109,87%	111,96%	**112,80%**
1,00E-04	161,19%	163,61%	149,87%	147,33%	**155,50%**
5,00E-04	536,39%	400,54%	508,80%	539,44%	**496,29%**

YES 2,4'-DDT + E2

1st experiment

540nm

cocentration [M]	Replicate 1	Replicate 2	Replicate 3	Replicate 4
1,00E-07	1,001799941	0,99089998	0,991100013	1,022099972
1,00E-06	0,907599986	0,977900028	0,98360002	1,004899979
5,00E-06	0,885200024	0,931999981	0,963699996	0,907199979
1,00E-05	0,922800004	0,893400013	0,991400003	0,966000021
5,00E-05	0,989799976	0,936999977	0,999899983	1,065000057
1,00E-04	0,930199981	1,006700039	1,397699952	1,120700002
5,00E-04	1,121399999	1,204399943	1,21329999	1,279999971

690nm

cocentration [M]	Replicate 1	Replicate 2	Replicate 3	Replicate 4
1,00E-07	0,503700018	0,475800008	0,466899991	0,475199997
1,00E-06	0,421900004	0,463999987	0,47299999	0,490399987
5,00E-06	0,399899989	0,439399987	0,456900001	0,41929999
1,00E-05	0,414700001	0,402700007	0,461400002	0,42809999
5,00E-05	0,430000007	0,397700012	0,441700011	0,451099992
1,00E-04	0,387100011	0,417100012	0,431800008	0,415899992
5,00E-04	0,406800002	0,446399987	0,399599999	0,413599998

solvent control 690nm

	Replicate 1	Replicate 2	Replicate 3	Replicate 4
	0,528299987	0,526600003	0,562300026	0,527800024

540-690nm

cocentration [M]	Replicate 1	Replicate 2	Replicate 3	Replicate 4
1,00E-07	0,498100013	0,515100002	0,524100006	0,546999991
1,00E-06	0,485700011	0,513899982	0,510699987	0,514500022
5,00E-06	0,485300004	0,492599994	0,506799996	0,487899989
1,00E-05	0,50819999	0,490799993	0,529900014	0,537899971
5,00E-05	0,559800029	0,539300025	0,558200002	0,613900006
1,00E-04	0,543099999	0,58950001	0,965900004	0,704699993
5,00E-04	0,714699984	0,758000016	0,81370002	0,866400003

solvent control 540nm-690nm

	Replicate 1	Replicate 2	Replicate 3	Replicate 4
	0,131200001	0,1294	0,132799998	0,126900002

(540nm-690nm)-solvent control (540nm-690nm)

cocentration [M]	Replicate 1	Replicate 2	Replicate 3	Replicate 4
1,00E-07	0,36690001	0,3857	0,39130001	0,42009999
1,00E-06	0,354500011	0,384499982	0,377899989	0,38760002
5,00E-06	0,354100004	0,363199994	0,373999998	0,360999987
1,00E-05	0,376999989	0,361399993	0,397100016	0,410999969
5,00E-05	0,428600028	0,409900025	0,425400004	0,487000003
1,00E-04	0,411899999	0,46010001	0,833100006	0,577799991
5,00E-04	0,583499983	0,628600016	0,680900022	0,739500001

(540nm-690nm)-solvent control E2 reference

cocentration [M]	Replicate 1	Replicate 2	Replicate 3	Replicate 4
1,00E-09	0,415099978	0,391199991	0,428800032	0,407999977

Normalization (substance/1 nM E2)

cocentration [M]	Replicate 1	Replicate 2	Replicate 3	Replicate 4	Mean
1,00E-07	31,61%	33,08%	30,97%	31,10%	**31,69%**
1,00E-06	85,40%	98,29%	88,13%	95,00%	**91,70%**
5,00E-06	85,30%	92,84%	87,22%	88,48%	**88,46%**
1,00E-05	90,82%	92,38%	92,61%	100,74%	**94,14%**
5,00E-05	103,25%	104,78%	99,21%	119,36%	**106,65%**
1,00E-04	99,23%	117,61%	194,29%	141,62%	**138,19%**
5,00E-04	140,57%	160,69%	158,79%	181,25%	**160,32%**

yeast-growth/solubility 690nm/solvent control 690nm

cocentration [M]	Replicate 1	Replicate 2	Replicate 3	Replicate 4	Mean
1,00E-07	95,34%	90,35%	83,03%	90,03%	**89,69%**
1,00E-06	79,86%	88,11%	84,12%	92,91%	**86,25%**
5,00E-06	75,70%	83,44%	81,26%	79,44%	**79,96%**
1,00E-05	78,50%	76,47%	82,06%	81,11%	**79,53%**
5,00E-05	73,27%	79,21%	76,79%	78,80%	**77,02%**
1,00E-04	81,39%	75,52%	78,55%	85,47%	**80,23%**
5,00E-04	77,00%	84,77%	71,07%	78,36%	**77,80%**

4-HT (540nm-690nm) anti-estrogenic control

cocentration [M]	Replicate 1	Replicate 2	Replicate 3	Replicate 4
1,00E-06	0,060000002	0,049700007	0,056500003	0,070600003

4-HT (540nm-690nm)/1 nM E2 anti-estrogenic control

cocentration [M]	Replicate 1	Replicate 2	Replicate 3	Replicate 4	Mean
1,00E-06	14,45%	12,70%	13,18%	17,30%	**14,41%**

2nd experient

540nm

cocentration [M]	Replicate 1	Replicate 2	Replicate 3	Replicate 4
1,00E-07	0,927299976	0,898699999	0,932600021	0,935299993
1,00E-06	0,934400022	0,936800003	0,988600016	0,925700009
5,00E-06	0,927900016	0,916199982	0,935800016	0,9023
1,00E-05	0,941399992	0,893299997	0,920700014	0,907400012
5,00E-05	0,931100011	0,968699992	0,945299983	0,845600009
1,00E-04	0,919200003	0,905900002	0,89230001	1,063500047
5,00E-04	0,944599986	0,941600025	0,904100001	0,921500027

690nm

cocentration [M]	Replicate 1	Replicate 2	Replicate 3	Replicate 4
1,00E-07	0,447600007	0,444900006	0,468800008	0,467700005
1,00E-06	0,452600002	0,472799987	0,512199998	0,472900003
5,00E-06	0,460599989	0,448799998	0,467099994	0,441300005
1,00E-05	0,487599999	0,4366	0,451799989	0,452399999
5,00E-05	0,463200003	0,468699992	0,466800004	0,390799999
1,00E-04	0,424400002	0,419999987	0,4111	0,520799994
5,00E-04	0,448599994	0,449999988	0,417199999	0,42019999

solvent control 690nm

	Replicate 1	Replicate 2	Replicate 3	Replicate 4
	0,566299975	0,580500007	0,508599997	0,560199976

540-690nm

cocentration [M]	Replicate 1	Replicate 2	Replicate 3	Replicate 4
1,00E-07	0,479699999	0,453700006	0,463800013	0,467599988
1,00E-06	0,481900007	0,463999987	0,476399988	0,452800006
5,00E-06	0,467400014	0,467400014	0,468699992	0,460999995
1,00E-05	0,453900009	0,45660001	0,468899995	0,455000013
5,00E-05	0,467999995	0,5	0,478599995	0,454699993
1,00E-04	0,494800001	0,485900015	0,48120001	0,542800009
5,00E-04	0,495999992	0,491600007	0,486900002	0,501299977

solvent control 540nm-690nm

	Replicate 1	Replicate 2	Replicate 3	Replicate 4
	0,139599994	0,137799993	0,130799994	0,129600003

(540nm-690nm)-solvent control (540nm-690nm)

cocentration [M]	Replicate 1	Replicate 2	Replicate 3	Replicate 4
1,00E-07	0,340100005	0,315900013	0,333000019	0,337999985
1,00E-06	0,342300013	0,326199993	0,345599994	0,323200002
5,00E-06	0,327800021	0,329600021	0,337899998	0,331399992
1,00E-05	0,314300016	0,318800017	0,338100001	0,32540001
5,00E-05	0,328400001	0,362200007	0,347800002	0,32509999
1,00E-04	0,355200008	0,348100021	0,350400016	0,413200006
5,00E-04	0,356399998	0,353800014	0,356100008	0,371699974

(540nm-690nm)-solvent control E2 reference

cocentration [M]	Replicate 1	Replicate 2	Replicate 3	Replicate 4
1,00E-09	0,284299985	0,386300012	0,336300001	0,354599983

Normalization (substance/1 nM E2)

cocentration [M]	Replicate 1	Replicate 2	Replicate 3	Replicate 4	Mean
1,00E-07	119,63%	81,78%	99,02%	95,32%	**98,94%**
1,00E-06	120,40%	84,44%	102,77%	91,14%	**99,69%**
5,00E-06	115,30%	85,32%	100,48%	93,46%	**98,64%**
1,00E-05	110,55%	82,53%	100,54%	91,77%	**96,34%**
5,00E-05	115,51%	93,76%	103,42%	91,68%	**101,09%**
1,00E-04	124,94%	90,11%	104,19%	116,53%	**108,94%**
5,00E-04	125,36%	91,59%	105,89%	104,82%	**106,91%**

yeast-growth/solubility 690nm/solvent control 690nm

cocentration [M]	Replicate 1	Replicate 2	Replicate 3	Replicate 4	Mean
1,00E-07	79,04%	76,64%	92,17%	83,49%	**82,84%**
1,00E-06	79,92%	81,45%	100,71%	84,42%	**86,62%**
5,00E-06	81,33%	77,31%	91,84%	78,78%	**82,32%**
1,00E-05	86,10%	75,21%	88,83%	80,76%	**82,73%**
5,00E-05	81,79%	80,74%	91,78%	69,76%	**81,02%**
1,00E-04	74,94%	72,35%	80,83%	92,97%	**80,27%**
5,00E-04	79,22%	77,52%	82,03%	75,01%	**78,44%**

4-HT (540nm-690nm) anti-estrogenic control

cocentration [M]	Replicate 1	Replicate 2	Replicate 3	Replicate 4
1,00E-06	0,048500001	0,046800002	0,05430001	0,0546

4-HT (540nm-690nm)/1 nM E2 anti-estrogenic control

cocentration [M]	Replicate 1	Replicate 2	Replicate 3	Replicate 4	Mean
1,00E-06	17,06%	12,11%	16,15%	15,40%	**15,18%**

3rd experiment

540nm

cocentration [M	Replicate 1	Replicate 2	Replicate 3	Replicate 4
1,00E-07	1,302800059	1,215800047	1,235800028	1,287199974
1,00E-06	1,156999946	1,251899958	1,186499953	1,173900008
5,00E-06	1,152899981	1,153699994	1,131100059	1,157699943
1,00E-05	1,120100021	1,116700053	1,100999951	1,166499972
5,00E-05	1,192000031	1,181900024	1,163100004	1,181800008
1,00E-04	1,203699946	1,171000004	1,190500021	1,128399968
5,00E-04	1,526800036	1,32190001	1,716699958	1,271000028

690nm

cocentration [M	Replicate 1	Replicate 2	Replicate 3	Replicate 4
1,00E-07	0,624300003	0,581900001	0,600000024	0,617699981
1,00E-06	0,561200023	0,611100018	0,578400016	0,573499978
5,00E-06	0,579900026	0,567099988	0,557900012	0,557099998
1,00E-05	0,540400028	0,549600005	0,535099983	0,584000022
5,00E-05	0,574599981	0,568700016	0,566799998	0,573800027
1,00E-04	0,595499992	0,565699995	0,581900001	0,509899974
5,00E-04	0,690199971	0,610099971	0,701799989	0,566100001

solvent control 690nm

	Replicate 1	Replicate 2	Replicate 3	Replicate 4
	0,684300005	0,649200022	0,652400017	0,613499999

540-690nm

cocentration [M	Replicate 1	Replicate 2	Replicate 3	Replicate 4
1,00E-07	0,67839998	0,633899987	0,635900021	0,669499993
1,00E-06	0,595799983	0,640799999	0,608099997	0,600499988
5,00E-06	0,573000014	0,586600006	0,573199987	0,600600004
1,00E-05	0,579699993	0,567099988	0,565900028	0,58160001
5,00E-05	0,617299974	0,613300025	0,596300006	0,608099997
1,00E-04	0,608200014	0,605300009	0,608500004	0,618499994
5,00E-04	0,836600006	0,711700022	1,014999986	0,704900026

solvent control 540nm-690nm

	Replicate 1	Replicate 2	Replicate 3	Replicate 4
	0,1219	0,119599998	0,118100002	0,115999997

(540nm-690nm)-solvent control (540nm-690nm)

cocentration [M	Replicate 1	Replicate 2	Replicate 3	Replicate 4
1,00E-07	0,55649998	0,514299989	0,517800018	0,553499997
1,00E-06	0,473899983	0,521200001	0,489999995	0,484499991
5,00E-06	0,451100014	0,467000008	0,455099985	0,484600008
1,00E-05	0,457799993	0,44749999	0,447800025	0,465600014
5,00E-05	0,495399974	0,493700027	0,478200004	0,4921
1,00E-04	0,486300014	0,485700011	0,490400001	0,502499998
5,00E-04	0,714700006	0,592100024	0,896899983	0,58890003

(540nm-690nm)-solvent control E2 reference

cocentration [M	Replicate 1	Replicate 2	Replicate 3	Replicate 4
1,00E-09	0,527300008	0,5211	0,49220001	0,550800018

Normalization (substance/1 nM E2)

cocentration [M	Replicate 1	Replicate 2	Replicate 3	Replicate 4	Mean
1,00E-07	105,54%	98,70%	105,20%	100,49%	**102,48%**
1,00E-06	89,87%	100,02%	99,55%	87,96%	**94,35%**
5,00E-06	85,55%	89,62%	92,46%	87,98%	**88,90%**
1,00E-05	86,82%	85,88%	90,98%	84,53%	**87,05%**
5,00E-05	93,95%	94,74%	97,16%	89,34%	**93,80%**
1,00E-04	92,22%	93,21%	99,63%	91,23%	**94,07%**
5,00E-04	135,54%	113,63%	182,22%	106,92%	**134,58%**

yeast-growth/solubility 690nm/solvent control 690nm

cocentration [M	Replicate 1	Replicate 2	Replicate 3	Replicate 4	Mean
1,00E-07	91,23%	89,63%	91,97%	100,68%	**93,38%**
1,00E-06	82,01%	94,13%	88,66%	93,48%	**89,57%**
5,00E-06	84,74%	87,35%	85,52%	90,81%	**87,10%**
1,00E-05	78,97%	84,66%	82,02%	95,34%	**85,25%**
5,00E-05	83,97%	87,60%	86,88%	93,53%	**87,99%**
1,00E-04	87,02%	87,14%	89,19%	83,11%	**86,62%**
5,00E-04	100,86%	93,98%	107,57%	92,27%	**98,67%**

4-HT (540nm-690nm) anti-estrogenic control

cocentration [M	Replicate 1	Replicate 2	Replicate 3	Replicate 4
1,00E-06	0,083300002	0,085700005	0,079699993	0,086900011

4-HT (540nm-690nm)/1 nM E2 anti-estrogenic control

cocentration [M	Replicate 1	Replicate 2	Replicate 3	Replicate 4	Mean
1,00E-06	15,80%	16,45%	16,19%	15,78%	**16,05%**

4th experiment

540nm

cocentration [M	Replicate 1	Replicate 2	Replicate 3	Replicate 4
1,00E-07	1,057700038	1,078600049	0,995800018	1,002699971
1,00E-06	1,044800043	0,965900004	0,993399978	1,021199942
5,00E-06	1,006199956	0,956900001	1,023000002	1,0273
1,00E-05	0,96359998	1,032999992	0,995999992	1,111899972
5,00E-05	1,011500001	1,049100041	1,02670002	1,074100018
1,00E-04	1,077100039	0,994700015	1,087200046	1,087700009
5,00E-04	1,210899949	1,189499974	1,165899992	1,199599981

690nm

cocentration [M	Replicate 1	Replicate 2	Replicate 3	Replicate 4
1,00E-07	0,548500001	0,542699993	0,493999988	0,486600012
1,00E-06	0,533200026	0,445800006	0,507700026	0,508899987
5,00E-06	0,524900019	0,47389999	0,534500003	0,517400026
1,00E-05	0,508099973	0,549399972	0,516300023	0,568899989
5,00E-05	0,535600007	0,537	0,529999971	0,539099991
1,00E-04	0,555100024	0,507200003	0,570900023	0,542999983
5,00E-04	0,621800005	0,61650002	0,583400011	0,622099996

solvent control 690nm

	Replicate 1	Replicate 2	Replicate 3	Replicate 4
	0,690199971	0,596099973	0,649100006	0,625699997

540-690nm

cocentration [M	Replicate 1	Replicate 2	Replicate 3	Replicate 4
1,00E-07	0,509299994	0,535899997	0,501800001	0,516099989
1,00E-06	0,511600018	0,520099998	0,485700011	0,512300014
5,00E-06	0,481299996	0,48300001	0,488499999	0,50999999
1,00E-05	0,455500007	0,483599991	0,479799986	0,542999983
5,00E-05	0,475899994	0,512099981	0,496699989	0,535000026
1,00E-04	0,521899998	0,487500012	0,516300023	0,544799984
5,00E-04	0,58920002	0,572899997	0,582499981	0,577499986

solvent control 540nm-690nm

	Replicate 1	Replicate 2	Replicate 3	Replicate 4
	0,105599999	0,097999997	0,101899996	0,100500003

(540nm-690nm)-solvent control (540nm-690nm)

cocentration [M	Replicate 1	Replicate 2	Replicate 3	Replicate 4
1,00E-07	0,403699994	0,437899999	0,399000004	0,415599987
1,00E-06	0,406000018	0,4221	0,383800015	0,411800012
5,00E-06	0,375699997	0,385000013	0,386600003	0,409499988
1,00E-05	0,349900007	0,385599993	0,377899989	0,44249998
5,00E-05	0,370299995	0,414099984	0,394799992	0,434500024
1,00E-04	0,416299999	0,389500014	0,414400026	0,444299981
5,00E-04	0,48360002	0,4749	0,480599985	0,476999983

(540nm-690nm)-solvent control E2 reference

cocentration [M	Replicate 1	Replicate 2	Replicate 3	Replicate 4
1,00E-09	0,545099981	0,497699991	0,566499993	0,454399973

Normalization (substance/1 nM E2)

cocentration [M	Replicate 1	Replicate 2	Replicate 3	Replicate 4	Mean
1,00E-07	74,06%	87,98%	70,59%	91,46%	**81,02%**
1,00E-06	74,48%	84,81%	67,75%	90,63%	**79,42%**
5,00E-06	68,92%	77,36%	68,24%	90,12%	**76,16%**
1,00E-05	64,19%	77,48%	66,71%	97,38%	**76,44%**
5,00E-05	67,93%	83,20%	69,69%	95,62%	**79,11%**
1,00E-04	76,37%	78,26%	73,15%	97,78%	**81,39%**
5,00E-04	88,72%	95,42%	84,84%	104,97%	**93,49%**

yeast-growth/solubility 690nm/solvent control 690nm

cocentration [M	Replicate 1	Replicate 2	Replicate 3	Replicate 4	Mean
1,00E-07	79,47%	91,04%	76,11%	77,77%	**81,10%**
1,00E-06	77,25%	74,79%	78,22%	81,33%	**77,90%**
5,00E-06	76,05%	79,50%	82,34%	82,69%	**80,15%**
1,00E-05	73,62%	92,17%	79,54%	90,92%	**84,06%**
5,00E-05	77,60%	90,09%	81,65%	86,16%	**83,87%**
1,00E-04	80,43%	85,09%	87,95%	86,78%	**85,06%**
5,00E-04	90,09%	103,42%	89,88%	99,42%	**95,70%**

4-HT (540nm-690nm) anti-estrogenic control

cocentration [M	Replicate 1	Replicate 2	Replicate 3	Replicate 4
1,00E-06	0,060599998	0,0682	0,070000008	0,063799992

4-HT (540nm-690nm)/1 nM E2 anti-estrogenic control

cocentration [M	Replicate 1	Replicate 2	Replicate 3	Replicate 4	Mean
1,00E-06	11,12%	13,70%	12,36%	14,04%	**12,80%**

5th experiment

540nm

cocentration [M	Replicate 1	Replicate 2	Replicate 3	Replicate 4
1,00E-07	1,058500051	1,127300024	1,090399981	1,095800042
1,00E-06	1,075000048	1,167299986	1,129299998	1,080600023
5,00E-06	1,14349997	1,110399961	1,068600059	1,089100003
1,00E-05	1,037500024	1,092700005	1,131500006	1,073199987
5,00E-05	1,051399946	1,092000008	1,061100006	1,047600031
1,00E-04	1,042600036	1,061100006	1,066699982	1,086300015
5,00E-04	1,045899987	1,085600019	1,358299971	1,153300047

690nm

cocentration [M	Replicate 1	Replicate 2	Replicate 3	Replicate 4
1,00E-07	0,489100009	0,537999988	0,501800001	0,499799997
1,00E-06	0,505699992	0,555499971	0,528599977	0,485199988
5,00E-06	0,573499978	0,52700001	0,51639998	0,500699997
1,00E-05	0,488099992	0,499000013	0,528999984	0,498600006
5,00E-05	0,485700011	0,527800024	0,507300019	0,478599995
1,00E-04	0,482600003	0,503099978	0,515799999	0,523500025
5,00E-04	0,466800004	0,475600004	0,609799981	0,517400026

solvent control 690nm

	Replicate 1	Replicate 2	Replicate 3	Replicate 4
	0,595700026	0,568300009	0,586899996	0,530399978

540-690nm

cocentration [M	Replicate 1	Replicate 2	Replicate 3	Replicate 4
1,00E-07	0,569299996	0,589399993	0,588699996	0,596000016
1,00E-06	0,569199979	0,611699998	0,600700021	0,595399976
5,00E-06	0,569999993	0,583400011	0,552200019	0,588400006
1,00E-05	0,549399972	0,593699992	0,602500021	0,574599981
5,00E-05	0,565699995	0,564199984	0,553799987	0,569000006
1,00E-04	0,560000002	0,558000028	0,550899982	0,562699974
5,00E-04	0,579100013	0,610000014	0,74849999	0,635900021

solvent control 540nm-690nm

	Replicate 1	Replicate 2	Replicate 3	Replicate 4
	0,108199999	0,105599999	0,108599998	0,101800002

(540nm-690nm)-solvent control (540nm-690nm)

cocentration [M	Replicate 1	Replicate 2	Replicate 3	Replicate 4
1,00E-07	0,461099997	0,483799994	0,480099998	0,494200014
1,00E-06	0,460999981	0,506099999	0,492100023	0,493599974
5,00E-06	0,461799994	0,477800012	0,443600021	0,486600004
1,00E-05	0,441199973	0,488099992	0,493900023	0,472799979
5,00E-05	0,457499996	0,458599985	0,445199989	0,467200004
1,00E-04	0,451800004	0,452400029	0,442299984	0,460899971
5,00E-04	0,470900014	0,504400015	0,639899991	0,534100018

(540nm-690nm)-solvent control E2 reference

cocentration [M	Replicate 1	Replicate 2	Replicate 3	Replicate 4
1,00E-09	0,478699975	0,45010002	0,478799999	0,461399972

Normalization (substance/1 nM E2)

cocentration [M	Replicate 1	Replicate 2	Replicate 3	Replicate 4	**Mean**
1,00E-07	96,32%	107,49%	100,27%	107,11%	**102,80%**
1,00E-06	96,30%	112,44%	102,78%	106,98%	**104,63%**
5,00E-06	96,47%	106,15%	92,65%	105,46%	**100,18%**
1,00E-05	92,17%	108,44%	103,15%	102,47%	**101,56%**
5,00E-05	95,57%	101,89%	92,98%	101,26%	**97,92%**
1,00E-04	94,38%	100,51%	92,38%	99,89%	**96,79%**
5,00E-04	98,37%	112,06%	133,65%	115,76%	**114,96%**

yeast-growth/solubility 690nm/solvent control 690nm

cocentration [M	Replicate 1	Replicate 2	Replicate 3	Replicate 4	**Mean**
1,00E-07	82,11%	94,67%	85,50%	94,23%	**89,13%**
1,00E-06	84,89%	97,75%	90,07%	91,48%	**91,05%**
5,00E-06	96,27%	92,73%	87,99%	94,40%	**92,85%**
1,00E-05	81,94%	87,81%	90,13%	94,00%	**88,47%**
5,00E-05	81,53%	92,87%	86,44%	90,23%	**87,77%**
1,00E-04	81,01%	88,53%	87,89%	98,70%	**89,03%**
5,00E-04	78,36%	83,69%	103,90%	97,55%	**90,88%**

4-HT (540nm-690nm) anti-estrogenic control

cocentration [M	Replicate 1	Replicate 2	Replicate 3	Replicate 4
1,00E-06	0,047199994	0,051799998	0,045700006	0,059299998

4-HT (540nm-690nm)/1 nM E2 anti-estrogenic control

cocentration [M	Replicate 1	Replicate 2	Replicate 3	Replicate 4	**Mean**
1,00E-06	9,86%	11,51%	9,54%	12,85%	**10,94%**

cell-free 690nm

cocentration [M	Replicate 1	Replicate 2	Replicate 3	Replicate 4
1,00E-07	0,038800001	0,0396	0,037999999	0,0372
1,00E-06	0,037500001	0,0381	0,043400001	0,0374
5,00E-06	0,0381	0,038699999	0,038199998	0,037700001
1,00E-05	0,038199998	0,0381	0,037999999	0,037900001
5,00E-05	0,046799999	0,048	0,044199999	0,044199999
1,00E-04	0,052200001	0,0568	0,054299999	0,053199999
5,00E-04	0,1646	0,194199994	0,189799994	0,175699994

cell-free solvent control 690nm

cocentration [M	Replicate 1	Replicate 2	Replicate 3	Replicate 4
	0,037300002	0,0374	0,039799999	0,040100001

cell-free (substance/solvent control)

cocentration [M	Replicate 1	Replicate 2	Replicate 3	Replicate 4	**Mean**
1,00E-07	104,02%	105,88%	95,48%	92,77%	**99,54%**
1,00E-06	100,54%	101,87%	109,05%	93,27%	**101,18%**
5,00E-06	102,14%	103,48%	95,98%	94,01%	**98,90%**
1,00E-05	102,41%	101,87%	95,48%	94,51%	**98,57%**
5,00E-05	125,47%	128,34%	111,06%	110,22%	**118,77%**
1,00E-04	139,95%	151,87%	136,43%	132,67%	**140,23%**
5,00E-04	441,29%	519,25%	476,88%	438,15%	**468,89%**

YES pirimicarb

1st experient

540nm

cocentration [M]	Replicate 1	Replicate 2	Replicate 3	Replicate 4
1,00E-10	1,01970005	0,907599986	0,915000021	0,824599981
1,00E-09	0,950900018	0,930499971	0,916999996	0,825999975
1,00E-08	0,94569999	0,897400022	0,897199988	0,833599985
1,00E-07	0,93809998	0,929400027	0,921599984	0,870000005
1,00E-06	0,939400017	0,874000013	0,910899997	0,788200021
1,00E-05	0,91049999	0,939599991	0,884199977	0,788600028
1,00E-04	0,680100024	0,698199987	0,7324	0,843400002

690nm

cocentration [M]	Replicate 1	Replicate 2	Replicate 3	Replicate 4
1,00E-10	0,75150001	0,748300016	0,768000007	0,678300023
1,00E-09	0,784200013	0,776799977	0,760599971	0,675800025
1,00E-08	0,776000023	0,745800018	0,741900027	0,688399971
1,00E-07	0,789799988	0,763100028	0,76639998	0,718100011
1,00E-06	0,727199972	0,703599989	0,737600029	0,629999995
1,00E-05	0,718200028	0,752200007	0,734700024	0,646499991
1,00E-04	0,541299999	0,566399992	0,595399976	0,699500024

solvent control 690nm

	Replicate 1	Replicate 2	Replicate 3	Replicate 4
	0,67049998	0,716199994	0,696500003	0,718500018

540-690nm

cocentration [M]	Replicate 1	Replicate 2	Replicate 3	Replicate 4
1,00E-10	0,268299997	0,159299999	0,147	0,146300003
1,00E-09	0,166700006	0,153699994	0,156399995	0,150199994
1,00E-08	0,169599995	0,151600003	0,155300006	0,145199999
1,00E-07	0,148399994	0,166299999	0,155300006	0,151899993
1,00E-06	0,212200001	0,170399994	0,173299998	0,158299997
1,00E-05	0,192300007	0,187399998	0,149499997	0,142100006
1,00E-04	0,138799995	0,131799996	0,137099996	0,143900007

solvent control 540nm-690nm

	Replicate 1	Replicate 2	Replicate 3	Replicate 4
	0,145099998	0,1479	0,143299997	0,148900002

(540nm-690nm)-solvent control (540nm-690nm)

cocentration [M]	Replicate 1	Replicate 2	Replicate 3	Replicate 4
1,00E-10	0,123199999	0,011399999	0,003700003	-0,002599999
1,00E-09	0,021600008	0,005799994	0,013099998	0,001299992
1,00E-08	0,024499997	0,003700003	0,012000009	-0,003700003
1,00E-07	0,003299996	0,018399999	0,012000009	0,002999991
1,00E-06	0,067100003	0,022499993	0,030000001	0,009399995
1,00E-05	0,047200009	0,039499998	0,006200001	-0,006799996
1,00E-04	-0,006300002	-0,016100004	-0,006200001	-0,004999995

(540nm-690nm)-solvent control E2 reference

cocentration [M]	Replicate 1	Replicate 2	Replicate 3	Replicate 4
1,00E-09	0,288800001	0,275399998	0,277500004	0,264999986

Normalization (substance/1 nM E2)

cocentration [M]	Replicate 1	Replicate 2	Replicate 3	Replicate 4	**Mean**
1,00E-10	42,66%	4,14%	1,33%	-0,98%	**11,79%**
1,00E-09	7,48%	2,11%	4,72%	0,49%	**3,70%**
1,00E-08	8,48%	1,34%	4,32%	-1,40%	**3,19%**
1,00E-07	1,14%	6,68%	4,32%	1,13%	**3,32%**
1,00E-06	23,23%	8,17%	10,81%	3,55%	**11,44%**
1,00E-05	16,34%	14,34%	2,23%	-2,57%	**7,59%**
1,00E-04	-2,18%	-5,85%	-2,23%	-1,89%	**-3,04%**

yeast-growth/solubility 690nm/solvent control 690nm

cocentration [M]	Replicate 1	Replicate 2	Replicate 3	Replicate 4	**Mean**
1,00E-10	112,08%	104,48%	110,27%	94,41%	**105,31%**
1,00E-09	116,96%	108,46%	109,20%	94,06%	**107,17%**
1,00E-08	115,73%	104,13%	106,52%	95,81%	**105,55%**
1,00E-07	117,79%	106,55%	110,04%	99,94%	**108,58%**
1,00E-06	108,46%	98,24%	105,90%	87,68%	**100,07%**
1,00E-05	107,11%	105,03%	105,48%	89,98%	**101,90%**
1,00E-04	80,73%	79,08%	85,48%	97,36%	**85,66%**

2nd experient

540nm

cocentration [M]	Replicate 1	Replicate 2	Replicate 3	Replicate 4
1,00E-10	0,966799974	0,953400016	0,891099989	0,791299999
1,00E-09	0,868200004	0,878000021	0,926199973	0,802399993
1,00E-08	0,906599998	0,908100009	0,989099979	1,033100009
1,00E-07	0,864600003	0,925899982	0,956700027	0,882799983
1,00E-06	0,858399987	0,955200016	0,915899992	0,708000004
1,00E-05	0,878199995	0,881500006	0,938000023	0,823400021
1,00E-04	0,995700002	0,996800005	1,037700057	0,917400002

690nm

cocentration [M]	Replicate 1	Replicate 2	Replicate 3	Replicate 4
1,00E-10	0,82130003	0,809199989	0,754999995	0,680299997
1,00E-09	0,742699981	0,744499981	0,793200016	0,694299996
1,00E-08	0,771899998	0,769900024	0,847299993	0,896899998
1,00E-07	0,724900007	0,783100009	0,81370002	0,763800025
1,00E-06	0,719600022	0,810000002	0,773199975	0,600499988
1,00E-05	0,729300022	0,74150002	0,790799975	0,701499999
1,00E-04	0,8574	0,860700011	0,899399996	0,795099974

solvent control 690nm

	Replicate 1	Replicate 2	Replicate 3	Replicate 4
	0,57980001	0,720700026	0,676400006	0,709299982

540-690nm

cocentration [M]	Replicate 1	Replicate 2	Replicate 3	Replicate 4
1,00E-10	0,145500004	0,144199997	0,136199996	0,111000001
1,00E-09	0,125599995	0,133399993	0,133100003	0,108099997
1,00E-08	0,1347	0,1382	0,141800001	0,136199996
1,00E-07	0,139799997	0,142800003	0,143000007	0,119000003
1,00E-06	0,138799995	0,145199999	0,142700002	0,107500002
1,00E-05	0,148900002	0,140000001	0,147300005	0,1219
1,00E-04	0,138300002	0,136099994	0,138300002	0,122299999

solvent control 540nm-690nm

	Replicate 1	Replicate 2	Replicate 3	Replicate 4
	0,172299996	0,137999997	0,136600003	0,138699993

(540nm-690nm)-solvent control (540nm-690nm)

cocentration [M]	Replicate 1	Replicate 2	Replicate 3	Replicate 4
1,00E-10	-0,026799992	0,006200001	-0,000400007	-0,027699992
1,00E-09	-0,046700001	-0,004600003	-0,0035	-0,030599996
1,00E-08	-0,037599996	0,000200003	0,005199999	-0,002499998
1,00E-07	-0,032499999	0,004800007	0,006400004	-0,019699991
1,00E-06	-0,033500001	0,007200003	0,006099999	-0,031199992
1,00E-05	-0,023399994	0,002000004	0,010700002	-0,016799994
1,00E-04	-0,033999994	-0,001900002	0,001699999	-0,016399994

(540nm-690nm)-solvent control E2 reference

cocentration [M]	Replicate 1	Replicate 2	Replicate 3	Replicate 4
1,00E-09	0,28109999	0,352200016	0,36119999	0,35270001

Normalization (substance/1 nM E2)

cocentration [M]	Replicate 1	Replicate 2	Replicate 3	Replicate 4	**Mean**
1,00E-10	-9,53%	1,76%	-0,11%	-7,85%	**-3,93%**
1,00E-09	-16,61%	-1,31%	-0,97%	-8,68%	**-6,89%**
1,00E-08	-13,38%	0,06%	1,44%	-0,71%	**-3,15%**
1,00E-07	-11,56%	1,36%	1,77%	-5,59%	**-3,50%**
1,00E-06	-11,92%	2,04%	1,69%	-8,85%	**-4,26%**
1,00E-05	-8,32%	0,57%	2,96%	-4,76%	**-2,39%**
1,00E-04	-12,10%	-0,54%	0,47%	-4,65%	**-4,20%**

yeast-growth/solubility 690nm/solvent control 690nm

cocentration [M]	Replicate 1	Replicate 2	Replicate 3	Replicate 4	**Mean**
1,00E-10	141,65%	112,28%	111,62%	95,91%	**115,37%**
1,00E-09	128,10%	103,30%	117,27%	97,89%	**111,64%**
1,00E-08	133,13%	106,83%	125,27%	126,45%	**122,92%**
1,00E-07	125,03%	108,66%	120,30%	107,68%	**115,42%**
1,00E-06	124,11%	112,39%	114,31%	84,66%	**108,87%**
1,00E-05	125,78%	102,89%	116,91%	98,90%	**111,12%**
1,00E-04	147,88%	119,43%	132,97%	112,10%	**128,09%**

3rd experiment

540nm

cocentration [M	Replicate 1	Replicate 2	Replicate 3	Replicate 4
1,00E-10	1,022699952	0,915600002	0,871699989	0,825699985
1,00E-09	0,912100017	0,904200017	0,890399992	0,797699988
1,00E-08	1,051699996	0,929499984	0,922999978	0,809800029
1,00E-07	0,954299986	0,922599971	0,873700023	0,786199987
1,00E-06	0,931400001	0,888199985	0,925199986	0,735599995
1,00E-05	0,899299979	0,862299979	0,851199985	0,77609998
1,00E-04	0,737699986	0,769599974	0,502300024	0,730199993

690nm

cocentration [M	Replicate 1	Replicate 2	Replicate 3	Replicate 4
1,00E-10	0,734300017	0,750800014	0,722100019	0,675300002
1,00E-09	0,729799986	0,756600022	0,743900001	0,650300026
1,00E-08	0,800800025	0,766600013	0,772700012	0,666400015
1,00E-07	0,754400015	0,771000028	0,726599991	0,644800007
1,00E-06	0,750100017	0,741100013	0,776600003	0,600000024
1,00E-05	0,667299986	0,711199999	0,708400011	0,633099973
1,00E-04	0,60589999	0,644500017	0,413399994	0,610199988

solvent control 690nm

	Replicate 1	Replicate 2	Replicate 3	Replicate 4
	0,458999991	0,599300027	0,644500017	0,652700007

540-690nm

cocentration [M	Replicate 1	Replicate 2	Replicate 3	Replicate 4
1,00E-10	0,288399994	0,164800003	0,149599999	0,150399998
1,00E-09	0,1822	0,147599995	0,146500006	0,147400007
1,00E-08	0,2509	0,163000003	0,150299996	0,143399999
1,00E-07	0,199900001	0,151500002	0,147100002	0,141399994
1,00E-06	0,181299999	0,147100002	0,148599997	0,135600001
1,00E-05	0,232099995	0,151099995	0,142800003	0,143000007
1,00E-04	0,131799996	0,125200003	0,0889	0,119999997

solvent control 540nm-690nm

	Replicate 1	Replicate 2	Replicate 3	Replicate 4
	0,142299995	0,150700003	0,152099997	0,143600002

(540nm-690nm)-solvent control (540nm-690nm)

cocentration [M	Replicate 1	Replicate 2	Replicate 3	Replicate 4
1,00E-10	0,1461	0,0141	-0,002499998	0,006799996
1,00E-09	0,039900005	-0,003100008	-0,00559999	0,003800005
1,00E-08	0,108600006	0,0123	-0,001800001	-0,000200003
1,00E-07	0,057600006	0,000799999	-0,004999995	-0,002200007
1,00E-06	0,039000005	-0,003600001	-0,0035	-0,008000001
1,00E-05	0,0898	0,000399992	-0,009299994	-0,000599995
1,00E-04	-0,010499999	-0,0255	-0,063199997	-0,023600005

(540nm-690nm)-solvent control E2 reference

cocentration [M	Replicate 1	Replicate 2	Replicate 3	Replicate 4
1,00E-09	0,304000005	0,229799986	0,252800018	0,23560001

Normalization (substance/1 nM E2)

cocentration [M	Replicate 1	Replicate 2	Replicate 3	Replicate 4	**Mean**
1,00E-10	48,06%	6,14%	-0,99%	2,89%	**14,02%**
1,00E-09	13,13%	-1,35%	-2,22%	1,61%	**2,79%**
1,00E-08	35,72%	5,35%	-0,71%	-0,08%	**10,07%**
1,00E-07	18,95%	0,35%	-1,98%	-0,93%	**4,10%**
1,00E-06	12,83%	-1,57%	-1,38%	-3,40%	**1,62%**
1,00E-05	29,54%	0,17%	-3,68%	-0,25%	**6,45%**
1,00E-04	-3,45%	-11,10%	-25,00%	-10,02%	**-12,39%**

yeast-growth/solubility 690nm/solvent control 690nm

cocentration [M	Replicate 1	Replicate 2	Replicate 3	Replicate 4	**Mean**
1,00E-10	159,98%	125,28%	112,04%	103,46%	**125,19%**
1,00E-09	159,00%	126,25%	115,42%	99,63%	**125,08%**
1,00E-08	174,47%	127,92%	119,89%	102,10%	**131,09%**
1,00E-07	164,36%	128,65%	112,74%	98,79%	**126,13%**
1,00E-06	163,42%	123,66%	120,50%	91,93%	**124,88%**
1,00E-05	145,38%	118,67%	109,91%	97,00%	**117,74%**
1,00E-04	132,00%	107,54%	64,14%	93,49%	**99,29%**

4th experiment

540nm

cocentration [M	Replicate 1	Replicate 2	Replicate 3	Replicate 4
1,00E-10	0,986599982	0,984000027	0,986000001	0,915799975
1,00E-09	1,166900039	0,987600029	0,999100029	1,006299973
1,00E-08	1,113399982	0,96359998	0,994899988	0,809700012
1,00E-07	1,004199982	0,880299985	0,937900007	0,897400022
1,00E-06	1,022799969	0,97420001	0,906099975	0,895200014
1,00E-05	0,994199991	0,925400019	0,9023	0,848800004
1,00E-04	0,934800029	0,880100012	0,994799972	0,915499985

690nm

cocentration [M	Replicate 1	Replicate 2	Replicate 3	Replicate 4
1,00E-10	0,79369998	0,809000015	0,821200013	0,75910002
1,00E-09	0,81309998	0,782999992	0,826099992	0,845499992
1,00E-08	0,822700024	0,801800013	0,814899981	0,663299978
1,00E-07	0,83950001	0,726100028	0,779399991	0,740400016
1,00E-06	0,789699972	0,806100011	0,753099978	0,740700006
1,00E-05	0,780399978	0,769699991	0,754000008	0,702400029
1,00E-04	0,753899992	0,734899998	0,850300014	0,770500004

solvent control 690nm

	Replicate 1	Replicate 2	Replicate 3	Replicate 4
	0,755599976	0,791999996	0,779900014	0,713800013

540-690nm

cocentration [M	Replicate 1	Replicate 2	Replicate 3	Replicate 4
1,00E-10	0,193000004	0,174999997	0,164800003	0,1567
1,00E-09	0,353799999	0,204600006	0,173099995	0,160699993
1,00E-08	0,290699989	0,161799997	0,180000007	0,146400005
1,00E-07	0,1646	0,154200003	0,158500001	0,157000005
1,00E-06	0,233099997	0,167999998	0,152999997	0,154499993
1,00E-05	0,213799998	0,155699998	0,148399994	0,146400005
1,00E-04	0,180899993	0,145199999	0,144400001	0,144999996

solvent control 540nm-690nm

	Replicate 1	Replicate 2	Replicate 3	Replicate 4
	0,158800006	0,156399995	0,156599998	0,1461

(540nm-690nm)-solvent control (540nm-690nm)

cocentration [M	Replicate 1	Replicate 2	Replicate 3	Replicate 4
1,00E-10	0,034199998	0,018600002	0,008200005	0,010600001
1,00E-09	0,194999993	0,048200011	0,016499996	0,014599994
1,00E-08	0,131899983	0,005400002	0,023400009	0,000300005
1,00E-07	0,005799994	-0,002199993	0,001900002	0,010900006
1,00E-06	0,074299991	0,011600003	-0,003600001	0,008399993
1,00E-05	0,054999992	-0,000699997	-0,008200005	0,000300005
1,00E-04	0,022099987	-0,011199996	-0,012199998	-0,001100004

(540nm-690nm)-solvent control E2 reference

cocentration [M	Replicate 1	Replicate 2	Replicate 3	Replicate 4
1,00E-09	0,547500014	0,585800022	0,578599989	0,581199989

Normalization (substance/1 nM E2)

cocentration [M	Replicate 1	Replicate 2	Replicate 3	Replicate 4	**Mean**
1,00E-10	6,25%	3,18%	1,42%	1,82%	**3,17%**
1,00E-09	35,62%	8,23%	2,85%	2,51%	**12,30%**
1,00E-08	24,09%	0,92%	4,04%	0,05%	**7,28%**
1,00E-07	1,06%	-0,38%	0,33%	1,88%	**0,72%**
1,00E-06	13,57%	1,98%	-0,62%	1,45%	**4,09%**
1,00E-05	10,05%	-0,12%	-1,42%	0,05%	**2,14%**
1,00E-04	4,04%	-1,91%	-2,11%	-0,19%	**-0,04%**

yeast-growth/solubility 690nm/solvent control 690nm

cocentration [M	Replicate 1	Replicate 2	Replicate 3	Replicate 4	**Mean**
1,00E-10	105,04%	102,15%	105,30%	106,35%	**104,71%**
1,00E-09	107,61%	98,86%	105,92%	118,45%	**107,71%**
1,00E-08	108,88%	101,24%	104,49%	92,93%	**101,88%**
1,00E-07	111,10%	91,68%	99,94%	103,73%	**101,61%**
1,00E-06	104,51%	101,78%	96,56%	103,77%	**101,66%**
1,00E-05	103,28%	97,18%	96,68%	98,40%	**98,89%**
1,00E-04	99,78%	92,79%	109,03%	107,94%	**102,38%**

5th experiment

540nm

cocentration [M	Replicate 1	Replicate 2	Replicate 3	Replicate 4
1,00E-10	1,069599986	1,030699968	1,011600018	1,116299987
1,00E-09	1,033499956	0,965699971	1,059599996	1,113000035
1,00E-08	0,99150002	1,002699971	0,982500017	0,901499987
1,00E-07	0,979900002	1,004799962	0,972800016	0,734200001
1,00E-06	1,001899958	1,023599982	0,970899999	0,988699973
1,00E-05	0,939899981	0,949699998	0,966899991	0,980799973
1,00E-04	0,91049999	0,916700006	0,985199988	1,097800016

690nm

cocentration [M	Replicate 1	Replicate 2	Replicate 3	Replicate 4
1,00E-10	0,904600024	0,883300006	0,866599977	0,966899991
1,00E-09	0,877799988	0,819999993	0,910700023	0,961199999
1,00E-08	0,835799992	0,856100023	0,833700001	0,768100023
1,00E-07	0,832099974	0,856000006	0,827000022	0,613799989
1,00E-06	0,855799973	0,877099991	0,827099979	0,84740001
1,00E-05	0,796299994	0,803799987	0,821099997	0,836499989
1,00E-04	0,771700025	0,777800024	0,845000029	0,95599997

solvent control 690nm

	Replicate 1	Replicate 2	Replicate 3	Replicate 4
	0,909799993	0,839999974	0,772499979	0,877799988

540-690nm

cocentration [M	Replicate 1	Replicate 2	Replicate 3	Replicate 4
1,00E-10	0,165000007	0,147300005	0,144999996	0,149399996
1,00E-09	0,155699998	0,145699993	0,148900002	0,151800007
1,00E-08	0,155699998	0,146599993	0,148800001	0,133399993
1,00E-07	0,147699997	0,148900002	0,145799994	0,120399997
1,00E-06	0,1461	0,146500006	0,143800005	0,141299993
1,00E-05	0,143600002	0,145899996	0,145799994	0,144299999
1,00E-04	0,138899997	0,138899997	0,140200004	0,141800001

solvent control 540nm-690nm

	Replicate 1	Replicate 2	Replicate 3	Replicate 4
	0,152099997	0,144099995	0,137600005	0,142199993

(540nm-690nm)-solvent control (540nm-690nm)

cocentration [M	Replicate 1	Replicate 2	Replicate 3	Replicate 4
1,00E-10	0,01290001	0,003200009	0,007399991	0,007200003
1,00E-09	0,003600001	0,001599997	0,011299998	0,009600013
1,00E-08	0,003600001	0,002499998	0,011199996	-0,0088
1,00E-07	-0,0044	0,004800007	0,00819999	-0,021799996
1,00E-06	-0,005999997	0,002400011	0,006200001	-0,0009
1,00E-05	-0,008499995	0,001800001	0,00819999	0,002100006
1,00E-04	-0,0132	-0,005199999	0,002599999	-0,000399992

(540nm-690nm)-solvent control E2 reference

cocentration [M	Replicate 1	Replicate 2	Replicate 3	Replicate 4
1,00E-09	0,498999983	0,503600016	0,50999999	0,501399994

Normalization (substance/1 nM E2)

cocentration [M	Replicate 1	Replicate 2	Replicate 3	Replicate 4	Mean
1,00E-10	2,59%	0,64%	1,45%	1,44%	**1,53%**
1,00E-09	0,72%	0,32%	2,22%	1,91%	**1,29%**
1,00E-08	0,72%	0,50%	2,20%	-1,76%	**0,41%**
1,00E-07	-0,88%	0,95%	1,61%	-4,35%	**-0,67%**
1,00E-06	-1,20%	0,48%	1,22%	-0,18%	**0,08%**
1,00E-05	-1,70%	0,36%	1,61%	0,42%	**0,17%**
1,00E-04	-2,65%	-1,03%	0,51%	-0,08%	**-0,81%**

yeast-growth/solubility 690nm/solvent control 690nm

cocentration [M	Replicate 1	Replicate 2	Replicate 3	Replicate 4	Mean
1,00E-10	99,43%	105,15%	112,18%	110,15%	**106,73%**
1,00E-09	96,48%	97,62%	117,89%	109,50%	**105,37%**
1,00E-08	91,87%	101,92%	107,92%	87,50%	**97,30%**
1,00E-07	91,46%	101,90%	107,06%	69,92%	**92,59%**
1,00E-06	94,06%	104,42%	107,07%	96,54%	**100,52%**
1,00E-05	87,52%	95,69%	106,29%	95,30%	**96,20%**
1,00E-04	84,82%	92,60%	109,39%	108,91%	**98,93%**

6th experiment

540nm

cocentration [M	Replicate 1	Replicate 2	Replicate 3	Replicate 4
1,00E-10	0,843500018	0,884800017	0,869199991	0,851499975
1,00E-09	0,878700018	0,906000018	0,888199985	0,817700028
1,00E-08	0,871200025	0,96329999	0,984000027	0,844099998
1,00E-07	0,865800023	0,929899991	0,898299992	0,794200003
1,00E-06	0,852699995	0,868700027	0,860000014	0,825699985
1,00E-05	0,883899987	0,862299979	0,885999978	0,810899973
1,00E-04	0,763499975	0,824000001	0,765600026	0,771899998

690nm

cocentration [M	Replicate 1	Replicate 2	Replicate 3	Replicate 4
1,00E-10	0,681400001	0,736899972	0,724699974	0,708299994
1,00E-09	0,728600025	0,755999982	0,740700006	0,677999973
1,00E-08	0,719699979	0,81279999	0,831200004	0,701600015
1,00E-07	0,720000029	0,780399978	0,751600027	0,653999984
1,00E-06	0,701900005	0,72240001	0,715699971	0,691799998
1,00E-05	0,734399974	0,717299998	0,742299974	0,671500027
1,00E-04	0,629800022	0,688300014	0,637499988	0,6426

solvent control 690nm

	Replicate 1	Replicate 2	Replicate 3	Replicate 4
	0,657000005	0,642099977	0,66839999	0,686699986

540-690nm

cocentration [M	Replicate 1	Replicate 2	Replicate 3	Replicate 4
1,00E-10	0,162200004	0,1479	0,144500002	0,143199995
1,00E-09	0,150099993	0,150000006	0,147499993	0,139799997
1,00E-08	0,151500002	0,1505	0,152799994	0,142499998
1,00E-07	0,145799994	0,149499997	0,146699995	0,140100002
1,00E-06	0,150800005	0,146300003	0,144400001	0,133900002
1,00E-05	0,149499997	0,144999996	0,143700004	0,139400005
1,00E-04	0,1338	0,135700002	0,128099993	0,129199997

solvent control 540nm-690nm

	Replicate 1	Replicate 2	Replicate 3	Replicate 4
	0,139200002	0,139599994	0,142399997	0,143099993

(540nm-690nm)-solvent control (540nm-690nm)

cocentration [M	Replicate 1	Replicate 2	Replicate 3	Replicate 4
1,00E-10	0,023000002	0,008300006	0,002100006	0,000100002
1,00E-09	0,010899991	0,010400012	0,005099997	-0,003299996
1,00E-08	0,0123	0,010900006	0,010399997	-0,000599995
1,00E-07	0,006599993	0,009900004	0,004299998	-0,002999991
1,00E-06	0,011600003	0,006700009	0,002000004	-0,009199992
1,00E-05	0,010299996	0,005400002	0,001300007	-0,003699988
1,00E-04	-0,005400002	-0,003899992	-0,014300004	-0,013899997

(540nm-690nm)-solvent control E2 reference

cocentration [M	Replicate 1	Replicate 2	Replicate 3	Replicate 4
1,00E-09	0,683299989	0,639200017	0,670399994	0,671300024

Normalization (substance/1 nM E2)

cocentration [M	Replicate 1	Replicate 2	Replicate 3	Replicate 4	Mean
1,00E-10	3,37%	1,30%	0,31%	0,01%	**1,25%**
1,00E-09	1,60%	1,63%	0,76%	-0,49%	**0,87%**
1,00E-08	1,80%	1,71%	1,55%	-0,09%	**1,24%**
1,00E-07	0,97%	1,55%	0,64%	-0,45%	**0,68%**
1,00E-06	1,70%	1,05%	0,30%	-1,37%	**0,42%**
1,00E-05	1,51%	0,84%	0,19%	-0,55%	**0,50%**
1,00E-04	-0,79%	-0,61%	-2,13%	-2,07%	**-1,40%**

yeast-growth/solubility 690nm/solvent control 690nm

cocentration [M	Replicate 1	Replicate 2	Replicate 3	Replicate 4	Mean
1,00E-10	103,71%	114,76%	108,42%	103,15%	**107,51%**
1,00E-09	110,90%	117,74%	110,82%	98,73%	**109,55%**
1,00E-08	109,54%	126,58%	124,36%	102,17%	**115,66%**
1,00E-07	109,59%	121,54%	112,45%	95,24%	**109,70%**
1,00E-06	106,83%	112,51%	107,08%	100,74%	**106,79%**
1,00E-05	111,78%	111,71%	111,06%	97,79%	**108,08%**
1,00E-04	95,86%	107,20%	95,38%	93,58%	**98,00%**

7th experiment

540nm

cocentration [M	Replicate 1	Replicate 2	Replicate 3	Replicate 4
1,00E-10	0,932900012	0,941500008	0,909699976	0,883099973
1,00E-09	0,879800022	0,903900027	0,899399996	0,849099994
1,00E-08	0,912800014	0,870800018	0,904200017	0,880299985
1,00E-07	0,924199998	0,943599999	0,90140003	0,82069999
1,00E-06	0,917299986	0,885500014	0,893999994	0,7852
1,00E-05	0,90259999	0,946699977	0,871999979	0,857599974
1,00E-04	0,816500008	0,850700021	1,324200034	0,840600014

690nm

cocentration [M	Replicate 1	Replicate 2	Replicate 3	Replicate 4
1,00E-10	0,752099991	0,772700012	0,748899996	0,723200023
1,00E-09	0,716899991	0,744199991	0,738300025	0,691200018
1,00E-08	0,734399974	0,716000021	0,744700015	0,721499979
1,00E-07	0,754400015	0,781899989	0,742699981	0,666199982
1,00E-06	0,743600011	0,726499975	0,737299979	0,634299994
1,00E-05	0,731899977	0,782299995	0,716700017	0,698300004
1,00E-04	0,65990001	0,701300025	0,78579998	0,705299973

solvent control 690nm

	Replicate 1	Replicate 2	Replicate 3	Replicate 4
	0,558899999	0,740800023	0,768400013	0,739899993

540-690nm

cocentration [M	Replicate 1	Replicate 2	Replicate 3	Replicate 4
1,00E-10	0,180899993	0,168799996	0,160899997	0,159899995
1,00E-09	0,163000003	0,159700006	0,1611	0,157900006
1,00E-08	0,178399995	0,154799998	0,159500003	0,158800006
1,00E-07	0,169799998	0,161699995	0,158700004	0,154400006
1,00E-06	0,173700005	0,158999994	0,156599998	0,150999993
1,00E-05	0,170599997	0,164499998	0,155300006	0,159199998
1,00E-04	0,156599998	0,149399996	0,538399994	0,135299996

solvent control 540nm-690nm

	Replicate 1	Replicate 2	Replicate 3	Replicate 4
	0,111299999	0,159500003	0,161799997	0,159700006

(540nm-690nm)-solvent control (540nm-690nm)

cocentration [M	Replicate 1	Replicate 2	Replicate 3	Replicate 4
1,00E-10	0,069599994	0,009299994	-0,0009	0,000199988
1,00E-09	0,051700003	0,000200003	-0,000699997	-0,001800001
1,00E-08	0,067099996	-0,004700005	-0,002299994	-0,0009
1,00E-07	0,058499999	0,002199993	-0,003099993	-0,0053
1,00E-06	0,062400006	-0,000500008	-0,005199999	-0,008700013
1,00E-05	0,059299998	0,004999995	-0,006499991	-0,000500008
1,00E-04	0,045299999	-0,010100007	0,376599997	-0,024400011

(540nm-690nm)-solvent control E2 reference

cocentration [M	Replicate 1	Replicate 2	Replicate 3	Replicate 4
1,00E-09	0,700200014	0,651600003	0,658099979	0,669500023

Normalization (substance/1 nM E2)

cocentration [M	Replicate 1	Replicate 2	Replicate 3	Replicate 4	Mean
1,00E-10	9,94%	1,43%	-0,14%	0,03%	**2,82%**
1,00E-09	7,38%	0,03%	-0,11%	-0,27%	**1,76%**
1,00E-08	9,58%	-0,72%	-0,35%	-0,13%	**2,09%**
1,00E-07	8,35%	0,34%	-0,47%	-0,79%	**1,86%**
1,00E-06	8,91%	-0,08%	-0,79%	-1,30%	**1,69%**
1,00E-05	8,47%	0,77%	-0,99%	-0,07%	**2,04%**
1,00E-04	6,47%	-1,55%	57,23%	-3,64%	**14,63%**

yeast-growth/solubility 690nm/solvent control 690nm

cocentration [M	Replicate 1	Replicate 2	Replicate 3	Replicate 4	Mean
1,00E-10	134,57%	104,31%	97,46%	97,74%	**108,52%**
1,00E-09	128,27%	100,46%	96,08%	93,42%	**104,56%**
1,00E-08	131,40%	96,65%	96,92%	97,51%	**105,62%**
1,00E-07	134,98%	105,55%	96,66%	90,04%	**106,81%**
1,00E-06	133,05%	98,07%	95,95%	85,73%	**103,20%**
1,00E-05	130,95%	105,60%	93,27%	94,38%	**106,05%**
1,00E-04	118,07%	94,67%	102,26%	95,32%	**102,58%**

8th experiment

540nm

cocentration [M	Replicate 1	Replicate 2	Replicate 3	Replicate 4
1,00E-10	0,895600021	0,8671	0,874599993	0,89380002
1,00E-09	0,929499984	0,895500004	0,908200026	0,908100009
1,00E-08	0,887000024	0,870299995	0,905799985	0,88499999
1,00E-07	0,932799995	0,896600008	0,888999999	0,895699978
1,00E-06	0,913200021	0,871500015	0,882099986	0,867200017
1,00E-05	1,029800057	0,902100027	0,895500004	0,859499991
1,00E-04	0,911000013	0,83920002	0,848399997	0,834500015

690nm

cocentration [M	Replicate 1	Replicate 2	Replicate 3	Replicate 4
1,00E-10	0,728999972	0,718400002	0,726300001	0,741400003
1,00E-09	0,753499985	0,744899988	0,756699979	0,757499993
1,00E-08	0,724399984	0,721599996	0,757399976	0,737600029
1,00E-07	0,742600024	0,742799997	0,740100026	0,745299995
1,00E-06	0,751600027	0,726100028	0,736800015	0,720600009
1,00E-05	0,780399978	0,73210001	0,746900022	0,711899996
1,00E-04	0,701799989	0,705600023	0,714900017	0,702400029

solvent control 690nm

	Replicate 1	Replicate 2	Replicate 3	Replicate 4
	0,746900022	0,730700016	0,753099978	0,693000019

540-690nm

cocentration [M	Replicate 1	Replicate 2	Replicate 3	Replicate 4
1,00E-10	0,166600004	0,148599997	0,148399994	0,152400002
1,00E-09	0,175999999	0,150600001	0,151500002	0,150600001
1,00E-08	0,162599996	0,148699999	0,148399994	0,147400007
1,00E-07	0,190200001	0,153799996	0,149000004	0,150399998
1,00E-06	0,161699995	0,145400003	0,145300001	0,146599993
1,00E-05	0,249300003	0,170000002	0,148599997	0,147599995
1,00E-04	0,209199995	0,133599997	0,133499995	0,132100001

solvent control 540nm-690nm

	Replicate 1	Replicate 2	Replicate 3	Replicate 4
	0,154499993	0,150399998	0,151299998	0,145199999

(540nm-690nm)-solvent control (540nm-690nm)

cocentration [M	Replicate 1	Replicate 2	Replicate 3	Replicate 4
1,00E-10	0,012100011	-0,001800001	-0,002900004	0,007200003
1,00E-09	0,021500006	0,000200003	0,000200003	0,005400002
1,00E-08	0,008100003	-0,001699999	-0,002900004	0,002200007
1,00E-07	0,035700008	0,003399998	-0,002299994	0,005199999
1,00E-06	0,007200003	-0,004999995	-0,005999997	0,001399994
1,00E-05	0,09480001	0,019600004	-0,002700001	0,002399996
1,00E-04	0,054700002	-0,016800001	-0,017800003	-0,013099998

(540nm-690nm)-solvent control E2 reference

cocentration [M	Replicate 1	Replicate 2	Replicate 3	Replicate 4
1,00E-09	0,323899999	0,312899992	0,313000008	0,324100003

Normalization (substance/1 nM E2)

cocentration [M	Replicate 1	Replicate 2	Replicate 3	Replicate 4	Mean
1,00E-10	3,74%	-0,58%	-0,93%	2,22%	**1,11%**
1,00E-09	6,64%	0,06%	0,06%	1,67%	**2,11%**
1,00E-08	2,50%	-0,54%	-0,93%	0,68%	**0,43%**
1,00E-07	11,02%	1,09%	-0,73%	1,60%	**3,24%**
1,00E-06	2,22%	-1,60%	-1,92%	0,43%	**-0,22%**
1,00E-05	29,27%	6,26%	-0,86%	0,74%	**8,85%**
1,00E-04	16,89%	-5,37%	-5,69%	-4,04%	**0,45%**

yeast-growth/solubility 690nm/solvent control 690nm

cocentration [M	Replicate 1	Replicate 2	Replicate 3	Replicate 4	Mean
1,00E-10	97,60%	98,32%	96,44%	106,98%	**99,84%**
1,00E-09	100,88%	101,94%	100,48%	109,31%	**103,15%**
1,00E-08	96,99%	98,75%	100,57%	106,44%	**100,69%**
1,00E-07	99,42%	101,66%	98,27%	107,55%	**101,73%**
1,00E-06	100,63%	99,37%	97,84%	103,98%	**100,45%**
1,00E-05	104,49%	100,19%	99,18%	102,73%	**101,65%**
1,00E-04	93,96%	96,56%	94,93%	101,36%	**96,70%**

YES propamocarb

1st experient

540nm

cocentration [M]	Replicate 1	Replicate 2	Replicate 3	Replicate 4
1,00E-10	0,932500005	0,869400024	0,729300022	0,778500021
1,00E-09	0,865700006	0,856700003	0,91140002	0,818599999
1,00E-08	0,860199988	0,797699988	0,845600009	0,731299996
1,00E-07	0,856000006	0,847199976	0,862100005	0,71509999
1,00E-06	0,815999985	0,795400023	0,826399982	0,730300009
1,00E-05	0,817900002	0,770900011	0,754100025	0,713500023
1,00E-04	0,72420001	0,844200015	0,727800012	0,725899994

690nm

cocentration [M]	Replicate 1	Replicate 2	Replicate 3	Replicate 4
1,00E-10	0,751399994	0,720899999	0,593900025	0,634599984
1,00E-09	0,712100029	0,712100029	0,752300024	0,667500019
1,00E-08	0,715200007	0,659099996	0,697399974	0,594799995
1,00E-07	0,710600019	0,701499999	0,712599993	0,580500007
1,00E-06	0,67049998	0,653900027	0,682699978	0,590499997
1,00E-05	0,676400006	0,6347	0,620700002	0,577000022
1,00E-04	0,597000003	0,675100029	0,598999977	0,601400018

solvent control 690nm

	Replicate 1	Replicate 2	Replicate 3	Replicate 4
	0,653699994	0,648899972	0,654100001	0,693400025

540-690nm

cocentration [M]	Replicate 1	Replicate 2	Replicate 3	Replicate 4
1,00E-10	0,181099996	0,148499995	0,135499999	0,143900007
1,00E-09	0,153500006	0,144600004	0,159099996	0,151199996
1,00E-08	0,144999996	0,138600007	0,148100004	0,136500001
1,00E-07	0,145500004	0,145799994	0,149499997	0,135499999
1,00E-06	0,145600006	0,141499996	0,143600002	0,139799997
1,00E-05	0,141499996	0,136299998	0,133300006	0,136500001
1,00E-04	0,127200007	0,169100001	0,128800005	0,124499999

solvent control 540nm-690nm

	Replicate 1	Replicate 2	Replicate 3	Replicate 4
	0,140799999	0,140100002	0,134399995	0,142000005

(540nm-690nm)-solvent control (540nm-690nm)

cocentration [M]	Replicate 1	Replicate 2	Replicate 3	Replicate 4
1,00E-10	0,040299997	0,008399993	0,001100004	0,001900002
1,00E-09	0,012700006	0,004500002	0,024700001	0,009199992
1,00E-08	0,004199997	-0,001499996	0,013700008	-0,005500004
1,00E-07	0,004700005	0,005699992	0,015100002	-0,007400006
1,00E-06	0,004800007	0,001399994	0,009200007	-0,002200007
1,00E-05	0,000699997	-0,003800005	-0,001099989	-0,005500004
1,00E-04	-0,013599992	0,028999999	-0,00559999	-0,017500006

(540nm-690nm)-solvent control E2 reference

cocentration [M]	Replicate 1	Replicate 2	Replicate 3	Replicate 4
1,00E-09	0,3398	0,291799992	0,272400007	0,287199989

Normalization (substance/1 nM E2)

cocentration [M]	Replicate 1	Replicate 2	Replicate 3	Replicate 4	Mean
1,00E-10	11,86%	2,88%	0,40%	0,66%	**3,95%**
1,00E-09	3,74%	1,54%	9,07%	3,20%	**4,39%**
1,00E-08	1,24%	-0,51%	5,03%	-1,92%	**0,96%**
1,00E-07	1,38%	1,95%	5,54%	-2,58%	**1,58%**
1,00E-06	1,41%	0,48%	3,38%	-0,77%	**1,13%**
1,00E-05	0,21%	-1,30%	-0,40%	-1,92%	**-0,85%**
1,00E-04	-4,00%	9,94%	-2,06%	-6,09%	**-0,55%**

yeast-growth/solubility 690nm/solvent control 690nm

cocentration [M]	Replicate 1	Replicate 2	Replicate 3	Replicate 4	Mean
1,00E-10	114,95%	111,10%	90,80%	91,52%	**102,09%**
1,00E-09	108,93%	109,74%	115,01%	96,26%	**107,49%**
1,00E-08	109,41%	101,57%	106,62%	85,78%	**100,84%**
1,00E-07	108,70%	108,11%	108,94%	83,72%	**102,37%**
1,00E-06	103,47%	97,81%	94,89%	83,21%	**94,85%**
1,00E-05	102,57%	100,77%	104,37%	85,16%	**98,22%**
1,00E-04	91,33%	104,04%	91,58%	86,73%	**93,42%**

2nd experient

540nm

cocentration [M]	Replicate 1	Replicate 2	Replicate 3	Replicate 4
1,00E-10	0,880599976	0,719900012	0,692900002	0,656899989
1,00E-09	0,930599988	0,864499986	0,770900011	0,715799987
1,00E-08	0,941200018	0,859099984	0,78549999	0,631699979
1,00E-07	0,938499987	0,981599987	0,807500005	0,610400021
1,00E-06	0,960900009	0,892400026	0,663399994	0,630800009
1,00E-05	0,822399974	0,787800014	0,780399978	0,604200006
1,00E-04	0,671800017	0,616999984	0,601400018	0,723100007

690nm

cocentration [M]	Replicate 1	Replicate 2	Replicate 3	Replicate 4
1,00E-10	0,712499976	0,5898	0,566500008	0,528100014
1,00E-09	0,752900004	0,720399976	0,631799996	0,584999979
1,00E-08	0,775399983	0,716199994	0,644500017	0,509199977
1,00E-07	0,7755	0,83039999	0,662299991	0,489800006
1,00E-06	0,795899987	0,745599985	0,539200008	0,503300011
1,00E-05	0,671199977	0,646200001	0,643000007	0,482800007
1,00E-04	0,518000007	0,475100011	0,466300011	0,564899981

solvent control 690nm

	Replicate 1	Replicate 2	Replicate 3	Replicate 4
	0,61500001	0,598100007	0,540700018	0,537299991

540-690nm

cocentration [M]	Replicate 1	Replicate 2	Replicate 3	Replicate 4
1,00E-10	0,168099999	0,130199999	0,126399994	0,128800005
1,00E-09	0,177699998	0,144099995	0,1391	0,130799994
1,00E-08	0,165800005	0,142800003	0,141100004	0,122500002
1,00E-07	0,163000003	0,151199996	0,145199999	0,1206
1,00E-06	0,165099993	0,146799996	0,124200001	0,127499998
1,00E-05	0,151199996	0,141499996	0,137500003	0,1215
1,00E-04	0,153899997	0,141900003	0,135100007	0,158199996

solvent control 540nm-690nm

	Replicate 1	Replicate 2	Replicate 3	Replicate 4
	0,149000004	0,137799993	0,130999997	0,131999999

(540nm-690nm)-solvent control (540nm-690nm)

cocentration [M]	Replicate 1	Replicate 2	Replicate 3	Replicate 4
1,00E-10	0,019099995	-0,007599995	-0,004600003	-0,003199995
1,00E-09	0,028699994	0,006300002	0,008100003	-0,001200005
1,00E-08	0,016800001	0,00500001	0,010100007	-0,009499997
1,00E-07	0,013999999	0,013400003	0,014200002	-0,011399999
1,00E-06	0,016099989	0,009000003	-0,006799996	-0,004500002
1,00E-05	0,002199993	0,003700003	0,006500006	-0,010499999
1,00E-04	0,004899994	0,00410001	0,00410001	0,026199996

(540nm-690nm)-solvent control E2 reference

cocentration [M]	Replicate 1	Replicate 2	Replicate 3	Replicate 4
1,00E-09	0,273800001	0,321899995	0,327999994	0,322899997

Normalization (substance/1 nM E2)

cocentration [M]	Replicate 1	Replicate 2	Replicate 3	Replicate 4	Mean
1,00E-10	6,98%	-2,36%	-1,40%	-0,99%	**0,56%**
1,00E-09	10,48%	1,96%	2,47%	-0,37%	**3,63%**
1,00E-08	6,14%	1,55%	3,08%	-2,94%	**1,96%**
1,00E-07	5,11%	4,16%	4,33%	-3,53%	**2,52%**
1,00E-06	5,88%	2,80%	-2,07%	-1,39%	**1,30%**
1,00E-05	0,80%	1,15%	1,98%	-3,25%	**0,17%**
1,00E-04	1,79%	1,27%	1,25%	8,11%	**3,11%**

yeast-growth/solubility 690nm/solvent control 690nm

cocentration [M]	Replicate 1	Replicate 2	Replicate 3	Replicate 4	Mean
1,00E-10	115,85%	98,61%	104,77%	98,29%	**104,38%**
1,00E-09	122,42%	120,45%	116,85%	108,88%	**117,15%**
1,00E-08	126,08%	119,75%	119,20%	94,77%	**114,95%**
1,00E-07	126,10%	138,84%	122,49%	91,16%	**119,65%**
1,00E-06	129,41%	124,66%	99,72%	93,67%	**111,87%**
1,00E-05	109,14%	108,04%	118,92%	89,86%	**106,49%**
1,00E-04	84,23%	79,43%	86,24%	105,14%	**88,76%**

3rd experient

540nm

cocentration [M	Replicate 1	Replicate 2	Replicate 3	Replicate 4
1,00E-10	0,914200008	0,828100026	0,682399988	0,843500018
1,00E-09	0,898500025	0,836799979	0,834299982	0,865400016
1,00E-08	0,953400016	0,934899986	0,910300016	0,8477
1,00E-07	0,969600022	0,881600022	0,855499983	0,739099979
1,00E-06	0,939599991	0,93599999	0,908999979	0,815900028
1,00E-05	0,868399978	0,881699979	0,798699975	0,826399982
1,00E-04	0,702700019	0,770799994	0,661199987	0,744799972

690nm

cocentration [M	Replicate 1	Replicate 2	Replicate 3	Replicate 4
1,00E-10	0,727699995	0,682900012	0,557799995	0,69690001
1,00E-09	0,745199978	0,694000006	0,698300004	0,717299998
1,00E-08	0,799899995	0,783100009	0,764999986	0,706099987
1,00E-07	0,811100006	0,732200027	0,711199999	0,601999998
1,00E-06	0,787100017	0,784200013	0,764100015	0,673099995
1,00E-05	0,70660001	0,730199993	0,661800027	0,684800029
1,00E-04	0,577600002	0,629700005	0,551999986	0,621800005

solvent control 690nm

	Replicate 1	Replicate 2	Replicate 3	Replicate 4
	0,597000003	0,607900023	0,449000001	0,521899998

540-690nm

cocentration [M	Replicate 1	Replicate 2	Replicate 3	Replicate 4
1,00E-10	0,186399996	0,145199999	0,124600001	0,146599993
1,00E-09	0,153300002	0,142800003	0,136000007	0,148100004
1,00E-08	0,153500006	0,151800007	0,145199999	0,141499996
1,00E-07	0,158500001	0,149399996	0,144299999	0,137099996
1,00E-06	0,152400002	0,151800007	0,144899994	0,142800003
1,00E-05	0,161899999	0,151500002	0,136899993	0,141599998
1,00E-04	0,125100002	0,141200006	0,109200001	0,123000003

solvent control 540nm-690nm

	Replicate 1	Replicate 2	Replicate 3	Replicate 4
	0,143800005	0,129600003	0,115999997	0,135199994

(540nm-690nm)-solvent control (540nm-690nm)

cocentration [M	Replicate 1	Replicate 2	Replicate 3	Replicate 4
1,00E-10	0,042599991	0,015599996	0,008600004	0,011399999
1,00E-09	0,009499997	0,0132	0,020000011	0,01290001
1,00E-08	0,0097	0,022200003	0,029200003	0,006300002
1,00E-07	0,014699996	0,019799992	0,028300002	0,001900002
1,00E-06	0,008599997	0,022200003	0,028899997	0,007600009
1,00E-05	0,018099993	0,021899998	0,020899996	0,006400004
1,00E-04	-0,018700004	0,011600003	-0,006799996	-0,01219999

(540nm-690nm)-solvent control E2 reference

cocentration [M	Replicate 1	Replicate 2	Replicate 3	Replicate 4
1,00E-09	0,272799984	0,2377	0,280699998	0,259600013

Normalization (substance/1 nM E2)

cocentration [M	Replicate 1	Replicate 2	Replicate 3	Replicate 4	Mean
1,00E-10	15,62%	6,56%	3,06%	4,39%	**7,41%**
1,00E-09	3,48%	5,55%	7,13%	4,97%	**5,28%**
1,00E-08	3,56%	9,34%	10,40%	2,43%	**6,43%**
1,00E-07	5,39%	8,33%	10,08%	0,73%	**6,13%**
1,00E-06	3,15%	9,34%	10,30%	2,93%	**6,43%**
1,00E-05	6,63%	9,21%	7,45%	2,47%	**6,44%**
1,00E-04	-6,85%	4,88%	-2,42%	-4,70%	**-2,27%**

yeast-growth/solubility 690nm/solvent control 690nm

cocentration [M	Replicate 1	Replicate 2	Replicate 3	Replicate 4	Mean
1,00E-10	121,89%	112,34%	124,23%	133,53%	**123,00%**
1,00E-09	124,82%	114,16%	155,52%	137,44%	**132,99%**
1,00E-08	133,99%	128,82%	170,38%	135,29%	**142,12%**
1,00E-07	135,86%	120,45%	158,40%	115,35%	**132,51%**
1,00E-06	131,84%	129,00%	170,18%	128,97%	**140,00%**
1,00E-05	118,36%	120,12%	147,39%	131,21%	**129,27%**
1,00E-04	96,75%	103,59%	122,94%	119,14%	**110,60%**

4th experient

540nm

cocentration [M	Replicate 1	Replicate 2	Replicate 3	Replicate 4
1,00E-10	1,090299964	0,906899989	0,958599985	0,906499982
1,00E-09	1,043400049	0,930299997	0,946699977	0,896899998
1,00E-08	1,00880003	0,93720001	0,935899973	0,929799974
1,00E-07	1,067499995	0,957799971	0,925000012	0,89410001
1,00E-06	0,9903	0,903199971	0,932600021	0,855099976
1,00E-05	0,978399992	1,004600048	0,958999991	0,858500004
1,00E-04	0,915899992	0,910600007	0,880200028	0,866999984

690nm

cocentration [M	Replicate 1	Replicate 2	Replicate 3	Replicate 4
1,00E-10	0,85619998	0,75150001	0,805599988	0,75029999
1,00E-09	0,834500015	0,775099993	0,792100012	0,743499994
1,00E-08	0,735199988	0,771499991	0,780099988	0,77670002
1,00E-07	0,80430001	0,797399998	0,771600008	0,743700027
1,00E-06	0,799499989	0,74970001	0,77759999	0,705900013
1,00E-05	0,802699983	0,850700021	0,809800029	0,709900022
1,00E-04	0,76910001	0,763499975	0,738600016	0,721899986

solvent control 690nm

	Replicate 1	Replicate 2	Replicate 3	Replicate 4
	0,712499976	0,805299997	0,707300007	0,761300027

540-690nm

cocentration [M	Replicate 1	Replicate 2	Replicate 3	Replicate 4
1,00E-10	0,234099996	0,155399993	0,152999997	0,156200007
1,00E-09	0,208900005	0,155200005	0,154699996	0,153400004
1,00E-08	0,273600012	0,165600002	0,155900002	0,153099999
1,00E-07	0,263200015	0,160500005	0,153400004	0,150399998
1,00E-06	0,190799996	0,153500006	0,155000001	0,149200007
1,00E-05	0,175699994	0,153899997	0,149200007	0,148599997
1,00E-04	0,146799996	0,147	0,141599998	0,144999996

solvent control 540nm-690nm

	Replicate 1	Replicate 2	Replicate 3	Replicate 4
	0,146400005	0,153400004	0,143399999	0,150299996

(540nm-690nm)-solvent control (540nm-690nm)

cocentration [M	Replicate 1	Replicate 2	Replicate 3	Replicate 4
1,00E-10	0,08769999	0,00199999	0,0096	0,00590001
1,00E-09	0,0625	0,001800001	0,011299998	0,003100008
1,00E-08	0,127200007	0,012199998	0,012500003	0,002800003
1,00E-07	0,11680001	0,007100001	0,010000005	0,000100002
1,00E-06	0,044399992	0,000100002	0,011600003	-0,001099989
1,00E-05	0,029299989	0,000499994	0,005800009	-0,001699999
1,00E-04	0,000399992	-0,006400004	-0,001800001	-0,0053

(540nm-690nm)-solvent control E2 reference

cocentration [M	Replicate 1	Replicate 2	Replicate 3	Replicate 4
1,00E-09	0,560300022	0,562099993	0,587200001	0,591399997

Normalization (substance/1 nM E2)

cocentration [M	Replicate 1	Replicate 2	Replicate 3	Replicate 4	Mean
1,00E-10	26,13%	27,29%	24,42%	25,41%	**25,81%**
1,00E-09	11,15%	0,32%	1,92%	0,52%	**3,48%**
1,00E-08	22,70%	2,17%	2,13%	0,47%	**6,87%**
1,00E-07	20,85%	1,26%	1,70%	0,02%	**5,96%**
1,00E-06	7,92%	0,02%	1,98%	-0,19%	**2,43%**
1,00E-05	5,23%	0,09%	0,99%	-0,29%	**1,50%**
1,00E-04	0,07%	-1,14%	-0,31%	-0,90%	**-0,57%**

yeast-growth/solubility 690nm/solvent control 690nm

cocentration [M	Replicate 1	Replicate 2	Replicate 3	Replicate 4	Mean
1,00E-10	120,17%	93,32%	113,90%	98,56%	**106,49%**
1,00E-09	117,12%	96,25%	111,99%	97,66%	**105,76%**
1,00E-08	103,19%	95,80%	110,29%	102,02%	**102,83%**
1,00E-07	112,88%	99,02%	109,09%	97,69%	**104,67%**
1,00E-06	112,21%	93,10%	109,94%	92,72%	**101,99%**
1,00E-05	112,66%	105,64%	114,49%	93,25%	**106,51%**
1,00E-04	107,94%	94,81%	104,43%	94,82%	**100,50%**

5th experiment

540nm

cocentration [M	Replicate 1	Replicate 2	Replicate 3	Replicate 4
1,00E-10	1,001399994	0,985199988	1,009600043	0,927100003
1,00E-09	0,967199981	0,969500005	0,961300015	0,940500021
1,00E-08	0,958000004	0,97299999	0,972100019	0,919700027
1,00E-07	1,012099981	0,956099987	1,001000047	0,933099985
1,00E-06	0,870700002	0,888000011	0,858299971	0,930899978
1,00E-05	0,901899993	0,800199986	0,784699976	0,887600005
1,00E-04	0,712199986	0,648599982	0,681800008	0,825600028

690nm

cocentration [M	Replicate 1	Replicate 2	Replicate 3	Replicate 4
1,00E-10	0,841799974	0,835600019	0,860300004	0,7773
1,00E-09	0,813499987	0,821200013	0,814999998	0,792800009
1,00E-08	0,81190002	0,8204	0,823700011	0,777499974
1,00E-07	0,860000014	0,807900012	0,854499996	0,784300029
1,00E-06	0,729700029	0,74970001	0,721700013	0,782500029
1,00E-05	0,759599984	0,668200016	0,651199996	0,743700027
1,00E-04	0,581700027	0,536499977	0,567600012	0,683600008

solvent control 690nm

	Replicate 1	Replicate 2	Replicate 3	Replicate 4
	0,800499976	0,787599981	0,797900021	0,769500017

540-690nm

cocentration [M	Replicate 1	Replicate 2	Replicate 3	Replicate 4
1,00E-10	0,159500003	0,149599999	0,149299994	0,149700001
1,00E-09	0,153699994	0,148200005	0,146300003	0,147699997
1,00E-08	0,1461	0,152500004	0,148399994	0,142199993
1,00E-07	0,152099997	0,148200005	0,146500006	0,148800001
1,00E-06	0,140900001	0,138300002	0,136500001	0,148399994
1,00E-05	0,142299995	0,131999999	0,133499995	0,143900007
1,00E-04	0,130500004	0,112099998	0,114200003	0,142000005

solvent control 540nm-690nm

	Replicate 1	Replicate 2	Replicate 3	Replicate 4
	0,154400006	0,151899993	0,151600003	0,147499993

(540nm-690nm)-solvent control (540nm-690nm)

cocentration [M	Replicate 1	Replicate 2	Replicate 3	Replicate 4
1,00E-10	0,005099997	-0,002299994	-0,002300009	0,002200007
1,00E-09	-0,000700012	-0,003699988	-0,0053	0,000200003
1,00E-08	-0,008300006	0,00060001	-0,003200009	-0,0053
1,00E-07	-0,002300009	-0,003699988	-0,005099997	0,001300007
1,00E-06	-0,013500005	-0,013599992	-0,015100002	0,0009
1,00E-05	-0,012100011	-0,019899994	-0,018100008	-0,003599986
1,00E-04	-0,023900002	-0,039799996	-0,0374	-0,005499989

(540nm-690nm)-solvent control E2 reference

cocentration [M	Replicate 1	Replicate 2	Replicate 3	Replicate 4
1,00E-09	0,474000022	0,47420001	0,477100015	0,466400012

Normalization (substance/1 nM E2)

cocentration [M	Replicate 1	Replicate 2	Replicate 3	Replicate 4	Mean
1,00E-10	1,08%	-0,49%	-0,48%	0,47%	**0,15%**
1,00E-09	-0,15%	-0,78%	-1,11%	0,04%	**-0,50%**
1,00E-08	-1,75%	0,13%	-0,67%	-1,14%	**-0,86%**
1,00E-07	-0,49%	-0,78%	-1,07%	0,28%	**-0,51%**
1,00E-06	-2,85%	-2,87%	-3,16%	0,19%	**-2,17%**
1,00E-05	-2,55%	-4,20%	-3,79%	-0,77%	**-2,83%**
1,00E-04	-5,04%	-8,39%	-7,84%	-1,18%	**-5,61%**

yeast-growth/solubility 690nm/solvent control 690nm

cocentration [M	Replicate 1	Replicate 2	Replicate 3	Replicate 4	Mean
1,00E-10	105,16%	106,09%	107,82%	101,01%	**105,02%**
1,00E-09	101,62%	104,27%	102,14%	103,03%	**102,77%**
1,00E-08	101,42%	104,16%	103,23%	101,04%	**102,47%**
1,00E-07	107,43%	102,58%	107,09%	101,92%	**104,76%**
1,00E-06	91,16%	95,19%	90,45%	101,69%	**94,62%**
1,00E-05	94,89%	84,84%	81,61%	96,65%	**89,50%**
1,00E-04	72,67%	68,12%	71,14%	88,84%	**75,19%**

6th experiment

540nm

cocentration [M	Replicate 1	Replicate 2	Replicate 3	Replicate 4
1,00E-10	0,856899977	0,847800016	0,813899994	0,809099972
1,00E-09	0,800300002	0,788900018	0,777100027	0,766499996
1,00E-08	0,829200029	0,889599979	0,847199976	0,776000023
1,00E-07	0,833800018	0,86559999	0,844500005	0,820900023
1,00E-06	0,781400025	0,861999989	0,851100028	0,785300016
1,00E-05	0,872799993	0,896600008	0,832799971	0,77759999
1,00E-04	0,684800029	0,664499998	0,6523	0,684400022

690nm

cocentration [M	Replicate 1	Replicate 2	Replicate 3	Replicate 4
1,00E-10	0,707099974	0,703800023	0,674399972	0,666999996
1,00E-09	0,661199987	0,65109998	0,640100002	0,630100012
1,00E-08	0,680000007	0,743200004	0,703199983	0,638700008
1,00E-07	0,690599978	0,721099973	0,700699985	0,679099977
1,00E-06	0,634299994	0,71509999	0,708599985	0,646200001
1,00E-05	0,725399971	0,748000026	0,690800011	0,640200019
1,00E-04	0,561500013	0,541999996	0,533699989	0,560599983

solvent control 690nm

	Replicate 1	Replicate 2	Replicate 3	Replicate 4
	0,614099979	0,652800024	0,661899984	0,671899974

540-690nm

cocentration [M	Replicate 1	Replicate 2	Replicate 3	Replicate 4
1,00E-10	0,149800003	0,143999994	0,139500007	0,142100006
1,00E-09	0,1391	0,137799993	0,136999995	0,136399999
1,00E-08	0,149200007	0,146400005	0,143999994	0,1373
1,00E-07	0,143299997	0,144500002	0,143800005	0,141800001
1,00E-06	0,147100002	0,146899998	0,142499998	0,1391
1,00E-05	0,147400007	0,148699999	0,142000005	0,1373
1,00E-04	0,123300001	0,122400001	0,118600003	0,1237

solvent control 540nm-690nm

	Replicate 1	Replicate 2	Replicate 3	Replicate 4
	0,136399999	0,139400005	0,139599994	0,140499994

(540nm-690nm)-solvent control (540nm-690nm)

cocentration [M	Replicate 1	Replicate 2	Replicate 3	Replicate 4
1,00E-10	0,013400003	0,004599988	-9,99868E-05	0,001600012
1,00E-09	0,002700001	-0,001600012	-0,002599999	-0,004099995
1,00E-08	0,012800008	0,006999999	0,0044	-0,003199995
1,00E-07	0,006899998	0,005099997	0,004200011	0,001300007
1,00E-06	0,010700002	0,007499993	0,002900004	-0,001399994
1,00E-05	0,011000007	0,009299994	0,002400011	-0,003199995
1,00E-04	-0,013099998	-0,017000005	-0,02099999	-0,016799994

(540nm-690nm)-solvent control E2 reference

cocentration [M	Replicate 1	Replicate 2	Replicate 3	Replicate 4
1,00E-09	0,674400017	0,68690002	0,68490003	0,693200007

Normalization (substance/1 nM E2)

cocentration [M	Replicate 1	Replicate 2	Replicate 3	Replicate 4	Mean
1,00E-10	1,99%	0,67%	-0,01%	0,23%	**0,72%**
1,00E-09	0,40%	-0,23%	-0,38%	-0,59%	**-0,20%**
1,00E-08	1,90%	1,02%	0,64%	-0,46%	**0,77%**
1,00E-07	1,02%	0,74%	0,61%	0,19%	**0,64%**
1,00E-06	1,59%	1,09%	0,42%	-0,20%	**0,72%**
1,00E-05	1,63%	1,35%	0,35%	-0,46%	**0,72%**
1,00E-04	-1,94%	-2,47%	-3,07%	-2,42%	**-2,48%**

yeast-growth/solubility 690nm/solvent control 690nm

cocentration [M	Replicate 1	Replicate 2	Replicate 3	Replicate 4	Mean
1,00E-10	115,14%	107,81%	101,89%	99,27%	**106,03%**
1,00E-09	107,67%	99,74%	96,71%	93,78%	**99,47%**
1,00E-08	110,73%	113,85%	106,24%	95,06%	**106,47%**
1,00E-07	112,46%	110,46%	105,86%	101,07%	**107,46%**
1,00E-06	103,29%	109,54%	107,06%	96,18%	**104,02%**
1,00E-05	118,12%	114,58%	104,37%	95,28%	**108,09%**
1,00E-04	91,43%	83,03%	80,63%	83,44%	**84,63%**

7th experiment

540nm

cocentration [M	Replicate 1	Replicate 2	Replicate 3	Replicate 4
1,00E-10	0,902899981	0,895799994	0,885699987	0,829200029
1,00E-09	0,909799993	0,874199986	0,886900008	0,799199998
1,00E-08	0,915099978	0,924199998	0,863499999	0,818799973
1,00E-07	0,8671	0,818700016	0,904699981	0,843500018
1,00E-06	0,933600008	0,875800014	0,864799976	0,761099994
1,00E-05	0,864700019	0,842999995	0,816600025	0,816999972
1,00E-04	0,7676	0,73150003	0,71390003	0,755699992

690nm

cocentration [M	Replicate 1	Replicate 2	Replicate 3	Replicate 4
1,00E-10	0,733699977	0,738499999	0,727699995	0,673300028
1,00E-09	0,753000021	0,720200002	0,728399992	0,647000015
1,00E-08	0,743700027	0,764800012	0,708700001	0,667800009
1,00E-07	0,713500023	0,672299981	0,748899996	0,688600004
1,00E-06	0,75029999	0,719399989	0,712100029	0,61650002
1,00E-05	0,711700022	0,690500021	0,669200003	0,666400015
1,00E-04	0,623099983	0,592100024	0,5801	0,616699994

solvent control 690nm

	Replicate 1	Replicate 2	Replicate 3	Replicate 4
	0,671999991	0,728200018	0,716600001	0,753400028

540-690nm

cocentration [M	Replicate 1	Replicate 2	Replicate 3	Replicate 4
1,00E-10	0,169200003	0,157299995	0,158099994	0,155900002
1,00E-09	0,156800002	0,153999999	0,158600003	0,1523
1,00E-08	0,171399996	0,159500003	0,154799998	0,150900006
1,00E-07	0,153600007	0,146400005	0,155699998	0,154899999
1,00E-06	0,183300003	0,156399995	0,152799994	0,144600004
1,00E-05	0,152999997	0,152500004	0,147400007	0,1505
1,00E-04	0,144500002	0,139400005	0,1338	0,138999999

solvent control 540nm-690nm

	Replicate 1	Replicate 2	Replicate 3	Replicate 4
	0,159199998	0,162400007	0,155300006	0,159500003

(540nm-690nm)-solvent control (540nm-690nm)

cocentration [M	Replicate 1	Replicate 2	Replicate 3	Replicate 4
1,00E-10	0,010000005	-0,005100012	0,002799988	-0,003600001
1,00E-09	-0,002399996	-0,008400008	0,003299996	-0,007200003
1,00E-08	0,012199998	-0,002900004	-0,000500008	-0,008599997
1,00E-07	-0,00559999	-0,016000003	0,000399992	-0,004600003
1,00E-06	0,024100006	-0,006000012	-0,002500013	-0,014899999
1,00E-05	-0,006200001	-0,009900004	-0,0079	-0,009000003
1,00E-04	-0,014699996	-0,023000002	-0,021500006	-0,020500004

(540nm-690nm)-solvent control E2 reference

cocentration [M	Replicate 1	Replicate 2	Replicate 3	Replicate 4
1,00E-09	0,626299992	0,650099993	0,643300012	0,647899985

Normalization (substance/1 nM E2)

cocentration [M	Replicate 1	Replicate 2	Replicate 3	Replicate 4	Mean
1,00E-10	1,60%	-0,78%	0,44%	-0,56%	**0,17%**
1,00E-09	-0,38%	-1,29%	0,51%	-1,11%	**-0,57%**
1,00E-08	1,95%	-0,45%	-0,08%	-1,33%	**0,02%**
1,00E-07	-0,89%	-2,46%	0,06%	-0,71%	**-1,00%**
1,00E-06	3,85%	-0,92%	-0,39%	-2,30%	**0,06%**
1,00E-05	-0,99%	-1,52%	-1,23%	-1,39%	**-1,28%**
1,00E-04	-2,35%	-3,54%	-3,34%	-3,16%	**-3,10%**

yeast-growth/solubility 690nm/solvent control 690nm

cocentration [M	Replicate 1	Replicate 2	Replicate 3	Replicate 4	Mean
1,00E-10	109,18%	101,41%	101,55%	89,37%	**100,38%**
1,00E-09	112,05%	98,90%	101,65%	85,88%	**99,62%**
1,00E-08	110,67%	105,03%	98,90%	88,64%	**100,81%**
1,00E-07	106,18%	92,32%	104,51%	91,40%	**98,60%**
1,00E-06	111,65%	98,79%	99,37%	81,83%	**97,91%**
1,00E-05	105,91%	94,82%	93,39%	88,45%	**95,64%**
1,00E-04	92,72%	81,31%	80,95%	81,86%	**84,21%**

8th experiment

540nm

cocentration [M	Replicate 1	Replicate 2	Replicate 3	Replicate 4
1,00E-10	0,977999985	0,872300029	0,915300012	0,881399989
1,00E-09	0,895099998	0,922599971	0,938300014	0,917500019
1,00E-08	0,985599995	0,929499984	0,903999984	0,908999979
1,00E-07	0,9921	0,899299979	0,91869998	0,871599972
1,00E-06	0,991900027	0,872200012	0,823800027	0,861199975
1,00E-05	0,825200021	0,856599987	0,854700029	0,868099988
1,00E-04	0,782599986	0,843599975	0,850000024	0,8125

690nm

cocentration [M	Replicate 1	Replicate 2	Replicate 3	Replicate 4
1,00E-10	0,753300011	0,722199976	0,762300014	0,731999993
1,00E-09	0,741900027	0,773699999	0,787500024	0,767400026
1,00E-08	0,751299977	0,768999994	0,754700005	0,758599997
1,00E-07	0,741599977	0,733099997	0,760200024	0,720700026
1,00E-06	0,708400011	0,708100021	0,675499976	0,713199973
1,00E-05	0,680000007	0,710200012	0,711099982	0,720700026
1,00E-04	0,653599977	0,708100021	0,71420002	0,680899978

solvent control 690nm

	Replicate 1	Replicate 2	Replicate 3	Replicate 4
	0,711899996	0,720799983	0,763899982	0,737800002

540-690nm

cocentration [M	Replicate 1	Replicate 2	Replicate 3	Replicate 4
1,00E-10	0,224700004	0,150099993	0,153099999	0,149499997
1,00E-09	0,153099999	0,148900002	0,150800005	0,150099993
1,00E-08	0,234400004	0,160500005	0,149399996	0,150399998
1,00E-07	0,250400007	0,166199997	0,158500001	0,150999993
1,00E-06	0,283600003	0,164100006	0,148200005	0,148000002
1,00E-05	0,145099998	0,146400005	0,143600002	0,147400007
1,00E-04	0,128999993	0,135499999	0,135800004	0,131500006

solvent control 540nm-690nm

	Replicate 1	Replicate 2	Replicate 3	Replicate 4
	0,149900004	0,150600001	0,150800005	0,148900002

(540nm-690nm)-solvent control (540nm-690nm)

cocentration [M	Replicate 1	Replicate 2	Replicate 3	Replicate 4
1,00E-10	0,0748	-0,000500008	0,002299994	0,000599995
1,00E-09	0,003199995	-0,001699999	0	0,001199991
1,00E-08	0,0845	0,009900004	-0,001400009	0,001499996
1,00E-07	0,100500003	0,015599996	0,007699996	0,002099991
1,00E-06	0,133699998	0,013500005	-0,002599999	-0,0009
1,00E-05	-0,004800007	-0,004199997	-0,007200003	-0,001499996
1,00E-04	-0,020900011	-0,015100002	-0,015000001	-0,017399997

(540nm-690nm)-solvent control E2 reference

cocentration [M	Replicate 1	Replicate 2	Replicate 3	Replicate 4
1,00E-09	0,339200005	0,327099994	0,320900008	0,316100001

Normalization (substance/1 nM E2)

cocentration [M	Replicate 1	Replicate 2	Replicate 3	Replicate 4	Mean
1,00E-10	22,05%	-0,15%	0,72%	0,19%	**5,70%**
1,00E-09	0,94%	-0,52%	0,00%	0,38%	**0,20%**
1,00E-08	24,91%	3,03%	-0,44%	0,47%	**6,99%**
1,00E-07	29,63%	4,77%	2,40%	0,66%	**9,37%**
1,00E-06	39,42%	4,13%	-0,81%	-0,28%	**10,61%**
1,00E-05	-1,42%	-1,28%	-2,24%	-0,47%	**-1,35%**
1,00E-04	-6,16%	-4,62%	-4,67%	-5,50%	**-5,24%**

yeast-growth/solubility 690nm/solvent control 690nm

cocentration [M	Replicate 1	Replicate 2	Replicate 3	Replicate 4	Mean
1,00E-10	105,82%	100,19%	99,79%	99,21%	**101,25%**
1,00E-09	104,21%	107,34%	103,09%	104,01%	**104,66%**
1,00E-08	105,53%	106,69%	98,80%	102,82%	**103,46%**
1,00E-07	104,17%	101,71%	99,52%	97,68%	**100,77%**
1,00E-06	99,51%	98,24%	88,43%	96,67%	**95,71%**
1,00E-05	95,52%	98,53%	93,09%	97,68%	**96,20%**
1,00E-04	91,81%	98,24%	93,49%	92,29%	**93,96%**

YES chlorpyrifos

1st experient

540nm

cocentration [M]	Replicate 1	Replicate 2	Replicate 3	Replicate 4
1,00E-07	0,65140003	0,570800006	0,623700023	0,70539999
1,00E-06	0,63380003	0,745599985	0,741400003	0,833899975
1,00E-05	0,850499988	0,912299991	0,870999992	0,805800021
5,00E-05	0,864799976	0,8926	0,840699971	0,81220001
1,00E-04	0,864099979	0,819500029	0,836399972	0,859600008
5,00E-04	0,969500005	0,92839998	0,949299991	0,97390002
1,00E-03	0,991599977	1,013700008	1,021600008	1,004199982

690nm

cocentration [M]	Replicate 1	Replicate 2	Replicate 3	Replicate 4
1,00E-07	0,525699973	0,454400003	0,501399994	0,57889998
1,00E-06	0,503700018	0,611500025	0,607599974	0,694000006
1,00E-05	0,704900026	0,761600018	0,726100028	0,668299973
5,00E-05	0,716499984	0,745800018	0,696200013	0,672599971
1,00E-04	0,717899978	0,672399998	0,694100022	0,715799987
5,00E-04	0,825299978	0,781700015	0,804899991	0,80309999
1,00E-03	0,821500003	0,846700013	0,850199997	0,834100008

solvent control 690nm

	Replicate 1	Replicate 2	Replicate 3	Replicate 4
	0,556299984	0,432999998	0,301299989	0,492900014

540-690nm

cocentration [M]	Replicate 1	Replicate 2	Replicate 3	Replicate 4
1,00E-07	0,125699997	0,116400003	0,122299999	0,126499996
1,00E-06	0,130099997	0,134100005	0,1338	0,139899999
1,00E-05	0,145600006	0,150700003	0,144899994	0,137500003
5,00E-05	0,148200005	0,146799996	0,144500002	0,139500007
1,00E-04	0,146200001	0,147100002	0,142199993	0,143800005
5,00E-04	0,144199997	0,146699995	0,144400001	0,170699999
1,00E-03	0,170000002	0,166999996	0,171499997	0,170000002

solvent control 540nm-690nm

	Replicate 1	Replicate 2	Replicate 3	Replicate 4
	0,126800001	0,114200003	0,097999997	0,124399997

(540nm-690nm)-solvent control (540nm-690nm)

cocentration [M]	Replicate 1	Replicate 2	Replicate 3	Replicate 4
1,00E-07	-0,001100004	0,0022	0,024300002	0,002099998
1,00E-06	0,003299996	0,019900002	0,035800003	0,015500002
1,00E-05	0,018800005	0,036499999	0,046899997	0,013100006
5,00E-05	0,021400005	0,032599993	0,046500005	0,01510001
1,00E-04	0,019400001	0,032899998	0,044199996	0,019400008
5,00E-04	0,017399997	0,032499991	0,046400003	0,046300001
1,00E-03	0,043200001	0,052799992	0,0735	0,045600004

(540nm-690nm)-solvent control E2 reference

cocentration [M]	Replicate 1	Replicate 2	Replicate 3	Replicate 4
1,00E-09	0,432500005	0,401799969	0,412900023	0,42240002

Normalization (substance/1 nM E2)

cocentration [M]	Replicate 1	Replicate 2	Replicate 3	Replicate 4	Mean
1,00E-07	-0,25%	0,55%	5,89%	0,50%	**1,67%**
1,00E-06	0,76%	4,95%	8,67%	3,67%	**4,51%**
1,00E-05	4,35%	9,08%	11,36%	3,10%	**6,97%**
5,00E-05	4,95%	8,11%	11,26%	3,57%	**6,97%**
1,00E-04	4,49%	8,19%	10,70%	4,59%	**6,99%**
5,00E-04	4,02%	8,09%	11,24%	10,96%	**8,58%**
1,00E-03	9,99%	13,14%	17,80%	10,80%	**12,93%**

yeast-growth/solubility 690nm/solvent control 690nm

cocentration [M]	Replicate 1	Replicate 2	Replicate 3	Replicate 4	Mean
1,00E-07	94,50%	104,94%	166,41%	117,45%	**120,83%**
1,00E-06	90,54%	141,22%	201,66%	140,80%	**143,56%**
1,00E-05	126,71%	175,89%	240,99%	135,59%	**169,79%**
5,00E-05	128,80%	172,24%	231,07%	136,46%	**167,14%**
1,00E-04	148,36%	180,53%	267,14%	162,93%	**189,74%**
5,00E-04	129,05%	155,29%	230,37%	145,22%	**164,98%**
1,00E-03	147,67%	195,54%	282,18%	169,22%	**198,65%**

2nd experient

540nm

cocentration [M]	Replicate 1	Replicate 2	Replicate 3	Replicate 4
1,00E-07	0,797599971	0,702600002	0,693599999	0,640200019
1,00E-06	0,739899993	0,766499996	0,70480001	0,610499978
1,00E-05	0,839600027	0,819299996	0,783900023	0,768999994
5,00E-05	0,842499971	0,854600012	0,960900009	0,824899971
1,00E-04	0,835600019	0,814800024	0,902100027	0,798099995
5,00E-04	0,981700003	1,004199982	0,971700013	0,944000006
1,00E-03	1,013499975	0,995199978	1,009799957	0,969200015

690nm

cocentration [M]	Replicate 1	Replicate 2	Replicate 3	Replicate 4
1,00E-07	0,657599986	0,566799998	0,56129998	0,520600021
1,00E-06	0,605400026	0,628300011	0,569100022	0,490700006
1,00E-05	0,698300004	0,677699983	0,640999973	0,635399997
5,00E-05	0,698899984	0,71509999	0,8204	0,682200015
1,00E-04	0,694299996	0,671400011	0,761200011	0,657199979
5,00E-04	0,836700022	0,856299996	0,829299986	0,800700009
1,00E-03	0,835500002	0,818099976	0,832700014	0,791100025

solvent control 690nm

	Replicate 1	Replicate 2	Replicate 3	Replicate 4
	0,491400003	0,569100022	0,453299999	0,4912

540-690nm

cocentration [M]	Replicate 1	Replicate 2	Replicate 3	Replicate 4
1,00E-07	0,140000001	0,135800004	0,132300004	0,119599998
1,00E-06	0,134499997	0,1382	0,135700002	0,119900003
1,00E-05	0,141299993	0,1417	0,142800003	0,133599997
5,00E-05	0,143600002	0,139500007	0,140499994	0,142700002
1,00E-04	0,141299993	0,143399999	0,140900001	0,140900001
5,00E-04	0,144999996	0,1479	0,142399997	0,143299997
1,00E-03	0,178000003	0,177100003	0,177100003	0,178100005

solvent control 540nm-690nm

	Replicate 1	Replicate 2	Replicate 3	Replicate 4
	0,128000006	0,131699994	0,122900002	0,120899998

(540nm-690nm)-solvent control (540nm-690nm)

cocentration [M]	Replicate 1	Replicate 2	Replicate 3	Replicate 4
1,00E-07	0,011999995	0,00410001	0,009400003	-0,0013
1,00E-06	0,006499991	0,006500006	0,012800001	-0,000999995
1,00E-05	0,013299987	0,010000005	0,019900002	0,012699999
5,00E-05	0,015599996	0,007800013	0,017599992	0,021800004
1,00E-04	0,013299987	0,011700004	0,017999999	0,020000003
5,00E-04	0,01699999	0,016200006	0,019499995	0,022399999
1,00E-03	0,049999997	0,045400009	0,054200001	0,057200007

(540nm-690nm)-solvent control E2 reference

cocentration [M]	Replicate 1	Replicate 2	Replicate 3	Replicate 4
1,00E-09	0,323400006	0,306000009	0,330699988	0,313099988

Normalization (substance/1 nM E2)

cocentration [M]	Replicate 1	Replicate 2	Replicate 3	Replicate 4	Mean
1,00E-07	3,71%	1,34%	2,84%	-0,42%	**1,87%**
1,00E-06	2,01%	2,12%	3,87%	-0,32%	**1,92%**
1,00E-05	4,11%	3,27%	6,02%	4,06%	**4,36%**
5,00E-05	4,82%	2,55%	5,32%	6,96%	**4,91%**
1,00E-04	4,11%	3,82%	5,44%	6,39%	**4,94%**
5,00E-04	5,26%	5,29%	5,90%	7,15%	**5,90%**
1,00E-03	15,46%	14,84%	16,39%	18,27%	**16,24%**

yeast-growth/solubility 690nm/solvent control 690nm

cocentration [M]	Replicate 1	Replicate 2	Replicate 3	Replicate 4	Mean
1,00E-07	133,82%	99,60%	123,83%	105,99%	**115,81%**
1,00E-06	123,20%	110,40%	125,55%	99,90%	**114,76%**
1,00E-05	142,10%	119,08%	141,41%	129,36%	**132,99%**
5,00E-05	142,23%	125,65%	180,98%	138,88%	**146,94%**
1,00E-04	141,29%	117,98%	167,92%	133,79%	**140,25%**
5,00E-04	170,27%	150,47%	182,95%	163,01%	**166,67%**
1,00E-03	170,02%	143,75%	183,70%	161,05%	**164,63%**

3rd experiment

540nm

cocentration [M	Replicate 1	Replicate 2	Replicate 3	Replicate 4
1,00E-07	0,691100001	0,669799984	0,766799986	0,787100017
1,00E-06	0,666999996	0,799099982	0,730400026	0,780099988
1,00E-05	0,913299978	0,821200013	0,811200023	0,781899989
5,00E-05	0,867299974	0,87650001	0,867200017	0,888499975
1,00E-04	0,872500002	0,891200006	0,87620002	0,89319998
5,00E-04	0,877399981	0,893000007	0,865999997	0,870299995
1,00E-03	0,98210001	0,875400007	0,870999992	0,926199973

690nm

cocentration [M	Replicate 1	Replicate 2	Replicate 3	Replicate 4
1,00E-07	0,58160001	0,566799998	0,654500008	0,675999999
1,00E-06	0,559700012	0,683000028	0,619700015	0,664499998
1,00E-05	0,793500006	0,704200029	0,693099976	0,665099978
5,00E-05	0,745899975	0,753400028	0,742299974	0,75999999
1,00E-04	0,749199986	0,762300014	0,753400028	0,763700008
5,00E-04	0,736599982	0,748899996	0,721199989	0,728699982
1,00E-03	0,810299993	0,72359997	0,746100008	0,781300008

solvent control 690nm

	Replicate 1	Replicate 2	Replicate 3	Replicate 4
	0,552600026	0,572399974	0,594099998	0,573499978

540-690nm

cocentration [M	Replicate 1	Replicate 2	Replicate 3	Replicate 4
1,00E-07	0,1096	0,103	0,112300001	0,111100003
1,00E-06	0,107199997	0,115999997	0,110600002	0,115500003
1,00E-05	0,119800001	0,116999997	0,118000001	0,116800003
5,00E-05	0,121399999	0,123099998	0,124899998	0,1285
1,00E-04	0,123300001	0,128800005	0,122699998	0,129600003
5,00E-04	0,140799999	0,144099995	0,144800007	0,141499996
1,00E-03	0,171800002	0,151800007	0,124899998	0,144899994

solvent control 540nm-690nm

	Replicate 1	Replicate 2	Replicate 3	Replicate 4
	0,0973	0,104599997	0,1074	0,1052

(540nm-690nm)-solvent control (540nm-690nm)

cocentration [M	Replicate 1	Replicate 2	Replicate 3	Replicate 4
1,00E-07	0,0123	-0,001599997	0,004900001	0,005900003
1,00E-06	0,009899996	0,011399999	0,003200002	0,010300003
1,00E-05	0,022500001	0,012400001	0,010600001	0,011600003
5,00E-05	0,024099998	0,0185	0,017499998	0,0233
1,00E-04	0,026000001	0,024200007	0,015299998	0,024400003
5,00E-04	0,043499999	0,039499998	0,037400007	0,036299996
1,00E-03	0,074500002	0,047200009	0,017499998	0,039699994

(540nm-690nm)-solvent control E2 reference

cocentration [M	Replicate 1	Replicate 2	Replicate 3	Replicate 4
1,00E-09	0,587700002	0,401399985	0,414099991	0,434400015

Normalization (substance/1 nM E2)

cocentration [M	Replicate 1	Replicate 2	Replicate 3	Replicate 4	Mean
1,00E-07	2,09%	-0,40%	1,18%	1,36%	**1,06%**
1,00E-06	1,68%	2,84%	0,77%	2,37%	**1,92%**
1,00E-05	3,83%	3,09%	2,56%	2,67%	**3,04%**
5,00E-05	4,10%	4,61%	4,23%	5,36%	**4,57%**
1,00E-04	4,42%	6,03%	3,69%	5,62%	**4,94%**
5,00E-04	7,40%	9,84%	9,03%	8,36%	**8,66%**
1,00E-03	12,68%	11,76%	4,23%	9,14%	**9,45%**

yeast-growth/solubility 690nm/solvent control 690nm

cocentration [M	Replicate 1	Replicate 2	Replicate 3	Replicate 4	Mean
1,00E-07	105,25%	99,02%	110,17%	117,87%	**108,08%**
1,00E-06	101,28%	119,32%	104,31%	115,87%	**110,20%**
1,00E-05	143,59%	123,03%	116,66%	115,97%	**124,81%**
5,00E-05	134,98%	131,62%	124,95%	132,52%	**131,02%**
1,00E-04	135,58%	133,18%	126,81%	133,16%	**132,18%**
5,00E-04	133,30%	130,84%	121,39%	127,06%	**128,15%**
1,00E-03	146,63%	126,42%	125,58%	136,23%	**133,72%**

4th experiment

540nm

cocentration [M	Replicate 1	Replicate 2	Replicate 3	Replicate 4
1,00E-07	0,842299998	0,860599995	0,843500018	0,753099978
1,00E-06	0,810299993	0,790799975	0,761500001	0,773999989
1,00E-05	0,937900007	0,793500006	0,9199	0,807099998
5,00E-05	0,947399974	0,949800014	0,928600013	0,899900019
1,00E-04	0,968800008	0,949800014	0,917400002	0,939700007
5,00E-04	0,921899974	0,961700022	0,915499985	0,891900003
1,00E-03	0,921999991	0,921400011	0,935599983	0,967700005

690nm

cocentration [M	Replicate 1	Replicate 2	Replicate 3	Replicate 4
1,00E-07	0,734899998	0,747399986	0,735099971	0,637199998
1,00E-06	0,704200029	0,690299988	0,658500016	0,672999978
1,00E-05	0,825399995	0,689499974	0,805800021	0,699500024
5,00E-05	0,829699993	0,830900013	0,809000015	0,776899993
1,00E-04	0,841099977	0,825100005	0,796500027	0,79460001
5,00E-04	0,788100004	0,823499978	0,779600024	0,757600009
1,00E-03	0,781700015	0,779699981	0,791700006	0,819800019

solvent control 690nm

	Replicate 1	Replicate 2	Replicate 3	Replicate 4
	0,684000015	0,71450001	0,658100009	0,666400015

540-690nm

cocentration [M	Replicate 1	Replicate 2	Replicate 3	Replicate 4
1,00E-07	0,107299998	0,1131	0,108400002	0,115900002
1,00E-06	0,1061	0,100599997	0,103	0,101000004
1,00E-05	0,112499997	0,104000002	0,114100002	0,107600003
5,00E-05	0,117600001	0,119000003	0,119599998	0,123000003
1,00E-04	0,127800003	0,124600001	0,120899998	0,145099998
5,00E-04	0,1338	0,1382	0,136000007	0,134200007
1,00E-03	0,140300006	0,1417	0,143800005	0,1479

solvent control 540nm-690nm

	Replicate 1	Replicate 2	Replicate 3	Replicate 4
	0,103600003	0,105499998	0,099699996	0,1008

(540nm-690nm)-solvent control (540nm-690nm)

cocentration [M	Replicate 1	Replicate 2	Replicate 3	Replicate 4
1,00E-07	0,003699996	0,007600002	0,008700006	0,015100002
1,00E-06	0,002499998	-0,004900001	0,003300004	0,000200003
1,00E-05	0,008899994	-0,001499996	0,014400005	0,006800003
5,00E-05	0,013999999	0,013500005	0,019900002	0,022200003
1,00E-04	0,0242	0,019100003	0,021200001	0,044299997
5,00E-04	0,030199997	0,032700002	0,036300011	0,033400007
1,00E-03	0,036700003	0,036200002	0,044100009	0,0471

(540nm-690nm)-solvent control E2 reference

cocentration [M	Replicate 1	Replicate 2	Replicate 3	Replicate 4
1,00E-09	0,51979997	0,531299993	0,54239998	0,55270002

Normalization (substance/1 nM E2)

cocentration [M	Replicate 1	Replicate 2	Replicate 3	Replicate 4	Mean
1,00E-07	0,71%	1,43%	1,60%	2,73%	**1,62%**
1,00E-06	0,48%	-0,92%	0,61%	0,04%	**0,05%**
1,00E-05	1,71%	-0,28%	2,65%	1,23%	**1,33%**
5,00E-05	2,69%	2,54%	3,67%	4,02%	**3,23%**
1,00E-04	4,66%	3,59%	3,91%	8,02%	**5,04%**
5,00E-04	5,81%	6,15%	6,69%	6,04%	**6,18%**
1,00E-03	7,06%	6,81%	8,13%	8,52%	**7,63%**

yeast-growth/solubility 690nm/solvent control 690nm

cocentration [M	Replicate 1	Replicate 2	Replicate 3	Replicate 4	Mean
1,00E-07	107,44%	104,60%	111,70%	95,62%	**104,84%**
1,00E-06	102,95%	96,61%	100,06%	100,99%	**100,15%**
1,00E-05	120,67%	96,50%	122,44%	104,97%	**111,15%**
5,00E-05	121,30%	116,29%	122,93%	116,58%	**119,28%**
1,00E-04	122,97%	115,48%	121,03%	119,24%	**119,68%**
5,00E-04	115,22%	115,26%	118,46%	113,69%	**115,66%**
1,00E-03	114,28%	109,13%	120,30%	123,02%	**116,68%**

5th experiment

540nm

cocentration [M	Replicate 1	Replicate 2	Replicate 3	Replicate 4
1,00E-07	0,771300018	0,780200005	0,757300019	0,778400004
1,00E-06	0,768599987	0,774200022	0,749199986	0,711799979
1,00E-05	0,757200003	0,72359997	0,755200028	0,788399994
5,00E-05	0,811999977	0,814700007	0,860099971	0,882300019
1,00E-04	0,862299979	0,8829	0,894599974	0,874599993
5,00E-04	0,877699971	0,872300029	0,881200016	0,878700018
1,00E-03	0,913600028	0,909200013	0,90170002	0,91960001

690nm

cocentration [M	Replicate 1	Replicate 2	Replicate 3	Replicate 4
1,00E-07	0,663200021	0,674799979	0,649399996	0,666800022
1,00E-06	0,657100022	0,662800014	0,639900029	0,605599999
1,00E-05	0,645500004	0,615800023	0,645399988	0,675899982
5,00E-05	0,694100022	0,696500003	0,741900027	0,762700021
1,00E-04	0,727699995	0,7597	0,773400009	0,755500019
5,00E-04	0,732200027	0,731400013	0,740700006	0,735899985
1,00E-03	0,766700029	0,760399997	0,751399994	0,76880002

solvent control 690nm

	Replicate 1	Replicate 2	Replicate 3	Replicate 4
	0,638499975	0,62470001	0,653800011	0,65200001

540-690nm

cocentration [M	Replicate 1	Replicate 2	Replicate 3	Replicate 4
1,00E-07	0,108099997	0,105400003	0,108000003	0,111699998
1,00E-06	0,111500002	0,111400001	0,109300002	0,106200002
1,00E-05	0,111699998	0,107799999	0,109800003	0,112599999
5,00E-05	0,117899999	0,118199997	0,118199997	0,119499996
1,00E-04	0,134499997	0,123199999	0,121200003	0,119000003
5,00E-04	0,145500004	0,140900001	0,140499994	0,142800003
1,00E-03	0,147	0,148800001	0,150299996	0,150900006

solvent control 540nm-690nm

	Replicate 1	Replicate 2	Replicate 3	Replicate 4
	0,107600003	0,107100002	0,109800003	0,107799999

(540nm-690nm)-solvent control (540nm-690nm)

cocentration [M	Replicate 1	Replicate 2	Replicate 3	Replicate 4
1,00E-07	0,000499994	-0,001699999	-0,001800001	0,003899999
1,00E-06	0,003899999	0,004299998	-0,000500001	-0,001599997
1,00E-05	0,004099995	0,000699997	0	0,004799999
5,00E-05	0,010299996	0,011099994	0,008399993	0,011699997
1,00E-04	0,026899993	0,016099997	0,011399999	0,011200003
5,00E-04	0,037900001	0,033799998	0,030699991	0,035000004
1,00E-03	0,039399996	0,041699998	0,040499993	0,043100007

(540nm-690nm)-solvent control E2 reference

cocentration [M	Replicate 1	Replicate 2	Replicate 3	Replicate 4
1,00E-09	0,512500018	0,546300001	0,527200021	0,555800028

Normalization (substance/1 nM E2)

cocentration [M	Replicate 1	Replicate 2	Replicate 3	Replicate 4	**Mean**
1,00E-07	0,10%	-0,31%	-0,34%	0,70%	**0,04%**
1,00E-06	0,76%	0,79%	-0,09%	-0,29%	**0,29%**
1,00E-05	0,80%	0,13%	0,00%	0,86%	**0,45%**
5,00E-05	2,01%	2,03%	1,59%	2,11%	**1,93%**
1,00E-04	5,25%	2,95%	2,16%	2,02%	**3,09%**
5,00E-04	7,40%	6,19%	5,82%	6,30%	**6,43%**
1,00E-03	7,69%	7,63%	7,68%	7,75%	**7,69%**

yeast-growth/solubility 690nm/solvent control 690nm

cocentration [M	Replicate 1	Replicate 2	Replicate 3	Replicate 4	**Mean**
1,00E-07	103,87%	108,02%	99,33%	102,27%	**103,37%**
1,00E-06	102,91%	106,10%	97,87%	92,88%	**99,94%**
1,00E-05	101,10%	98,58%	98,72%	103,67%	**100,51%**
5,00E-05	108,71%	111,49%	113,48%	116,98%	**112,66%**
1,00E-04	113,97%	121,61%	118,29%	115,87%	**117,44%**
5,00E-04	114,68%	117,08%	113,29%	112,87%	**114,48%**
1,00E-03	120,08%	121,72%	114,93%	117,91%	**118,66%**

cell-free 690nm

cocentration [M	Replicate 1	Replicate 2	Replicate 3	Replicate 4
1,00E-07	0,0374	0,037	0,036699999	0,040100001
1,00E-06	0,036800001	0,036699999	0,037900001	0,037999999
1,00E-05	0,037	0,0363	0,0372	0,037700001
5,00E-05	0,044500001	0,0438	0,044799998	0,044399999
1,00E-04	0,050700001	0,049899999	0,052299999	0,056000002
5,00E-04	0,1193	0,132599995	0,169200003	0,105999999
1,00E-03	0,123899996	0,152199998	0,177900001	0,149100006

cell-free solvent control 690nm

cocentration [M	Replicate 1	Replicate 2	Replicate 3	Replicate 4
	0,037900001	0,036600001	0,036699999	0,036699999

cell-free (substance/solvent control)

cocentration [M	Replicate 1	Replicate 2	Replicate 3	Replicate 4	**Mean**
1,00E-07	98,68%	101,09%	100,00%	109,26%	**102,26%**
1,00E-06	97,10%	100,27%	103,27%	103,54%	**101,05%**
1,00E-05	97,63%	99,18%	101,36%	102,72%	**100,22%**
5,00E-05	117,41%	119,67%	122,07%	120,98%	**120,03%**
1,00E-04	133,77%	136,34%	142,51%	152,59%	**141,30%**
5,00E-04	314,78%	362,30%	461,04%	288,83%	**356,73%**
1,00E-03	326,91%	415,85%	484,74%	406,27%	**408,44%**

YES fenarimol

1st experiment

540nm

cocentration [M]	Replicate 1	Replicate 2	Replicate 3	Replicate 4
1,00E-06	0,64319998	0,648000002	0,647899985	0,663900018
5,00E-06	0,631500006	0,605400026	0,597599983	0,671800017
1,00E-05	0,548600018	0,657599986	0,651000023	0,675700009
5,00E-05	0,931299984	1,180999994	1,116600037	1,021100044
1,00E-04	1,42990005	1,412199974	1,470600009	1,613000035
5,00E-04	0,959399998	0,929099977	0,958400011	0,959800005
1,00E-03	1,17750001	1,202499986	1,278599977	1,2227

690nm

cocentration [M]	Replicate 1	Replicate 2	Replicate 3	Replicate 4
1,00E-06	0,512499988	0,521000028	0,51730001	0,535600007
5,00E-06	0,50029999	0,477299988	0,471799999	0,541299999
1,00E-05	0,427300006	0,51849997	0,514400005	0,538399994
5,00E-05	0,50059998	0,541299999	0,576900005	0,537199974
1,00E-04	0,408300012	0,397599995	0,4287	0,471799999
5,00E-04	0,289000005	0,255899996	0,323399991	0,304899991
1,00E-03	0,495900005	0,519200027	0,595099986	0,540000021

solvent control 690nm

	Replicate 1	Replicate 2	Replicate 3	Replicate 4
	0,460200012	0,503899992	0,412800014	0,508899987

540-690nm

cocentration [M]	Replicate 1	Replicate 2	Replicate 3	Replicate 4
1,00E-06	0,130600005	0,127100006	0,130600005	0,128299996
5,00E-06	0,131099999	0,128099993	0,125799999	0,130400002
1,00E-05	0,121299997	0,1391	0,136600003	0,137400001
5,00E-05	0,430700004	0,639800012	0,539699972	0,483900011
1,00E-04	1,021600008	1,014700055	1,041900039	1,141199946
5,00E-04	0,670400023	0,673300028	0,63499999	0,654999971
1,00E-03	0,681599975	0,683300018	0,683600008	0,682699978

solvent control 540nm-690nm

	Replicate 1	Replicate 2	Replicate 3	Replicate 4
	0,121799998	0,125100002	0,115900002	0,124600001

(540nm-690nm)-solvent control (540nm-690nm)

cocentration [M]	Replicate 1	Replicate 2	Replicate 3	Replicate 4
1,00E-06	0,008800007	0,002000004	0,014700003	0,003699996
5,00E-06	0,009300001	0,002999991	0,009899996	0,005800001
1,00E-05	-0,000500001	0,013999999	0,0207	0,012800001
5,00E-05	0,308900006	0,51470001	0,423799969	0,35930001
1,00E-04	0,89980001	0,889600053	0,926000036	1,016599946
5,00E-04	0,548600025	0,548200026	0,519099988	0,530399971
1,00E-03	0,559799977	0,558200017	0,567700006	0,558099978

(540nm-690nm)-solvent control E2 reference

cocentration [M]	Replicate 1	Replicate 2	Replicate 3	Replicate 4
1,00E-09	0,408400007	0,401200011	0,419999994	0,417299978

Normalization (substance/1 nM E2)

cocentration [M]	Replicate 1	Replicate 2	Replicate 3	Replicate 4	Mean
1,00E-06	2,15%	0,50%	3,50%	0,89%	**1,76%**
5,00E-06	2,28%	0,75%	2,36%	1,39%	**1,69%**
1,00E-05	-0,12%	3,49%	4,93%	3,07%	**2,84%**
5,00E-05	75,64%	128,29%	100,90%	86,10%	**97,73%**
1,00E-04	220,32%	221,73%	220,48%	243,61%	**226,54%**
5,00E-04	134,33%	136,64%	123,60%	127,10%	**130,42%**
1,00E-03	137,07%	139,13%	135,17%	133,74%	**136,28%**

yeast-growth/solubility 690nm/solvent control 690nm

cocentration [M]	Replicate 1	Replicate 2	Replicate 3	Replicate 4	Mean
1,00E-06	111,36%	103,39%	125,31%	105,25%	**111,33%**
5,00E-06	108,71%	94,72%	114,29%	106,37%	**106,02%**
1,00E-05	92,85%	102,90%	124,61%	105,80%	**106,54%**
5,00E-05	108,78%	107,42%	139,75%	105,56%	**115,38%**
1,00E-04	62,80%	50,78%	78,34%	59,91%	**62,96%**
5,00E-04	88,72%	78,90%	103,85%	92,71%	**91,05%**
1,00E-03	107,76%	103,04%	144,16%	106,11%	**115,27%**

excluded

2nd experient

540nm

cocentration [M]	Replicate 1	Replicate 2	Replicate 3	Replicate 4
1,00E-06	0,654699981	0,650900006	0,656799972	0,629400015
5,00E-06	0,650099993	0,614199996	0,6778	0,603600025
1,00E-05	0,649299979	0,509500027	0,626299977	0,705299973
5,00E-05	1,243800044	1,113899946	1,305799961	1,288499951
1,00E-04	1,593999982	1,542700052	1,511800051	1,616600037
5,00E-04	0,857599974	0,853799999	0,944199979	0,907899976
1,00E-03	0,995700002	1,023000002	0,993499994	0,99849999

690nm

cocentration [M]	Replicate 1	Replicate 2	Replicate 3	Replicate 4
1,00E-06	0,522499979	0,520900011	0,527899981	0,502399981
5,00E-06	0,521600008	0,488999993	0,545300007	0,478899986
1,00E-05	0,51789999	0,394400001	0,497599989	0,570200026
5,00E-05	0,514400005	0,514299989	0,538100004	0,500500023
1,00E-04	0,441799998	0,436300009	0,419999987	0,412200004
5,00E-04	0,305799991	0,271200001	0,351000011	0,310200006
1,00E-03	0,41080001	0,470299989	0,437400013	0,429199994

solvent control 690nm

	Replicate 1	Replicate 2	Replicate 3	Replicate 4
	0,473399997	0,492500007	0,510200024	0,525600016

540-690nm

cocentration [M]	Replicate 1	Replicate 2	Replicate 3	Replicate 4
1,00E-06	0,132200003	0,129999995	0,128900006	0,127000004
5,00E-06	0,1285	0,125200003	0,132400006	0,124700002
1,00E-05	0,131500006	0,115099996	0,128800005	0,135100007
5,00E-05	0,729399979	0,599600017	0,767700016	0,787999988
1,00E-04	1,152099967	1,106400013	1,091799974	1,204300046
5,00E-04	0,551800013	0,582599998	0,593299985	0,5977
1,00E-03	0,584900022	0,552699983	0,556200027	0,569299996

solvent control 540nm-690nm

	Replicate 1	Replicate 2	Replicate 3	Replicate 4
	0,124700002	0,127700001	0,132499993	0,130700007

(540nm-690nm)-solvent control (540nm-690nm)

cocentration [M]	Replicate 1	Replicate 2	Replicate 3	Replicate 4
1,00E-06	0,0075	0,002299994	-0,003599986	-0,003700003
5,00E-06	0,003799997	-0,002499998	-9,99868E-05	-0,006000005
1,00E-05	0,006800003	-0,012600005	-0,003699988	0,0044
5,00E-05	0,604699977	0,471900016	0,635200024	0,657299981
1,00E-04	1,027399965	0,978700012	0,959299982	1,073600039
5,00E-04	0,42710001	0,454899997	0,460799992	0,466999993
1,00E-03	0,460200019	0,424999982	0,423700035	0,438599989

(540nm-690nm)-solvent control E2 reference

cocentration [M]	Replicate 1	Replicate 2	Replicate 3	Replicate 4
1,00E-09	0,348099984	0,324999988	0,328999996	0,350099996

Normalization (substance/1 nM E2)

cocentration [M]	Replicate 1	Replicate 2	Replicate 3	Replicate 4	Mean
1,00E-06	2,15%	0,71%	-1,09%	-1,06%	**0,18%**
5,00E-06	1,09%	-0,77%	-0,03%	-1,71%	**-0,36%**
1,00E-05	1,95%	-3,88%	-1,12%	1,26%	**-0,45%**
5,00E-05	173,71%	145,20%	193,07%	187,75%	**174,93%**
1,00E-04	295,15%	301,14%	291,58%	306,66%	**298,63%**
5,00E-04	122,69%	139,97%	140,06%	133,39%	**134,03%**
1,00E-03	132,20%	130,77%	128,78%	125,28%	**129,26%**

yeast-growth/solubility 690nm/solvent control 690nm

cocentration [M]	Replicate 1	Replicate 2	Replicate 3	Replicate 4	Mean
1,00E-06	110,37%	105,77%	103,47%	95,59%	**103,80%**
5,00E-06	110,18%	99,29%	106,88%	91,11%	**101,87%**
1,00E-05	109,40%	80,08%	97,53%	108,49%	**98,87%**
5,00E-05	108,66%	104,43%	105,47%	95,22%	**103,45%**
1,00E-04	93,32%	88,59%	82,32%	78,42%	**85,66%**
5,00E-04	64,60%	55,07%	68,80%	59,02%	**61,87%**
1,00E-03	86,78%	95,49%	85,73%	81,66%	**87,41%**

3rd experiment

540nm

cocentration [M	Replicate 1	Replicate 2	Replicate 3	Replicate 4
1,00E-06	0,639299989	0,713400006	0,755800009	0,700800002
5,00E-06	0,677999973	0,773599982	0,701300025	0,73180002
1,00E-05	0,728799999	0,686200023	0,657100022	0,66079998
5,00E-05	1,318199992	1,1954	1,280200005	1,376299977
1,00E-04	1,883100033	1,850499988	1,883000016	1,935500026
5,00E-04	0,923699975	0,913200021	0,938000023	0,904500008
1,00E-03	1,040400028	0,939700007	0,995500028	0,986899972

690nm

cocentration [M	Replicate 1	Replicate 2	Replicate 3	Replicate 4
1,00E-06	0,538800001	0,60710001	0,643100023	0,593299985
5,00E-06	0,570500016	0,660099983	0,593500018	0,625899971
1,00E-05	0,620100021	0,576799989	0,552200019	0,553900003
5,00E-05	0,640100002	0,518999994	0,568400025	0,644400001
1,00E-04	0,539399981	0,524600029	0,534300029	0,572600007
5,00E-04	0,246399999	0,224600002	0,277799994	0,229599997
1,00E-03	0,368099988	0,278299987	0,367199987	0,350699991

solvent control 690nm

	Replicate 1	Replicate 2	Replicate 3	Replicate 4
	0,566299975	0,580299973	0,61589998	0,523000002

540-690nm

cocentration [M	Replicate 1	Replicate 2	Replicate 3	Replicate 4
1,00E-06	0,100500003	0,106200002	0,112599999	0,107600003
5,00E-06	0,1074	0,113499999	0,107900001	0,105899997
1,00E-05	0,1087	0,109399997	0,104900002	0,106899993
5,00E-05	0,67809999	0,676400006	0,711799979	0,731899977
1,00E-04	1,343700051	1,325899959	1,34860003	1,363000035
5,00E-04	0,677299976	0,688600004	0,660099983	0,674899995
1,00E-03	0,672200024	0,661499977	0,628300011	0,636300027

solvent control 540nm-690nm

	Replicate 1	Replicate 2	Replicate 3	Replicate 4
	0,104699999	0,106700003	0,110799998	0,098800004

(540nm-690nm)-solvent control (540nm-690nm)

cocentration [M	Replicate 1	Replicate 2	Replicate 3	Replicate 4
1,00E-06	-0,004199997	-0,000500001	0,001800001	0,0088
5,00E-06	0,002700001	0,006799996	-0,002899997	0,007099994
1,00E-05	0,004000001	0,002699994	-0,005899996	0,008099996
5,00E-05	0,573399991	0,569700003	0,600999981	0,633099973
1,00E-04	1,239000052	1,219199955	1,237800032	1,264200032
5,00E-04	0,572599977	0,581900001	0,549299985	0,576099992
1,00E-03	0,567500025	0,554799974	0,517500013	0,537500024

(540nm-690nm)-solvent control E2 reference

cocentration [M	Replicate 1	Replicate 2	Replicate 3	Replicate 4
1,00E-09	0,482899994	0,438600004	0,426799983	0,468699992

Normalization (substance/1 nM E2)

cocentration [M	Replicate 1	Replicate 2	Replicate 3	Replicate 4	Mean
1,00E-06	-0,87%	-0,11%	0,42%	1,88%	**0,33%**
5,00E-06	0,56%	1,55%	-0,68%	1,51%	**0,74%**
1,00E-05	0,83%	0,62%	-1,38%	1,73%	**0,45%**
5,00E-05	118,74%	129,89%	140,82%	135,08%	**131,13%**
1,00E-04	256,57%	277,98%	290,02%	269,72%	**273,57%**
5,00E-04	118,58%	132,67%	128,70%	122,91%	**125,72%**
1,00E-03	117,52%	126,49%	121,25%	114,68%	**119,99%**

yeast-growth/solubility 690nm/solvent control 690nm

cocentration [M	Replicate 1	Replicate 2	Replicate 3	Replicate 4	Mean
1,00E-06	95,14%	104,62%	104,42%	113,44%	**104,41%**
5,00E-06	100,74%	113,75%	96,36%	119,67%	**107,63%**
1,00E-05	109,50%	99,40%	89,66%	105,91%	**101,12%**
5,00E-05	113,03%	89,44%	92,29%	123,21%	**104,49%**
1,00E-04	95,25%	90,40%	86,75%	109,48%	**95,47%**
5,00E-04	43,51%	38,70%	45,10%	43,90%	**42,80%**
1,00E-03	65,00%	47,96%	59,62%	67,06%	**59,91%**

4th experiment

540nm

cocentration [M	Replicate 1	Replicate 2	Replicate 3	Replicate 4
1,00E-06	0,855700016	0,816600025	0,789799988	0,807200015
5,00E-06	0,794799984	0,818899989	0,814400017	0,742299974
1,00E-05	0,805999994	0,792200029	0,760299981	0,730000019
5,00E-05	1,377099991	1,409600019	1,452800035	1,712200046
1,00E-04	1,970600009	1,987699986	2,005899906	1,996999979
5,00E-04	0,906000018	0,933700025	0,934400022	0,964299977
1,00E-03	1,039800048	1,060199976	1,045699954	1,075600028

690nm

cocentration [M	Replicate 1	Replicate 2	Replicate 3	Replicate 4
1,00E-06	0,74849999	0,714399993	0,685199976	0,69599998
5,00E-06	0,694199979	0,715200007	0,706700027	0,641499996
1,00E-05	0,704599977	0,691600025	0,658599973	0,628199995
5,00E-05	0,610000014	0,636500001	0,585900009	0,644800007
1,00E-04	0,588400006	0,546400011	0,535700023	0,528100014
5,00E-04	0,187000006	0,203700006	0,218600005	0,223199993
1,00E-03	0,32190001	0,356999993	0,342700005	0,365500003

solvent control 690nm

	Replicate 1	Replicate 2	Replicate 3	Replicate 4
	0,648000002	0,636200011	0,647300005	0,64230001

540-690nm

cocentration [M	Replicate 1	Replicate 2	Replicate 3	Replicate 4
1,00E-06	0,107100002	0,102200001	0,104599997	0,111199997
5,00E-06	0,100599997	0,103699997	0,107699998	0,1008
1,00E-05	0,101400003	0,100599997	0,1017	0,101800002
5,00E-05	0,767199993	0,773000002	0,866999984	1,067399979
1,00E-04	1,382200003	1,441400051	1,470299959	1,468799949
5,00E-04	0,718999982	0,729900002	0,715900004	0,740999997
1,00E-03	0,717899978	0,703199983	0,702899992	0,710200012

solvent control 540nm-690nm

	Replicate 1	Replicate 2	Replicate 3	Replicate 4
	0,0964	0,0995	0,099399999	0,097999997

(540nm-690nm)-solvent control (540nm-690nm)

cocentration [M	Replicate 1	Replicate 2	Replicate 3	Replicate 4
1,00E-06	0,010700002	0,002700001	0,005199999	0,0132
5,00E-06	0,004199997	0,004199997	0,008299999	0,002800003
1,00E-05	0,005000003	0,001099996	0,002300002	0,003800005
5,00E-05	0,670799993	0,673500001	0,767599985	0,969399981
1,00E-04	1,285800003	1,341900051	1,37089996	1,370799951
5,00E-04	0,622599982	0,630400002	0,616500005	0,642999999
1,00E-03	0,621499978	0,603699982	0,603499994	0,612200014

(540nm-690nm)-solvent control E2 reference

cocentration [M	Replicate 1	Replicate 2	Replicate 3	Replicate 4
1,00E-09	0,53770002	0,519900024	0,550800011	0,51920002

Normalization (substance/1 nM E2)

cocentration [M	Replicate 1	Replicate 2	Replicate 3	Replicate 4	Mean
1,00E-06	1,99%	0,52%	0,94%	2,54%	**1,50%**
5,00E-06	0,78%	0,81%	1,51%	0,54%	**0,91%**
1,00E-05	0,93%	0,21%	0,42%	0,73%	**0,57%**
5,00E-05	124,75%	129,54%	139,36%	186,71%	**145,09%**
1,00E-04	239,13%	258,11%	248,89%	264,02%	**252,54%**
5,00E-04	115,79%	121,25%	111,93%	123,84%	**118,20%**
1,00E-03	115,58%	116,12%	109,57%	117,91%	**114,80%**

yeast-growth/solubility 690nm/solvent control 690nm

cocentration [M	Replicate 1	Replicate 2	Replicate 3	Replicate 4	Mean
1,00E-06	115,51%	112,29%	105,86%	108,36%	**110,50%**
5,00E-06	107,13%	112,42%	109,18%	99,88%	**107,15%**
1,00E-05	108,73%	108,71%	101,75%	97,80%	**104,25%**
5,00E-05	94,14%	100,05%	90,51%	100,39%	**96,27%**
1,00E-04	90,80%	85,88%	82,76%	82,22%	**85,42%**
5,00E-04	28,86%	32,02%	33,77%	34,75%	**32,35%**
1,00E-03	49,68%	56,11%	52,94%	56,90%	**53,91%**

5th experiment

540nm

cocentration [M	Replicate 1	Replicate 2	Replicate 3	Replicate 4
1,00E-06	0,715399981	0,756500006	0,736599982	0,7852
5,00E-06	0,720600009	0,729499996	0,744199991	0,731899977
1,00E-05	0,692600012	0,721700013	0,660399973	0,738799989
5,00E-05	1,386700034	1,284399986	1,375699997	1,463799953
1,00E-04	1,946799994	2,01609993	2,045099974	1,813199997
5,00E-04	0,94569999	0,954200029	0,93870002	0,936999977
1,00E-03	1,016000032	1,011000037	1,074399948	1,051499963

690nm

cocentration [M	Replicate 1	Replicate 2	Replicate 3	Replicate 4
1,00E-06	0,610199988	0,646499991	0,627499998	0,668799996
5,00E-06	0,613900006	0,621699989	0,634299994	0,623700023
1,00E-05	0,584200025	0,612600029	0,557299972	0,625400007
5,00E-05	0,523599982	0,526499987	0,504400015	0,510999978
1,00E-04	0,417699993	0,430900007	0,425900012	0,386200011
5,00E-04	0,204600006	0,225099996	0,218099996	0,184499994
1,00E-03	0,311100006	0,308899999	0,376199991	0,329100013

solvent control 690nm

	Replicate 1	Replicate 2	Replicate 3	Replicate 4
	0,610300004	0,610599995	0,626800001	0,592199981

540-690nm

cocentration [M	Replicate 1	Replicate 2	Replicate 3	Replicate 4
1,00E-06	0,1052	0,109999999	0,109099999	0,116400003
5,00E-06	0,106799997	0,107799999	0,109899998	0,108199999
1,00E-05	0,108400002	0,109200001	0,103100002	0,113399997
5,00E-05	0,863099992	0,7579	0,871299982	0,952799976
1,00E-04	1,529199958	1,585199952	1,619199991	1,427000046
5,00E-04	0,741100013	0,729099989	0,720600009	0,752499998
1,00E-03	0,704900026	0,702099979	0,698199987	0,722299993

solvent control 540nm-690nm

	Replicate 1	Replicate 2	Replicate 3	Replicate 4
	0,102200001	0,104800001	0,102899998	0,103600003

(540nm-690nm)-solvent control (540nm-690nm)

cocentration [M	Replicate 1	Replicate 2	Replicate 3	Replicate 4
1,00E-06	0,002999999	0,005199999	0,006200001	0,012800001
5,00E-06	0,004599996	0,002999999	0,006999999	0,004599996
1,00E-05	0,006200001	0,0044	0,000200003	0,009799995
5,00E-05	0,760899991	0,653099999	0,768399984	0,849199973
1,00E-04	1,426999956	1,480399951	1,516299993	1,323400043
5,00E-04	0,638900012	0,624299988	0,617700011	0,648899995
1,00E-03	0,602700025	0,597299978	0,595299989	0,61869999

(540nm-690nm)-solvent control E2 reference

cocentration [M	Replicate 1	Replicate 2	Replicate 3	Replicate 4
1,00E-09	0,486399978	0,481599972	0,496199995	0,475299977

Normalization (substance/1 nM E2)

cocentration [M	Replicate 1	Replicate 2	Replicate 3	Replicate 4	Mean
1,00E-06	0,62%	1,08%	1,25%	2,69%	**1,41%**
5,00E-06	0,95%	0,62%	1,41%	0,97%	**0,99%**
1,00E-05	1,27%	0,91%	0,04%	2,06%	**1,07%**
5,00E-05	156,44%	135,61%	154,86%	178,67%	**156,39%**
1,00E-04	293,38%	307,39%	305,58%	278,43%	**296,20%**
5,00E-04	131,35%	129,63%	124,49%	136,52%	**130,50%**
1,00E-03	123,91%	124,02%	119,97%	130,17%	**124,52%**

yeast-growth/solubility 690nm/solvent control 690nm

cocentration [M	Replicate 1	Replicate 2	Replicate 3	Replicate 4	Mean
1,00E-06	99,98%	105,88%	100,11%	112,93%	**104,73%**
5,00E-06	100,59%	101,82%	101,20%	105,32%	**102,23%**
1,00E-05	95,72%	100,33%	88,91%	105,61%	**97,64%**
5,00E-05	85,79%	86,23%	80,47%	86,29%	**84,70%**
1,00E-04	68,44%	70,57%	67,95%	65,21%	**68,04%**
5,00E-04	33,52%	36,87%	34,80%	31,16%	**34,09%**
1,00E-03	50,97%	50,59%	60,02%	55,57%	**54,29%**

cell-free 690nm

cocentration [M	Replicate 1	Replicate 2	Replicate 3	Replicate 4
1,00E-06	0,037099998	0,037	0,036699999	0,037
5,00E-06	0,037300002	0,037799999	0,037500001	0,0374
1,00E-05	0,037500001	0,037300002	0,037099998	0,038199998
5,00E-05	0,0385	0,037	0,0396	0,0383
1,00E-04	0,0429	0,040199999	0,0449	0,041000001
5,00E-04	0,2086	0,279399991	0,157399997	0,175400004
1,00E-03	0,431600004	0,506200016	0,462900013	0,3222

cell-free solvent control 690nm

cocentration [M	Replicate 1	Replicate 2	Replicate 3	Replicate 4
	0,0385	0,036600001	0,0374	0,043699998

cell-free (substance/solvent control)

cocentration [M	Replicate 1	Replicate 2	Replicate 3	Replicate 4	Mean
1,00E-06	96,36%	101,09%	98,13%	84,67%	**95,06%**
5,00E-06	96,88%	103,28%	100,27%	85,58%	**96,50%**
1,00E-05	97,40%	101,91%	99,20%	87,41%	**96,48%**
5,00E-05	100,00%	101,09%	105,88%	87,64%	**98,65%**
1,00E-04	111,43%	109,84%	120,05%	93,82%	**108,78%**
5,00E-04	541,82%	763,39%	420,86%	401,37%	**531,86%**
1,00E-03	1121,04%	1383,06%	1237,70%	737,30%	**1119,77%**

YES fludioxonil

1st experiment

540nm

cocentration [M]	Replicate 1	Replicate 2	Replicate 3	Replicate 4
1,00E-07	0,673099995	0,678300023	0,62379998	0,647300005
1,00E-06	0,756600022	0,631299973	0,611000001	0,589600027
1,00E-05	0,528699994	0,609200001	0,598399997	0,564199984
5,00E-05	0,567799985	0,597800016	0,5995	0,612600029
1,00E-04	0,654900014	0,637600005	0,653699994	0,532000005
5,00E-04	0,810500026	0,779699981	0,728900015	0,649800003
1,00E-03	0,947600007	0,851800025	0,898699999	0,828100026

690nm

cocentration [M]	Replicate 1	Replicate 2	Replicate 3	Replicate 4
1,00E-07	0,535600007	0,539200008	0,4912	0,514400005
1,00E-06	0,609300017	0,497599989	0,480699986	0,463699996
1,00E-05	0,380100012	0,448799998	0,443300009	0,411000013
5,00E-05	0,417199999	0,438300014	0,4472	0,456
1,00E-04	0,494800001	0,474799991	0,490399987	0,382299989
5,00E-04	0,617399991	0,592700005	0,552299976	0,480699986
1,00E-03	0,723699987	0,651300013	0,697899997	0,624899983

solvent control 690nm

	Replicate 1	Replicate 2	Replicate 3	Replicate 4
	0,537599981	0,501600027	0,546000004	0,495599985

540-690nm

cocentration [M]	Replicate 1	Replicate 2	Replicate 3	Replicate 4
1,00E-07	0,137500003	0,1391	0,132599995	0,1329
1,00E-06	0,147300005	0,133699998	0,130199999	0,1259
1,00E-05	0,148599997	0,160300002	0,155100003	0,153200001
5,00E-05	0,150600001	0,159500003	0,1523	0,156599998
1,00E-04	0,160099998	0,162799999	0,163299993	0,149700001
5,00E-04	0,193100005	0,187099993	0,176499993	0,169100001
1,00E-03	0,223900005	0,200499997	0,200900003	0,203199998

solvent control 540nm-690nm

	Replicate 1	Replicate 2	Replicate 3	Replicate 4
	0,133499995	0,130199999	0,1373	0,130700007

(540nm-690nm)-solvent control (540nm-690nm)

cocentration [M]	Replicate 1	Replicate 2	Replicate 3	Replicate 4
1,00E-07	0,004000008	0,008900002	-0,004700005	0,002199993
1,00E-06	0,01380001	0,0035	-0,007100001	-0,004800007
1,00E-05	0,015100002	0,030100003	0,017800005	0,022499993
5,00E-05	0,017100006	0,029300004	0,015000001	0,025899991
1,00E-04	0,026600003	0,032600001	0,025999993	0,018999994
5,00E-04	0,059600011	0,056899995	0,039199993	0,038399994
1,00E-03	0,09040001	0,070299998	0,063600004	0,07249999

(540nm-690nm)-solvent control E2 reference

cocentration [M]	Replicate 1	Replicate 2	Replicate 3	Replicate 4
1,00E-09	0,394699976	0,422400028	0,38970001	0,397199973

Normalization (substance/1 nM E2)

cocentration [M]	Replicate 1	Replicate 2	Replicate 3	Replicate 4	Mean
1,00E-07	1,01%	2,11%	-1,21%	0,55%	**0,62%**
1,00E-06	3,50%	0,83%	-1,82%	-1,21%	**0,32%**
1,00E-05	3,83%	7,13%	4,57%	5,66%	**5,30%**
5,00E-05	4,33%	6,94%	3,85%	6,52%	**5,41%**
1,00E-04	6,74%	7,72%	6,67%	4,78%	**6,48%**
5,00E-04	15,10%	13,47%	10,06%	9,67%	**12,07%**
1,00E-03	22,90%	16,64%	16,32%	18,25%	**18,53%**

yeast-growth/solubility 690nm/solvent control 690nm

cocentration [M]	Replicate 1	Replicate 2	Replicate 3	Replicate 4	Mean
1,00E-07	99,63%	107,50%	89,96%	103,79%	**100,22%**
1,00E-06	113,34%	99,20%	88,04%	93,56%	**98,54%**
1,00E-05	70,70%	89,47%	81,19%	82,93%	**81,07%**
5,00E-05	77,60%	87,38%	81,90%	92,01%	**84,72%**
1,00E-04	114,84%	118,16%	101,15%	96,99%	**107,79%**
5,00E-04	92,04%	94,66%	89,82%	77,14%	**88,41%**
1,00E-03	134,62%	129,84%	127,82%	126,09%	**129,59%**

excluded

2nd experiment

540nm

cocentration [M]	Replicate 1	Replicate 2	Replicate 3	Replicate 4
1,00E-07	0,708400011	0,691299975	0,644800007	0,63440001
1,00E-06	0,694800019	0,677999973	0,654500008	0,684199989
1,00E-05	0,604200006	0,610700011	0,569000006	0,707099974
5,00E-05	0,602299988	0,606000006	0,630800009	0,522199988
1,00E-04	0,626800001	0,623899996	0,612600029	0,512600005
5,00E-04	0,715900004	0,694700003	0,736899972	0,699800014
1,00E-03	0,91900003	0,745800018	0,864000022	0,78640002

690nm

cocentration [M]	Replicate 1	Replicate 2	Replicate 3	Replicate 4
1,00E-07	0,568099976	0,556299984	0,513199985	0,504700005
1,00E-06	0,55400002	0,540600002	0,521899998	0,546299994
1,00E-05	0,444700003	0,452499986	0,414200008	0,532400012
5,00E-05	0,441399992	0,44749999	0,4648	0,361099988
1,00E-04	0,458700001	0,459399998	0,44690001	0,352699995
5,00E-04	0,523800015	0,507499993	0,537999988	0,512899995
1,00E-03	0,689300001	0,548900008	0,64410001	0,580299973

solvent control 690nm

	Replicate 1	Replicate 2	Replicate 3	Replicate 4
	0,452600002	0,468800008	0,481700003	0,510900021

540-690nm

cocentration [M]	Replicate 1	Replicate 2	Replicate 3	Replicate 4
1,00E-07	0,140200004	0,135000005	0,131699994	0,129700005
1,00E-06	0,140799999	0,137500003	0,132599995	0,137899995
1,00E-05	0,159500003	0,158199996	0,154899994	0,174799994
5,00E-05	0,160899997	0,158500001	0,166099995	0,160999998
1,00E-04	0,168099996	0,164499998	0,165700004	0,159899995
5,00E-04	0,192100003	0,187199995	0,199000001	0,186900005
1,00E-03	0,229699999	0,196899995	0,219899997	0,206100002

solvent control 540nm-690nm

	Replicate 1	Replicate 2	Replicate 3	Replicate 4
	0,122400001	0,1259	0,129500002	0,127800003

(540nm-690nm)-solvent control (540nm-690nm)

cocentration [M]	Replicate 1	Replicate 2	Replicate 3	Replicate 4
1,00E-07	-0,001100004	0,0022	0,024300002	0,002099998
1,00E-06	0,003299996	0,019900002	0,035800003	0,015500002
1,00E-05	0,018800005	0,036499999	0,046899997	0,013100006
5,00E-05	0,021400005	0,032599993	0,046500005	0,01510001
1,00E-04	0,019400001	0,032899998	0,044199996	0,019400008
5,00E-04	0,017399997	0,032499991	0,046400003	0,046300001
1,00E-03	0,043200001	0,052799992	0,0735	0,045600004

(540nm-690nm)-solvent control E2 reference

cocentration [M]	Replicate 1	Replicate 2	Replicate 3	Replicate 4
1,00E-09	0,432500005	0,401799969	0,412900023	0,42240002

Normalization (substance/1 nM E2)

cocentration [M]	Replicate 1	Replicate 2	Replicate 3	Replicate 4	Mean
1,00E-07	-0,25%	0,55%	5,89%	0,50%	**1,67%**
1,00E-06	0,76%	4,95%	8,67%	3,67%	**4,51%**
1,00E-05	4,35%	9,08%	11,36%	3,10%	**6,97%**
5,00E-05	4,95%	8,11%	11,26%	3,57%	**6,97%**
1,00E-04	4,49%	8,19%	10,70%	4,59%	**6,99%**
5,00E-04	4,02%	8,09%	11,24%	10,96%	**8,58%**
1,00E-03	9,99%	13,14%	17,80%	10,80%	**12,93%**

yeast-growth/solubility 690nm/solvent control 690nm

cocentration [M]	Replicate 1	Replicate 2	Replicate 3	Replicate 4	Mean
1,00E-07	125,52%	118,66%	106,54%	98,79%	**112,38%**
1,00E-06	122,40%	115,32%	108,35%	106,93%	**113,25%**
1,00E-05	98,25%	96,52%	85,99%	104,21%	**96,24%**
5,00E-05	97,53%	95,46%	96,49%	70,68%	**90,04%**
1,00E-04	101,35%	97,99%	92,78%	69,04%	**90,29%**
5,00E-04	115,73%	108,26%	111,69%	100,39%	**109,02%**
1,00E-03	152,30%	117,09%	133,71%	113,58%	**129,17%**

3rd experiment

540nm

cocentration [M	Replicate 1	Replicate 2	Replicate 3	Replicate 4
1,00E-07	0,776199996	0,754000008	0,757499993	0,739099979
1,00E-06	0,765299976	0,719200015	0,807799995	0,745800018
1,00E-05	0,752300024	0,74059999	0,775699973	0,759500027
5,00E-05	0,747900009	0,772199988	0,712899983	0,731599987
1,00E-04	0,75999999	0,761300027	0,739899993	0,749599993
5,00E-04	0,763899982	0,752900004	0,747300029	0,76849997
1,00E-03	0,904699981	0,858299971	0,780900002	0,791700006

690nm

cocentration [M	Replicate 1	Replicate 2	Replicate 3	Replicate 4
1,00E-07	0,66170001	0,641600013	0,643899977	0,627200007
1,00E-06	0,646300018	0,605199993	0,690800011	0,631699979
1,00E-05	0,589100003	0,577099979	0,597199976	0,586600006
5,00E-05	0,584800005	0,602199972	0,546400011	0,558399975
1,00E-04	0,595799983	0,593599975	0,574100018	0,580900013
5,00E-04	0,594699979	0,5801	0,576699972	0,590900004
1,00E-03	0,698300004	0,666999996	0,597599983	0,615999997

solvent control 690nm

	Replicate 1	Replicate 2	Replicate 3	Replicate 4
	0,584699988	0,674799979	0,640200019	0,609600008

540-690nm

cocentration [M	Replicate 1	Replicate 2	Replicate 3	Replicate 4
1,00E-07	0,114600003	0,112300001	0,113600001	0,111900002
1,00E-06	0,119000003	0,114	0,116999999	0,114100002
1,00E-05	0,163200006	0,163499996	0,178399995	0,172900006
5,00E-05	0,163100004	0,170000002	0,166500002	0,173199996
1,00E-04	0,164199993	0,167699993	0,165700004	0,168699995
5,00E-04	0,169200003	0,172800004	0,170599997	0,177599996
1,00E-03	0,206400007	0,191300005	0,183300003	0,175699994

solvent control 540nm-690nm

	Replicate 1	Replicate 2	Replicate 3	Replicate 4
	0,107600003	0,113799997	0,109999999	0,110699996

(540nm-690nm)-solvent control (540nm-690nm)

cocentration [M	Replicate 1	Replicate 2	Replicate 3	Replicate 4
1,00E-07	0,006999999	-0,001499996	0,003600001	0,001200005
1,00E-06	0,011399999	0,000200003	0,006999999	0,003400005
1,00E-05	0,055600002	0,049699999	0,068399996	0,06220001
5,00E-05	0,055500001	0,056200005	0,056500003	0,0625
1,00E-04	0,05659999	0,053899996	0,055700004	0,057999998
5,00E-04	0,0616	0,059000008	0,060599998	0,0669
1,00E-03	0,098800004	0,077500008	0,073300004	0,064999998

(540nm-690nm)-solvent control E2 reference

cocentration [M	Replicate 1	Replicate 2	Replicate 3	Replicate 4
1,00E-09	0,434100002	0,46790003	0,459899977	0,454300001

Normalization (substance/1 nM E2)

cocentration [M	Replicate 1	Replicate 2	Replicate 3	Replicate 4	Mean
1,00E-07	1,61%	-0,32%	0,78%	0,26%	**0,58%**
1,00E-06	2,63%	0,04%	1,52%	0,75%	**1,23%**
1,00E-05	12,81%	10,62%	14,87%	13,69%	**13,00%**
5,00E-05	12,79%	12,01%	12,29%	13,76%	**12,71%**
1,00E-04	13,04%	11,52%	12,11%	12,77%	**12,36%**
5,00E-04	14,19%	12,61%	13,18%	14,73%	**13,68%**
1,00E-03	22,76%	16,56%	15,94%	14,31%	**17,39%**

yeast-growth/solubility 690nm/solvent control 690nm

cocentration [M	Replicate 1	Replicate 2	Replicate 3	Replicate 4	Mean
1,00E-07	113,17%	95,08%	100,58%	102,89%	**102,93%**
1,00E-06	110,54%	89,69%	107,90%	103,63%	**102,94%**
1,00E-05	100,75%	85,52%	93,28%	96,23%	**93,95%**
5,00E-05	100,02%	89,24%	85,35%	91,60%	**91,55%**
1,00E-04	101,90%	87,97%	89,68%	95,29%	**93,71%**
5,00E-04	101,71%	85,97%	90,08%	96,93%	**93,67%**
1,00E-03	119,43%	98,84%	93,35%	101,05%	**103,17%**

4th experiment

540nm

cocentration [M	Replicate 1	Replicate 2	Replicate 3	Replicate 4
1,00E-07	0,650699973	0,690400004	0,747600019	0,780499995
1,00E-06	0,763400018	0,699999988	0,723800004	0,716300011
1,00E-05	0,782999992	0,742600024	0,733299971	0,7148
5,00E-05	0,798399985	0,709200025	0,748399973	0,728900015
1,00E-04	0,817099989	0,815299988	0,752799988	0,74000001
5,00E-04	0,856700003	0,840300024	0,892199993	0,844900012
1,00E-03	0,948400021	1,01820004	1,006600022	0,944700003

690nm

cocentration [M	Replicate 1	Replicate 2	Replicate 3	Replicate 4
1,00E-07	0,565199971	0,597500026	0,648199975	0,660300016
1,00E-06	0,661800027	0,605599999	0,626800001	0,618499994
1,00E-05	0,626699984	0,602699995	0,582799971	0,57190001
5,00E-05	0,629999995	0,559899986	0,593200028	0,569299996
1,00E-04	0,646600008	0,654600024	0,593500018	0,578599989
5,00E-04	0,673900008	0,662999988	0,701300025	0,665099978
1,00E-03	0,739099979	0,809899986	0,796400011	0,746299982

solvent control 690nm

	Replicate 1	Replicate 2	Replicate 3	Replicate 4
	0,652999997	0,631399989	0,642499983	0,521899998

540-690nm

cocentration [M	Replicate 1	Replicate 2	Replicate 3	Replicate 4
1,00E-07	0,085600004	0,092900001	0,099299997	0,120200001
1,00E-06	0,101599999	0,094300002	0,097099997	0,097800002
1,00E-05	0,156299993	0,139899999	0,1505	0,142900005
5,00E-05	0,168400005	0,149299994	0,155200005	0,159600005
1,00E-04	0,170499995	0,160699993	0,159299999	0,161400005
5,00E-04	0,182799995	0,177300006	0,191	0,179900005
1,00E-03	0,209299996	0,208199993	0,210199997	0,198400006

solvent control 540nm-690nm

	Replicate 1	Replicate 2	Replicate 3	Replicate 4
	0,100699998	0,098099999	0,100100003	0,086000003

(540nm-690nm)-solvent control (540nm-690nm)

cocentration [M	Replicate 1	Replicate 2	Replicate 3	Replicate 4
1,00E-07	-0,015099995	-0,005199999	-0,000800006	0,034199998
1,00E-06	0,0009	-0,003799997	-0,003000006	0,011799999
1,00E-05	0,055599995	0,0418	0,050399996	0,056900002
5,00E-05	0,067700006	0,051199995	0,055100001	0,073600002
1,00E-04	0,069799997	0,062599994	0,059199996	0,075400002
5,00E-04	0,082099997	0,079200007	0,090899996	0,093900003
1,00E-03	0,108599998	0,110099994	0,110099994	0,112400003

(540nm-690nm)-solvent control E2 reference

cocentration [M	Replicate 1	Replicate 2	Replicate 3	Replicate 4
1,00E-09	0,531699978	0,511200018	0,53269998	0,548800014

Normalization (substance/1 nM E2)

cocentration [M	Replicate 1	Replicate 2	Replicate 3	Replicate 4	Mean
1,00E-07	-2,84%	-1,02%	-0,15%	6,23%	**0,56%**
1,00E-06	0,17%	-0,74%	-0,56%	2,15%	**0,25%**
1,00E-05	10,46%	8,18%	9,46%	10,37%	**9,62%**
5,00E-05	12,73%	10,02%	10,34%	13,41%	**11,63%**
1,00E-04	13,13%	12,25%	11,11%	13,74%	**12,56%**
5,00E-04	15,44%	15,49%	17,06%	17,11%	**16,28%**
1,00E-03	20,43%	21,54%	20,67%	20,48%	**20,78%**

yeast-growth/solubility 690nm/solvent control 690nm

cocentration [M	Replicate 1	Replicate 2	Replicate 3	Replicate 4	Mean
1,00E-07	86,55%	94,63%	100,89%	126,52%	**102,15%**
1,00E-06	101,35%	95,91%	97,56%	118,51%	**103,33%**
1,00E-05	95,97%	95,45%	90,71%	109,58%	**97,93%**
5,00E-05	96,48%	88,68%	92,33%	109,08%	**96,64%**
1,00E-04	99,02%	103,67%	92,37%	110,86%	**101,48%**
5,00E-04	103,20%	105,00%	109,15%	127,44%	**111,20%**
1,00E-03	113,19%	128,27%	123,95%	143,00%	**127,10%**

5th experiment

540nm

cocentration [M	Replicate 1	Replicate 2	Replicate 3	Replicate 4
1,00E-07	0,678900003	0,792500019	0,788999975	0,763599992
1,00E-06	0,801100016	0,849799991	0,834299982	0,7421
1,00E-05	0,685500026	0,676400006	0,712700009	0,822300017
5,00E-05	0,743099988	0,747600019	0,694599986	0,731100023
1,00E-04	0,720600009	0,89139998	0,76700002	0,737100005
5,00E-04	0,878799975	0,915199995	0,873399973	0,824800014
1,00E-03	1,03760004	1,057299972	1,064800024	0,903400004

690nm

cocentration [M	Replicate 1	Replicate 2	Replicate 3	Replicate 4
1,00E-07	0,574199975	0,681500018	0,680599988	0,65259999
1,00E-06	0,684899986	0,735099971	0,718500018	0,630800009
1,00E-05	0,532500029	0,515399992	0,545099974	0,649200022
5,00E-05	0,574999988	0,575900018	0,529999971	0,560000002
1,00E-04	0,552900016	0,704900026	0,595200002	0,56190002
5,00E-04	0,686100006	0,71509999	0,676999986	0,626800001
1,00E-03	0,824100018	0,844099998	0,850700021	0,695500016

solvent control 690nm

	Replicate 1	Replicate 2	Replicate 3	Replicate 4
	0,694999993	0,599099994	0,707199991	0,6329

540-690nm

cocentration [M	Replicate 1	Replicate 2	Replicate 3	Replicate 4
1,00E-07	0,104699999	0,111100003	0,108400002	0,111000001
1,00E-06	0,116099998	0,114799999	0,115800001	0,111299999
1,00E-05	0,152899995	0,160999998	0,167500004	0,173099995
5,00E-05	0,168099999	0,171800002	0,164700001	0,171000004
1,00E-04	0,167699993	0,186499998	0,171900004	0,1752
5,00E-04	0,1928	0,200100005	0,1963	0,197899997
1,00E-03	0,213499993	0,213200003	0,214000002	0,207900003

solvent control 540nm-690nm

	Replicate 1	Replicate 2	Replicate 3	Replicate 4
	0,111299999	0,109899998	0,109899998	0,111500002

(540nm-690nm)-solvent control (540nm-690nm)

cocentration [M	Replicate 1	Replicate 2	Replicate 3	Replicate 4
1,00E-07	-0,0066	0,001200005	-0,001499996	-0,000500001
1,00E-06	0,004799999	0,004900001	0,005900003	-0,000200003
1,00E-05	0,041599996	0,051100001	0,057600006	0,061599992
5,00E-05	0,0568	0,061900005	0,054800004	0,059500001
1,00E-04	0,056399994	0,0766	0,062000006	0,063699998
5,00E-04	0,081500001	0,090200007	0,086400002	0,086399995
1,00E-03	0,102199994	0,103300005	0,104100004	0,0964

(540nm-690nm)-solvent control E2 reference

cocentration [M	Replicate 1	Replicate 2	Replicate 3	Replicate 4
1,00E-09	0,467500024	0,534699976	0,461499989	0,449799977

Normalization (substance/1 nM E2)

cocentration [M	Replicate 1	Replicate 2	Replicate 3	Replicate 4	Mean
1,00E-07	-1,41%	0,22%	-0,33%	-0,11%	**-0,41%**
1,00E-06	1,03%	0,92%	1,28%	-0,04%	**0,79%**
1,00E-05	8,90%	9,56%	12,48%	13,69%	**11,16%**
5,00E-05	12,15%	11,58%	11,87%	13,23%	**12,21%**
1,00E-04	12,06%	14,33%	13,43%	14,16%	**13,50%**
5,00E-04	17,43%	16,87%	18,72%	19,21%	**18,06%**
1,00E-03	21,86%	19,32%	22,56%	21,43%	**21,29%**

yeast-growth/solubility 690nm/solvent control 690nm

cocentration [M	Replicate 1	Replicate 2	Replicate 3	Replicate 4	Mean
1,00E-07	82,62%	113,75%	96,24%	103,11%	**98,93%**
1,00E-06	98,55%	122,70%	101,60%	99,67%	**105,63%**
1,00E-05	76,62%	86,03%	77,08%	102,58%	**85,58%**
5,00E-05	82,73%	96,13%	74,94%	88,48%	**85,57%**
1,00E-04	79,55%	117,66%	84,16%	88,78%	**92,54%**
5,00E-04	98,72%	119,36%	95,73%	99,04%	**103,21%**
1,00E-03	118,58%	140,89%	120,29%	109,89%	**122,41%**

cell-free 690nm

cocentration [M	Replicate 1	Replicate 2	Replicate 3	Replicate 4
1,00E-07	0,037	0,036899999	0,0372	0,0403
1,00E-06	0,0374	0,036800001	0,037900001	0,0381
1,00E-05	0,037500001	0,037099998	0,037300002	0,037999999
5,00E-05	0,046100002	0,043000001	0,046100002	0,051100001
1,00E-04	0,063900001	0,060400002	0,071699999	0,074900001
5,00E-04	0,203500003	0,255600005	0,208399996	0,242300004
1,00E-03	0,434300005	0,481099993	0,43779999	0,379400015

cell-free solvent control 690nm

cocentration [M	Replicate 1	Replicate 2	Replicate 3	Replicate 4
	0,0381	0,036200002	0,036600001	0,037099998

cell-free (substance/solvent control)

cocentration [M	Replicate 1	Replicate 2	Replicate 3	Replicate 4	Mean
1,00E-07	97,11%	101,93%	101,64%	108,63%	**102,33%**
1,00E-06	98,16%	101,66%	103,55%	102,70%	**101,52%**
1,00E-05	98,43%	102,49%	101,91%	102,43%	**101,31%**
5,00E-05	121,00%	118,78%	125,96%	137,74%	**125,87%**
1,00E-04	167,72%	166,85%	195,90%	201,89%	**183,09%**
5,00E-04	534,12%	706,08%	569,40%	653,10%	**615,67%**
1,00E-03	1139,90%	1329,01%	1196,17%	1022,64%	**1171,93%**

YES fenhexamid

1st experient

540nm

cocentration [M]	Replicate 1	Replicate 2	Replicate 3	Replicate 4
1,00E-07	0,590399981	0,628499985	0,674700022	0,635500014
1,00E-06	0,685400009	0,663900018	0,595799983	0,603399992
1,00E-05	0,665400028	0,591700017	0,634800017	0,610899985
5,00E-05	0,708599985	0,715499997	0,79460001	0,712100029
1,00E-04	0,726599991	0,745700002	0,816299975	0,755800009
5,00E-04	0,291299999	0,279799998	0,289799988	0,332500011
1,00E-03	0,291299999	0,282299995	0,301099986	0,288500011

690nm

cocentration [M]	Replicate 1	Replicate 2	Replicate 3	Replicate 4
1,00E-07	0,461100012	0,495400012	0,538699985	0,503899992
1,00E-06	0,542999983	0,527100027	0,4648	0,476799995
1,00E-05	0,514299989	0,448300004	0,485199988	0,462500006
5,00E-05	0,533599973	0,53640002	0,610599995	0,530900002
1,00E-04	0,544099987	0,552100003	0,611999989	0,561699986
5,00E-04	0,181999996	0,172299996	0,185499996	0,207699999
1,00E-03	0,182899997	0,172600001	0,192399994	0,178200006

solvent control 690nm

	Replicate 1	Replicate 2	Replicate 3	Replicate 4
	0,359299988	0,52579999	0,418099999	0,456099987

540-690nm

cocentration [M]	Replicate 1	Replicate 2	Replicate 3	Replicate 4
1,00E-07	0,129299998	0,133000001	0,136000007	0,131699994
1,00E-06	0,142399997	0,136700004	0,130999997	0,126599997
1,00E-05	0,151199996	0,143399999	0,149599999	0,148399994
5,00E-05	0,174999997	0,179100007	0,184	0,181199998
1,00E-04	0,182500005	0,193499997	0,204300001	0,194100007
5,00E-04	0,109300002	0,107600003	0,1043	0,124799997
1,00E-03	0,108400002	0,1096	0,1087	0,110399999

solvent control 540nm-690nm

	Replicate 1	Replicate 2	Replicate 3	Replicate 4
	0,105700001	0,135600001	0,114299998	0,121600002

(540nm-690nm)-solvent control (540nm-690nm)

cocentration [M]	Replicate 1	Replicate 2	Replicate 3	Replicate 4
1,00E-07	0,023599997	-0,002599999	0,02170001	0,010099992
1,00E-06	0,036699995	0,001100004	0,0167	0,004999995
1,00E-05	0,045499995	0,007799998	0,035300002	0,026799992
5,00E-05	0,069299996	0,043500006	0,069700003	0,059599996
1,00E-04	0,076800004	0,057899997	0,090000004	0,072500005
5,00E-04	0,003600001	-0,027999997	-0,009999998	0,003199995
1,00E-03	0,002700001	-0,026000001	-0,005599998	-0,011200003

(540nm-690nm)-solvent control E2 reference

cocentration [M]	Replicate 1	Replicate 2	Replicate 3	Replicate 4
1,00E-09	0,412499979	0,377599984	0,430899993	0,533399969

Normalization (substance/1 nM E2)

cocentration [M]	Replicate 1	Replicate 2	Replicate 3	Replicate 4	Mean
1,00E-07	5,72%	-0,69%	5,04%	1,89%	**2,99%**
1,00E-06	8,90%	0,29%	3,88%	0,94%	**3,50%**
1,00E-05	11,03%	2,07%	8,19%	5,02%	**6,58%**
5,00E-05	16,80%	11,52%	16,18%	11,17%	**13,92%**
1,00E-04	18,62%	15,33%	20,89%	13,59%	**17,11%**
5,00E-04	0,87%	-7,42%	-2,32%	0,60%	**-2,07%**
1,00E-03	0,65%	-6,89%	-1,30%	-2,10%	**-2,41%**

yeast-growth/solubility 690nm/solvent control 690nm

cocentration [M]	Replicate 1	Replicate 2	Replicate 3	Replicate 4	Mean
1,00E-07	128,33%	94,22%	128,84%	110,48%	**115,47%**
1,00E-06	151,13%	100,25%	111,17%	104,54%	**116,77%**
1,00E-05	143,14%	85,26%	116,05%	101,40%	**111,46%**
5,00E-05	148,51%	102,02%	146,04%	116,40%	**128,24%**
1,00E-04	50,65%	32,77%	44,37%	45,54%	**43,33%**
5,00E-04	151,43%	105,00%	146,38%	123,15%	**131,49%**
1,00E-03	50,90%	32,83%	46,02%	39,07%	**42,20%**

excluded

2nd experient

540nm

cocentration [M]	Replicate 1	Replicate 2	Replicate 3	Replicate 4
1,00E-07	0,676199973	0,637399971	0,627699971	0,595300019
1,00E-06	0,710799992	0,648899972	0,676699996	0,732800007
1,00E-05	0,69569999	0,643299997	0,609799981	0,550100029
5,00E-05	0,672299981	0,717299998	0,713699996	0,707099974
1,00E-04	0,689999998	0,783699989	0,781199992	0,711799979
5,00E-04	0,390700012	0,27790001	0,272899985	0,272899985
1,00E-03	0,276499987	0,264699996	0,293099999	0,267100006

690nm

cocentration [M]	Replicate 1	Replicate 2	Replicate 3	Replicate 4
1,00E-07	0,539200008	0,505999982	0,493000001	0,469399989
1,00E-06	0,569999993	0,514800012	0,537999988	0,598800004
1,00E-05	0,537599981	0,491100013	0,459800005	0,406399995
5,00E-05	0,494399995	0,532100022	0,524600029	0,517499983
1,00E-04	0,491899997	0,583000004	0,574899971	0,504499972
5,00E-04	0,236300007	0,153500006	0,153799996	0,156000003
1,00E-03	0,155900002	0,143000007	0,167099997	0,151099995

solvent control 690nm

	Replicate 1	Replicate 2	Replicate 3	Replicate 4
	0,474599987	0,621699989	0,486999989	0,496800005

540-690nm

cocentration [M]	Replicate 1	Replicate 2	Replicate 3	Replicate 4
1,00E-07	0,136899993	0,131400004	0,134800002	0,126000002
1,00E-06	0,140799999	0,134200007	0,138699993	0,134100005
1,00E-05	0,158099994	0,152199996	0,150000006	0,143700004
5,00E-05	0,177900001	0,185200006	0,189199999	0,189500004
1,00E-04	0,198100001	0,2007	0,206300005	0,207300007
5,00E-04	0,154400006	0,124300003	0,119099997	0,116899997
1,00E-03	0,1206	0,121699996	0,126000002	0,115999997

solvent control 540nm-690nm

	Replicate 1	Replicate 2	Replicate 3	Replicate 4
	0,123400003	0,134399995	0,128099993	0,126100004

(540nm-690nm)-solvent control (540nm-690nm)

cocentration [M]	Replicate 1	Replicate 2	Replicate 3	Replicate 4
1,00E-07	0,01349999	-0,002999991	0,006700009	-0,000100002
1,00E-06	0,017399997	-0,000199988	0,010600001	0,008000001
1,00E-05	0,034699991	0,017800003	0,021900013	0,0176
5,00E-05	0,054499999	0,050800011	0,061100006	0,0634
1,00E-04	0,074699998	0,066300005	0,078200012	0,081200004
5,00E-04	0,031000003	-0,010099992	-0,008999996	-0,009200007
1,00E-03	-0,002800003	-0,012699999	-0,002099991	-0,010100007

(540nm-690nm)-solvent control E2 reference

cocentration [M]	Replicate 1	Replicate 2	Replicate 3	Replicate 4
1,00E-09	0,318199992	0,327400014	0,32600002	0,377300024

Normalization (substance/1 nM E2)

cocentration [M]	Replicate 1	Replicate 2	Replicate 3	Replicate 4	Mean
1,00E-07	4,24%	-0,92%	2,06%	-0,03%	**1,34%**
1,00E-06	5,47%	-0,06%	3,25%	2,12%	**2,69%**
1,00E-05	10,91%	5,44%	6,72%	4,66%	**6,93%**
5,00E-05	17,13%	15,52%	18,74%	16,80%	**17,05%**
1,00E-04	23,48%	20,25%	23,99%	21,52%	**22,31%**
5,00E-04	9,74%	-3,08%	-2,76%	-2,44%	**0,36%**
1,00E-03	-0,88%	-3,88%	-0,64%	-2,68%	**-2,02%**

yeast-growth/solubility 690nm/solvent control 690nm

cocentration [M]	Replicate 1	Replicate 2	Replicate 3	Replicate 4	Mean
1,00E-07	113,61%	81,39%	101,23%	94,48%	**97,68%**
1,00E-06	120,10%	82,81%	110,47%	120,53%	**108,48%**
1,00E-05	113,27%	78,99%	94,41%	81,80%	**92,12%**
5,00E-05	104,17%	85,59%	107,72%	104,17%	**100,41%**
1,00E-04	103,65%	93,78%	118,05%	101,55%	**104,25%**
5,00E-04	49,79%	24,69%	31,58%	31,40%	**34,37%**
1,00E-03	32,85%	23,00%	34,31%	30,41%	**30,14%**

3rd experiment

540nm

cocentration [M	Replicate 1	Replicate 2	Replicate 3	Replicate 4
1,00E-07	0,720499992	0,800899982	0,779100001	0,70569998
1,00E-06	0,800000012	0,783399999	0,750999987	0,69749999
1,00E-05	0,809000015	0,792999983	0,73299998	0,667299986
5,00E-05	0,736800015	0,778999984	0,831900001	0,773599982
1,00E-04	0,674700022	0,663600028	0,776300013	0,739499986
5,00E-04	0,183599994	0,1175	0,117600001	0,115900002
1,00E-03	0,148300007	0,132200003	0,136800006	0,137400001

690nm

cocentration [M	Replicate 1	Replicate 2	Replicate 3	Replicate 4
1,00E-07	0,611599982	0,684400022	0,663200021	0,595099986
1,00E-06	0,67930001	0,664499998	0,636699975	0,586600006
1,00E-05	0,647199988	0,63380003	0,574800014	0,504499972
5,00E-05	0,562600017	0,5977	0,630200028	0,594399989
1,00E-04	0,506500006	0,49180001	0,597899973	0,558600008
5,00E-04	0,097499996	0,0495	0,0493	0,050500002
1,00E-03	0,071999997	0,0539	0,057300001	0,0502

solvent control 690nm

	Replicate 1	Replicate 2	Replicate 3	Replicate 4
	0,635699987	0,589299977	0,655399978	0,606700003

540-690nm

cocentration [M	Replicate 1	Replicate 2	Replicate 3	Replicate 4
1,00E-07	0,108900003	0,116499998	0,115900002	0,110600002
1,00E-06	0,120800003	0,118799999	0,114299998	0,1109
1,00E-05	0,161699995	0,159199998	0,158199996	0,162799999
5,00E-05	0,174199998	0,181299999	0,201700002	0,179100007
1,00E-04	0,168200001	0,171800002	0,178499997	0,180899993
5,00E-04	0,086099997	0,068000004	0,068300001	0,065399997
1,00E-03	0,076300003	0,078299999	0,079499997	0,087200001

solvent control 540nm-690nm

	Replicate 1	Replicate 2	Replicate 3	Replicate 4
	0,111199997	0,107600003	0,113499999	0,107699998

(540nm-690nm)-solvent control (540nm-690nm)

cocentration [M	Replicate 1	Replicate 2	Replicate 3	Replicate 4
1,00E-07	-0,002299994	0,008899994	0,002400003	0,002900004
1,00E-06	0,009600006	0,011199996	0,000799999	0,003200002
1,00E-05	0,050499998	0,051599994	0,044699997	0,055100001
5,00E-05	0,063000001	0,073699996	0,088200003	0,071400009
1,00E-04	0,057000004	0,064199999	0,064999998	0,073199995
5,00E-04	-0,0251	-0,0396	-0,045199998	-0,042300001
1,00E-03	-0,034899995	-0,029300004	-0,034000002	-0,020499997

(540nm-690nm)-solvent control E2 reference

cocentration [M	Replicate 1	Replicate 2	Replicate 3	Replicate 4
1,00E-09	0,474199988	0,476600021	0,456499994	0,480800025

Normalization (substance/1 nM E2)

cocentration [M	Replicate 1	Replicate 2	Replicate 3	Replicate 4	Mean
1,00E-07	-0,49%	1,87%	0,53%	0,60%	**0,63%**
1,00E-06	2,02%	2,35%	0,18%	0,67%	**1,30%**
1,00E-05	10,65%	10,83%	9,79%	11,46%	**10,68%**
5,00E-05	13,29%	15,46%	19,32%	14,85%	**15,73%**
1,00E-04	12,02%	13,47%	14,24%	15,22%	**13,74%**
5,00E-04	-5,29%	-8,31%	-9,90%	-8,80%	**-8,08%**
1,00E-03	-7,36%	-6,15%	-7,45%	-4,26%	**-6,30%**

yeast-growth/solubility 690nm/solvent control 690nm

cocentration [M	Replicate 1	Replicate 2	Replicate 3	Replicate 4	Mean
1,00E-07	96,21%	116,14%	101,19%	98,09%	**102,91%**
1,00E-06	106,86%	112,76%	97,15%	96,69%	**103,36%**
1,00E-05	101,81%	107,55%	87,70%	83,15%	**95,05%**
5,00E-05	88,50%	101,43%	96,16%	97,97%	**96,01%**
1,00E-04	79,68%	83,45%	91,23%	92,07%	**86,61%**
5,00E-04	15,34%	8,40%	7,52%	8,32%	**9,90%**
1,00E-03	11,33%	9,15%	8,74%	8,27%	**9,37%**

4th experiment

540nm

cocentration [M	Replicate 1	Replicate 2	Replicate 3	Replicate 4
1,00E-07	0,793200016	0,777800024	0,788800001	0,771700025
1,00E-06	0,815500021	0,735400021	0,7403	0,721800029
1,00E-05	0,782999992	0,80250001	0,722000003	0,79430002
5,00E-05	0,737800002	0,726499975	0,823499978	0,774800003
1,00E-04	0,685699999	0,723200023	0,65200001	0,774900019
5,00E-04	0,095299996	0,097099997	0,109099999	0,091899998
1,00E-03	0,115000002	0,124399997	0,119999997	0,104699999

690nm

cocentration [M	Replicate 1	Replicate 2	Replicate 3	Replicate 4
1,00E-07	0,688399971	0,674899995	0,681900024	0,669799984
1,00E-06	0,709699988	0,637099981	0,640200019	0,620800018
1,00E-05	0,645099998	0,657000005	0,594799995	0,651600003
5,00E-05	0,575500011	0,564700007	0,643700004	0,600799978
1,00E-04	0,52759999	0,56129998	0,4912	0,601000011
5,00E-04	0,054299999	0,053300001	0,061299998	0,0493
1,00E-03	0,070299998	0,0788	0,0748	0,059099998

solvent control 690nm

	Replicate 1	Replicate 2	Replicate 3	Replicate 4
	0,622200012	0,581200004	0,603100002	0,60589999

540-690nm

cocentration [M	Replicate 1	Replicate 2	Replicate 3	Replicate 4
1,00E-07	0,104800001	0,103	0,107000001	0,101899996
1,00E-06	0,105800003	0,098300003	0,100100003	0,101000004
1,00E-05	0,137899995	0,145400003	0,127200007	0,1426
5,00E-05	0,162300006	0,161799997	0,179800004	0,173999995
1,00E-04	0,158099994	0,161899996	0,160699993	0,173899993
5,00E-04	0,040899999	0,0438	0,047800001	0,042599998
1,00E-03	0,0447	0,045499999	0,045200001	0,045699999

solvent control 540nm-690nm

	Replicate 1	Replicate 2	Replicate 3	Replicate 4
	0,097000003	0,088	0,094599999	0,093599997

(540nm-690nm)-solvent control (540nm-690nm)

cocentration [M	Replicate 1	Replicate 2	Replicate 3	Replicate 4
1,00E-07	0,0088	0,010300003	0,005500004	0,007400006
1,00E-06	0,040899992	0,057400003	0,032600008	0,049000002
1,00E-05	0,065300003	0,073799998	0,085200004	0,080399998
5,00E-05	0,061099991	0,073899999	0,066099994	0,080299996
1,00E-04	-0,056100003	-0,044199999	-0,046799999	-0,050999999
5,00E-04	-0,052300002	-0,0425	-0,049399998	-0,047899999
1,00E-03	-0,097000003	-0,088	-0,094599999	-0,093599997

(540nm-690nm)-solvent control E2 reference

cocentration [M	Replicate 1	Replicate 2	Replicate 3	Replicate 4
1,00E-09	0,51669997	0,391900003	0,3957	0,394299991

Normalization (substance/1 nM E2)

cocentration [M	Replicate 1	Replicate 2	Replicate 3	Replicate 4	Mean
1,00E-07	1,70%	2,63%	1,39%	1,88%	**1,90%**
1,00E-06	7,92%	14,65%	8,24%	12,43%	**10,81%**
1,00E-05	12,64%	18,83%	21,53%	20,39%	**18,35%**
5,00E-05	11,83%	18,86%	16,70%	20,37%	**16,94%**
1,00E-04	-10,86%	-11,28%	-11,83%	-12,93%	**-11,72%**
5,00E-04	-10,12%	-10,84%	-12,48%	-12,15%	**-11,40%**
1,00E-03	-18,77%	-22,45%	-23,91%	-23,74%	**-22,22%**

yeast-growth/solubility 690nm/solvent control 690nm

cocentration [M	Replicate 1	Replicate 2	Replicate 3	Replicate 4	Mean
1,00E-07	110,64%	116,12%	113,07%	110,55%	**112,59%**
1,00E-06	114,06%	109,62%	106,15%	102,46%	**108,07%**
1,00E-05	103,68%	113,04%	98,62%	107,54%	**105,72%**
5,00E-05	92,49%	97,16%	106,73%	99,16%	**98,89%**
1,00E-04	84,80%	96,58%	81,45%	99,19%	**90,50%**
5,00E-04	8,73%	9,17%	10,16%	8,14%	**9,05%**
1,00E-03	11,30%	13,56%	12,40%	9,75%	**11,75%**

5th experiment

540nm

cocentration [M	Replicate 1	Replicate 2	Replicate 3	Replicate 4
1,00E-07	0,808399975	0,71359998	0,693400025	0,716600001
1,00E-06	0,765900016	0,76730001	0,831099987	0,751399994
1,00E-05	0,717899978	0,734300017	0,779500008	0,750400007
5,00E-05	0,723999977	0,657599986	0,759299994	0,714600027
1,00E-04	0,623700023	0,702300012	0,802299976	0,735899985
5,00E-04	0,264499992	0,1043	0,118500002	0,0986
1,00E-03	0,095700003	0,1131	0,102399997	0,138799995

690nm

cocentration [M	Replicate 1	Replicate 2	Replicate 3	Replicate 4
1,00E-07	0,695100009	0,609399974	0,586399972	0,610599995
1,00E-06	0,653900027	0,655300021	0,716600001	0,64230001
1,00E-05	0,576699972	0,588800013	0,626699984	0,604099989
5,00E-05	0,547100008	0,489100009	0,58099997	0,529200017
1,00E-04	0,448199987	0,524100006	0,623199999	0,547800004
5,00E-04	0,183200002	0,054400001	0,066200003	0,050299998
1,00E-03	0,049199998	0,064499997	0,050799999	0,086599998

solvent control 690nm

	Replicate 1	Replicate 2	Replicate 3	Replicate 4
	0,539600015	0,621699989	0,603900015	0,632200003

540-690nm

cocentration [M	Replicate 1	Replicate 2	Replicate 3	Replicate 4
1,00E-07	0,113300003	0,104099996	0,107000001	0,105999999
1,00E-06	0,112000003	0,112000003	0,114500001	0,109099999
1,00E-05	0,141100004	0,145500004	0,152799994	0,146300003
5,00E-05	0,176899999	0,168400005	0,178299993	0,185299993
1,00E-04	0,175500005	0,178200006	0,179100007	0,188099995
5,00E-04	0,081299998	0,049899999	0,052299999	0,048300002
1,00E-03	0,046500001	0,048599999	0,0515	0,052200001

solvent control 540nm-690nm

	Replicate 1	Replicate 2	Replicate 3	Replicate 4
	0,098899998	0,108999997	0,109200001	0,1061

(540nm-690nm)-solvent control (540nm-690nm)

cocentration [M	Replicate 1	Replicate 2	Replicate 3	Replicate 4
1,00E-07	0,014400005	-0,004900001	-0,0022	-0,000100002
1,00E-06	0,013100006	0,003000006	0,0053	0,002999999
1,00E-05	0,042200007	0,036500007	0,043599993	0,040200002
5,00E-05	0,078000002	0,059400007	0,069099993	0,079199992
1,00E-04	0,076600008	0,069200009	0,069900006	0,081999995
5,00E-04	-0,0176	-0,059099998	-0,056900002	-0,057799999
1,00E-03	-0,052399997	-0,060399998	-0,057700001	-0,0539

(540nm-690nm)-solvent control E2 reference

cocentration [M	Replicate 1	Replicate 2	Replicate 3	Replicate 4
1,00E-09	0,508200012	0,533000022	0,477800012	0,47639998

Normalization (substance/1 nM E2)

cocentration [M	Replicate 1	Replicate 2	Replicate 3	Replicate 4	Mean
1,00E-07	2,83%	-0,92%	-0,46%	-0,02%	**0,36%**
1,00E-06	2,58%	0,56%	1,11%	0,63%	**1,22%**
1,00E-05	8,30%	6,85%	9,13%	8,44%	**8,18%**
5,00E-05	15,35%	11,14%	14,46%	16,62%	**14,39%**
1,00E-04	15,07%	12,98%	14,63%	17,21%	**14,97%**
5,00E-04	-3,46%	-11,09%	-11,91%	-12,13%	**-9,65%**
1,00E-03	-10,31%	-11,33%	-12,08%	-11,31%	**-11,26%**

yeast-growth/solubility 690nm/solvent control 690nm

cocentration [M	Replicate 1	Replicate 2	Replicate 3	Replicate 4	Mean
1,00E-07	128,82%	98,02%	97,10%	96,58%	**105,13%**
1,00E-06	121,18%	105,40%	118,66%	101,60%	**111,71%**
1,00E-05	106,88%	94,71%	103,78%	95,56%	**100,23%**
5,00E-05	101,39%	78,67%	96,21%	83,71%	**89,99%**
1,00E-04	83,06%	84,30%	103,20%	86,65%	**89,30%**
5,00E-04	33,95%	8,75%	10,96%	7,96%	**15,40%**
1,00E-03	9,12%	10,37%	8,41%	13,70%	**10,40%**

cell-free 690nm

cocentration [M	Replicate 1	Replicate 2	Replicate 3	Replicate 4
1,00E-07	0,039099999	0,036800001	0,037	0,0418
1,00E-06	0,037099998	0,036899999	0,038899999	0,0381
1,00E-05	0,037300002	0,036800001	0,036899999	0,037999999
5,00E-05	0,038800001	0,037300002	0,037700001	0,0381
1,00E-04	0,0385	0,037799999	0,0385	0,0383
5,00E-04	0,0407	0,043699998	0,040600002	0,041499998
1,00E-03	0,041900001	0,0405	0,044100001	0,040600002

cell-free solvent control 690nm

cocentration [M	Replicate 1	Replicate 2	Replicate 3	Replicate 4
	0,0394	0,0374	0,037	0,036800001

cell-free (substance/solvent control)

cocentration [M	Replicate 1	Replicate 2	Replicate 3	Replicate 4	Mean
1,00E-07	99,24%	98,40%	100,00%	113,59%	**102,81%**
1,00E-06	94,16%	98,66%	105,14%	103,53%	**100,37%**
1,00E-05	94,67%	98,40%	99,73%	103,26%	**99,01%**
5,00E-05	98,48%	99,73%	101,89%	103,53%	**100,91%**
1,00E-04	97,72%	101,07%	104,05%	104,08%	**101,73%**
5,00E-04	103,30%	116,84%	109,73%	112,77%	**110,66%**
1,00E-03	106,35%	108,29%	119,19%	110,33%	**111,04%**

YES 4,4'-DDT

1st experient

540nm

cocentration [M]	Replicate 1	Replicate 2	Replicate 3	Replicate 4
1,00E-07	0,676900029	0,67809999	0,683899999	0,639900029
1,00E-06	0,666700006	0,670400023	0,638300002	0,632600009
5,00E-06	0,689599991	0,707700014	0,576600015	0,574299991
1,00E-05	0,64410001	0,682900012	0,601700008	0,542400002
5,00E-05	0,736000001	0,651000023	0,645600021	0,558899999
1,00E-04	0,738399982	0,730400026	0,602400005	0,663800001
5,00E-04	0,652499974	0,673699975	0,592700005	0,575800002

690nm

cocentration [M]	Replicate 1	Replicate 2	Replicate 3	Replicate 4
1,00E-07	0,538299978	0,540400028	0,547100008	0,513000011
1,00E-06	0,522599995	0,531899989	0,500199974	0,503700018
5,00E-06	0,521600008	0,540799975	0,432900012	0,434399992
1,00E-05	0,466600001	0,5	0,431699991	0,388399988
5,00E-05	0,524999976	0,449999988	0,445899993	0,373199999
1,00E-04	0,518400013	0,510399997	0,402399987	0,459600002
5,00E-04	0,442200005	0,4639	0,394899994	0,380800009

solvent control 690nm

	Replicate 1	Replicate 2	Replicate 3	Replicate 4
	0,594699979	0,503000021	0,492399991	0,466600001

540-690nm

cocentration [M]	Replicate 1	Replicate 2	Replicate 3	Replicate 4
1,00E-07	0,138699993	0,137600005	0,136800006	0,126800001
1,00E-06	0,144199997	0,138500005	0,137999997	0,128900006
5,00E-06	0,167899996	0,166899994	0,143700004	0,139799997
1,00E-05	0,177499995	0,182899997	0,170000002	0,153999999
5,00E-05	0,210999995	0,200900003	0,199699998	0,185699999
1,00E-04	0,219899997	0,220100001	0,200000003	0,2042
5,00E-04	0,210199997	0,209800005	0,197799996	0,194999993

solvent control 540nm-690nm

	Replicate 1	Replicate 2	Replicate 3	Replicate 4
	0,132799998	0,129199997	0,127299994	0,122699998

(540nm-690nm)-solvent control (540nm-690nm)

cocentration [M]	Replicate 1	Replicate 2	Replicate 3	Replicate 4
1,00E-07	0,005899996	0,008400008	0,009500012	0,004100002
1,00E-06	0,011399999	0,009300008	0,010700002	0,006200008
5,00E-06	0,035099998	0,037699997	0,016400009	0,017099999
1,00E-05	0,044699997	0,0537	0,042700008	0,031300001
5,00E-05	0,078199998	0,071700007	0,072400004	0,063000001
1,00E-04	0,087099999	0,090900004	0,072700009	0,081500001
5,00E-04	0,077399999	0,080600008	0,070500001	0,072299995

(540nm-690nm)-solvent control E2 reference

cocentration [M]	Replicate 1	Replicate 2	Replicate 3	Replicate 4
1,00E-09	0,47860001	0,408799991	0,405900031	0,414599992

Normalization (substance/1 nM E2)

cocentration [M]	Replicate 1	Replicate 2	Replicate 3	Replicate 4	Mean
1,00E-07	1,23%	2,05%	2,34%	0,99%	**1,65%**
1,00E-06	2,38%	2,27%	2,64%	1,50%	**2,20%**
5,00E-06	7,33%	9,22%	4,04%	4,12%	**6,18%**
1,00E-05	9,34%	13,14%	10,52%	7,55%	**10,14%**
5,00E-05	16,34%	17,54%	17,84%	15,20%	**16,73%**
1,00E-04	18,20%	22,24%	17,91%	19,66%	**19,50%**
5,00E-04	16,17%	19,72%	17,37%	17,44%	**17,67%**

yeast-growth/solubility 690nm/solvent control 690nm

cocentration [M]	Replicate 1	Replicate 2	Replicate 3	Replicate 4	Mean
1,00E-07	90,52%	107,44%	111,11%	109,94%	**104,75%**
1,00E-06	87,88%	105,75%	101,58%	107,95%	**100,79%**
5,00E-06	87,71%	107,51%	87,92%	93,10%	**94,06%**
1,00E-05	78,46%	99,40%	87,67%	83,24%	**87,19%**
5,00E-05	87,17%	101,47%	81,72%	98,50%	**92,22%**
1,00E-04	88,28%	89,46%	90,56%	79,98%	**87,07%**
5,00E-04	74,36%	92,23%	80,20%	81,61%	**82,10%**

2nd experient

540nm

cocentration [M]	Replicate 1	Replicate 2	Replicate 3	Replicate 4
1,00E-07	0,835200012	0,623000026	0,607299984	0,646499991
1,00E-06	0,703100026	0,624100029	0,612500012	0,586799979
5,00E-06	0,674399972	0,629499972	0,615100026	0,559700012
1,00E-05	0,673099995	0,697000027	0,672699988	0,683300018
5,00E-05	0,690900028	0,730599999	0,6699	0,650200009
1,00E-04	0,629100025	0,726899981	0,790600002	0,739300013
5,00E-04	0,587499976	0,579699993	0,730599999	0,656700015

690nm

cocentration [M]	Replicate 1	Replicate 2	Replicate 3	Replicate 4
1,00E-07	0,692099988	0,492300004	0,478300005	0,513700008
1,00E-06	0,558099985	0,489600003	0,476700008	0,454299986
5,00E-06	0,51730001	0,47299999	0,461499989	0,417199999
1,00E-05	0,502900004	0,522400022	0,500100017	0,514199972
5,00E-05	0,493600011	0,528299987	0,475499988	0,457800001
1,00E-04	0,4278	0,513400018	0,568300009	0,522499979
5,00E-04	0,389800012	0,387800008	0,506099999	0,437000006

solvent control 690nm

	Replicate 1	Replicate 2	Replicate 3	Replicate 4
	0,423099995	0,467900008	0,454400003	0,470899999

540-690nm

cocentration [M]	Replicate 1	Replicate 2	Replicate 3	Replicate 4
1,00E-07	0,143099993	0,130600005	0,128999993	0,132799998
1,00E-06	0,144999996	0,134399995	0,135700002	0,132400006
5,00E-06	0,157100007	0,156499997	0,153500006	0,142499998
1,00E-05	0,170300007	0,174600005	0,172600001	0,169100001
5,00E-05	0,197300002	0,202199996	0,194399998	0,192399994
1,00E-04	0,201299995	0,213499993	0,222299993	0,216700003
5,00E-04	0,197699994	0,1919	0,224399999	0,219699994

solvent control 540nm-690nm

	Replicate 1	Replicate 2	Replicate 3	Replicate 4
	0,118699998	0,127100006	0,124499999	0,130500004

(540nm-690nm)-solvent control (540nm-690nm)

cocentration [M]	Replicate 1	Replicate 2	Replicate 3	Replicate 4
1,00E-07	0,004000008	0,008900002	-0,004700005	0,002199993
1,00E-06	0,01380001	0,0035	-0,007100001	-0,004800007
5,00E-06	0,015100002	0,030100003	0,017800003	0,022499993
1,00E-05	0,017100006	0,029300004	0,015000001	0,025899991
5,00E-05	0,026600003	0,032600001	0,025999993	0,018999994
1,00E-04	0,059600011	0,056899995	0,039199993	0,038399994
5,00E-04	0,09040001	0,070299998	0,063600004	0,07249999

(540nm-690nm)-solvent control E2 reference

cocentration [M]	Replicate 1	Replicate 2	Replicate 3	Replicate 4
1,00E-09	0,394699976	0,422400028	0,38970001	0,397199973

Normalization (substance/1 nM E2)

cocentration [M]	Replicate 1	Replicate 2	Replicate 3	Replicate 4	Mean
1,00E-07	1,01%	2,11%	-1,21%	0,55%	**0,62%**
1,00E-06	3,50%	0,83%	-1,82%	-1,21%	**0,32%**
5,00E-06	3,83%	7,13%	4,57%	5,66%	**5,30%**
1,00E-05	4,33%	6,94%	3,85%	6,52%	**5,41%**
5,00E-05	6,74%	7,72%	6,67%	4,78%	**6,48%**
1,00E-04	15,10%	13,47%	10,06%	9,67%	**12,07%**
5,00E-04	22,90%	16,64%	16,32%	18,25%	**18,53%**

yeast-growth/solubility 690nm/solvent control 690nm

cocentration [M]	Replicate 1	Replicate 2	Replicate 3	Replicate 4	Mean
1,00E-07	163,58%	105,21%	105,26%	109,09%	**120,79%**
1,00E-06	131,91%	104,64%	104,91%	96,47%	**109,48%**
5,00E-06	122,26%	101,09%	101,56%	88,60%	**103,38%**
1,00E-05	118,86%	111,65%	110,06%	109,20%	**112,44%**
5,00E-05	116,66%	112,91%	104,64%	97,22%	**107,86%**
1,00E-04	101,11%	109,72%	125,07%	110,96%	**111,71%**
5,00E-04	92,13%	82,88%	111,38%	92,80%	**94,80%**

3rd experiment

540nm

cocentration [M	Replicate 1	Replicate 2	Replicate 3	Replicate 4
1,00E-07	0,75880003	0,790899992	0,771300018	0,804099977
1,00E-06	0,867799997	0,788900018	0,801599979	0,762099981
5,00E-06	0,772099972	0,769999981	0,758400023	0,780499995
1,00E-05	0,843200028	0,805299997	0,8574	0,906899989
5,00E-05	0,800100029	0,834500015	0,869400024	0,828599989
1,00E-04	0,769599974	0,873399973	0,860499978	0,801299989
5,00E-04	0,876399994	0,91170001	0,870599985	0,967400014

690nm

cocentration [M	Replicate 1	Replicate 2	Replicate 3	Replicate 4
1,00E-07	0,645799994	0,674099982	0,656000018	0,686500013
1,00E-06	0,729900002	0,664799988	0,672999978	0,637099981
5,00E-06	0,61559999	0,612299979	0,603500009	0,623099983
1,00E-05	0,662699997	0,623899996	0,675100029	0,726199985
5,00E-05	0,586700022	0,618099988	0,6505	0,611400008
1,00E-04	0,545700014	0,637499988	0,620999992	0,57069999
5,00E-04	0,60589999	0,643899977	0,60769999	0,683399975

solvent control 690nm

	Replicate 1	Replicate 2	Replicate 3	Replicate 4
	0,686100006	0,600399971	0,662400007	0,674199998

540-690nm

cocentration [M	Replicate 1	Replicate 2	Replicate 3	Replicate 4
1,00E-07	0,112999998	0,116700001	0,1153	0,1175
1,00E-06	0,137899995	0,1241	0,128600001	0,125
5,00E-06	0,156399995	0,157800004	0,154899999	0,157399997
1,00E-05	0,180500001	0,181400001	0,182300001	0,180700004
5,00E-05	0,213400006	0,216399997	0,218999997	0,217199996
1,00E-04	0,223900005	0,2359	0,239500001	0,230599999
5,00E-04	0,270500004	0,267800003	0,262899995	0,284000009

solvent control 540nm-690nm

	Replicate 1	Replicate 2	Replicate 3	Replicate 4
	0,115699999	0,111100003	0,115599997	0,117399998

(540nm-690nm)-solvent control (540nm-690nm)

cocentration [M	Replicate 1	Replicate 2	Replicate 3	Replicate 4
1,00E-07	-0,002700001	0,005599998	-0,000299998	0,000100002
1,00E-06	0,022199996	0,012999997	0,013000004	0,007600002
5,00E-06	0,040699996	0,046700001	0,039300002	0,039999999
1,00E-05	0,064800002	0,070299998	0,066700004	0,063300006
5,00E-05	0,097700007	0,105299994	0,103399999	0,099799998
1,00E-04	0,108200006	0,124799997	0,123900004	0,113200001
5,00E-04	0,154800005	0,1567	0,147299998	0,166600011

(540nm-690nm)-solvent control E2 reference

cocentration [M	Replicate 1	Replicate 2	Replicate 3	Replicate 4
1,00E-09	0,500000007	0,438200012	0,495200031	0,553300016

Normalization (substance/1 nM E2)

cocentration [M	Replicate 1	Replicate 2	Replicate 3	Replicate 4	Mean
1,00E-07	-0,54%	1,28%	-0,06%	0,02%	**0,17%**
1,00E-06	4,44%	2,97%	2,63%	1,37%	**2,85%**
5,00E-06	8,14%	10,66%	7,94%	7,23%	**8,49%**
1,00E-05	12,96%	16,04%	13,47%	11,44%	**13,48%**
5,00E-05	19,54%	24,03%	20,88%	18,04%	**20,62%**
1,00E-04	21,64%	28,48%	25,02%	20,46%	**23,90%**
5,00E-04	30,96%	35,76%	29,75%	30,11%	**31,64%**

yeast-growth/solubility 690nm/solvent control 690nm

cocentration [M	Replicate 1	Replicate 2	Replicate 3	Replicate 4	Mean
1,00E-07	94,13%	112,28%	99,03%	101,82%	**101,81%**
1,00E-06	106,38%	110,73%	101,60%	94,50%	**103,30%**
5,00E-06	89,72%	101,98%	91,11%	92,42%	**93,81%**
1,00E-05	96,59%	103,91%	101,92%	107,71%	**102,53%**
5,00E-05	85,51%	102,95%	98,20%	90,69%	**94,34%**
1,00E-04	79,54%	106,18%	93,75%	84,65%	**91,03%**
5,00E-04	88,31%	107,25%	91,74%	101,36%	**97,17%**

4th experiment

540nm

cocentration [M	Replicate 1	Replicate 2	Replicate 3	Replicate 4
1,00E-07	0,762099981	0,766099989	0,759500027	0,716199994
1,00E-06	0,780799985	0,726899981	0,789499998	0,763100028
5,00E-06	0,767099977	0,870299995	0,721899986	0,781700015
1,00E-05	0,838	0,860499978	0,836499989	0,835900009
5,00E-05	0,894800007	0,886399984	0,829200029	0,836499989
1,00E-04	0,885299981	0,866900027	0,838400006	0,942399979
5,00E-04	1,06400001	0,898800015	1,016700029	1,093099952

690nm

cocentration [M	Replicate 1	Replicate 2	Replicate 3	Replicate 4
1,00E-07	0,662	0,665899992	0,657400012	0,619199991
1,00E-06	0,672800004	0,623399973	0,682200015	0,656599998
5,00E-06	0,633000016	0,731100023	0,595300019	0,648599982
1,00E-05	0,68809998	0,698000014	0,680899978	0,680000007
5,00E-05	0,712000012	0,695800006	0,65109998	0,649600029
1,00E-04	0,689599991	0,672500014	0,641600013	0,735599995
5,00E-04	0,791199982	0,677299976	0,783100009	0,848100007

solvent control 690nm

	Replicate 1	Replicate 2	Replicate 3	Replicate 4
	0,586600006	0,625500023	0,548900008	0,590300024

540-690nm

cocentration [M	Replicate 1	Replicate 2	Replicate 3	Replicate 4
1,00E-07	0,100100003	0,100199997	0,102200001	0,096900001
1,00E-06	0,108099997	0,103500001	0,107299998	0,1065
5,00E-06	0,134100005	0,139200002	0,126599997	0,133200005
1,00E-05	0,149900004	0,162499994	0,155599996	0,156000003
5,00E-05	0,182799995	0,190599993	0,178100005	0,186900005
1,00E-04	0,195800006	0,194499999	0,196799994	0,206699997
5,00E-04	0,272899985	0,221499994	0,233600006	0,245000005

solvent control 540nm-690nm

	Replicate 1	Replicate 2	Replicate 3	Replicate 4
	0,097999997	0,097900003	0,088299997	0,090400003

(540nm-690nm)-solvent control (540nm-690nm)

cocentration [M	Replicate 1	Replicate 2	Replicate 3	Replicate 4
1,00E-07	0,002100006	0,002299994	0,013900004	0,006499998
1,00E-06	0,0101	0,005599998	0,019000001	0,016099997
5,00E-06	0,036100008	0,041299999	0,0383	0,042800002
1,00E-05	0,051900007	0,064599991	0,067299999	0,0656
5,00E-05	0,084799998	0,09269999	0,089800008	0,096500002
1,00E-04	0,097800009	0,096599996	0,108499996	0,116299994
5,00E-04	0,174899988	0,123599991	0,145300008	0,154600002

(540nm-690nm)-solvent control E2 reference

cocentration [M	Replicate 1	Replicate 2	Replicate 3	Replicate 4
1,00E-09	0,509299986	0,480200022	0,531100027	0,515799977

Normalization (substance/1 nM E2)

cocentration [M	Replicate 1	Replicate 2	Replicate 3	Replicate 4	Mean
1,00E-07	0,41%	0,48%	2,62%	1,26%	**1,19%**
1,00E-06	1,98%	1,17%	3,58%	3,12%	**2,46%**
5,00E-06	7,09%	8,60%	7,21%	8,30%	**7,80%**
1,00E-05	10,19%	13,45%	12,67%	12,72%	**12,26%**
5,00E-05	16,65%	19,30%	16,91%	18,71%	**17,89%**
1,00E-04	19,20%	20,12%	20,43%	22,55%	**20,57%**
5,00E-04	34,34%	25,74%	27,36%	29,97%	**29,35%**

yeast-growth/solubility 690nm/solvent control 690nm

cocentration [M	Replicate 1	Replicate 2	Replicate 3	Replicate 4	Mean
1,00E-07	112,85%	106,46%	119,77%	104,90%	**110,99%**
1,00E-06	114,69%	99,66%	124,28%	111,23%	**112,47%**
5,00E-06	107,91%	116,88%	108,45%	109,88%	**110,78%**
1,00E-05	117,30%	111,59%	124,05%	115,20%	**117,03%**
5,00E-05	121,38%	111,24%	118,62%	110,05%	**115,32%**
1,00E-04	117,56%	107,51%	116,89%	124,61%	**116,64%**
5,00E-04	134,88%	108,28%	142,67%	143,67%	**132,38%**

5th experiment

540nm

cocentration [M	Replicate 1	Replicate 2	Replicate 3	Replicate 4
1,00E-07	0,755500019	0,806699991	0,811200023	0,82190001
1,00E-06	0,754199982	0,75999999	0,7245	0,707300007
5,00E-06	0,85680002	0,765299976	0,754199982	0,758400023
1,00E-05	0,810199976	0,769500017	0,792400002	0,780499995
5,00E-05	0,825200021	0,887099981	0,818499982	0,768400013
1,00E-04	0,744899988	0,765500009	0,815100014	0,747300029
5,00E-04	0,730099976	0,820599973	0,844299972	0,825699985

690nm

cocentration [M	Replicate 1	Replicate 2	Replicate 3	Replicate 4
1,00E-07	0,642099977	0,690400004	0,692200005	0,709500015
1,00E-06	0,626699984	0,634800017	0,602500021	0,587300003
5,00E-06	0,693799973	0,611699998	0,595399976	0,610099971
1,00E-05	0,640100002	0,606000006	0,629899979	0,614300013
5,00E-05	0,629899979	0,694100022	0,62440002	0,581900001
1,00E-04	0,547399998	0,56279999	0,603600025	0,537400007
5,00E-04	0,53549999	0,623700023	0,633899987	0,604200006

solvent control 690nm

	Replicate 1	Replicate 2	Replicate 3	Replicate 4
	0,658999979	0,520299971	0,576200008	0,555800021

540-690nm

cocentration [M	Replicate 1	Replicate 2	Replicate 3	Replicate 4
1,00E-07	0,113399997	0,116300002	0,119000003	0,112400003
1,00E-06	0,127499998	0,125200003	0,122000001	0,119999997
5,00E-06	0,163000003	0,153600007	0,158800006	0,148300007
1,00E-05	0,170100003	0,163499996	0,162499994	0,166199997
5,00E-05	0,195299998	0,193000004	0,194100007	0,1866
1,00E-04	0,197500005	0,202700004	0,211500004	0,209900007
5,00E-04	0,194600001	0,196899995	0,210299999	0,221499994

solvent control 540nm-690nm

	Replicate 1	Replicate 2	Replicate 3	Replicate 4
	0,122000001	0,103500001	0,110299997	0,110699996

(540nm-690nm)-solvent control (540nm-690nm)

cocentration [M	Replicate 1	Replicate 2	Replicate 3	Replicate 4
1,00E-07	-0,008600004	0,012800001	0,008700006	0,001700006
1,00E-06	0,005499996	0,021700002	0,011700004	0,009300001
5,00E-06	0,041000001	0,050100006	0,048500009	0,037600011
1,00E-05	0,048100002	0,059999995	0,052199997	0,055500001
5,00E-05	0,073299997	0,089500003	0,08380001	0,075900003
1,00E-04	0,075500004	0,099200003	0,101200007	0,09920001
5,00E-04	0,0726	0,093399994	0,100000001	0,110799998

(540nm-690nm)-solvent control E2 reference

cocentration [M	Replicate 1	Replicate 2	Replicate 3	Replicate 4
1,00E-09	0,44319997	0,4608	0,457400031	0,45190002

Normalization (substance/1 nM E2)

cocentration [M	Replicate 1	Replicate 2	Replicate 3	Replicate 4	Mean
1,00E-07	-1,94%	2,78%	1,90%	0,38%	**0,78%**
1,00E-06	1,24%	4,71%	2,56%	2,06%	**2,64%**
5,00E-06	9,25%	10,87%	10,60%	8,32%	**9,76%**
1,00E-05	10,85%	13,02%	11,41%	12,28%	**11,89%**
5,00E-05	16,54%	19,42%	18,32%	16,80%	**17,77%**
1,00E-04	17,04%	21,53%	22,13%	21,95%	**20,66%**
5,00E-04	16,38%	20,27%	21,86%	24,52%	**20,76%**

yeast-growth/solubility 690nm/solvent control 690nm

cocentration [M	Replicate 1	Replicate 2	Replicate 3	Replicate 4	Mean
1,00E-07	97,44%	132,69%	120,13%	127,65%	**119,48%**
1,00E-06	95,10%	122,01%	104,56%	105,67%	**106,83%**
5,00E-06	105,28%	117,57%	103,33%	109,77%	**108,99%**
1,00E-05	97,13%	116,47%	109,32%	110,53%	**108,36%**
5,00E-05	95,58%	133,40%	108,37%	104,70%	**110,51%**
1,00E-04	83,07%	108,17%	104,76%	96,69%	**98,17%**
5,00E-04	81,26%	119,87%	110,01%	108,71%	**104,96%**

cell-free 690nm

cocentration [M	Replicate 1	Replicate 2	Replicate 3	Replicate 4
1,00E-07	0,0372	0,036899999	0,036899999	0,0403
1,00E-06	0,0374	0,036899999	0,0383	0,038600001
5,00E-06	0,0381	0,0372	0,037500001	0,038600001
1,00E-05	0,040800001	0,038699999	0,0392	0,0392
5,00E-05	0,045400001	0,041900001	0,043900002	0,0429
1,00E-04	0,067199998	0,067299999	0,064900003	0,061900001
5,00E-04	0,278899997	0,204099998	0,233199999	0,188899994

cell-free solvent control 690nm

cocentration [M	Replicate 1	Replicate 2	Replicate 3	Replicate 4
	0,037500001	0,0361	0,036699999	0,036699999

cell-free (substance/solvent control)

cocentration [M	Replicate 1	Replicate 2	Replicate 3	Replicate 4	Mean
1,00E-07	99,20%	102,22%	100,54%	109,81%	**102,94%**
1,00E-06	99,73%	102,22%	104,36%	105,18%	**102,87%**
5,00E-06	101,60%	103,05%	102,18%	105,18%	**103,00%**
1,00E-05	108,80%	107,20%	106,81%	106,81%	**107,41%**
5,00E-05	121,07%	116,07%	119,62%	116,89%	**118,41%**
1,00E-04	179,20%	186,43%	176,84%	168,66%	**177,78%**
5,00E-04	743,73%	565,37%	635,42%	514,71%	**614,81%**

YES 2,4'-DDT

1st experient

540nm

cocentration [M]	Replicate 1	Replicate 2	Replicate 3	Replicate 4
1,00E-07	0,770500004	0,748600006	0,764800012	0,735300004
1,00E-06	0,7676	0,789099991	0,666400015	0,700399995
5,00E-06	0,818499982	0,802100003	0,779399991	0,766799986
1,00E-05	0,899900019	0,83069998	0,828499973	0,813600004
5,00E-05	0,936699986	0,910099983	0,882700026	0,903500021
1,00E-04	1,032199979	1,070700049	1,060299993	1,100299954
5,00E-04	1,19509995	0,944000006	1,076200008	1,03550005

690nm

cocentration [M]	Replicate 1	Replicate 2	Replicate 3	Replicate 4
1,00E-07	0,625800014	0,60589999	0,622300029	0,592100024
1,00E-06	0,57130003	0,584200025	0,477400005	0,510800004
5,00E-06	0,535899997	0,525699973	0,504800022	0,500699997
1,00E-05	0,567300022	0,514999986	0,50999999	0,506200016
5,00E-05	0,537500024	0,511200011	0,490999997	0,509199977
1,00E-04	0,577499986	0,588	0,594600022	0,617399991
5,00E-04	0,621699989	0,411199987	0,558200002	0,480800003

solvent control 690nm

	Replicate 1	Replicate 2	Replicate 3	Replicate 4
	0,459100008	0,586000025	0,433099985	0,491299987

540-690nm

cocentration [M]	Replicate 1	Replicate 2	Replicate 3	Replicate 4
1,00E-07	0,144700006	0,142700002	0,142499998	0,143299997
1,00E-06	0,196400002	0,204799995	0,188999996	0,189500004
5,00E-06	0,282700002	0,2764	0,274599999	0,266099989
1,00E-05	0,332599998	0,315699995	0,318500012	0,307300001
5,00E-05	0,399199992	0,398900002	0,391600013	0,394300014
1,00E-04	0,454600006	0,482600003	0,465700001	0,48300001
5,00E-04	0,573400021	0,532800019	0,518000007	0,5546

solvent control 540nm-690nm

	Replicate 1	Replicate 2	Replicate 3	Replicate 4
	0,125	0,1382	0,121299997	0,128900006

(540nm-690nm)-solvent control (540nm-690nm)

cocentration [M]	Replicate 1	Replicate 2	Replicate 3	Replicate 4
1,00E-07	0,366900012	0,385700002	0,391300008	0,420099989
1,00E-06	0,354500011	0,384499982	0,377899989	0,38760002
5,00E-06	0,354100004	0,363199994	0,373999998	0,360999987
1,00E-05	0,376999989	0,361399993	0,397100016	0,410999969
5,00E-05	0,428600028	0,409900025	0,425400004	0,487000003
1,00E-04	0,411899999	0,46010001	0,833100006	0,577799991
5,00E-04	0,583499983	0,628600016	0,680900022	0,739500001

(540nm-690nm)-solvent control E2 reference

cocentration [M]	Replicate 1	Replicate 2	Replicate 3	Replicate 4
1,00E-09	0,415099978	0,391199991	0,428800032	0,407999977

Normalization (substance/1 nM E2)

cocentration [M]	Replicate 1	Replicate 2	Replicate 3	Replicate 4	**Mean**
1,00E-07	88,39%	98,59%	91,25%	102,97%	**95,30%**
1,00E-06	85,40%	98,29%	88,13%	95,00%	**91,70%**
5,00E-06	85,30%	92,84%	87,22%	88,48%	**88,46%**
1,00E-05	90,82%	92,38%	92,61%	100,74%	**94,14%**
5,00E-05	103,25%	104,78%	99,21%	119,36%	**106,65%**
1,00E-04	99,23%	117,61%	194,29%	141,62%	**138,19%**
5,00E-04	140,57%	160,69%	158,79%	181,25%	**160,32%**

yeast-growth/solubility 690nm/solvent control 690nm

cocentration [M]	Replicate 1	Replicate 2	Replicate 3	Replicate 4	**Mean**
1,00E-07	136,31%	103,40%	143,69%	120,52%	**125,98%**
1,00E-06	124,44%	99,69%	110,23%	103,97%	**109,58%**
5,00E-06	116,73%	89,71%	116,56%	101,91%	**106,23%**
1,00E-05	123,57%	87,88%	117,76%	103,03%	**108,06%**
5,00E-05	125,79%	100,34%	137,29%	125,67%	**122,27%**
1,00E-04	117,08%	87,24%	113,37%	103,64%	**105,33%**
5,00E-04	135,42%	70,17%	128,88%	97,86%	**108,08%**

2nd experient

540nm

cocentration [M]	Replicate 1	Replicate 2	Replicate 3	Replicate 4
1,00E-07	0,717599988	0,72240001	0,70599997	0,666299999
1,00E-06	0,74940002	0,732500017	0,758000016	0,750699997
5,00E-06	0,783599973	0,753899992	0,817799985	0,753899992
1,00E-05	0,863600016	0,827499986	0,837499976	0,759599984
5,00E-05	0,914600015	0,926299989	0,80369997	0,805999994
1,00E-04	0,922399998	0,865499973	0,867799997	1,021999955
5,00E-04	0,880500019	1,039899945	0,952499986	1,074399948

690nm

cocentration [M]	Replicate 1	Replicate 2	Replicate 3	Replicate 4
1,00E-07	0,577000022	0,581700027	0,563399971	0,528900027
1,00E-06	0,551400006	0,538800001	0,559300005	0,564800024
5,00E-06	0,517700016	0,49180001	0,552900016	0,502399981
1,00E-05	0,555700004	0,513700008	0,527899981	0,472200006
5,00E-05	0,543600023	0,546299994	0,447100013	0,455500007
1,00E-04	0,508000016	0,4648	0,476700008	0,555700004
5,00E-04	0,4384	0,53490001	0,493600011	0,583299994

solvent control 690nm

	Replicate 1	Replicate 2	Replicate 3	Replicate 4
	0,338200003	0,463699996	0,623700023	0,533200026

540-690nm

cocentration [M]	Replicate 1	Replicate 2	Replicate 3	Replicate 4
1,00E-07	0,140499994	0,140799999	0,1426	0,137500003
1,00E-06	0,197999999	0,193800002	0,198699996	0,185900003
5,00E-06	0,265899986	0,261999995	0,264899999	0,25150001
1,00E-05	0,307900012	0,313800007	0,309700012	0,287400007
5,00E-05	0,371100008	0,379999995	0,356599987	0,350499988
1,00E-04	0,414400011	0,400700003	0,391200006	0,466300011
5,00E-04	0,442000002	0,504999995	0,458900005	0,491100013

solvent control 540nm-690nm

	Replicate 1	Replicate 2	Replicate 3	Replicate 4
	0,222100005	0,1285	0,139599994	0,137600005

(540nm-690nm)-solvent control (540nm-690nm)

cocentration [M]	Replicate 1	Replicate 2	Replicate 3	Replicate 4
1,00E-07	-0,08160001	0,0123	0,003000006	-0,000100002
1,00E-06	-0,024100006	0,065300003	0,059100002	0,048299998
5,00E-06	0,043799981	0,133499995	0,125300005	0,113900006
1,00E-05	0,085800007	0,185300007	0,170100018	0,149800003
5,00E-05	0,149000004	0,251499996	0,216999993	0,212899983
1,00E-04	0,192300007	0,272200003	0,251600012	0,328700006
5,00E-04	0,219899997	0,376499996	0,319300011	0,353500009

(540nm-690nm)-solvent control E2 reference

cocentration [M]	Replicate 1	Replicate 2	Replicate 3	Replicate 4
1,00E-09	0,284299985	0,386300012	0,336300001	0,354599983

Normalization (substance/1 nM E2)

cocentration [M]	Replicate 1	Replicate 2	Replicate 3	Replicate 4	**Mean**
1,00E-07	-28,70%	3,18%	0,89%	-0,03%	**-6,16%**
1,00E-06	-8,48%	16,90%	17,57%	13,62%	**9,91%**
5,00E-06	15,41%	34,56%	37,26%	32,12%	**29,84%**
1,00E-05	30,18%	47,97%	50,58%	42,24%	**42,74%**
5,00E-05	52,41%	65,10%	64,53%	60,04%	**60,52%**
1,00E-04	67,64%	70,46%	74,81%	92,70%	**76,40%**
5,00E-04	77,35%	97,46%	94,94%	99,69%	**92,36%**

yeast-growth/solubility 690nm/solvent control 690nm

cocentration [M]	Replicate 1	Replicate 2	Replicate 3	Replicate 4	**Mean**
1,00E-07	170,61%	125,45%	90,33%	99,19%	**121,40%**
1,00E-06	163,04%	116,20%	89,67%	105,93%	**118,71%**
5,00E-06	153,08%	106,06%	88,65%	94,22%	**110,50%**
1,00E-05	164,31%	110,78%	84,64%	88,56%	**112,07%**
5,00E-05	160,73%	117,81%	71,69%	85,43%	**108,91%**
1,00E-04	150,21%	100,24%	76,43%	104,22%	**107,77%**
5,00E-04	129,63%	115,35%	79,14%	109,40%	**108,38%**

3rd experiment

540nm

cocentration [M	Replicate 1	Replicate 2	Replicate 3	Replicate 4
1,00E-07	0,837400019	0,843800008	0,83859998	0,84009999
1,00E-06	0,938499987	0,886799991	1,021299958	0,87349999
5,00E-06	0,991400003	0,988799989	1,022600055	1,071699977
1,00E-05	1,011999965	1,027400017	1,101400018	1,098399997
5,00E-05	1,123399973	1,323799968	1,169700027	1,112499952
1,00E-04	1,128499985	1,074000031	1,212700009	1,136500001
5,00E-04	1,227100015	1,275500059	1,357599974	1,32130003

690nm

cocentration [M	Replicate 1	Replicate 2	Replicate 3	Replicate 4
1,00E-07	0,701399982	0,711899996	0,702199996	0,706399977
1,00E-06	0,705900013	0,668600023	0,767099977	0,649900019
5,00E-06	0,66170001	0,656099975	0,68779999	0,725799978
1,00E-05	0,628600001	0,641799986	0,699699998	0,709200025
5,00E-05	0,648599982	0,730199993	0,674300015	0,640399992
1,00E-04	0,61589998	0,558000028	0,679499984	0,617999971
5,00E-04	0,62529999	0,653800011	0,687600017	0,657199979

solvent control 690nm

	Replicate 1	Replicate 2	Replicate 3	Replicate 4
	0,685299993	0,717400014	0,693499982	0,686399996

540-690nm

cocentration [M	Replicate 1	Replicate 2	Replicate 3	Replicate 4
1,00E-07	0,135900006	0,131799996	0,136399999	0,133699998
1,00E-06	0,232600003	0,218199998	0,254299998	0,2236
5,00E-06	0,329699993	0,332700014	0,334800005	0,345800012
1,00E-05	0,383399993	0,385600001	0,401800007	0,389200002
5,00E-05	0,474700004	0,593500018	0,495400012	0,472200006
1,00E-04	0,512600005	0,516799986	0,533200026	0,51849997
5,00E-04	0,601800025	0,621699989	0,670000017	0,664099991

solvent control 540nm-690nm

	Replicate 1	Replicate 2	Replicate 3	Replicate 4
	0,121100001	0,120399997	0,117399998	0,118500002

(540nm-690nm)-solvent control (540nm-690nm)

cocentration [M	Replicate 1	Replicate 2	Replicate 3	Replicate 4
1,00E-07	0,014800005	0,011399999	0,019000001	0,015199997
1,00E-06	0,111500002	0,097800002	0,1369	0,105099998
5,00E-06	0,208599992	0,212300017	0,217400007	0,227300011
1,00E-05	0,262299992	0,265200004	0,284400009	0,2707
5,00E-05	0,353600003	0,473100021	0,378000014	0,353700005
1,00E-04	0,391500004	0,39639999	0,415800028	0,399999969
5,00E-04	0,480700023	0,501299992	0,552600019	0,54559999

(540nm-690nm)-solvent control E2 reference

cocentration [M	Replicate 1	Replicate 2	Replicate 3	Replicate 4
1,00E-09	0,527300008	0,5211	0,49220001	0,550800018

Normalization (substance/1 nM E2)

cocentration [M	Replicate 1	Replicate 2	Replicate 3	Replicate 4	**Mean**
1,00E-07	2,81%	2,19%	3,86%	2,76%	**2,90%**
1,00E-06	21,15%	18,77%	27,81%	19,08%	**21,70%**
5,00E-06	39,56%	40,74%	44,17%	41,27%	**41,43%**
1,00E-05	49,74%	50,89%	57,78%	49,15%	**51,89%**
5,00E-05	67,06%	90,79%	76,80%	64,22%	**74,72%**
1,00E-04	74,25%	76,07%	84,48%	72,62%	**76,85%**
5,00E-04	91,16%	96,20%	112,27%	99,06%	**99,67%**

yeast-growth/solubility 690nm/solvent control 690nm

cocentration [M	Replicate 1	Replicate 2	Replicate 3	Replicate 4	**Mean**
1,00E-07	102,35%	99,23%	101,25%	102,91%	**101,44%**
1,00E-06	103,01%	93,20%	110,61%	94,68%	**100,37%**
5,00E-06	96,56%	91,46%	99,18%	105,74%	**98,23%**
1,00E-05	91,73%	89,46%	100,89%	103,32%	**96,35%**
5,00E-05	94,64%	101,78%	97,23%	93,30%	**96,74%**
1,00E-04	89,87%	77,78%	97,98%	90,03%	**88,92%**
5,00E-04	91,24%	91,13%	99,15%	95,75%	**94,32%**

4th experiment

540nm

cocentration [M	Replicate 1	Replicate 2	Replicate 3	Replicate 4
1,00E-07	0,678300023	0,712199986	0,748099983	0,781199992
1,00E-06	0,737299979	0,806699991	0,788900018	0,868200004
5,00E-06	0,860800028	0,934000015	0,892199993	0,907999992
1,00E-05	0,892700016	1,093299985	0,981299996	1,031299949
5,00E-05	1,013700008	1,143399954	1,047199965	1,089400053
1,00E-04	1,191200018	1,163300037	1,12469995	1,107200027
5,00E-04	1,212700009	1,307600021	1,451699972	1,299399972

690nm

cocentration [M	Replicate 1	Replicate 2	Replicate 3	Replicate 4
1,00E-07	0,579200029	0,606000006	0,635299981	0,657400012
1,00E-06	0,552100003	0,614400029	0,59920001	0,667299986
5,00E-06	0,576099992	0,645799994	0,611699998	0,62650001
1,00E-05	0,550300002	0,727699995	0,627600014	0,684199989
5,00E-05	0,593699992	0,682500005	0,606999993	0,640699983
1,00E-04	0,686500013	0,672100008	0,659600019	0,619499981
5,00E-04	0,655900002	0,723699987	0,743900001	0,711000025

solvent control 690nm

	Replicate 1	Replicate 2	Replicate 3	Replicate 4
	0,623300016	0,719900012	0,650200009	0,578800023

540-690nm

cocentration [M	Replicate 1	Replicate 2	Replicate 3	Replicate 4
1,00E-07	0,099100001	0,106200002	0,1127	0,123800002
1,00E-06	0,185100004	0,192300007	0,189700007	0,200900003
5,00E-06	0,284700006	0,288100004	0,280600011	0,281500012
1,00E-05	0,342400014	0,36559999	0,353700012	0,34709999
5,00E-05	0,419999987	0,460900009	0,440200001	0,448599994
1,00E-04	0,504700005	0,491299987	0,46509999	0,487599999
5,00E-04	0,556800008	0,583800018	0,707799971	0,588400006

solvent control 540nm-690nm

	Replicate 1	Replicate 2	Replicate 3	Replicate 4
	0,104199998	0,107500002	0,099399999	0,092500001

(540nm-690nm)-solvent control (540nm-690nm)

cocentration [M	Replicate 1	Replicate 2	Replicate 3	Replicate 4
1,00E-07	-0,005099997	-0,0013	0,013300002	0,031300001
1,00E-06	0,080900006	0,084800005	0,090300009	0,108400002
5,00E-06	0,180500008	0,180600002	0,181200013	0,18900001
1,00E-05	0,238200016	0,258099988	0,254300013	0,254599988
5,00E-05	0,315799989	0,353400007	0,340800002	0,356099993
1,00E-04	0,400500007	0,383799985	0,365699992	0,395099998
5,00E-04	0,45260001	0,476300016	0,608399972	0,495900005

(540nm-690nm)-solvent control E2 reference

cocentration [M	Replicate 1	Replicate 2	Replicate 3	Replicate 4
1,00E-09	0,545099981	0,497699991	0,566499993	0,454399973

Normalization (substance/1 nM E2)

cocentration [M	Replicate 1	Replicate 2	Replicate 3	Replicate 4	**Mean**
1,00E-07	-0,94%	-0,26%	2,35%	6,89%	**2,01%**
1,00E-06	14,84%	17,04%	15,94%	23,86%	**17,92%**
5,00E-06	33,11%	36,29%	31,99%	41,59%	**35,74%**
1,00E-05	43,70%	51,86%	44,89%	56,03%	**49,12%**
5,00E-05	57,93%	71,01%	60,16%	78,37%	**66,87%**
1,00E-04	73,47%	77,11%	64,55%	86,95%	**75,52%**
5,00E-04	83,03%	95,70%	107,40%	109,13%	**98,82%**

yeast-growth/solubility 690nm/solvent control 690nm

cocentration [M	Replicate 1	Replicate 2	Replicate 3	Replicate 4	**Mean**
1,00E-07	92,92%	84,18%	97,71%	113,58%	**97,10%**
1,00E-06	88,58%	85,35%	92,16%	115,29%	**95,34%**
5,00E-06	92,43%	89,71%	94,08%	108,24%	**96,11%**
1,00E-05	88,29%	101,08%	96,52%	118,21%	**101,03%**
5,00E-05	95,25%	94,80%	93,36%	110,69%	**98,53%**
1,00E-04	110,14%	93,36%	101,45%	107,03%	**102,99%**
5,00E-04	105,23%	100,53%	114,41%	122,84%	**110,75%**

5th experiment

540nm

cocentration [M	Replicate 1	Replicate 2	Replicate 3	Replicate 4
1,00E-07	0,651300013	0,820200026	0,746800005	0,724799991
1,00E-06	0,808099985	0,807500005	0,84890002	0,762099981
5,00E-06	0,748199999	0,798900008	0,843500018	0,823700011
1,00E-05	0,927100003	0,922500014	0,896300018	0,892099977
5,00E-05	0,914099991	0,988300025	1,002099991	0,939599991
1,00E-04	0,976599991	0,992799997	0,999800026	1,099599957
5,00E-04	0,971300006	0,953299999	0,974900007	1,06219995

690nm

cocentration [M	Replicate 1	Replicate 2	Replicate 3	Replicate 4
1,00E-07	0,540799975	0,693499982	0,627099991	0,607500017
1,00E-06	0,614099979	0,615100026	0,648599982	0,581799984
5,00E-06	0,497099996	0,529999971	0,583700001	0,568599999
1,00E-05	0,612500012	0,598500013	0,582599998	0,590300024
5,00E-05	0,522400022	0,60710001	0,615700006	0,561600029
1,00E-04	0,567700028	0,58920002	0,585200012	0,657999992
5,00E-04	0,547299981	0,521600008	0,552999973	0,589299977

solvent control 690nm

	Replicate 1	Replicate 2	Replicate 3	Replicate 4
	0,532999992	0,60650003	0,612399995	0,582400024

540-690nm

cocentration [M	Replicate 1	Replicate 2	Replicate 3	Replicate 4
1,00E-07	0,1105	0,126699999	0,1197	0,117200002
1,00E-06	0,194000006	0,192399994	0,200299993	0,180399999
5,00E-06	0,251100004	0,26879999	0,259799987	0,255100012
1,00E-05	0,314599991	0,323900014	0,313600004	0,301800013
5,00E-05	0,3917	0,381199986	0,386400014	0,377999991
1,00E-04	0,408899993	0,403699994	0,414499998	0,441599995
5,00E-04	0,423999995	0,431699991	0,421900004	0,472900003

solvent control 540nm-690nm

	Replicate 1	Replicate 2	Replicate 3	Replicate 4
	0,101599999	0,108199999	0,104900002	0,103799999

(540nm-690nm)-solvent control (540nm-690nm)

cocentration [M	Replicate 1	Replicate 2	Replicate 3	Replicate 4
1,00E-07	0,008900002	0,0185	0,014799997	0,013400003
1,00E-06	0,092400007	0,084199995	0,095399991	0,0766
5,00E-06	0,149500005	0,160599992	0,154899985	0,151300013
1,00E-05	0,212999992	0,215700015	0,208700001	0,198000014
5,00E-05	0,290100001	0,272999987	0,281500012	0,274199992
1,00E-04	0,307299994	0,295499995	0,309599996	0,337799996
5,00E-04	0,322399996	0,323499992	0,317000002	0,369100004

(540nm-690nm)-solvent control E2 reference

cocentration [M	Replicate 1	Replicate 2	Replicate 3	Replicate 4
1,00E-09	0,478699975	0,45010002	0,478799999	0,461399972

Normalization (substance/1 nM E2)

cocentration [M	Replicate 1	Replicate 2	Replicate 3	Replicate 4	Mean
1,00E-07	1,86%	4,11%	3,09%	2,90%	**2,99%**
1,00E-06	19,30%	18,71%	19,92%	16,60%	**18,63%**
5,00E-06	31,23%	35,68%	32,35%	32,79%	**33,01%**
1,00E-05	44,50%	47,92%	43,59%	42,91%	**44,73%**
5,00E-05	60,60%	60,65%	58,79%	59,43%	**59,87%**
1,00E-04	64,19%	65,65%	64,66%	73,21%	**66,93%**
5,00E-04	67,35%	71,87%	66,21%	80,00%	**71,36%**

yeast-growth/solubility 690nm/solvent control 690nm

cocentration [M	Replicate 1	Replicate 2	Replicate 3	Replicate 4	Mean
1,00E-07	101,46%	114,34%	102,40%	104,31%	**105,63%**
1,00E-06	115,22%	101,42%	105,91%	99,90%	**105,61%**
5,00E-06	93,26%	87,39%	95,31%	97,63%	**93,40%**
1,00E-05	114,92%	98,68%	95,13%	101,36%	**102,52%**
5,00E-05	98,01%	100,10%	100,54%	96,43%	**98,77%**
1,00E-04	106,51%	97,15%	95,56%	112,98%	**103,05%**
5,00E-04	102,68%	86,00%	90,30%	101,18%	**95,04%**

cell-free 690nm

cocentration [M	Replicate 1	Replicate 2	Replicate 3	Replicate 4
1,00E-07	0,037099998	0,0372	0,037	0,040800001
1,00E-06	0,038800001	0,038699999	0,038699999	0,039299998
5,00E-06	0,038699999	0,037999999	0,043200001	0,0405
1,00E-05	0,039900001	0,0381	0,0403	0,040899999
5,00E-05	0,046100002	0,0451	0,056200001	0,045400001
1,00E-04	0,054699998	0,059	0,0568	0,059799999
5,00E-04	0,164100006	0,151800007	0,174600005	0,158700004

cell-free solvent control 690nm

cocentration [M	Replicate 1	Replicate 2	Replicate 3	Replicate 4
	0,037599999	0,038600001	0,037099998	0,037300002

cell-free (substance/solvent control)

cocentration [M	Replicate 1	Replicate 2	Replicate 3	Replicate 4	Mean
1,00E-07	98,67%	96,37%	99,73%	109,38%	**101,04%**
1,00E-06	103,19%	100,26%	104,31%	105,36%	**103,28%**
5,00E-06	102,93%	98,45%	116,44%	108,58%	**106,60%**
1,00E-05	106,12%	98,70%	108,63%	109,65%	**105,77%**
5,00E-05	122,61%	116,84%	151,48%	121,72%	**128,16%**
1,00E-04	145,48%	152,85%	153,10%	160,32%	**152,94%**
5,00E-04	436,44%	393,26%	470,62%	425,47%	**431,45%**

ERα CALUX pirimicarb

1st experient

luminescence

cocentration [M]	Replicate 1	Replicate 2	Replicate 3	Mean
1,00E-08	1160	1042	1122	**1108**
1,00E-07	1038	1180	1371	**1196**
1,00E-06	933	830	1306	**1023**
3,00E-06	913	737	1223	**958**
6,00E-06	906	499	936	**780**
1,00E-05	1056	710	809	**858**
3,00E-05	1189	1338	1364	**1297**
6,00E-05	1420	1158	1421	**1333**

solvent control

	Replicate 1	Replicate 2	Replicate 3	Replicate 4	Replicate 5	Replicate 6	Mean
	1309	1135	1136	1163	1132	1042	**1153**

sunbstance-solvent control

cocentration [M]	Mean
1,00E-08	**-45**
1,00E-07	**44**
1,00E-06	**-130**
3,00E-06	**-195**
6,00E-06	**-373**
1,00E-05	**-295**
3,00E-05	**144**
6,00E-05	**180**

E2 reference

cocentration [M]	Replicate 1	Replicate 2	Replicate 3	Mean
1,00E-10	15355	17180	17609	**16715**

E2 reference-solvent control

Mean
15562

Normalization (substance/0.1 nM E2)

cocentration [M]	**Mean**
1,00E-08	**-0,29%**
1,00E-07	**0,28%**
1,00E-06	**-0,83%**
3,00E-06	**-1,25%**
6,00E-06	**-2,39%**
1,00E-05	**-1,89%**
3,00E-05	**0,93%**
6,00E-05	**1,16%**

2nd experient

luminescence

cocentration [M]	Replicate 1	Replicate 2	Replicate 3	Mean
1,00E-08	1160	1042	1122	**1108**
1,00E-07	1038	1180	1371	**1196**
1,00E-06	933	830	1306	**1023**
3,00E-06	913	737	1223	**958**
6,00E-06	906	499	936	**780**
1,00E-05	1056	710	809	**858**
3,00E-05	1189	1338	1364	**1297**
6,00E-05	1420	1158	1421	**1333**

solvent control

	Replicate 1	Replicate 2	Replicate 3	Replicate 4	Replicate 5	Replicate 6	Mean
	952	770	880	806	902	721	**839**

sunbstance-solvent control

cocentration [M]	Mean
1,00E-08	**9**
1,00E-07	**-103**
1,00E-06	**-35**
3,00E-06	**-105**
6,00E-06	**-87**
1,00E-05	**-14**
3,00E-05	**203**
6,00E-05	**132**

E2 reference

cocentration [M]	Replicate 1	Replicate 2	Replicate 3	Mean
1,00E-10	13707	12852	12203	**12921**

E2 reference-solvent control

Mean
12082

Normalization (substance/0.1 nM E2)

cocentration [M]	**Mean**
1,00E-08	**0,07%**
1,00E-07	**-0,85%**
1,00E-06	**-0,29%**
3,00E-06	**-0,87%**
6,00E-06	**-0,72%**
1,00E-05	**-0,12%**
3,00E-05	**1,68%**
6,00E-05	**1,09%**

3rd experient

luminescence

cocentration [M	Replicate 1	Replicate 2	Replicate 3	Mean
1,00E-08	1423	1398	1558	**1460**
1,00E-07	1351	1256	1382	**1330**
1,00E-06	1186	1010	1220	**1139**
3,00E-06	1157	1091	1283	**1177**
6,00E-06	1289	1125	1273	**1229**
1,00E-05	1471	1064	1173	**1236**
3,00E-05	1474	1556	1534	**1521**
6,00E-05	1598	1489	1747	**1611**

solvent control

	Replicate 1	Replicate 2	Replicate 3	Replicate 4	Replicate 5	Replicate 6	Mean
	1471	770	1484	1367	1385	1437	**1319**

sunbstance-solvent control

cocentration [M	Mean
1,00E-08	**141**
1,00E-07	**11**
1,00E-06	**-180**
3,00E-06	**-142**
6,00E-06	**-90**
1,00E-05	**-83**
3,00E-05	**202**
6,00E-05	**292**

E2 reference

cocentration [M	Replicate 1	Replicate 2	Replicate 3	Mean
1,00E-10	15048	17253	17211	**16504**

E2 reference-solvent control

Mean
15185

Normalization (substance/0.1 nM E2)

cocentration [M	**Mean**
1,00E-08	**0,93%**
1,00E-07	**0,07%**
1,00E-06	**-1,19%**
3,00E-06	**-0,94%**
6,00E-06	**-0,59%**
1,00E-05	**-0,55%**
3,00E-05	**1,33%**
6,00E-05	**1,93%**

ERα CALUX propamocarb

1st experient

luminescence

cocentration [M]	Replicate 1	Replicate 2	Replicate 3	Mean
1,00E-08	1112	859	926	**966**
1,00E-07	1274	1038	860	**1057**
1,00E-06	3709	2967	3314	**3330**
3,00E-06	8923	8896	9391	**9070**
6,00E-06	12712	11286	12819	**12272**
1,00E-05	15148	13633	15668	**14816**
3,00E-05	17762	16897	18794	**17818**
6,00E-05	17247	16864	18778	**17630**

solvent control

	Replicate 1	Replicate 2	Replicate 3	Replicate 4	Replicate 5	Replicate 6	Mean
	1519	976	1027	883	900	1035	**1057**

substance-solvent control

cocentration [M]	Mean
1,00E-08	**-91**
1,00E-07	**1**
1,00E-06	**2273**
3,00E-06	**8013**
6,00E-06	**11216**
1,00E-05	**13760**
3,00E-05	**16761**
6,00E-05	**16573**

E2 reference

cocentration [M]	Replicate 1	Replicate 2	Replicate 3	Mean
1,00E-10	17288	18798	18903	**18330**

E2 reference-solvent control

Mean
17273

Normalization (substance/0.1 nM E2)

cocentration [M]	**Mean**
1,00E-08	**-0,53%**
1,00E-07	**0,00%**
1,00E-06	**13,16%**
3,00E-06	**46,39%**
6,00E-06	**64,93%**
1,00E-05	**79,66%**
3,00E-05	**97,04%**
6,00E-05	**95,95%**

excluded

2nd experient

luminescence

cocentration [M]	Replicate 1	Replicate 2	Replicate 3	Mean
1,00E-08	1222	1015	872	**1036**
1,00E-07	1174	1061	994	**1076**
1,00E-06	3509	3264	2960	**3244**
3,00E-06	8110	7216	6893	**7406**
6,00E-06	10572	10661	10243	**10492**
1,00E-05	13183	13069	12397	**12883**
3,00E-05	14729	14758	14113	**14533**
6,00E-05	12812	13997	13264	**13358**

solvent control

	Replicate 1	Replicate 2	Replicate 3	Replicate 4	Replicate 5	Replicate 6	Mean
	1339	888	940	906	1044	838	**993**

substance-solvent control

cocentration [M]	Mean
1,00E-08	**44**
1,00E-07	**84**
1,00E-06	**2252**
3,00E-06	**6414**
6,00E-06	**9500**
1,00E-05	**11891**
3,00E-05	**13541**
6,00E-05	**12365**

E2 reference

cocentration [M]	Replicate 1	Replicate 2	Replicate 3	Mean
1,00E-10	15474	15227	15546	**15416**

E2 reference-solvent control

Mean
14423

Normalization (substance/0.1 nM E2)

cocentration [M]	**Mean**
1,00E-08	**0,30%**
1,00E-07	**0,58%**
1,00E-06	**15,61%**
3,00E-06	**44,47%**
6,00E-06	**65,86%**
1,00E-05	**82,44%**
3,00E-05	**93,88%**
6,00E-05	**85,73%**

3rd experiment

luminescence

cocentration [M	Replicate 1	Replicate 2	Replicate 3	Mean
1,00E-08	1720	1587	1335	**1547**
1,00E-07	1807	1595	1462	**1621**
1,00E-06	3838	3998	3979	**3938**
3,00E-06	7658	9301	8107	**8355**
6,00E-06	11690	12460	12036	**12062**
1,00E-05	13590	15027	15449	**14689**
3,00E-05	15185	17086	17315	**16529**
6,00E-05	15468	16012	17262	**16247**

solvent control

	Replicate 1	Replicate 2	Replicate 3	Replicate 4	Replicate 5	Replicate 6	Mean
	1668	1352	1471	1079	1472	1468	**1418**

substance-solvent control

cocentration [M	Mean
1,00E-08	**129**
1,00E-07	**203**
1,00E-06	**2520**
3,00E-06	**6937**
6,00E-06	**10644**
1,00E-05	**13270**
3,00E-05	**15110**
6,00E-05	**14829**

E2 reference

cocentration [M	Replicate 1	Replicate 2	Replicate 3	Mean
1,00E-10	14764			**14764**
	15031			

E2 reference-solvent control

Mean
13346

Normalization (substance/0.1 nM E2)

cocentration [M	**Mean**
1,00E-08	**0,97%**
1,00E-07	**1,52%**
1,00E-06	**18,88%**
3,00E-06	**51,98%**
6,00E-06	**79,75%**
1,00E-05	**99,44%**
3,00E-05	**113,22%**
6,00E-05	**111,11%**

ERα CALUX chlorpyrifos

1st experient

luminescence

cocentration [M]	Replicate 1	Replicate 2	Replicate 3	Mean
1,00E-08	936	862	1119	**972**
1,00E-07	999	782	1116	**966**
1,00E-06	1434	1429	1315	**1393**
3,00E-06	2449	2830	2773	**2684**
6,00E-06	3955	4560	4697	**4404**
1,00E-05	6391	6852	7255	**6833**
3,00E-05	9190	9379	9304	**9291**
6,00E-05	11457	10346	9499	**10434**

solvent control

	Replicate 1	Replicate 2	Replicate 3	Replicate 4	Replicate 5	Replicate 6	Mean
	1519	976	1027	883	900	1035	**1057**

substance-solvent control

cocentration [M]	Mean
1,00E-08	**-84**
1,00E-07	**-91**
1,00E-06	**336**
3,00E-06	**1627**
6,00E-06	**3347**
1,00E-05	**5776**
3,00E-05	**8234**
6,00E-05	**9377**

E2 reference

cocentration [M]	Replicate 1	Replicate 2	Replicate 3	Mean
1,00E-10	17288	18798	18903	**18330**

E2 reference-solvent control

Mean
17273

Normalization (substance/0.1 nM E2)

cocentration [M]	**Mean**
1,00E-08	**-0,49%**
1,00E-07	**-0,53%**
1,00E-06	**1,95%**
3,00E-06	**9,42%**
6,00E-06	**19,38%**
1,00E-05	**33,44%**
3,00E-05	**47,67%**
6,00E-05	**54,29%**

2nd experient

luminescence

cocentration [M]	Replicate 1	Replicate 2	Replicate 3	Mean
1,00E-08	820	823	891	**845**
1,00E-07	1036	978	848	**954**
1,00E-06	1112	1107	975	**1065**
3,00E-06	1619	1453	2033	**1702**
6,00E-06	3319	2984	3105	**3136**
1,00E-05	4024	4721	4819	**4521**
3,00E-05	6147	6319	6287	**6251**
6,00E-05	6084	6635	7159	**6626**

solvent control

	Replicate 1	Replicate 2	Replicate 3	Replicate 4	Replicate 5	Replicate 6	Mean
	1339	888	940	906	1044	838	**993**

substance-solvent control

cocentration [M]	Mean
1,00E-08	**-148**
1,00E-07	**-39**
1,00E-06	**72**
3,00E-06	**709**
6,00E-06	**2144**
1,00E-05	**3529**
3,00E-05	**5259**
6,00E-05	**5634**

E2 reference

cocentration [M]	Replicate 1	Replicate 2	Replicate 3	Mean
1,00E-10	15474	15227	15546	**15416**

E2 reference-solvent control

Mean
14423

Normalization (substance/0.1 nM E2)

cocentration [M]	**Mean**
1,00E-08	**-1,02%**
1,00E-07	**-0,27%**
1,00E-06	**0,50%**
3,00E-06	**4,92%**
6,00E-06	**14,86%**
1,00E-05	**24,47%**
3,00E-05	**36,46%**
6,00E-05	**39,06%**

3rd experient

luminescence

cocentration [M	Replicate 1	Replicate 2	Replicate 3	Mean
1,00E-08	1295	1339	1490	**1375**
1,00E-07	1297	1333	1332	**1321**
1,00E-06	1602	1682	1303	**1529**
3,00E-06	2481	2531	2598	**2537**
6,00E-06	4754	4156	4514	**4475**
1,00E-05	6014	5319	5049	**5461**
3,00E-05	8343	7896	7893	**8044**
6,00E-05	7386	9057	7214	**7886**

solvent control

	Replicate 1	Replicate 2	Replicate 3	Replicate 4	Replicate 5	Replicate 6	Mean
	1668	1352	1471	1079	1472	1468	**1418**

substance-solvent control

cocentration [M	Mean
1,00E-08	**-44**
1,00E-07	**-98**
1,00E-06	**111**
3,00E-06	**1118**
6,00E-06	**3056**
1,00E-05	**4042**
3,00E-05	**6626**
6,00E-05	**6467**

E2 reference

cocentration [M	Replicate 1	Replicate 2	Replicate 3	Mean
1,00E-10	14764	15031	14758	**14764**

E2 reference-solvent control

Mean
13346

Normalization (substance/0.1 nM E2)

cocentration [M	**Mean**
1,00E-08	**-0,33%**
1,00E-07	**-0,73%**
1,00E-06	**0,83%**
3,00E-06	**8,38%**
6,00E-06	**22,90%**
1,00E-05	**30,29%**
3,00E-05	**49,65%**
6,00E-05	**48,46%**

ERα CALUX fenarimol

1st experient

luminescence

cocentration [M]	Replicate 1	Replicate 2	Replicate 3	Mean
1,00E-08	1032	978	1055	**1022**
1,00E-07	1182	896	1012	**1030**
1,00E-06	1541	1199	1147	**1296**
3,00E-06	3332	2849	2498	**2893**
6,00E-06	6016	5521	6817	**6118**
1,00E-05	9807	9440	9493	**9580**
3,00E-05	18603	18068	16362	**17678**
6,00E-05	20405	20276	22518	**21066**

solvent control

	Replicate 1	Replicate 2	Replicate 3	Replicate 4	Replicate 5	Replicate 6	Mean
	1483	1075	1088	790	904	1047	**1065**

substance-solvent control

cocentration [M]	Mean
1,00E-08	**-43**
1,00E-07	**-35**
1,00E-06	**231**
3,00E-06	**1829**
6,00E-06	**5054**
1,00E-05	**8516**
3,00E-05	**16613**
6,00E-05	**20002**

E2 reference

cocentration [M]	Replicate 1	Replicate 2	Replicate 3	Mean
1,00E-10	15811	16622	17147	**16527**

E2 reference-solvent control

Mean
15462

Normalization (substance/0.1 nM E2)

cocentration [M]	**Mean**
1,00E-08	**-0,28%**
1,00E-07	**-0,22%**
1,00E-06	**1,50%**
3,00E-06	**11,83%**
6,00E-06	**32,68%**
1,00E-05	**55,07%**
3,00E-05	**107,44%**
6,00E-05	**129,36%**

2nd experient

luminescence

cocentration [M]	Replicate 1	Replicate 2	Replicate 3	Mean
1,00E-08	1185	822	1028	**1012**
1,00E-07	1125	992	966	**1028**
1,00E-06	1575	1249	1162	**1329**
3,00E-06	2903	2717	2404	**2675**
6,00E-06	5836	5856	5700	**5797**
1,00E-05	9568	9057	8665	**9097**
3,00E-05	15869	15541	16757	**16056**
6,00E-05	14953	17037	18768	**16919**

solvent control

	Replicate 1	Replicate 2	Replicate 3	Replicate 4	Replicate 5	Replicate 6	Mean
	1056	1043	1093	1097	922	949	**1027**

substance-solvent control

cocentration [M]	Mean
1,00E-08	**-15**
1,00E-07	**1**
1,00E-06	**302**
3,00E-06	**1648**
6,00E-06	**4771**
1,00E-05	**8070**
3,00E-05	**15029**
6,00E-05	**15893**

E2 reference

cocentration [M]	Replicate 1	Replicate 2	Replicate 3	Mean
1,00E-10	15960	16777	15265	**16001**

E2 reference-solvent control

Mean
14974

Normalization (substance/0.1 nM E2)

cocentration [M]	**Mean**
1,00E-08	**-0,10%**
1,00E-07	**0,01%**
1,00E-06	**2,02%**
3,00E-06	**11,01%**
6,00E-06	**31,86%**
1,00E-05	**53,89%**
3,00E-05	**100,37%**
6,00E-05	**106,14%**

3rd experient

luminescence

cocentration [M]	Replicate 1	Replicate 2	Replicate 3	Mean
1,00E-08	1549	1396	1314	**1420**
1,00E-07	1567	1404	1418	**1463**
1,00E-06	1839	1782	1557	**1726**
3,00E-06	3157	2652	2475	**2761**
6,00E-06	6835	6563	6844	**6747**
1,00E-05	9522	11066	9956	**10181**
3,00E-05	16937	20025	19587	**18850**
6,00E-05	17594	19353	19206	**18718**

solvent control

	Replicate 1	Replicate 2	Replicate 3	Replicate 4	Replicate 5	Replicate 6	Mean
	1681	1741	1459	1439	1384	1348	**1509**

substance-solvent control

cocentration [M]	Mean
1,00E-08	**-89**
1,00E-07	**-46**
1,00E-06	**217**
3,00E-06	**1253**
6,00E-06	**5239**
1,00E-05	**8673**
3,00E-05	**17341**
6,00E-05	**17209**

E2 reference

cocentration [M]	Replicate 1	Replicate 2	Replicate 3	Mean
1,00E-10	16092	16580	16373	**16348**

E2 reference-solvent control

Mean
14840

Normalization (substance/0.1 nM E2)

cocentration [M]	**Mean**
1,00E-08	**-0,60%**
1,00E-07	**-0,31%**
1,00E-06	**1,46%**
3,00E-06	**8,44%**
6,00E-06	**35,30%**
1,00E-05	**58,44%**
3,00E-05	**116,86%**
6,00E-05	**115,97%**

ERα CALUX fludioxonil

1st experient

luminescence

cocentration [M]	Replicate 1	Replicate 2	Replicate 3	Mean
1,00E-08	1044	925	1034	**1001**
1,00E-07	935	788	1008	**910**
3,00E-07	1158	974	1152	**1095**
6,00E-07	1324	1312	1369	**1335**
1,00E-06	2093	1899	2111	**2034**
3,00E-06	7025	6643	6627	**6765**
6,00E-06	11383	10947	11323	**11218**
1,00E-05	11237	11026	10648	**10970**

solvent control

	Replicate 1	Replicate 2	Replicate 3	Replicate 4	Replicate 5	Replicate 6	Mean
	1056	1043	1093	1097	922	949	**1027**

substance-solvent control

cocentration [M]	Mean
1,00E-08	**-26**
1,00E-07	**-116**
3,00E-07	**68**
6,00E-07	**308**
1,00E-06	**1008**
3,00E-06	**5738**
6,00E-06	**10191**
1,00E-05	**9944**

E2 reference

cocentration [M]	Replicate 1	Replicate 2	Replicate 3	Mean
1,00E-10	15960	16777	15265	**16001**

E2 reference-solvent control

Mean
14974

Normalization (substance/0.1 nM E2)

cocentration [M]	**Mean**
1,00E-08	**-0,17%**
1,00E-07	**-0,78%**
3,00E-07	**0,45%**
6,00E-07	**2,06%**
1,00E-06	**6,73%**
3,00E-06	**38,32%**
6,00E-06	**68,06%**
1,00E-05	**66,41%**

2nd experient

luminescence

cocentration [M]	Replicate 1	Replicate 2	Replicate 3	Mean
1,00E-08	1363	1226	1402	**1330**
1,00E-07	1256	1179	1415	**1283**
3,00E-07	1555	1402	1558	**1505**
6,00E-07	1987	1607	2231	**1942**
1,00E-06	2852	2404	3131	**2796**
3,00E-06	7611	7827	6683	**7374**
6,00E-06	11302	11227	12132	**11554**
1,00E-05	11549	11815	12098	**11821**

solvent control

	Replicate 1	Replicate 2	Replicate 3	Replicate 4	Replicate 5	Replicate 6	Mean
	1681	1741	1459	1439	1384	1348	**1509**

substance-solvent control

cocentration [M]	Mean
1,00E-08	**-178**
1,00E-07	**-225**
3,00E-07	**-4**
6,00E-07	**433**
1,00E-06	**1287**
3,00E-06	**5865**
6,00E-06	**10045**
1,00E-05	**10312**

E2 reference

cocentration [M]	Replicate 1	Replicate 2	Replicate 3	Mean
1,00E-10	16092	16580	16373	**16348**

E2 reference-solvent control

Mean
14840

Normalization (substance/0.1 nM E2)

cocentration [M]	**Mean**
1,00E-08	**-1,20%**
1,00E-07	**-1,52%**
3,00E-07	**-0,02%**
6,00E-07	**2,92%**
1,00E-06	**8,67%**
3,00E-06	**39,52%**
6,00E-06	**67,69%**
1,00E-05	**69,49%**

3rd experiment

luminescence

cocentration [M]	Replicate 1	Replicate 2	Replicate 3	Mean
1,00E-08	765	888	919	**857**
1,00E-07	929	781	916	**875**
3,00E-07	867	877	700	**815**
6,00E-07	1056	1001	1162	**1073**
1,00E-06	1770	1633	2006	**1803**
3,00E-06	4738	5296	4806	**4947**
6,00E-06	7501	7673	7500	**7558**
1,00E-05	8068	7641	7105	**7605**

solvent control

	Replicate 1	Replicate 2	Replicate 3	Replicate 4	Replicate 5	Replicate 6	Mean
	1269	770	977	899	862	860	**940**

substance-solvent control

cocentration [M]	Mean
1,00E-08	**-82**
1,00E-07	**-64**
3,00E-07	**-125**
6,00E-07	**134**
1,00E-06	**864**
3,00E-06	**4007**
6,00E-06	**6619**
1,00E-05	**6665**

E2 reference

cocentration [M]	Replicate 1	Replicate 2	Replicate 3	Mean
1,00E-10	10226	11834	11787	**11282**

E2 reference-solvent control

Mean
10343

Normalization (substance/0.1 nM E2)

cocentration [M]	**Mean**
1,00E-08	**-0,79%**
1,00E-07	**-0,62%**
3,00E-07	**-1,21%**
6,00E-07	**1,29%**
1,00E-06	**8,35%**
3,00E-06	**38,74%**
6,00E-06	**63,99%**
1,00E-05	**64,44%**

ERα CALUX fenhexamid

1st experient

luminescence

cocentration [M]	Replicate 1	Replicate 2	Replicate 3	Mean
1,00E-08	1090	633	622	**782**
1,00E-07	699	595	566	**620**
1,00E-06	990	685	620	**765**
3,00E-06	2516	1075	1631	**1741**
6,00E-06	7271	3671	4522	**5155**
1,00E-05	7879	8521	6357	**7586**
3,00E-05	19032	17928	16504	**17821**
6,00E-05	20170	19141	21101	**20137**

solvent control

	Replicate 1	Replicate 2	Replicate 3	Replicate 4	Replicate 5	Replicate 6	Mean
	931	851	806	814	790	1042	**872**

substance-solvent control

cocentration [M]	Mean
1,00E-08	**-41**
1,00E-07	**-202**
1,00E-06	**-57**
3,00E-06	**918**
6,00E-06	**4332**
1,00E-05	**6763**
3,00E-05	**16999**
6,00E-05	**19315**

E2 reference

cocentration [M]	Replicate 1	Replicate 2	Replicate 3	Mean
1,00E-10	13079	14913	13825	**13939**

E2 reference-solvent control

Mean
13067

Normalization (substance/0.1 nM E2)

cocentration [M]	Mean
1,00E-08	**-0,31%**
1,00E-07	**-1,55%**
1,00E-06	**-0,44%**
3,00E-06	**7,03%**
6,00E-06	**33,16%**
1,00E-05	**51,76%**
3,00E-05	**130,09%**
6,00E-05	**147,82%**

2nd experient

luminescence

cocentration [M]	Replicate 1	Replicate 2	Replicate 3	Mean
1,00E-08	1243	1043	1024	**1103**
1,00E-07	1227	972	1037	**1079**
1,00E-06	1738	1107	1133	**1326**
3,00E-06	2768	2305	2200	**2424**
6,00E-06	7297	6635	6178	**6703**
1,00E-05	10681	10470	11581	**10911**
3,00E-05	19842	17326	19362	**18843**
6,00E-05	18666	19478	19990	**19378**

solvent control

	Replicate 1	Replicate 2	Replicate 3	Replicate 4	Replicate 5	Replicate 6	Mean
	1035	1132	973	1050	944	899	**1006**

substance-solvent control

cocentration [M]	Mean
1,00E-08	**98**
1,00E-07	**73**
1,00E-06	**321**
3,00E-06	**1419**
6,00E-06	**5698**
1,00E-05	**9905**
3,00E-05	**17838**
6,00E-05	**18373**

E2 reference

cocentration [M]	Replicate 1	Replicate 2	Replicate 3	Mean
1,00E-10	15659	15043	16403	**15702**

E2 reference-solvent control

Mean
14696

Normalization (substance/0.1 nM E2)

cocentration [M]	Mean
1,00E-08	**0,67%**
1,00E-07	**0,50%**
1,00E-06	**2,18%**
3,00E-06	**9,65%**
6,00E-06	**38,77%**
1,00E-05	**67,40%**
3,00E-05	**121,38%**
6,00E-05	**125,02%**

3rd experient

luminescence

cocentration [M	Replicate 1	Replicate 2	Replicate 3	Mean
1,00E-08	1462	1402	1161	**1342**
1,00E-07	1377	1297	1123	**1266**
1,00E-06	1634	1549	1476	**1553**
3,00E-06	2958	2811	2498	**2756**
6,00E-06	6332	6429	6873	**6545**
1,00E-05	9469	10548	10869	**10295**
3,00E-05	14956	18018	18323	**17099**
6,00E-05	16626	17452	18705	**17594**

solvent control

	Replicate 1	Replicate 2	Replicate 3	Replicate 4	Replicate 5	Replicate 6	Mean
	1738	1234	1376	1113	1261	1108	**1305**

substance-solvent control

cocentration [M	Mean
1,00E-08	**37**
1,00E-07	**-39**
1,00E-06	**248**
3,00E-06	**1451**
6,00E-06	**5240**
1,00E-05	**8990**
3,00E-05	**15794**
6,00E-05	**16289**

E2 reference

cocentration [M	Replicate 1	Replicate 2	Replicate 3	Mean
1,00E-10	12774	13136	13487	**13132**

E2 reference-solvent control

Mean
11827

Normalization (substance/0.1 nM E2)

cocentration [M	Mean
1,00E-08	**0,31%**
1,00E-07	**-0,33%**
1,00E-06	**2,10%**
3,00E-06	**12,27%**
6,00E-06	**44,30%**
1,00E-05	**76,01%**
3,00E-05	**133,54%**
6,00E-05	**137,73%**

ERα CALUX 4,4'-DDT

1st experient

luminescence

cocentration [M]	Replicate 1	Replicate 2	Replicate 3	Mean
1,00E-08	1097	785	966	**949**
1,00E-07	1021	1000	894	**972**
6,00E-07	1299	1307	1427	**1344**
1,00E-06	2256	2123	2460	**2280**
3,00E-06	7166	6905	7104	**7058**
6,00E-06	9591	9776	10444	**9937**
1,00E-05	11931	11710	11772	**11804**
3,00E-05	10934	11388	10801	**11041**

solvent control

	Replicate 1	Replicate 2	Replicate 3	Replicate 4	Replicate 5	Replicate 6	Mean
	1035	1132	973	1050	944	899	**1006**

substance-solvent control

cocentration [M]	Mean
1,00E-08	**-56**
1,00E-07	**-34**
6,00E-07	**339**
1,00E-06	**1274**
3,00E-06	**6053**
6,00E-06	**8932**
1,00E-05	**10799**
3,00E-05	**10036**

E2 reference

cocentration [M]	Replicate 1	Replicate 2	Replicate 3	Mean
1,00E-10	15659	15043	16403	**15702**

E2 reference-solvent control

Mean
14696

Normalization (substance/0.1 nM E2)

cocentration [M]	**Mean**
1,00E-08	**-0,38%**
1,00E-07	**-0,23%**
6,00E-07	**2,31%**
1,00E-06	**8,67%**
3,00E-06	**41,19%**
6,00E-06	**60,77%**
1,00E-05	**73,48%**
3,00E-05	**68,29%**

excluded

2nd experient

luminescence

cocentration [M]	Replicate 1	Replicate 2	Replicate 3	Mean
1,00E-08	1386	1119	1302	**1269**
1,00E-07	1342	1338	1216	**1299**
6,00E-07	2052	1927	1470	**1816**
1,00E-06	2819	2171	2178	**2389**
3,00E-06	8099	6615	7159	**7291**
6,00E-06	10120	9701	7820	**9214**
1,00E-05	11414	10357	9456	**10409**
3,00E-05	10569	10703	10352	**10541**

solvent control

	Replicate 1	Replicate 2	Replicate 3	Replicate 4	Replicate 5	Replicate 6	Mean
	1738	1234	1376	1113	1261	1108	**1305**

substance-solvent control

cocentration [M]	Mean
1,00E-08	**-36**
1,00E-07	**-6**
6,00E-07	**511**
1,00E-06	**1084**
3,00E-06	**5986**
6,00E-06	**7909**
1,00E-05	**9104**
3,00E-05	**9236**

E2 reference

cocentration [M]	Replicate 1	Replicate 2	Replicate 3	Mean
1,00E-10	12774	13136	13487	**13132**

E2 reference-solvent control

Mean
11827

Normalization (substance/0.1 nM E2)

cocentration [M]	**Mean**
1,00E-08	**-0,30%**
1,00E-07	**-0,05%**
6,00E-07	**4,32%**
1,00E-06	**9,17%**
3,00E-06	**50,61%**
6,00E-06	**66,87%**
1,00E-05	**76,97%**
3,00E-05	**78,09%**

3rd experient

luminescence

cocentration [M	Replicate 1	Replicate 2	Replicate 3	Mean
1,00E-08	1232	1191	1081	**1168**
1,00E-07	1371	1024	989	**1128**
6,00E-07	1697	1567	1378	**1547**
1,00E-06	2423	2196	1756	**2125**
3,00E-06	7344	6869	6768	**6994**
6,00E-06	9202	8632	8957	**8930**
1,00E-05	11387	10378	10397	**10721**
3,00E-05	10458	9920	10100	**10159**

solvent control

	Replicate 1	Replicate 2	Replicate 3	Replicate 4	Replicate 5	Replicate 6	Mean
	1301	1077	1020	1101	1034	1270	**1107**

substance-solvent control

cocentration [M	Mean
1,00E-08	**34**
1,00E-07	**-6**
6,00E-07	**414**
1,00E-06	**991**
3,00E-06	**5860**
6,00E-06	**7797**
1,00E-05	**9587**
3,00E-05	**9026**

E2 reference

cocentration [M	Replicate 1	Replicate 2	Replicate 3	Mean
1,00E-10	13610	14251	14609	**14157**

E2 reference-solvent control

Mean
13050

Normalization (substance/0.1 nM E2)

cocentration [M	**Mean**
1,00E-08	**0,26%**
1,00E-07	**-0,04%**
6,00E-07	**3,17%**
1,00E-06	**7,60%**
3,00E-06	**44,90%**
6,00E-06	**59,74%**
1,00E-05	**73,46%**
3,00E-05	**69,16%**

ERα CALUX 2,4'-DDT

1st experient

luminescence

cocentration [M]	Replicate 1	Replicate 2	Replicate 3	Mean
1,00E-08	1555	1096	1076	**1242**
1,00E-07	2839	2418	2478	**2578**
6,00E-07	14102	13124	14322	**13849**
1,00E-06	15778	16205	16094	**16026**
3,00E-06	19192	19778	19739	**19570**
6,00E-06	19246	19519	18492	**19086**
1,00E-05	19798	17593	19287	**18893**
3,00E-05	14569	14008	14718	**14432**

solvent control

	Replicate 1	Replicate 2	Replicate 3	Mean
	1226	1130	1127	**1161**

substance-solvent control

cocentration [M]	Mean
1,00E-08	**81**
1,00E-07	**1417**
6,00E-07	**12688**
1,00E-06	**14865**
3,00E-06	**18409**
6,00E-06	**17925**
1,00E-05	**17732**
3,00E-05	**13271**

E2 reference

cocentration [M]	Replicate 1	Replicate 2	Replicate 3	Mean
1,00E-10	15937	14900	16221	**15686**

E2 reference-solvent control

Mean
14525

Normalization (substance/0.1 nM E2)

cocentration [M]	**Mean**
1,00E-08	**0,56%**
1,00E-07	**9,76%**
6,00E-07	**87,36%**
1,00E-06	**102,34%**
3,00E-06	**126,74%**
6,00E-06	**123,41%**
1,00E-05	**122,08%**
3,00E-05	**91,36%**

excluded

2nd experient

luminescence

cocentration [M]	Replicate 1	Replicate 2	Replicate 3	Mean
1,00E-08	1570	1652	1395	**1539**
1,00E-07	3095	3289	2995	**3126**
6,00E-07	10647	13318	13726	**12564**
1,00E-06	14096	14761	17464	**15440**
3,00E-06	17217	19389	20457	**19021**
6,00E-06	16635	19432	21481	**19183**
1,00E-05	15108	18136	19555	**17600**
3,00E-05	12911	15533	16161	**14868**

solvent control

	Replicate 1	Replicate 2	Replicate 3	Mean
	1645	1252	1261	**1386**

substance-solvent control

cocentration [M]	Mean
1,00E-08	**153**
1,00E-07	**1740**
6,00E-07	**11178**
1,00E-06	**14054**
3,00E-06	**17635**
6,00E-06	**17797**
1,00E-05	**16214**
3,00E-05	**13482**

E2 reference

cocentration [M]	Replicate 1	Replicate 2	Replicate 3	Mean
1,00E-10	13114	13513	14419	**13682**

E2 reference-solvent control

Mean
12296

Normalization (substance/0.1 nM E2)

cocentration [M]	**Mean**
1,00E-08	**1,24%**
1,00E-07	**14,15%**
6,00E-07	**90,90%**
1,00E-06	**114,30%**
3,00E-06	**143,42%**
6,00E-06	**144,74%**
1,00E-05	**131,86%**
3,00E-05	**109,65%**

3rd experient

luminescence

cocentration [M	Replicate 1	Replicate 2	Replicate 3	Mean
1,00E-08	1029	951	1200	**1060**
1,00E-07	2480	2210	2412	**2367**
6,00E-07	11804	11403	10545	**11251**
1,00E-06	13519	14500	13080	**13700**
3,00E-06	16074	16694	16427	**16398**
6,00E-06	16805	17128	15738	**16557**
1,00E-05	15745	17622	14803	**16057**
3,00E-05	12195	11945	11793	**11978**

solvent control

	Replicate 1	Replicate 2	Replicate 3	Replicate 4	Replicate 5	Replicate 6	Mean
	1301	1077	1020	1101	1034	1270	**1134**

substance-solvent control

cocentration [M	Mean
1,00E-08	**-74**
1,00E-07	**1234**
6,00E-07	**10117**
1,00E-06	**12566**
3,00E-06	**15265**
6,00E-06	**15423**
1,00E-05	**14923**
3,00E-05	**10844**

E2 reference

cocentration [M	Replicate 1	Replicate 2	Replicate 3	Mean
1,00E-10	13610	14251	14609	**14157**

E2 reference-solvent control

Mean
13023

Normalization (substance/0.1 nM E2)

cocentration [M	**Mean**
1,00E-08	**-0,57%**
1,00E-07	**9,47%**
6,00E-07	**77,69%**
1,00E-06	**96,49%**
3,00E-06	**117,21%**
6,00E-06	**118,43%**
1,00E-05	**114,59%**
3,00E-05	**83,27%**

ERβ CALUX pirimicarb

1st experient

luminescence

cocentration [M]	Replicate 1	Replicate 2	Replicate 3	Mean
1,00E-08	131	171	139	**147**
1,00E-07	163	137	133	**144**
1,00E-06	205	192	196	**198**
3,00E-06	1055	846	910	**937**
6,00E-06	1881	1857	1962	**1900**
1,00E-05	2171	2791	2095	**2352**
3,00E-05	2568	2339	2224	**2377**
6,00E-05	2201	2085	2296	**2194**

solvent control

	Replicate 1	Replicate 2	Replicate 3	Replicate 4	Replicate 5	Replicate 6	Mean
	99	770	142	143	142	165	**244**

substance-solvent control

cocentration [M]	Mean
1,00E-08	**-104**
1,00E-07	**-108**
1,00E-06	**-99**
3,00E-06	**-108**
6,00E-06	**-107**
1,00E-05	**-120**
3,00E-05	**-100**
6,00E-05	**-88**

E2 reference

cocentration [M]	Replicate 1	Replicate 2	Replicate 3	Mean
3,00E-08	2201	2085	2296	**2194**

E2 reference-solvent control

Mean
1951

Normalization (substance/30 nM E2)

cocentration [M]	**Mean**
1,00E-08	**-5,34%**
1,00E-07	**-5,51%**
1,00E-06	**-5,07%**
3,00E-06	**-5,51%**
6,00E-06	**-5,48%**
1,00E-05	**-6,13%**
3,00E-05	**-5,14%**
6,00E-05	**-4,49%**

2nd experient

luminescence

cocentration [M]	Replicate 1	Replicate 2	Replicate 3	Mean
1,00E-08	183	213	187	**194**
1,00E-07	203	181	198	**194**
1,00E-06	222	218	197	**212**
3,00E-06	512	733	645	**630**
6,00E-06	721	1046	1263	**1010**
1,00E-05	1415	1520	1730	**1555**
3,00E-05	1489	1560	1570	**1540**
6,00E-05	1593	1851	1583	**1676**

solvent control

	Replicate 1	Replicate 2	Replicate 3	Replicate 4	Replicate 5	Replicate 6	Mean
	177	770	217	204	175	177	**287**

substance-solvent control

cocentration [M]	Mean
1,00E-08	**-106**
1,00E-07	**-105**
1,00E-06	**-95**
3,00E-06	**-108**
6,00E-06	**-80**
1,00E-05	**-84**
3,00E-05	**-107**
6,00E-05	**-101**

E2 reference

cocentration [M]	Replicate 1	Replicate 2	Replicate 3	Mean
3,00E-08	1593	1851	1583	**1676**

E2 reference-solvent control

Mean
1389

Normalization (substance/30 nM E2)

cocentration [M]	**Mean**
1,00E-08	**-7,61%**
1,00E-07	**-7,56%**
1,00E-06	**-6,82%**
3,00E-06	**-7,75%**
6,00E-06	**-5,76%**
1,00E-05	**-6,07%**
3,00E-05	**-7,68%**
6,00E-05	**-7,30%**

3rd experient

luminescence

cocentration [M	Replicate 1	Replicate 2	Replicate 3	Mean
1,00E-08	123	122	131	**125**
1,00E-07	143	123	158	**141**
1,00E-06	143	144	145	**144**
3,00E-06	129	100	153	**127**
6,00E-06	117	135	120	**124**
1,00E-05	157	115	112	**128**
3,00E-05	109	118	116	**114**
6,00E-05	134	126	173	**144**

solvent control

	Replicate 1	Replicate 2	Replicate 3	Replicate 4	Replicate 5	Replicate 6	Mean
	125	770	157	195	129	158	**256**

substance-solvent control

cocentration [M	Mean
1,00E-08	**-130**
1,00E-07	**-114**
1,00E-06	**-112**
3,00E-06	**-128**
6,00E-06	**-132**
1,00E-05	**-128**
3,00E-05	**-141**
6,00E-05	**-111**

E2 reference

cocentration [M	Replicate 1	Replicate 2	Replicate 3	Mean
3,00E-08	4695	3920	3620	**4078**

E2 reference-solvent control

Mean
3823

Normalization (substance/30 nM E2)

cocentration [M	**Mean**
1,00E-08	**-3,41%**
1,00E-07	**-2,99%**
1,00E-06	**-2,92%**
3,00E-06	**-3,36%**
6,00E-06	**-3,44%**
1,00E-05	**-3,34%**
3,00E-05	**-3,70%**
6,00E-05	**-2,91%**

ERβ CALUX propamocarb

1st experient

luminescence

cocentration [M]	Replicate 1	Replicate 2	Replicate 3	Mean
1,00E-08	138	156	132	**142**
1,00E-07	129	155	99	**128**
1,00E-06	165	126	167	**153**
3,00E-06	144	171	189	**168**
6,00E-06	292	316	224	**277**
1,00E-05	455	272	286	**338**
3,00E-05	957	972	1060	**996**
6,00E-05	1206	1474	1407	**1362**

solvent control

	Replicate 1	Replicate 2	Replicate 3	Replicate 4	Replicate 5	Replicate 6	Mean
	181	127	121	138	139	145	**142**

substance-solvent control

cocentration [M]	Mean
1,00E-08	**0**
1,00E-07	**-14**
1,00E-06	**11**
3,00E-06	**26**
6,00E-06	**136**
1,00E-05	**196**
3,00E-05	**855**
6,00E-05	**1221**

E2 reference

cocentration [M]	Replicate 1	Replicate 2	Replicate 3	Mean
3,00E-08	3802	3434	3573	**3603**

E2 reference-solvent control

Mean
3461

Normalization (substance/30 nM E2)

cocentration [M]	**Mean**
1,00E-08	**0,00%**
1,00E-07	**-0,41%**
1,00E-06	**0,31%**
3,00E-06	**0,76%**
6,00E-06	**3,91%**
1,00E-05	**5,66%**
3,00E-05	**24,69%**
6,00E-05	**35,26%**

2nd experient

luminescence

cocentration [M]	Replicate 1	Replicate 2	Replicate 3	Mean
1,00E-08	154	119	116	**130**
1,00E-07	127	133	137	**132**
1,00E-06	140	122	127	**130**
3,00E-06	132	137	155	**141**
6,00E-06	212	150	211	**191**
1,00E-05	189	170	160	**173**
3,00E-05	495	504	481	**493**
6,00E-05	805	715	978	**833**

solvent control

	Replicate 1	Replicate 2	Replicate 3	Replicate 4	Replicate 5	Replicate 6	Mean
	154	139	123	139	130	169	**142**

substance-solvent control

cocentration [M]	Mean
1,00E-08	**-13**
1,00E-07	**-10**
1,00E-06	**-13**
3,00E-06	**-1**
6,00E-06	**49**
1,00E-05	**31**
3,00E-05	**351**
6,00E-05	**690**

E2 reference

cocentration [M]	Replicate 1	Replicate 2	Replicate 3	Mean
3,00E-08	2365	2869	2848	**2694**

E2 reference-solvent control

Mean
2552

Normalization (substance/30 nM E2)

cocentration [M]	**Mean**
1,00E-08	**-0,50%**
1,00E-07	**-0,39%**
1,00E-06	**-0,50%**
3,00E-06	**-0,04%**
6,00E-06	**1,91%**
1,00E-05	**1,20%**
3,00E-05	**13,76%**
6,00E-05	**27,05%**

3rd experient

luminescence

cocentration [M	Replicate 1	Replicate 2	Replicate 3	Mean
1,00E-08	183	181	186	**183**
1,00E-07	204	206	202	**204**
1,00E-06	224	174	206	**201**
3,00E-06	203	215	196	**205**
6,00E-06	173	178	197	**183**
1,00E-05	218	229	202	**216**
3,00E-05	381	294	398	**358**
6,00E-05	566	702	513	**594**

solvent control

	Replicate 1	Replicate 2	Replicate 3	Replicate 4	Replicate 5	Replicate 6	Mean
	245	215	208	225	188	246	**221**

substance-solvent control

cocentration [M	Mean
1,00E-08	**-38**
1,00E-07	**-17**
1,00E-06	**-20**
3,00E-06	**-17**
6,00E-06	**-39**
1,00E-05	**-5**
3,00E-05	**137**
6,00E-05	**373**

E2 reference

cocentration [M	Replicate 1	Replicate 2	Replicate 3	Mean
3,00E-08	1469	1383	1391	**1414**

E2 reference-solvent control

Mean
1193

Normalization (substance/30 nM E2)

cocentration [M	**Mean**
1,00E-08	**-3,17%**
1,00E-07	**-1,44%**
1,00E-06	**-1,66%**
3,00E-06	**-1,38%**
6,00E-06	**-3,23%**
1,00E-05	**-0,41%**
3,00E-05	**11,44%**
6,00E-05	**31,22%**

ERα CALUX pirimicarb

1st experient

luminescence

cocentration [M]	Replicate 1	Replicate 2	Replicate 3	Mean
1,00E-08	135	149	121	**135**
1,00E-07	131	181	157	**156**
1,00E-06	163	162	142	**156**
3,00E-06	163	201	170	**178**
6,00E-06	230	164	237	**210**
1,00E-05	157	195	182	**178**
3,00E-05	239	192	267	**233**
6,00E-05	194	174	243	**204**

solvent control

	Replicate 1	Replicate 2	Replicate 3	Replicate 4	Replicate 5	Replicate 6	Mean
	181	127	121	138	139	145	**142**

substance-solvent control

cocentration [M]	Mean
1,00E-08	**-7**
1,00E-07	**15**
1,00E-06	**14**
3,00E-06	**36**
6,00E-06	**69**
1,00E-05	**36**
3,00E-05	**91**
6,00E-05	**62**

E2 reference

cocentration [M]	Replicate 1	Replicate 2	Replicate 3	Mean
3,00E-08	3802	3434	3573	**3603**

E2 reference-solvent control

Mean
3461

Normalization (substance/30 nM E2)

cocentration [M]	**Mean**
1,00E-08	**-0,20%**
1,00E-07	**0,42%**
1,00E-06	**0,40%**
3,00E-06	**1,04%**
6,00E-06	**1,98%**
1,00E-05	**1,04%**
3,00E-05	**2,62%**
6,00E-05	**1,79%**

2nd experiment

luminescence

cocentration [M]	Replicate 1	Replicate 2	Replicate 3	Mean
1,00E-08	145	153	154	**151**
1,00E-07	125	112	137	**125**
1,00E-06	105	134	137	**125**
3,00E-06	138	132	160	**143**
6,00E-06	150	137	123	**137**
1,00E-05	152	187	137	**159**
3,00E-05	149	215	157	**174**
6,00E-05	197	160	177	**178**

solvent control

	Replicate 1	Replicate 2	Replicate 3	Replicate 4	Replicate 5	Replicate 6	Mean
	154	139	123	139	130	169	**142**

substance-solvent control

cocentration [M]	Mean
1,00E-08	**8**
1,00E-07	**-18**
1,00E-06	**-17**
3,00E-06	**1**
6,00E-06	**-6**
1,00E-05	**16**
3,00E-05	**31**
6,00E-05	**36**

E2 reference

cocentration [M]	Replicate 1	Replicate 2	Replicate 3	Mean
3,00E-08	2365	2869	2848	**2694**

E2 reference-solvent control

Mean
2552

Normalization (substance/30 nM E2)

cocentration [M]	**Mean**
1,00E-08	**0,33%**
1,00E-07	**-0,69%**
1,00E-06	**-0,67%**
3,00E-06	**0,04%**
6,00E-06	**-0,22%**
1,00E-05	**0,64%**
3,00E-05	**1,23%**
6,00E-05	**1,40%**

3rd experiment

luminescence

cocentration [M]	Replicate 1	Replicate 2	Replicate 3	Mean
1,00E-08	206	189	228	**208**
1,00E-07	184	180	215	**193**
1,00E-06	216	212	188	**205**
3,00E-06	219	186	196	**200**
6,00E-06	202	199	192	**198**
1,00E-05	162	185	228	**192**
3,00E-05	210	203	229	**214**
6,00E-05	227	203	226	**219**

solvent control

	Replicate 1	Replicate 2	Replicate 3	Replicate 4	Replicate 5	Replicate 6	Mean
	245	215	208	225	188	246	**221**

substance-solvent control

cocentration [M]	Mean
1,00E-08	**-14**
1,00E-07	**-28**
1,00E-06	**-16**
3,00E-06	**-21**
6,00E-06	**-24**
1,00E-05	**-30**
3,00E-05	**-7**
6,00E-05	**-3**

E2 reference

cocentration [M]	Replicate 1	Replicate 2	Replicate 3	Mean
3,00E-08	1469	1383	1391	**1414**

E2 reference-solvent control

Mean
1193

Normalization (substance/30 nM E2)

cocentration [M]	**Mean**
1,00E-08	**-1,13%**
1,00E-07	**-2,36%**
1,00E-06	**-1,33%**
3,00E-06	**-1,75%**
6,00E-06	**-1,97%**
1,00E-05	**-2,47%**
3,00E-05	**-0,60%**
6,00E-05	**-0,21%**

ERβ CALUX fenarimol

1st experient

luminescence

cocentration [M]	Replicate 1	Replicate 2	Replicate 3	Mean
1,00E-08	139	131	96	**122**
1,00E-07	147	129	108	**128**
1,00E-06	135	188	102	**142**
3,00E-06	221	200	190	**204**
6,00E-06	294	369	248	**304**
1,00E-05	478	499	473	**483**
3,00E-05	920	878	839	**879**
6,00E-05	737	670	870	**759**

solvent control

	Replicate 1	Replicate 2	Replicate 3	Replicate 4	Replicate 5	Replicate 6	Mean
	149	102	111	145	143	170	**137**

substance-solvent control

cocentration [M]	Mean
1,00E-08	**-15**
1,00E-07	**-9**
1,00E-06	**5**
3,00E-06	**67**
6,00E-06	**167**
1,00E-05	**347**
3,00E-05	**742**
6,00E-05	**622**

E2 reference

cocentration [M]	Replicate 1	Replicate 2	Replicate 3	Mean
3,00E-08	3247	3298	3251	**3265**

E2 reference-solvent control

Mean
3129

Normalization (substance/30 nM E2)

cocentration [M]	**Mean**
1,00E-08	**-0,47%**
1,00E-07	**-0,28%**
1,00E-06	**0,16%**
3,00E-06	**2,14%**
6,00E-06	**5,34%**
1,00E-05	**11,08%**
3,00E-05	**23,73%**
6,00E-05	**19,89%**

2nd experient

luminescence

cocentration [M]	Replicate 1	Replicate 2	Replicate 3	Mean
1,00E-08	141	147	144	**144**
1,00E-07	162	141	147	**150**
1,00E-06	126	129	150	**135**
3,00E-06	167	156	168	**164**
6,00E-06	192	225	217	**211**
1,00E-05	297	303	284	**295**
3,00E-05	646	599	564	**603**
6,00E-05	505	527	615	**549**

solvent control

	Replicate 1	Replicate 2	Replicate 3	Replicate 4	Replicate 5	Replicate 6	Mean
	160	120	144	147	152	149	**145**

substance-solvent control

cocentration [M]	Mean
1,00E-08	**-1**
1,00E-07	**5**
1,00E-06	**-10**
3,00E-06	**18**
6,00E-06	**66**
1,00E-05	**149**
3,00E-05	**458**
6,00E-05	**404**

E2 reference

cocentration [M]	Replicate 1	Replicate 2	Replicate 3	Mean
3,00E-08	2623	2445	2497	**2522**

E2 reference-solvent control

Mean
2376

Normalization (substance/30 nM E2)

cocentration [M]	**Mean**
1,00E-08	**-0,06%**
1,00E-07	**0,20%**
1,00E-06	**-0,43%**
3,00E-06	**0,77%**
6,00E-06	**2,78%**
1,00E-05	**6,28%**
3,00E-05	**19,26%**
6,00E-05	**16,99%**

3rd experiment

luminescence

cocentration [M	Replicate 1	Replicate 2	Replicate 3	Mean
1,00E-08	231	193	200	**208**
1,00E-07	182	225	220	**209**
1,00E-06	215	235	196	**215**
3,00E-06	204	199	252	**218**
6,00E-06	226	225	244	**232**
1,00E-05	258	269	219	**249**
3,00E-05	356	353	358	**356**
6,00E-05	509	452	431	**464**

solvent control

	Replicate 1	Replicate 2	Replicate 3	Replicate 4	Replicate 5	Replicate 6	Mean
	195	223	229	237	231	244	**227**

substance-solvent control

cocentration [M	Mean
1,00E-08	**-19**
1,00E-07	**-18**
1,00E-06	**-11**
3,00E-06	**-8**
6,00E-06	**5**
1,00E-05	**22**
3,00E-05	**129**
6,00E-05	**238**

E2 reference

cocentration [M	Replicate 1	Replicate 2	Replicate 3	Mean
3,00E-08	1205	1386	1250	**1280**

E2 reference-solvent control

Mean
1054

Normalization (substance/30 nM E2)

cocentration [M	**Mean**
1,00E-08	**-1,76%**
1,00E-07	**-1,66%**
1,00E-06	**-1,06%**
3,00E-06	**-0,77%**
6,00E-06	**0,49%**
1,00E-05	**2,10%**
3,00E-05	**12,26%**
6,00E-05	**22,54%**

ERβ CALUX fenhexamid

1st experient

luminescence

cocentration [M]	Replicate 1	Replicate 2	Replicate 3	Mean
1,00E-08	163	123	143	**143**
1,00E-07	147	125	148	**140**
1,00E-06	171	177	149	**166**
3,00E-06	295	311	294	**300**
6,00E-06	606	602	597	**602**
1,00E-05	1134	903	961	**999**
3,00E-05	1611	1512	1558	**1560**
6,00E-05	1619	1895	1620	**1711**

solvent control

	Replicate 1	Replicate 2	Replicate 3	Replicate 4	Replicate 5	Replicate 6	Mean
	173	122	139	165	140	1042	**297**

substance-solvent control

cocentration [M]	Mean
1,00E-08	**-5**
1,00E-07	**-8**
1,00E-06	**17**
3,00E-06	**152**
6,00E-06	**453**
1,00E-05	**851**
3,00E-05	**1412**
6,00E-05	**1563**

E2 reference

cocentration [M]	Replicate 1	Replicate 2	Replicate 3	Mean
3,00E-08	4126	3748	3791	**3888**

E2 reference-solvent control

Mean
3592

Normalization (substance/30 nM E2)

cocentration [M]	**Mean**
1,00E-08	**-0,15%**
1,00E-07	**-0,23%**
1,00E-06	**0,48%**
3,00E-06	**4,22%**
6,00E-06	**12,62%**
1,00E-05	**23,69%**
3,00E-05	**39,32%**
6,00E-05	**43,52%**

2nd experient

luminescence

cocentration [M]	Replicate 1	Replicate 2	Replicate 3	Mean
1,00E-08	159	145	136	**147**
1,00E-07	155	135	158	**149**
1,00E-06	168	169	163	**167**
3,00E-06	306	260	235	**267**
6,00E-06	511	596	445	**517**
1,00E-05	1025	840	876	**914**
3,00E-05	1421	1081	1368	**1290**
6,00E-05	1385	1411	1452	**1416**

solvent control

	Replicate 1	Replicate 2	Replicate 3	Replicate 4	Replicate 5	Replicate 6	Mean
	144	150	169	137	138	149	**148**

substance-solvent control

cocentration [M]	Mean
1,00E-08	**-1**
1,00E-07	**2**
1,00E-06	**19**
3,00E-06	**119**
6,00E-06	**370**
1,00E-05	**766**
3,00E-05	**1142**
6,00E-05	**1268**

E2 reference

cocentration [M]	Replicate 1	Replicate 2	Replicate 3	Mean
3,00E-08	2382	2284	2726	**2464**

E2 reference-solvent control

Mean
2316

Normalization (substance/30 nM E2)

cocentration [M]	**Mean**
1,00E-08	**-0,05%**
1,00E-07	**0,06%**
1,00E-06	**0,81%**
3,00E-06	**5,14%**
6,00E-06	**15,95%**
1,00E-05	**33,06%**
3,00E-05	**49,31%**
6,00E-05	**54,75%**

3rd experiment

luminescence

cocentration [M	Replicate 1	Replicate 2	Replicate 3	Mean
1,00E-08	217	211	177	**202**
1,00E-07	160	191	177	**176**
1,00E-06	217	195	199	**204**
3,00E-06	328	308	292	**309**
6,00E-06	548	343	408	**433**
1,00E-05	785	589	653	**676**
3,00E-05	1039	890	992	**974**
6,00E-05	956	1058	1170	**1061**

solvent control

	Replicate 1	Replicate 2	Replicate 3	Replicate 4	Replicate 5	Replicate 6	Mean
	228	191	224	192	217	212	**211**

substance-solvent control

cocentration [M	Mean
1,00E-08	**-9**
1,00E-07	**-35**
1,00E-06	**-7**
3,00E-06	**99**
6,00E-06	**222**
1,00E-05	**465**
3,00E-05	**763**
6,00E-05	**851**

E2 reference

cocentration [M	Replicate 1	Replicate 2	Replicate 3	Mean
3,00E-08	2070	2316	2509	**2298**

E2 reference-solvent control

Mean
2088

Normalization (substance/30 nM E2)

cocentration [M	**Mean**
1,00E-08	**-0,43%**
1,00E-07	**-1,66%**
1,00E-06	**-0,34%**
3,00E-06	**4,73%**
6,00E-06	**10,65%**
1,00E-05	**22,27%**
3,00E-05	**36,55%**
6,00E-05	**40,75%**

ERβ CALUX fludioxonil

1st experient

luminescence

cocentration [M]	Replicate 1	Replicate 2	Replicate 3	Mean
1,00E-08	142	123	134	**133**
1,00E-07	157	103	115	**125**
1,00E-06	279	306	258	**281**
3,00E-06	716	574	661	**650**
6,00E-06	1090	833	1134	**1019**
1,00E-05	991	1052	1146	**1063**
3,00E-05	1272	1063	1223	**1186**
6,00E-05	1103	1097	1448	**1216**

solvent control

	Replicate 1	Replicate 2	Replicate 3	Replicate 4	Replicate 5	Replicate 6	Mean
	149	102	111	145	143	170	**137**

substance-solvent control

cocentration [M]	Mean
1,00E-08	**-4**
1,00E-07	**-12**
1,00E-06	**144**
3,00E-06	**514**
6,00E-06	**882**
1,00E-05	**926**
3,00E-05	**1049**
6,00E-05	**1079**

E2 reference

cocentration [M]	Replicate 1	Replicate 2	Replicate 3	Mean
3,00E-08	3247	3298	3251	**3265**

E2 reference-solvent control

Mean
3129

Normalization (substance/30 nM E2)

cocentration [M]	Mean
1,00E-08	**-0,12%**
1,00E-07	**-0,37%**
1,00E-06	**4,61%**
3,00E-06	**16,42%**
6,00E-06	**28,20%**
1,00E-05	**29,61%**
3,00E-05	**33,54%**
6,00E-05	**34,50%**

2nd experient

luminescence

cocentration [M]	Replicate 1	Replicate 2	Replicate 3	Mean
1,00E-08	160	145	168	**158**
1,00E-07	153	134	132	**140**
1,00E-06	150	122	137	**136**
3,00E-06	223	168	177	**189**
6,00E-06	176	172	170	**173**
1,00E-05	513	559	642	**571**
3,00E-05	1135	937	909	**994**
6,00E-05	1222	1006	1253	**1160**

solvent control

	Replicate 1	Replicate 2	Replicate 3	Replicate 4	Replicate 5	Replicate 6	Mean
	160	120	144	147	152	149	**145**

substance-solvent control

cocentration [M]	Mean
1,00E-08	**12**
1,00E-07	**-6**
1,00E-06	**-9**
3,00E-06	**44**
6,00E-06	**27**
1,00E-05	**426**
3,00E-05	**848**
6,00E-05	**1015**

E2 reference

cocentration [M]	Replicate 1	Replicate 2	Replicate 3	Mean
3,00E-08	2623	2445	2497	**2522**

E2 reference-solvent control

Mean
2376

Normalization (substance/30 nM E2)

cocentration [M]	Mean
1,00E-08	**0,52%**
1,00E-07	**-0,24%**
1,00E-06	**-0,38%**
3,00E-06	**1,85%**
6,00E-06	**1,15%**
1,00E-05	**17,93%**
3,00E-05	**35,70%**
6,00E-05	**42,71%**

3rd experiment

luminescence

cocentration [M	Replicate 1	Replicate 2	Replicate 3	Mean
1,00E-08	187	192	221	**200**
1,00E-07	191	224	214	**210**
1,00E-06	231	205	232	**223**
3,00E-06	238	222	234	**231**
6,00E-06	192	240	290	**241**
1,00E-05	300	436	464	**400**
3,00E-05	422	449	662	**511**
6,00E-05	694	736	813	**748**

solvent control

	Replicate 1	Replicate 2	Replicate 3	Replicate 4	Replicate 5	Replicate 6	Mean
	195	223	229	237	231	244	**227**

substance-solvent control

cocentration [M	Mean
1,00E-08	**-27**
1,00E-07	**-17**
1,00E-06	**-4**
3,00E-06	**5**
6,00E-06	**14**
1,00E-05	**174**
3,00E-05	**285**
6,00E-05	**521**

E2 reference

cocentration [M	Replicate 1	Replicate 2	Replicate 3	Mean
3,00E-08	1205	1386	1250	**1280**

E2 reference-solvent control

Mean
1054

Normalization (substance/30 nM E2)

cocentration [M	Mean
1,00E-08	**-2,51%**
1,00E-07	**-1,60%**
1,00E-06	**-0,36%**
3,00E-06	**0,46%**
6,00E-06	**1,34%**
1,00E-05	**16,46%**
3,00E-05	**27,00%**
6,00E-05	**49,45%**

ERβ CALUX 4,4'-DDT

1st experient

luminescence

cocentration [M]	Replicate 1	Replicate 2	Replicate 3	Mean
1,00E-08	118	121	137	**125**
1,00E-07	115	160	182	**152**
1,00E-06	176	187	225	**196**
3,00E-06	337	265	282	**295**
6,00E-06	414	426	458	**433**
1,00E-05	441	404	524	**456**
3,00E-05	359	342	437	**379**
6,00E-05	350	389	280	**340**

solvent control

	Replicate 1	Replicate 2	Replicate 3	Replicate 4	Replicate 5	Replicate 6	Mean
	173	122	139	165	140	1042	**297**

substance-solvent control

cocentration [M]	Mean
1,00E-08	**-23**
1,00E-07	**4**
1,00E-06	**48**
3,00E-06	**146**
6,00E-06	**284**
1,00E-05	**308**
3,00E-05	**231**
6,00E-05	**191**

E2 reference

cocentration [M]	Replicate 1	Replicate 2	Replicate 3	Mean
3,00E-08	4126	3748	3791	**3888**

E2 reference-solvent control

Mean
3592

Normalization (substance/30 nM E2)

cocentration [M]	**Mean**
1,00E-08	**-0,64%**
1,00E-07	**0,11%**
1,00E-06	**1,33%**
3,00E-06	**4,07%**
6,00E-06	**7,92%**
1,00E-05	**8,58%**
3,00E-05	**6,43%**
6,00E-05	**5,33%**

2nd experient

luminescence

cocentration [M]	Replicate 1	Replicate 2	Replicate 3	Mean
1,00E-08	126	124	143	**131**
1,00E-07	145	125	135	**135**
1,00E-06	176	165	168	**170**
3,00E-06	194	241	207	**214**
6,00E-06	278	251	321	**283**
1,00E-05	321	315	295	**310**
3,00E-05	360	358	297	**338**
6,00E-05	260	284	296	**280**

solvent control

	Replicate 1	Replicate 2	Replicate 3	Replicate 4	Replicate 5	Replicate 6	Mean
	144	150	169	137	138	149	**148**

substance-solvent control

cocentration [M]	Mean
1,00E-08	**-17**
1,00E-07	**-13**
1,00E-06	**22**
3,00E-06	**66**
6,00E-06	**136**
1,00E-05	**163**
3,00E-05	**191**
6,00E-05	**132**

E2 reference

cocentration [M]	Replicate 1	Replicate 2	Replicate 3	Mean
3,00E-08	2382	2284	2726	**2464**

E2 reference-solvent control

Mean
2316

Normalization (substance/30 nM E2)

cocentration [M]	**Mean**
1,00E-08	**-0,73%**
1,00E-07	**-0,55%**
1,00E-06	**0,94%**
3,00E-06	**2,86%**
6,00E-06	**5,85%**
1,00E-05	**7,02%**
3,00E-05	**8,22%**
6,00E-05	**5,71%**

3rd experiment

luminescence

cocentration [M	Replicate 1	Replicate 2	Replicate 3	Mean
1,00E-08	215	174	197	**195**
1,00E-07	165	162	203	**177**
1,00E-06	206	166	179	**184**
3,00E-06	192	220	193	**202**
6,00E-06	229	277	363	**290**
1,00E-05	308	313	352	**324**
3,00E-05	317	323	351	**330**
6,00E-05	424	347	396	**389**

solvent control

	Replicate 1	Replicate 2	Replicate 3	Replicate 4	Replicate 5	Replicate 6	Mean
	228	191	224	192	217	212	**211**

substance-solvent control

cocentration [M	Mean
1,00E-08	**-15**
1,00E-07	**-34**
1,00E-06	**-27**
3,00E-06	**-9**
6,00E-06	**79**
1,00E-05	**114**
3,00E-05	**120**
6,00E-05	**178**

E2 reference

cocentration [M	Replicate 1	Replicate 2	Replicate 3	Mean
3,00E-08	2070	2316	2509	**2298**

E2 reference-solvent control

Mean
2088

Normalization (substance/30 nM E2)

cocentration [M	**Mean**
1,00E-08	**-0,73%**
1,00E-07	**-1,63%**
1,00E-06	**-1,29%**
3,00E-06	**-0,43%**
6,00E-06	**3,78%**
1,00E-05	**5,44%**
3,00E-05	**5,73%**
6,00E-05	**8,54%**

ERβ CALUX 2,4'-DDT

1st experient

luminescence

cocentration [M]	Replicate 1	Replicate 2	Replicate 3	Mean
1,00E-08	152	137	144	**144**
1,00E-07	205	137	179	**174**
6,00E-07	459	409	495	**454**
1,00E-06	625	555	595	**592**
3,00E-06	917	875	946	**913**
6,00E-06	1127	1086	1020	**1078**
1,00E-05	1207	892	936	**1012**
3,00E-05	1093	962	919	**991**

solvent control

	Replicate 1	Replicate 2	Replicate 3	Mean
	158	148	154	**153**

substance-solvent control

cocentration [M]	Mean
1,00E-08	**-9**
1,00E-07	**20**
6,00E-07	**301**
1,00E-06	**438**
3,00E-06	**759**
6,00E-06	**924**
1,00E-05	**858**
3,00E-05	**838**

E2 reference

cocentration [M]	Replicate 1	Replicate 2	Replicate 3	Mean
3,00E-08	2929	2319	2561	**2603**

E2 reference-solvent control

Mean
2450

Normalization (substance/30 nM E2)

cocentration [M]	**Mean**
1,00E-08	**-0,37%**
1,00E-07	**0,83%**
6,00E-07	**12,29%**
1,00E-06	**17,89%**
3,00E-06	**31,00%**
6,00E-06	**37,73%**
1,00E-05	**35,04%**
3,00E-05	**34,21%**

2nd experient

luminescence

cocentration [M]	Replicate 1	Replicate 2	Replicate 3	Mean
1,00E-08	186	217	210	**204**
1,00E-07	197	198	182	**192**
6,00E-07	452	510	371	**444**
1,00E-06	476	519	501	**499**
3,00E-06	665	813	647	**708**
6,00E-06	737	647	636	**673**
1,00E-05	641	565	825	**677**
3,00E-05	884	1016	938	**946**

solvent control

	Replicate 1	Replicate 2	Replicate 3	Mean
	222	213	202	**212**

substance-solvent control

cocentration [M]	Mean
1,00E-08	**-8**
1,00E-07	**-20**
6,00E-07	**232**
1,00E-06	**286**
3,00E-06	**496**
6,00E-06	**461**
1,00E-05	**465**
3,00E-05	**734**

E2 reference

cocentration [M]	Replicate 1	Replicate 2	Replicate 3	Mean
3,00E-08	2081	2006	2450	**2179**

E2 reference-solvent control

Mean
1967

Normalization (substance/30 nM E2)

cocentration [M]	**Mean**
1,00E-08	**-0,41%**
1,00E-07	**-1,02%**
6,00E-07	**11,80%**
1,00E-06	**14,56%**
3,00E-06	**25,22%**
6,00E-06	**23,44%**
1,00E-05	**23,63%**
3,00E-05	**37,31%**

3rd experient

luminescence

cocentration [M	Replicate 1	Replicate 2	Replicate 3	Mean
1,00E-08	125	128	111	**121**
1,00E-07	129	139	130	**133**
6,00E-07	212	258	219	**230**
1,00E-06	275	277	272	**275**
3,00E-06	254	332	439	**342**
6,00E-06	503	445	471	**473**
1,00E-05	433	539	450	**474**
3,00E-05	624	717	647	**663**

solvent control

	Replicate 1	Replicate 2	Replicate 3	Replicate 4	Replicate 5	Replicate 6	Mean
	115	133	145	113	121	139	**128**

substance-solvent control

cocentration [M	Mean
1,00E-08	**-6**
1,00E-07	**5**
6,00E-07	**102**
1,00E-06	**147**
3,00E-06	**214**
6,00E-06	**345**
1,00E-05	**346**
3,00E-05	**535**

E2 reference

cocentration [M	Replicate 1	Replicate 2	Replicate 3	Mean
3,00E-08	1376	1483	1649	**1503**

E2 reference-solvent control

Mean
1375

Normalization (substance/30 nM E2)

cocentration [M	**Mean**
1,00E-08	**-0,46%**
1,00E-07	**0,36%**
6,00E-07	**7,42%**
1,00E-06	**10,69%**
3,00E-06	**15,56%**
6,00E-06	**25,12%**
1,00E-05	**25,19%**
3,00E-05	**38,91%**

YES fenhexamid fludioxonil EC01

1st experient

540nm

cocentration [M]	Replicate 1	Replicate 2	Replicate 3	Replicate 4
1,00E-10	0,693400025	0,703499973	0,722800016	0,65990001
1,00E-09	0,639699996	0,687600017	0,718900025	0,549000025
1,00E-08	0,692700028	0,702099979	0,713500023	0,641200006
1,00E-07	0,676100016	0,699100018	0,702199996	0,627300024
1,00E-06	0,635399997	0,661899984	0,679499984	0,632199983
1,00E-05	0,737699986	0,683499992	0,656300008	0,671199977
1,00E-04	0,731100023	0,707400024	0,688799977	0,749499977

690nm

cocentration [M]	Replicate 1	Replicate 2	Replicate 3	Replicate 4
1,00E-10	0,59920001	0,607200027	0,62379998	0,568899989
1,00E-09	0,544499993	0,591099977	0,621999979	0,466199994
1,00E-08	0,5977	0,603900015	0,616299987	0,551199973
1,00E-07	0,580500007	0,599799991	0,604600012	0,537199974
1,00E-06	0,543500006	0,566900015	0,583199978	0,538600028
1,00E-05	0,5995	0,548600018	0,524500012	0,539499998
1,00E-04	0,587899983	0,558499992	0,541199982	0,602699995

solvent control 690nm

	Replicate 1	Replicate 2	Replicate 3	Replicate 4
	0,682200015	0,687900007	0,636600018	0,56400001

540-690nm

cocentration [M]	Replicate 1	Replicate 2	Replicate 3	Replicate 4
1,00E-10	0,0942	0,096299998	0,098999999	0,090999998
1,00E-09	0,095200002	0,0964	0,096900001	0,082699999
1,00E-08	0,094899997	0,098200001	0,097199999	0,089900002
1,00E-07	0,095600002	0,099299997	0,097499996	0,090099998
1,00E-06	0,091899998	0,095100001	0,096299998	0,0942
1,00E-05	0,1382	0,134900004	0,131799996	0,131699994
1,00E-04	0,143199995	0,148800001	0,147699997	0,146799996

solvent control 540nm-690nm

	Replicate 1	Replicate 2	Replicate 3	Replicate 4
	0,102700002	0,101300001	0,099600002	0,087499999

(540nm-690nm)-solvent control (540nm-690nm)

cocentration [M]	Replicate 1	Replicate 2	Replicate 3	Replicate 4
1,00E-10	-0,003575001	-0,001475003	0,001224998	-0,006775003
1,00E-09	-0,002574999	-0,001375001	-0,000875	-0,015075002
1,00E-08	-0,002875004	0,000425	-0,000575002	-0,007874999
1,00E-07	-0,002175	0,001524996	-0,000275005	-0,007675003
1,00E-06	-0,005875003	-0,002675001	-0,001475003	-0,003575001
1,00E-05	0,040424999	0,037125003	0,034024995	0,033924993
1,00E-04	0,045424994	0,051024999	0,049924996	0,049024995

(540nm-690nm)-solvent control E2 reference

cocentration [M]	Replicate 1	Replicate 2	Replicate 3	Replicate 4
1,00E-09	0,367625009	0,331525009	0,298325013	0,294624995

Normalization (substance/1 nM E2)

cocentration [M]	Replicate 1	Replicate 2	Replicate 3	Replicate 4	Mean
1,00E-10	-0,97%	-0,44%	0,41%	-2,30%	**-0,83%**
1,00E-09	-0,70%	-0,41%	-0,29%	-5,12%	**-1,63%**
1,00E-08	-0,78%	0,13%	-0,19%	-2,67%	**-0,88%**
1,00E-07	-0,59%	0,46%	-0,09%	-2,61%	**-0,71%**
1,00E-06	-1,60%	-0,81%	-0,49%	-1,21%	**-1,03%**
1,00E-05	11,00%	11,20%	11,41%	11,51%	**11,28%**
1,00E-04	12,36%	15,39%	16,74%	16,64%	**15,28%**

yeast-growth/solubility 690nm/solvent control 690nm

cocentration [M]	Replicate 1	Replicate 2	Replicate 3	Replicate 4	Mean
1,00E-10	87,83%	88,27%	97,99%	100,87%	**93,74%**
1,00E-09	79,82%	85,93%	97,71%	82,66%	**86,53%**
1,00E-08	87,61%	87,79%	96,81%	97,73%	**92,49%**
1,00E-07	85,09%	87,19%	94,97%	95,25%	**90,63%**
1,00E-06	87,88%	79,75%	82,39%	95,66%	**86,42%**
1,00E-05	79,67%	82,41%	91,61%	95,50%	**87,30%**
1,00E-04	86,18%	81,19%	85,01%	106,86%	**89,81%**

2nd experient

540nm

cocentration [M]	Replicate 1	Replicate 2	Replicate 3	Replicate 4
1,00E-10	0,565500021	0,630200028	0,551800013	0,648599982
1,00E-09	0,78579998	0,686800003	0,691100001	0,707199991
1,00E-08	0,891200006	0,787100017	0,754100025	0,797599971
1,00E-07	0,856899977	0,797900021	0,637600005	0,782299995
1,00E-06	0,825999975	0,632799983	0,66960001	0,69569999
1,00E-05	0,758000016	0,687900007	0,754599988	0,771499991
1,00E-04	0,782700002	0,891900003	0,789399981	0,791700006

690nm

cocentration [M]	Replicate 1	Replicate 2	Replicate 3	Replicate 4
1,00E-10	0,4824	0,538699985	0,465499997	0,558399975
1,00E-09	0,680100024	0,586600006	0,590499997	0,605400026
1,00E-08	0,778100014	0,67930001	0,649399996	0,691600025
1,00E-07	0,746500015	0,689499974	0,542800009	0,675999999
1,00E-06	0,71509999	0,535099983	0,569500029	0,596199989
1,00E-05	0,601499975	0,537	0,595799983	0,614300013
1,00E-04	0,607500017	0,707899988	0,611400008	0,613900006

solvent control 690nm

	Replicate 1	Replicate 2	Replicate 3	Replicate 4
	0,628499985	0,417100012	0,548099995	0,487800002

540-690nm

cocentration [M]	Replicate 1	Replicate 2	Replicate 3	Replicate 4
1,00E-10	0,083099999	0,091499999	0,086199999	0,0902
1,00E-09	0,105700001	0,100199997	0,100599997	0,101800002
1,00E-08	0,1131	0,107799999	0,104699999	0,105999999
1,00E-07	0,110399999	0,108400002	0,094800003	0,106299996
1,00E-06	0,1109	0,0977	0,100100003	0,0995
1,00E-05	0,156499997	0,150900006	0,158800006	0,157299995
1,00E-04	0,1752	0,184100002	0,177900001	0,1778

solvent control 540nm-690nm

	Replicate 1	Replicate 2	Replicate 3	Replicate 4
	0,104699999	0,079999998	0,091700003	0,089599997

(540nm-690nm)-solvent control (540nm-690nm)

cocentration [M]	Replicate 1	Replicate 2	Replicate 3	Replicate 4
1,00E-10	-0,008400001	0	-0,0053	-0,0013
1,00E-09	0,014200002	0,008699998	0,009099998	0,010300003
1,00E-08	0,021600001	0,0163	0,0132	0,0145
1,00E-07	0,0189	0,016900003	0,003300004	0,014799997
1,00E-06	0,019400001	0,006200001	0,008600004	0,008000001
1,00E-05	0,064999998	0,059400007	0,067300007	0,065799996
1,00E-04	0,083700001	0,092600003	0,086400002	0,086300001

(540nm-690nm)-solvent control E2 reference

cocentration [M]	Replicate 1	Replicate 2	Replicate 3	Replicate 4
1,00E-09	0,44219999	0,417399988	0,404699996	0,463799998

Normalization (substance/1 nM E2)

cocentration [M]	Replicate 1	Replicate 2	Replicate 3	Replicate 4	Mean
1,00E-10	-1,90%	0,00%	-1,31%	-0,28%	**-0,87%**
1,00E-09	3,21%	2,08%	2,25%	2,22%	**2,44%**
1,00E-08	4,88%	3,91%	3,26%	3,13%	**3,79%**
1,00E-07	4,27%	4,05%	0,82%	3,19%	**3,08%**
1,00E-06	4,39%	1,49%	2,13%	1,72%	**2,43%**
1,00E-05	14,70%	14,23%	16,63%	14,19%	**14,94%**
1,00E-04	18,93%	22,18%	21,35%	18,61%	**20,27%**

yeast-growth/solubility 690nm/solvent control 690nm

cocentration [M]	Replicate 1	Replicate 2	Replicate 3	Replicate 4	Mean
1,00E-10	76,75%	129,15%	84,93%	114,47%	**101,33%**
1,00E-09	108,21%	140,64%	107,74%	124,11%	**120,17%**
1,00E-08	123,80%	162,86%	118,48%	141,78%	**136,73%**
1,00E-07	118,77%	165,31%	99,03%	138,58%	**130,42%**
1,00E-06	113,78%	128,29%	103,90%	122,22%	**117,05%**
1,00E-05	95,70%	128,75%	108,70%	125,93%	**114,77%**
1,00E-04	96,66%	169,72%	111,55%	125,85%	**125,94%**

3rd experient

540nm

cocentration [M	Replicate 1	Replicate 2	Replicate 3	Replicate 4
1,00E-10	0,622600019	0,603600025	0,517099977	0,558700025
1,00E-09	0,626100004	0,664600015	0,754800022	0,811500013
1,00E-08	0,686500013	0,722000003	0,7597	0,718999982
1,00E-07	0,735599995	0,771499991	0,77759999	0,784200013
1,00E-06	0,739099979	0,79519999	0,747699976	0,795599997
1,00E-05	0,697399974	0,6796	0,656599998	0,629299998
1,00E-04	0,836899996	0,82980001	0,808399975	0,773999989

690nm

cocentration [M	Replicate 1	Replicate 2	Replicate 3	Replicate 4
1,00E-10	0,532199979	0,514699996	0,442900002	0,479499996
1,00E-09	0,539499998	0,573899984	0,658800006	0,708000004
1,00E-08	0,592199981	0,621100008	0,657599986	0,626800001
1,00E-07	0,634899974	0,665799975	0,672200024	0,678600013
1,00E-06	0,637199998	0,686999977	0,648000002	0,692700028
1,00E-05	0,552900016	0,534699976	0,511300027	0,484899998
1,00E-04	0,658200026	0,656099975	0,630800009	0,601899981

solvent control 690nm

	Replicate 1	Replicate 2	Replicate 3	Replicate 4
	0,610000014	0,414299995	0,659300029	0,559700012

540-690nm

cocentration [M	Replicate 1	Replicate 2	Replicate 3	Replicate 4
1,00E-10	0,090400003	0,0889	0,074199997	0,0792
1,00E-09	0,086599998	0,090700001	0,096000001	0,103500001
1,00E-08	0,094300002	0,100900002	0,1021	0,092100002
1,00E-07	0,100699998	0,105700001	0,105400003	0,105599999
1,00E-06	0,101800002	0,108199999	0,099699996	0,102899998
1,00E-05	0,144500002	0,144800007	0,145400003	0,144400001
1,00E-04	0,1787	0,173800007	0,177599996	0,172099993

solvent control 540nm-690nm

	Replicate 1	Replicate 2	Replicate 3	Replicate 4
	0,098800004	0,0757	0,103	0,093900003

(540nm-690nm)-solvent control (540nm-690nm)

cocentration [M	Replicate 1	Replicate 2	Replicate 3	Replicate 4
1,00E-10	-0,002449999	-0,003950002	-0,018650005	-0,013650002
1,00E-09	-0,006250003	-0,002150001	0,003149999	0,01065
1,00E-08	0,00145	0,00805	0,009249998	-0,00075
1,00E-07	0,007849997	0,01285	0,012550002	0,012749998
1,00E-06	0,008950001	0,015349997	0,006849995	0,010049997
1,00E-05	0,051650001	0,051950006	0,052550001	0,051549999
1,00E-04	0,085849999	0,080950005	0,084749995	0,079249991

(540nm-690nm)-solvent control E2 reference

cocentration [M	Replicate 1	Replicate 2	Replicate 3	Replicate 4
1,00E-09	0,456450013	0,511050014	0,461550025	0,480150012

Normalization (substance/1 nM E2)

cocentration [M	Replicate 1	Replicate 2	Replicate 3	Replicate 4	Mean
1,00E-10	-0,54%	-0,77%	-4,04%	-2,84%	**-2,05%**
1,00E-09	-1,37%	-0,42%	0,68%	2,22%	**0,28%**
1,00E-08	0,32%	1,58%	2,00%	-0,16%	**0,94%**
1,00E-07	1,72%	2,51%	2,72%	2,66%	**2,40%**
1,00E-06	1,96%	3,00%	1,48%	2,09%	**2,14%**
1,00E-05	11,32%	10,17%	11,39%	10,74%	**10,90%**
1,00E-04	18,81%	15,84%	18,36%	16,51%	**17,38%**

yeast-growth/solubility 690nm/solvent control 690nm

cocentration [M	Replicate 1	Replicate 2	Replicate 3	Replicate 4	Mean
1,00E-10	87,25%	124,23%	67,18%	85,67%	**91,08%**
1,00E-09	88,44%	138,52%	99,92%	126,50%	**113,35%**
1,00E-08	97,08%	149,92%	99,74%	111,99%	**114,68%**
1,00E-07	104,08%	160,70%	101,96%	121,24%	**122,00%**
1,00E-06	104,46%	165,82%	98,29%	123,76%	**123,08%**
1,00E-05	90,64%	129,06%	77,55%	86,64%	**95,97%**
1,00E-04	107,90%	158,36%	95,68%	107,54%	**117,37%**

4th experiment

540nm

cocentration [M	Replicate 1	Replicate 2	Replicate 3	Replicate 4
1,00E-10	0,735599995	0,752300024	0,690299988	0,701699972
1,00E-09	0,761500001	0,717999995	0,706900001	0,666599989
1,00E-08	0,769400001	0,916000009	0,69630003	0,676400006
1,00E-07	0,716000021	0,742600024	0,747699976	0,699800014
1,00E-06	0,646000028	0,75120002	0,665899992	0,710500002
1,00E-05	0,675899982	0,668900013	0,650600016	0,646000028
1,00E-04	0,666800022	0,672999978	0,716400027	0,741699994

690nm

cocentration [M	Replicate 1	Replicate 2	Replicate 3	Replicate 4
1,00E-10	0,631799996	0,643599987	0,587700009	0,5995
1,00E-09	0,653699994	0,612800002	0,604499996	0,564899981
1,00E-08	0,661899984	0,807600021	0,592999995	0,57249999
1,00E-07	0,610499978	0,636200011	0,642099977	0,597199976
1,00E-06	0,546700001	0,643400013	0,56309998	0,605499983
1,00E-05	0,542200029	0,539300025	0,526799977	0,518599987
1,00E-04	0,527199984	0,529200017	0,573000014	0,5995

solvent control 690nm

	Replicate 1	Replicate 2	Replicate 3	Replicate 4
	0,590499997	0,568000019	0,572300017	0,475899994

540-690nm

cocentration [M	Replicate 1	Replicate 2	Replicate 3	Replicate 4
1,00E-10	0,103699997	0,108599998	0,102600001	0,102200001
1,00E-09	0,107799999	0,1052	0,102399997	0,101800002
1,00E-08	0,107500002	0,108499996	0,103200004	0,1039
1,00E-07	0,105400003	0,106399998	0,105599999	0,102700002
1,00E-06	0,099299997	0,107699998	0,102799997	0,104999997
1,00E-05	0,133699998	0,129600003	0,123800002	0,127499998
1,00E-04	0,139599994	0,143900007	0,143299997	0,142199993

solvent control 540nm-690nm

	Replicate 1	Replicate 2	Replicate 3	Replicate 4
	0,1052	0,101499997	0,102200001	0,089000002

(540nm-690nm)-solvent control (540nm-690nm)

cocentration [M	Replicate 1	Replicate 2	Replicate 3	Replicate 4
1,00E-10	0,004224997	0,009124998	0,003125001	0,002725001
1,00E-09	0,008324999	0,005725	0,002924997	0,002325002
1,00E-08	0,008025002	0,009024996	0,003725003	0,004425
1,00E-07	0,005925003	0,006924998	0,006124999	0,003225002
1,00E-06	-0,000175003	0,008224998	0,003324997	0,005524997
1,00E-05	0,034224998	0,030125003	0,024325002	0,028024998
1,00E-04	0,040124994	0,044425007	0,043824997	0,042724993

(540nm-690nm)-solvent control E2 reference

cocentration [M	Replicate 1	Replicate 2	Replicate 3	Replicate 4
1,00E-09	0,422525015	0,406025019	0,402924981	0,404024985

Normalization (substance/1 nM E2)

cocentration [M	Replicate 1	Replicate 2	Replicate 3	Replicate 4	Mean
1,00E-10	1,00%	2,25%	0,78%	0,67%	**1,17%**
1,00E-09	1,97%	1,41%	0,73%	0,58%	**1,17%**
1,00E-08	1,90%	2,22%	0,92%	1,10%	**1,54%**
1,00E-07	1,40%	1,71%	1,52%	0,80%	**1,36%**
1,00E-06	-0,04%	2,03%	0,83%	1,37%	**1,04%**
1,00E-05	8,10%	7,42%	6,04%	6,94%	**7,12%**
1,00E-04	9,50%	10,94%	10,88%	10,57%	**10,47%**

yeast-growth/solubility 690nm/solvent control 690nm

cocentration [M	Replicate 1	Replicate 2	Replicate 3	Replicate 4	Mean
1,00E-10	106,99%	113,31%	102,69%	125,97%	**112,24%**
1,00E-09	110,70%	107,89%	105,63%	118,70%	**110,73%**
1,00E-08	112,09%	142,18%	103,62%	120,30%	**119,55%**
1,00E-07	103,39%	112,01%	112,20%	125,49%	**113,27%**
1,00E-06	92,58%	113,27%	98,39%	127,23%	**107,87%**
1,00E-05	91,82%	94,95%	92,05%	108,97%	**96,95%**
1,00E-04	89,28%	93,17%	100,12%	125,97%	**102,14%**

5th experiment

540nm

cocentration [M	Replicate 1	Replicate 2	Replicate 3	Replicate 4
1,00E-10	0,692600012	0,757600009	0,726100028	0,735700011
1,00E-09	0,866299987	0,808899999	0,810599983	0,809899986
1,00E-08	0,780300021	0,779200017	0,774600029	0,769800007
1,00E-07	0,783800006	0,760200024	0,708199978	0,697399974
1,00E-06	0,685400009	0,734499991	0,67750001	0,699100018
1,00E-05	0,685699999	0,653199971	0,593100011	0,643100023
1,00E-04	0,697399974	0,751999974	0,722599983	0,754899979

690nm

cocentration [M	Replicate 1	Replicate 2	Replicate 3	Replicate 4
1,00E-10	0,605300009	0,660099983	0,6329	0,639500022
1,00E-09	0,765200019	0,708299994	0,711799979	0,711899996
1,00E-08	0,682399988	0,6778	0,675800025	0,671199977
1,00E-07	0,684599996	0,660300016	0,615800023	0,605000019
1,00E-06	0,595799983	0,640699983	0,585300028	0,602599978
1,00E-05	0,557699978	0,535700023	0,48089999	0,518700004
1,00E-04	0,5528	0,606000006	0,575200021	0,596199989

solvent control 690nm

	Replicate 1	Replicate 2	Replicate 3	Replicate 4
	0,609499991	0,48120001	0,538800001	0,5255

540-690nm

cocentration [M	Replicate 1	Replicate 2	Replicate 3	Replicate 4
1,00E-10	0,087300003	0,097499996	0,093199998	0,096199997
1,00E-09	0,101199999	0,100599997	0,098800004	0,097999997
1,00E-08	0,097800002	0,101400003	0,098800004	0,0986
1,00E-07	0,099200003	0,0999	0,092399999	0,092399999
1,00E-06	0,089599997	0,093900003	0,092200004	0,096500002
1,00E-05	0,128000006	0,1175	0,112199999	0,124399997
1,00E-04	0,144600004	0,145999998	0,147400007	0,158700004

solvent control 540nm-690nm

	Replicate 1	Replicate 2	Replicate 3	Replicate 4
	0,093699999	0,080600001	0,088399999	0,0854

(540nm-690nm)-solvent control (540nm-690nm)

cocentration [M	Replicate 1	Replicate 2	Replicate 3	Replicate 4
1,00E-10	0,000275003	0,010474997	0,006174998	0,009174997
1,00E-09	0,014175	0,013574997	0,011775004	0,010974998
1,00E-08	0,010775002	0,014375003	0,011775004	0,011575
1,00E-07	0,012175003	0,012875	0,005375	0,005375
1,00E-06	0,002574997	0,006875003	0,005175004	0,009475002
1,00E-05	0,040975006	0,030475	0,025175	0,037374998
1,00E-04	0,057575004	0,058974998	0,060375007	0,071675004

(540nm-690nm)-solvent control E2 reference

cocentration [M	Replicate 1	Replicate 2	Replicate 3	Replicate 4
1,00E-09	0,450574981	0,473675	0,586975025	0,509975003

Normalization (substance/1 nM E2)

cocentration [M	Replicate 1	Replicate 2	Replicate 3	Replicate 4	Mean
1,00E-10	0,06%	2,21%	1,05%	1,80%	**1,28%**
1,00E-09	3,15%	2,87%	2,01%	2,15%	**2,54%**
1,00E-08	2,39%	3,03%	2,01%	2,27%	**2,43%**
1,00E-07	2,70%	2,72%	0,92%	1,05%	**1,85%**
1,00E-06	0,57%	1,45%	0,88%	1,86%	**1,19%**
1,00E-05	9,09%	6,43%	4,29%	7,33%	**6,79%**
1,00E-04	12,78%	12,45%	10,29%	14,05%	**12,39%**

yeast-growth/solubility 690nm/solvent control 690nm

cocentration [M	Replicate 1	Replicate 2	Replicate 3	Replicate 4	Mean
1,00E-10	99,31%	137,18%	117,46%	121,69%	**118,91%**
1,00E-09	125,55%	147,19%	132,11%	135,47%	**135,08%**
1,00E-08	111,96%	140,86%	125,43%	127,73%	**126,49%**
1,00E-07	112,32%	137,22%	114,29%	115,13%	**119,74%**
1,00E-06	97,75%	133,15%	108,63%	114,67%	**113,55%**
1,00E-05	91,50%	111,33%	89,25%	98,71%	**97,70%**
1,00E-04	90,70%	125,94%	106,76%	113,45%	**109,21%**

YES fenhexamid fludioxonil EC10

1st experient

540nm

cocentration [M]	Replicate 1	Replicate 2	Replicate 3	Replicate 4
1,00E-10	0,675199986	0,627399981	0,700299978	0,680299997
1,00E-09	0,674499989	0,583400011	0,702600002	0,716199994
1,00E-08	0,707899988	0,654999971	0,751100004	0,70599997
1,00E-07	0,686500013	0,731700003	0,670300007	0,673699975
1,00E-06	0,636500001	0,648400009	0,694800019	0,693199992
1,00E-05	0,699299991	0,656799972	0,712899983	0,672200024
1,00E-04	0,662199974	0,65200001	0,647899985	0,725300014

690nm

cocentration [M]	Replicate 1	Replicate 2	Replicate 3	Replicate 4
1,00E-10	0,582099974	0,540300012	0,604499996	0,583700001
1,00E-09	0,581200004	0,500199974	0,60710001	0,616699994
1,00E-08	0,608099997	0,561800003	0,649200022	0,60769999
1,00E-07	0,590900004	0,632300019	0,576699972	0,57889998
1,00E-06	0,545400023	0,55430001	0,599799991	0,596199989
1,00E-05	0,567499995	0,530300021	0,584999979	0,541000009
1,00E-04	0,513700008	0,506299973	0,499300003	0,576099992

solvent control 690nm

	Replicate 1	Replicate 2	Replicate 3	Replicate 4
	0,682200015	0,687900007	0,636600018	0,56400001

540-690nm

cocentration [M]	Replicate 1	Replicate 2	Replicate 3	Replicate 4
1,00E-10	0,093000002	0,087099999	0,095799997	0,096699998
1,00E-09	0,0933	0,0832	0,0955	0,099399999
1,00E-08	0,099799998	0,093199998	0,101899996	0,098300003
1,00E-07	0,0955	0,0995	0,093500003	0,094800003
1,00E-06	0,0911	0,094099998	0,094999999	0,096900001
1,00E-05	0,131799996	0,126499996	0,127800003	0,131200001
1,00E-04	0,148399994	0,145600006	0,148599997	0,149200007

solvent control 540nm-690nm

	Replicate 1	Replicate 2	Replicate 3	Replicate 4
	0,102700002	0,101300001	0,099600002	0,087499999

(540nm-690nm)-solvent control (540nm-690nm)

cocentration [M]	Replicate 1	Replicate 2	Replicate 3	Replicate 4
1,00E-10	-0,004774999	-0,010675002	-0,001975004	-0,001075003
1,00E-09	-0,004475001	-0,014575001	-0,002275001	0,001624998
1,00E-08	0,002024997	-0,004575003	0,004124995	0,000525001
1,00E-07	-0,002275001	0,001724999	-0,004274998	-0,002974998
1,00E-06	-0,006675001	-0,003675003	-0,002775002	-0,000875
1,00E-05	0,034024995	0,028724995	0,030025002	0,033425
1,00E-04	0,050624993	0,047825005	0,050824996	0,051425006

(540nm-690nm)-solvent control E2 reference

cocentration [M]	Replicate 1	Replicate 2	Replicate 3	Replicate 4
1,00E-09	0,367625009	0,331525009	0,298325013	0,294624995

Normalization (substance/1 nM E2)

cocentration [M]	Replicate 1	Replicate 2	Replicate 3	Replicate 4	Mean
1,00E-10	-1,30%	-3,22%	-0,66%	-0,36%	**-1,39%**
1,00E-09	-1,22%	-4,40%	-0,76%	0,55%	**-1,46%**
1,00E-08	0,55%	-1,38%	1,38%	0,18%	**0,18%**
1,00E-07	-0,62%	0,52%	-1,43%	-1,01%	**-0,64%**
1,00E-06	-1,82%	-1,11%	-0,93%	-0,30%	**-1,04%**
1,00E-05	9,26%	8,66%	10,06%	11,34%	**9,83%**
1,00E-04	13,77%	14,43%	17,04%	17,45%	**15,67%**

yeast-growth/solubility 690nm/solvent control 690nm

cocentration [M]	Replicate 1	Replicate 2	Replicate 3	Replicate 4	Mean
1,00E-10	85,33%	78,54%	94,96%	103,49%	**90,58%**
1,00E-09	85,19%	72,71%	95,37%	109,34%	**90,65%**
1,00E-08	89,14%	81,67%	101,98%	107,75%	**95,13%**
1,00E-07	86,62%	91,92%	90,59%	102,64%	**92,94%**
1,00E-06	83,19%	77,09%	91,89%	95,92%	**87,02%**
1,00E-05	79,95%	80,58%	94,22%	105,71%	**90,11%**
1,00E-04	75,30%	73,60%	78,43%	102,15%	**82,37%**

2nd experient

540nm

cocentration [M]	Replicate 1	Replicate 2	Replicate 3	Replicate 4
1,00E-10	0,527800024	0,559099972	0,560299993	0,714399993
1,00E-09	0,722500026	0,583299994	0,700600028	0,752099991
1,00E-08	0,862399995	0,791499972	0,816600025	0,775699973
1,00E-07	0,826300025	0,728100002	0,74849999	0,761799991
1,00E-06	0,753099978	0,736199975	0,788800001	0,837000012
1,00E-05	0,697300017	0,768400013	0,761300027	0,829100013
1,00E-04	0,808799982	0,725099981	0,723800004	0,76789999

690nm

cocentration [M]	Replicate 1	Replicate 2	Replicate 3	Replicate 4
1,00E-10	0,440600008	0,471199989	0,469999999	0,609200001
1,00E-09	0,619799972	0,491100013	0,597800016	0,645099998
1,00E-08	0,754000008	0,683600008	0,706399977	0,661300004
1,00E-07	0,717499971	0,624899983	0,644500017	0,654399991
1,00E-06	0,644999981	0,630800009	0,680700004	0,698800027
1,00E-05	0,551400006	0,61619997	0,608699977	0,6602
1,00E-04	0,630599976	0,555299997	0,557399988	0,589299977

solvent control 690nm

	Replicate 1	Replicate 2	Replicate 3	Replicate 4
	0,628499985	0,417100012	0,548099995	0,487800002

540-690nm

cocentration [M]	Replicate 1	Replicate 2	Replicate 3	Replicate 4
1,00E-10	0,087099999	0,087899998	0,090300001	0,1052
1,00E-09	0,102700002	0,092200004	0,102799997	0,107000001
1,00E-08	0,108499996	0,107900001	0,110200003	0,114500001
1,00E-07	0,108800001	0,103100002	0,104000002	0,1074
1,00E-06	0,108099997	0,105499998	0,108099997	0,1382
1,00E-05	0,145899996	0,152199998	0,152600005	0,168899998
1,00E-04	0,178200006	0,169799998	0,1664	0,178599998

solvent control 540nm-690nm

	Replicate 1	Replicate 2	Replicate 3	Replicate 4
	0,104699999	0,079999998	0,091700003	0,089599997

(540nm-690nm)-solvent control (540nm-690nm)

cocentration [M]	Replicate 1	Replicate 2	Replicate 3	Replicate 4
1,00E-10	-0,0044	-0,003600001	-0,001199998	0,013700001
1,00E-09	0,011200003	0,000700004	0,011299998	0,015500002
1,00E-08	0,016999997	0,016400002	0,018700004	0,023000002
1,00E-07	0,017300002	0,011600003	0,012500003	0,015900001
1,00E-06	0,016599998	0,013999999	0,016599998	0,046700001
1,00E-05	0,054399997	0,060699999	0,061100006	0,077399999
1,00E-04	0,086700007	0,078299999	0,074900001	0,087099999

(540nm-690nm)-solvent control E2 reference

cocentration [M]	Replicate 1	Replicate 2	Replicate 3	Replicate 4
1,00E-09	0,44219999	0,417399988	0,404699996	0,463799998

Normalization (substance/1 nM E2)

cocentration [M]	Replicate 1	Replicate 2	Replicate 3	Replicate 4	Mean
1,00E-10	-1,00%	-0,86%	-0,30%	2,95%	**0,20%**
1,00E-09	2,53%	0,17%	2,79%	3,34%	**2,21%**
1,00E-08	3,84%	3,93%	4,62%	4,96%	**4,34%**
1,00E-07	3,91%	2,78%	3,09%	3,43%	**3,30%**
1,00E-06	3,75%	3,35%	4,10%	10,07%	**5,32%**
1,00E-05	12,30%	14,54%	15,10%	16,69%	**14,66%**
1,00E-04	19,61%	18,76%	18,51%	18,78%	**18,91%**

yeast-growth/solubility 690nm/solvent control 690nm

cocentration [M]	Replicate 1	Replicate 2	Replicate 3	Replicate 4	Mean
1,00E-10	70,10%	112,97%	85,75%	124,89%	**98,43%**
1,00E-09	98,62%	117,74%	109,07%	132,25%	**114,42%**
1,00E-08	119,97%	163,89%	128,88%	135,57%	**137,08%**
1,00E-07	114,16%	149,82%	117,59%	134,15%	**128,93%**
1,00E-06	102,63%	151,23%	124,19%	143,26%	**130,33%**
1,00E-05	87,73%	147,73%	111,06%	135,34%	**120,47%**
1,00E-04	100,33%	133,13%	101,70%	120,81%	**113,99%**

3rd experiment

540nm

cocentration [M	Replicate 1	Replicate 2	Replicate 3	Replicate 4
1,00E-10	0,738799989	0,542599976	0,765999973	0,655099988
1,00E-09	0,735199988	0,834100008	0,771499991	0,845799983
1,00E-08	0,759500027	0,846300006	0,739799976	0,676699996
1,00E-07	0,672999978	0,787199974	0,730799973	0,768400013
1,00E-06	0,674499989	0,713800013	0,646899998	0,696399987
1,00E-05	0,749100029	0,610300004	0,677600026	0,632600009
1,00E-04	0,725499988	0,671199977	0,751299977	0,720799983

690nm

cocentration [M	Replicate 1	Replicate 2	Replicate 3	Replicate 4
1,00E-10	0,639100015	0,468499988	0,666299999	0,569700003
1,00E-09	0,637499988	0,730199993	0,672399998	0,741400003
1,00E-08	0,656400025	0,738600016	0,64410001	0,604900002
1,00E-07	0,579900026	0,684000015	0,629899979	0,671299994
1,00E-06	0,581200004	0,614099979	0,555800021	0,59799999
1,00E-05	0,602999985	0,47330001	0,539699972	0,497700006
1,00E-04	0,552600026	0,505900025	0,578199983	0,55339998

solvent control 690nm

	Replicate 1	Replicate 2	Replicate 3	Replicate 4
	0,610000014	0,414299995	0,659300029	0,559700012

540-690nm

cocentration [M	Replicate 1	Replicate 2	Replicate 3	Replicate 4
1,00E-10	0,099699996	0,074100003	0,099699996	0,085500002
1,00E-09	0,097599998	0,104000002	0,099100001	0,104400001
1,00E-08	0,103100002	0,107699998	0,095700003	0,071699999
1,00E-07	0,093099996	0,103200004	0,100900002	0,097000003
1,00E-06	0,0933	0,099600002	0,0911	0,098399997
1,00E-05	0,1461	0,136899993	0,137799993	0,134900004
1,00E-04	0,172900006	0,165299997	0,173099995	0,167400002

solvent control 540nm-690nm

	Replicate 1	Replicate 2	Replicate 3	Replicate 4
	0,098800004	0,0757	0,103	0,093900003

(540nm-690nm)-solvent control (540nm-690nm)

cocentration [M	Replicate 1	Replicate 2	Replicate 3	Replicate 4
1,00E-10	0,006849995	-0,018749999	0,006849995	-0,00735
1,00E-09	0,004749997	0,011150001	0,00625	0,01155
1,00E-08	0,01025	0,014849996	0,002850002	-0,021150002
1,00E-07	0,000249995	0,010350002	0,00805	0,004150001
1,00E-06	0,000449998	0,006750001	-0,001750002	0,005549995
1,00E-05	0,053249998	0,044049991	0,044949992	0,042050002
1,00E-04	0,080050005	0,072449995	0,080249993	0,074550001

(540nm-690nm)-solvent control E2 reference

cocentration [M	Replicate 1	Replicate 2	Replicate 3	Replicate 4
1,00E-09	0,456450013	0,511050014	0,461550025	0,480150012

Normalization (substance/1 nM E2)

cocentration [M	Replicate 1	Replicate 2	Replicate 3	Replicate 4	Mean
1,00E-10	1,50%	-3,67%	1,48%	-1,53%	**-0,55%**
1,00E-09	1,04%	2,18%	1,35%	2,41%	**1,75%**
1,00E-08	2,25%	2,91%	0,62%	-4,40%	**0,34%**
1,00E-07	0,05%	2,03%	1,74%	0,86%	**1,17%**
1,00E-06	0,10%	1,32%	-0,38%	1,16%	**0,55%**
1,00E-05	11,67%	8,62%	9,74%	8,76%	**9,70%**
1,00E-04	17,54%	14,18%	17,39%	15,53%	**16,16%**

yeast-growth/solubility 690nm/solvent control 690nm

cocentration [M	Replicate 1	Replicate 2	Replicate 3	Replicate 4	Mean
1,00E-10	104,77%	113,08%	101,06%	101,79%	**105,18%**
1,00E-09	104,51%	176,25%	101,99%	132,46%	**128,80%**
1,00E-08	107,61%	178,28%	97,69%	108,08%	**122,91%**
1,00E-07	95,07%	165,10%	95,54%	119,94%	**118,91%**
1,00E-06	95,28%	148,23%	84,30%	106,84%	**108,66%**
1,00E-05	98,85%	114,24%	81,86%	88,92%	**95,97%**
1,00E-04	90,59%	122,11%	87,70%	98,87%	**99,82%**

4th experiment

540nm

cocentration [M	Replicate 1	Replicate 2	Replicate 3	Replicate 4
1,00E-10	0,710699975	0,807200015	0,736899972	0,777100027
1,00E-09	0,746200025	0,715600014	0,680000007	0,721199989
1,00E-08	0,742600024	0,716600001	0,738099992	0,786499977
1,00E-07	0,72240001	0,704999983	0,739600003	0,692900002
1,00E-06	0,66900003	0,650099993	0,683399975	0,710300028
1,00E-05	0,594799995	0,651600003	0,65140003	0,705799997
1,00E-04	0,662999988	0,696099997	0,648400009	0,684400022

690nm

cocentration [M	Replicate 1	Replicate 2	Replicate 3	Replicate 4
1,00E-10	0,60769999	0,701699972	0,630900025	0,667999983
1,00E-09	0,64139998	0,612200022	0,580500007	0,614400029
1,00E-08	0,637300014	0,616299987	0,63499999	0,677299976
1,00E-07	0,621399999	0,604700029	0,634199977	0,59009999
1,00E-06	0,568799973	0,550700009	0,581900001	0,604300022
1,00E-05	0,475300014	0,52640003	0,526899993	0,573000014
1,00E-04	0,526600003	0,554700017	0,516200006	0,538600028

solvent control 690nm

	Replicate 1	Replicate 2	Replicate 3	Replicate 4
	0,590499997	0,568000019	0,572300017	0,475899994

540-690nm

cocentration [M	Replicate 1	Replicate 2	Replicate 3	Replicate 4
1,00E-10	0,103	0,105499998	0,105999999	0,109200001
1,00E-09	0,104800001	0,103299998	0,0995	0,106799997
1,00E-08	0,105300002	0,100299999	0,103100002	0,109200001
1,00E-07	0,101000004	0,100299999	0,105400003	0,102799997
1,00E-06	0,100199997	0,099399999	0,101400003	0,105999999
1,00E-05	0,119499996	0,125200003	0,124399997	0,132799998
1,00E-04	0,136500001	0,141399994	0,132300004	0,145799994

solvent control 540nm-690nm

	Replicate 1	Replicate 2	Replicate 3	Replicate 4
	0,1052	0,101499997	0,102200001	0,089000002

(540nm-690nm)-solvent control (540nm-690nm)

cocentration [M	Replicate 1	Replicate 2	Replicate 3	Replicate 4
1,00E-10	0,003525	0,006024998	0,006524999	0,009725001
1,00E-09	0,005325001	0,003824998	2,50004E-05	0,007324997
1,00E-08	0,005825002	0,000824999	0,003625002	0,009725001
1,00E-07	0,001525003	0,000824999	0,005925003	0,003324997
1,00E-06	0,000724997	-7,50013E-05	0,001925003	0,006524999
1,00E-05	0,020024996	0,025725003	0,024924997	0,033324998
1,00E-04	0,037025001	0,041924994	0,032825004	0,046324994

(540nm-690nm)-solvent control E2 reference

cocentration [M	Replicate 1	Replicate 2	Replicate 3	Replicate 4
1,00E-09	0,422525015	0,406025019	0,402924981	0,404024985

Normalization (substance/1 nM E2)

cocentration [M	Replicate 1	Replicate 2	Replicate 3	Replicate 4	Mean
1,00E-10	0,83%	1,48%	1,62%	2,41%	**1,59%**
1,00E-09	1,26%	0,94%	0,01%	1,81%	**1,01%**
1,00E-08	1,38%	0,20%	0,90%	2,41%	**1,22%**
1,00E-07	0,36%	0,20%	1,47%	0,82%	**0,71%**
1,00E-06	0,17%	-0,02%	0,48%	1,61%	**0,56%**
1,00E-05	4,74%	6,34%	6,19%	8,25%	**6,38%**
1,00E-04	8,76%	10,33%	8,15%	11,47%	**9,68%**

yeast-growth/solubility 690nm/solvent control 690nm

cocentration [M	Replicate 1	Replicate 2	Replicate 3	Replicate 4	Mean
1,00E-10	102,91%	123,54%	110,24%	140,37%	**119,26%**
1,00E-09	108,62%	107,78%	101,43%	129,10%	**111,73%**
1,00E-08	107,93%	108,50%	110,96%	142,32%	**117,43%**
1,00E-07	105,23%	106,46%	110,82%	124,00%	**111,63%**
1,00E-06	96,33%	96,95%	101,68%	126,98%	**105,48%**
1,00E-05	80,49%	92,68%	92,07%	120,40%	**96,41%**
1,00E-04	89,18%	97,66%	90,20%	113,18%	**97,55%**

5th experiment

540nm

cocentration [M	Replicate 1	Replicate 2	Replicate 3	Replicate 4
1,00E-10	0,523199975	0,717499971	0,69749999	0,647499979
1,00E-09	0,770099998	0,782000005	0,739899993	0,650399983
1,00E-08	0,770399988	0,694400012	0,646799982	0,64319998
1,00E-07	0,7421	0,713400006	0,718100011	0,751399994
1,00E-06	0,74089998	0,774500012	0,696099997	0,572399974
1,00E-05	0,725199997	0,670599997	0,670000017	0,664799988
1,00E-04	0,807500005	0,817900002	0,839299977	0,812699974

690nm

cocentration [M	Replicate 1	Replicate 2	Replicate 3	Replicate 4
1,00E-10	0,445100009	0,621800005	0,602199972	0,548900008
1,00E-09	0,671700001	0,681500018	0,640200019	0,555400014
1,00E-08	0,673099995	0,598100007	0,554700017	0,547399998
1,00E-07	0,646700025	0,618700027	0,619099975	0,650600016
1,00E-06	0,645399988	0,661000013	0,597299993	0,486799985
1,00E-05	0,603799999	0,552299976	0,552399993	0,539699972
1,00E-04	0,655399978	0,662100017	0,684400022	0,650200009

solvent control 690nm

	Replicate 1	Replicate 2	Replicate 3	Replicate 4
	0,609499991	0,48120001	0,538800001	0,5255

540-690nm

cocentration [M	Replicate 1	Replicate 2	Replicate 3	Replicate 4
1,00E-10	0,078100003	0,095700003	0,095399998	0,0986
1,00E-09	0,098399997	0,100500003	0,099699996	0,094999999
1,00E-08	0,0973	0,096299998	0,092100002	0,095799997
1,00E-07	0,095399998	0,094700001	0,098999999	0,1008
1,00E-06	0,095600002	0,113499999	0,098800004	0,085699998
1,00E-05	0,121299997	0,118299998	0,117600001	0,125100002
1,00E-04	0,152099997	0,1558	0,154899999	0,162499994

solvent control 540nm-690nm

	Replicate 1	Replicate 2	Replicate 3	Replicate 4
	0,093699999	0,080600001	0,088399999	0,0854

(540nm-690nm)-solvent control (540nm-690nm)

cocentration [M	Replicate 1	Replicate 2	Replicate 3	Replicate 4
1,00E-10	-0,008924996	0,008675003	0,008374998	0,011575
1,00E-09	0,011374997	0,013475003	0,012674997	0,007974999
1,00E-08	0,010275001	0,009274999	0,005075002	0,008774998
1,00E-07	0,008374998	0,007675001	0,011975	0,013775
1,00E-06	0,008575002	0,026474999	0,011775004	-0,001325002
1,00E-05	0,034274997	0,031274999	0,030575002	0,038075002
1,00E-04	0,065074997	0,068775	0,067875	0,075474994

(540nm-690nm)-solvent control E2 reference

cocentration [M	Replicate 1	Replicate 2	Replicate 3	Replicate 4
1,00E-09	0,450574981	0,473675	0,586975025	0,509975003

Normalization (substance/1 nM E2)

cocentration [M	Replicate 1	Replicate 2	Replicate 3	Replicate 4	Mean
1,00E-10	-1,98%	1,83%	1,43%	2,27%	**0,89%**
1,00E-09	2,52%	2,84%	2,16%	1,56%	**2,27%**
1,00E-08	2,28%	1,96%	0,86%	1,72%	**1,71%**
1,00E-07	1,86%	1,62%	2,04%	2,70%	**2,06%**
1,00E-06	1,90%	5,59%	2,01%	-0,26%	**2,31%**
1,00E-05	7,61%	6,60%	5,21%	7,47%	**6,72%**
1,00E-04	14,44%	14,52%	11,56%	14,80%	**13,83%**

yeast-growth/solubility 690nm/solvent control 690nm

cocentration [M	Replicate 1	Replicate 2	Replicate 3	Replicate 4	Mean
1,00E-10	73,03%	129,22%	111,77%	104,45%	**104,62%**
1,00E-09	110,21%	141,63%	118,82%	105,69%	**119,08%**
1,00E-08	110,43%	124,29%	102,95%	104,17%	**110,46%**
1,00E-07	106,10%	128,57%	114,90%	123,81%	**118,35%**
1,00E-06	105,89%	137,36%	110,86%	92,64%	**111,69%**
1,00E-05	99,06%	114,78%	102,52%	102,70%	**104,77%**
1,00E-04	107,53%	137,59%	127,02%	123,73%	**123,97%**

YES E2 chlorpyrifos fenarimol EC101

1st experient

540nm

cocentration [M]	Replicate 1	Replicate 2	Replicate 3	Replicate 4
1,00E-06	0,889199972	0,836399972	0,85860002	0,835500002
3,00E-06	0,850399971	0,853399992	0,850499988	0,822300017
6,00E-06	0,887000024	0,94569999	0,8301	0,9005
1,00E-05	0,904799998	0,893000007	0,899200022	0,862100005
3,00E-05	1,130300045	1,109099984	1,103199959	1,067499995
6,00E-05	1,899600029	1,833099961	1,835299969	1,815099955
1,00E-04	2,283200026	2,230200052	2,235899925	2,281500101

690nm

cocentration [M]	Replicate 1	Replicate 2	Replicate 3	Replicate 4
1,00E-06	0,488599986	0,435099989	0,459199995	0,441199988
3,00E-06	0,459300011	0,463400006	0,449200004	0,434199989
6,00E-06	0,486200005	0,528699994	0,437900007	0,490799993
1,00E-05	0,493400007	0,495499998	0,478899986	0,466399997
3,00E-05	0,505800009	0,489800006	0,476700008	0,471300006
6,00E-05	0,468899995	0,512799978	0,466399997	0,464899987
1,00E-04	0,48269999	0,567600012	0,442200005	0,435499996

solvent control 690nm

	Replicate 1	Replicate 2	Replicate 3	Replicate 4
	0,544399977	0,614700019	0,305799991	0,601199985

540-690nm

cocentration [M]	Replicate 1	Replicate 2	Replicate 3	Replicate 4
1,00E-06	0,400599986	0,401300013	0,399399996	0,394300014
3,00E-06	0,391200006	0,389999986	0,401300013	0,388099998
6,00E-06	0,40079999	0,416999996	0,392199993	0,409700006
1,00E-05	0,41139999	0,397500008	0,42019999	0,395700008
3,00E-05	0,624499977	0,619300008	0,62650001	0,596199989
6,00E-05	1,430799961	1,320299983	1,368899941	1,350200057
1,00E-04	1,800400019	1,66260004	1,793799996	1,845999956

solvent control 540nm-690nm

	Replicate 1	Replicate 2	Replicate 3	Replicate 4
	0,087700002	0,098200001	0,057	0,084200002

(540nm-690nm)-solvent control (540nm-690nm)

cocentration [M]	Replicate 1	Replicate 2	Replicate 3	Replicate 4
1,00E-06	0,318824985	0,319525012	0,317624995	0,312525013
3,00E-06	0,309425005	0,308224984	0,319525012	0,306324997
6,00E-06	0,319024988	0,335224994	0,310424992	0,327925005
1,00E-05	0,329624989	0,315725007	0,338424989	0,313925006
3,00E-05	0,542724975	0,537525007	0,544725009	0,514424988
6,00E-05	1,34902496	1,238524982	1,28712494	1,268425056
1,00E-04	1,718625017	1,580825039	1,712024995	1,764224955

(540nm-690nm)-solvent control E2 reference

cocentration [M]	Replicate 1	Replicate 2	Replicate 3	Replicate 4
1,00E-09	0,40610002	0,311099991	0,297400013	0,334099993

Normalization (substance/1 nM E2)

cocentration [M]	Replicate 1	Replicate 2	Replicate 3	Replicate 4	Mean
1,00E-06	78,51%	102,71%	106,80%	93,54%	**95,39%**
3,00E-06	76,19%	99,08%	107,44%	91,69%	**93,60%**
6,00E-06	78,56%	107,75%	104,38%	98,15%	**97,21%**
1,00E-05	81,17%	101,49%	113,79%	93,96%	**97,60%**
3,00E-05	133,64%	172,78%	183,16%	153,97%	**160,89%**
6,00E-05	332,19%	398,11%	432,79%	379,65%	**385,69%**
1,00E-04	423,20%	508,14%	575,66%	528,05%	**508,76%**

yeast-growth/solubility 690nm/solvent control 690nm

cocentration [M]	Replicate 1	Replicate 2	Replicate 3	Replicate 4	Mean
1,00E-06	89,75%	70,78%	150,16%	73,39%	**96,02%**
3,00E-06	84,37%	75,39%	146,89%	72,22%	**94,72%**
6,00E-06	89,31%	86,01%	143,20%	81,64%	**100,04%**
1,00E-05	90,63%	80,61%	156,61%	77,58%	**101,36%**
3,00E-05	86,13%	83,42%	152,52%	77,33%	**99,85%**
6,00E-05	92,91%	79,68%	155,89%	78,39%	**101,72%**
1,00E-04	88,67%	92,34%	144,60%	72,44%	**99,51%**

2nd experient

540nm

cocentration [M]	Replicate 1	Replicate 2	Replicate 3	Replicate 4
1,00E-06	1,086899996	0,91960001	0,885200024	0,960799992
3,00E-06	0,974900007	0,996999979	0,944700003	1,067299962
6,00E-06	0,973100007	0,994199991	0,968200028	1,10710001
1,00E-05	0,988699973	0,977199972	0,919099987	0,996500015
3,00E-05	1,441300035	1,423699975	1,4296	1,452499986
6,00E-05	2,367000103	2,389199972	2,358500004	2,410399914
1,00E-04	2,648699999	2,605900049	2,640399933	2,596299887

690nm

cocentration [M]	Replicate 1	Replicate 2	Replicate 3	Replicate 4
1,00E-06	0,483200014	0,438499987	0,3935	0,454699993
3,00E-06	0,488999993	0,500400007	0,461400002	0,559300005
6,00E-06	0,500500023	0,496399999	0,480399996	0,56220001
1,00E-05	0,507399976	0,516200006	0,465299994	0,515500009
3,00E-05	0,595300019	0,555899978	0,578700006	0,547200024
6,00E-05	0,550000012	0,524999976	0,54339999	0,517400026
1,00E-04	0,493499994	0,48089999	0,513700008	0,485900015

solvent control 690nm

	Replicate 1	Replicate 2	Replicate 3	Replicate 4
	0,623199999	0,583700001	0,487699986	0,664200008

540-690nm

cocentration [M]	Replicate 1	Replicate 2	Replicate 3	Replicate 4
1,00E-06	0,603699982	0,481099993	0,491699994	0,506099999
3,00E-06	0,486000001	0,496600002	0,4833	0,508000016
6,00E-06	0,4727	0,497799993	0,487800002	0,5449
1,00E-05	0,481299996	0,460900009	0,453799993	0,481000006
3,00E-05	0,846000016	0,867799997	0,850899994	0,905300021
6,00E-05	1,817000031	1,864199996	1,815199971	1,893000007
1,00E-04	2,155100107	2,125099897	2,12680006	2,110399961

solvent control 540nm-690nm

	Replicate 1	Replicate 2	Replicate 3	Replicate 4
	0,108099997	0,111100003	0,100299999	0,116400003

(540nm-690nm)-solvent control (540nm-690nm)

cocentration [M]	Replicate 1	Replicate 2	Replicate 3	Replicate 4
1,00E-06	0,494724981	0,372124992	0,382724993	0,397124998
3,00E-06	0,377025001	0,387625001	0,374325	0,399025016
6,00E-06	0,363724999	0,388824992	0,378825001	0,435924999
1,00E-05	0,372324996	0,351925008	0,344824992	0,372025006
3,00E-05	0,737025015	0,758824997	0,741924994	0,79632502
6,00E-05	1,708025031	1,755224995	1,706224971	1,784025006
1,00E-04	2,046125107	2,016124897	2,01782506	2,001424961

(540nm-690nm)-solvent control E2 reference

cocentration [M]	Replicate 1	Replicate 2	Replicate 3	Replicate 4
1,00E-09	0,431299984	0,381599993	0,374599993	0,377200007

Normalization (substance/1 nM E2)

cocentration [M]	Replicate 1	Replicate 2	Replicate 3	Replicate 4	Mean
1,00E-06	114,71%	97,52%	102,17%	105,28%	**104,92%**
3,00E-06	87,42%	101,58%	99,93%	105,79%	**98,68%**
6,00E-06	84,33%	101,89%	101,13%	115,57%	**100,73%**
1,00E-05	86,33%	92,22%	92,05%	98,63%	**92,31%**
3,00E-05	170,88%	198,85%	198,06%	211,11%	**194,73%**
6,00E-05	396,02%	459,96%	455,48%	472,97%	**446,11%**
1,00E-04	474,41%	528,33%	538,66%	530,60%	**518,00%**

yeast-growth/solubility 690nm/solvent control 690nm

cocentration [M]	Replicate 1	Replicate 2	Replicate 3	Replicate 4	Mean
1,00E-06	77,54%	75,12%	80,68%	68,46%	**75,45%**
3,00E-06	78,47%	85,73%	94,61%	84,21%	**85,75%**
6,00E-06	80,31%	85,04%	98,50%	84,64%	**87,13%**
1,00E-05	81,42%	88,44%	95,41%	77,61%	**85,72%**
3,00E-05	95,52%	95,24%	118,66%	82,38%	**97,95%**
6,00E-05	88,25%	89,94%	111,42%	77,90%	**91,88%**
1,00E-04	79,19%	82,39%	105,33%	73,16%	**85,02%**

3rd experiment

540nm

cocentration [M	Replicate 1	Replicate 2	Replicate 3	Replicate 4
1,00E-06	1,150099993	1,114199996	1,097800016	1,016000032
3,00E-06	1,078799963	1,044199944	1,090299964	1,094599962
6,00E-06	1,119899988	1,157299995	1,01880002	1,020799994
1,00E-05	1,107499957	1,063199997	1,045099974	1,059599996
3,00E-05	1,284700036	1,227800012	1,237300038	1,280699968
6,00E-05	2,1796	2,059400082	2,042599916	2,022000074
1,00E-04	2,440900087	2,39989996	2,415299892	2,410099983

690nm

cocentration [M	Replicate 1	Replicate 2	Replicate 3	Replicate 4
1,00E-06	0,528100014	0,5	0,495000005	0,443100005
3,00E-06	0,483500004	0,467999995	0,506600022	0,502099991
6,00E-06	0,537	0,528800011	0,461800009	0,461199999
1,00E-05	0,523299992	0,498299986	0,495400012	0,511099994
3,00E-05	0,542299986	0,515299976	0,513000011	0,520399988
6,00E-05	0,511600018	0,491699994	0,480199993	0,480199993
1,00E-04	0,470800012	0,467299998	0,444099993	0,451799989

solvent control 690nm

	Replicate 1	Replicate 2	Replicate 3	Replicate 4
	0,667299986	0,712599993	0,666199982	0,557500005

540-690nm

cocentration [M	Replicate 1	Replicate 2	Replicate 3	Replicate 4
1,00E-06	0,621999979	0,614199996	0,602800012	0,572899997
3,00E-06	0,595300019	0,576200008	0,583700001	0,592499971
6,00E-06	0,582899988	0,628499985	0,556999981	0,559599996
1,00E-05	0,584200025	0,564899981	0,549700022	0,548500001
3,00E-05	0,742399991	0,712599993	0,724300027	0,760299981
6,00E-05	1,667999983	1,567700028	1,562299967	1,541700006
1,00E-04	1,970100045	1,932600021	1,971099973	1,958400011

solvent control 540nm-690nm

	Replicate 1	Replicate 2	Replicate 3	Replicate 4
	0,102899998	0,103600003	0,106399998	0,083099999

(540nm-690nm)-solvent control (540nm-690nm)

cocentration [M	Replicate 1	Replicate 2	Replicate 3	Replicate 4
1,00E-06	0,52299998	0,515199997	0,503800012	0,473899998
3,00E-06	0,496300019	0,477200009	0,484700002	0,493499972
6,00E-06	0,483899988	0,529499985	0,457999982	0,460599996
1,00E-05	0,485200025	0,465899982	0,450700022	0,449500002
3,00E-05	0,643399991	0,613599993	0,625300027	0,661299981
6,00E-05	1,568999983	1,468700029	1,463299967	1,442700006
1,00E-04	1,871100046	1,833600022	1,872099973	1,859400012

(540nm-690nm)-solvent control E2 reference

cocentration [M	Replicate 1	Replicate 2	Replicate 3	Replicate 4
1,00E-09	0,526925027	0,468824983	0,49712503	0,507725

Normalization (substance/1 nM E2)

cocentration [M	Replicate 1	Replicate 2	Replicate 3	Replicate 4	Mean
1,00E-06	99,26%	109,89%	101,34%	93,34%	**100,96%**
3,00E-06	94,19%	101,79%	97,50%	97,20%	**97,67%**
6,00E-06	91,83%	112,94%	92,13%	90,72%	**96,91%**
1,00E-05	92,08%	99,38%	90,66%	88,53%	**92,66%**
3,00E-05	122,10%	130,88%	125,78%	130,25%	**127,25%**
6,00E-05	297,77%	313,27%	294,35%	284,15%	**297,39%**
1,00E-04	355,10%	391,11%	376,59%	366,22%	**372,25%**

yeast-growth/solubility 690nm/solvent control 690nm

cocentration [M	Replicate 1	Replicate 2	Replicate 3	Replicate 4	Mean
1,00E-06	79,14%	70,17%	74,30%	79,48%	**75,77%**
3,00E-06	72,46%	65,67%	76,04%	90,06%	**76,06%**
6,00E-06	80,47%	74,21%	69,32%	82,73%	**76,68%**
1,00E-05	78,42%	69,93%	74,36%	91,68%	**78,60%**
3,00E-05	81,27%	72,31%	77,00%	93,35%	**80,98%**
6,00E-05	76,67%	69,00%	72,08%	86,13%	**75,97%**
1,00E-04	70,55%	65,58%	66,66%	81,04%	**70,96%**

4th experiment

540nm

cocentration [M	Replicate 1	Replicate 2	Replicate 3	Replicate 4
1,00E-06	0,913900018	0,915700018	0,887199998	0,91109997
3,00E-06	0,869799972	0,973100007	0,934899986	0,913699985
6,00E-06	1,011999965	0,921400011	0,897599995	0,902499974
1,00E-05	0,898299992	1,021700025	0,9551	0,98299998
3,00E-05	1,088600039	1,014999986	1,052700043	1,007799983
6,00E-05	1,992599964	1,874600053	1,903100014	1,824399948
1,00E-04	2,198800087	2,15260005	2,130100012	2,193399906

690nm

cocentration [M	Replicate 1	Replicate 2	Replicate 3	Replicate 4
1,00E-06	0,478799999	0,477200001	0,472200006	0,478100002
3,00E-06	0,444599986	0,496899992	0,497399986	0,474400014
6,00E-06	0,510299981	0,495799989	0,469399989	0,48210001
1,00E-05	0,474099994	0,558700025	0,529200017	0,543600023
3,00E-05	0,488999993	0,452600002	0,484299988	0,452899992
6,00E-05	0,571099997	0,465499997	0,453200012	0,467200011
1,00E-04	0,455000013	0,436199993	0,436800003	0,460399985

solvent control 690nm

	Replicate 1	Replicate 2	Replicate 3	Replicate 4
	0,581399977	0,580600023	0,595700026	0,596700013

540-690nm

cocentration [M	Replicate 1	Replicate 2	Replicate 3	Replicate 4
1,00E-06	0,435000002	0,438499987	0,414999992	0,432999998
3,00E-06	0,425199986	0,476099998	0,437400013	0,439300001
6,00E-06	0,501699984	0,425599992	0,428200006	0,420399994
1,00E-05	0,424299985	0,463099986	0,425900012	0,439399987
3,00E-05	0,599600017	0,5625	0,568400025	0,554899991
6,00E-05	1,421599984	1,409199953	1,449900031	1,357200027
1,00E-04	1,743800044	1,716400027	1,693199992	1,733100057

solvent control 540nm-690nm

	Replicate 1	Replicate 2	Replicate 3	Replicate 4
	0,097800002	0,098899998	0,102399997	0,100500003

(540nm-690nm)-solvent control (540nm-690nm)

cocentration [M	Replicate 1	Replicate 2	Replicate 3	Replicate 4
1,00E-06	0,335100003	0,338599987	0,315099992	0,333099999
3,00E-06	0,325299986	0,376199998	0,337500013	0,339400001
6,00E-06	0,401799984	0,325699992	0,328300007	0,320499994
1,00E-05	0,324399985	0,363199987	0,326000012	0,339499988
3,00E-05	0,499700017	0,4626	0,468500026	0,454999991
6,00E-05	1,321699984	1,309299953	1,350000031	1,257300027
1,00E-04	1,643900044	1,616500027	1,593299992	1,633200057

(540nm-690nm)-solvent control E2 reference

cocentration [M	Replicate 1	Replicate 2	Replicate 3	Replicate 4
1,00E-09	0,379349984	0,384850003	0,386549987	0,385450013

Normalization (substance/1 nM E2)

cocentration [M	Replicate 1	Replicate 2	Replicate 3	Replicate 4	Mean
1,00E-06	88,34%	87,98%	81,52%	86,42%	**86,06%**
3,00E-06	85,75%	97,75%	87,31%	88,05%	**89,72%**
6,00E-06	105,92%	84,63%	84,93%	83,15%	**89,66%**
1,00E-05	85,51%	94,37%	84,34%	88,08%	**88,08%**
3,00E-05	131,73%	120,20%	121,20%	118,04%	**122,79%**
6,00E-05	348,41%	340,21%	349,24%	326,19%	**341,01%**
1,00E-04	433,35%	420,03%	412,18%	423,71%	**422,32%**

yeast-growth/solubility 690nm/solvent control 690nm

cocentration [M	Replicate 1	Replicate 2	Replicate 3	Replicate 4	Mean
1,00E-06	82,35%	82,19%	79,27%	80,12%	**80,98%**
3,00E-06	76,47%	85,58%	83,50%	79,50%	**81,26%**
6,00E-06	87,77%	85,39%	78,80%	80,79%	**83,19%**
1,00E-05	81,54%	96,23%	88,84%	91,10%	**89,43%**
3,00E-05	84,11%	77,95%	81,30%	75,90%	**79,82%**
6,00E-05	98,23%	80,18%	76,08%	78,30%	**83,19%**
1,00E-04	78,26%	75,13%	73,33%	77,16%	**75,97%**

5th experiment

540nm

cocentration [M	Replicate 1	Replicate 2	Replicate 3	Replicate 4
1,00E-06	1,102399945	1,168499947	1,050899982	1,093099952
3,00E-06	1,104400039	1,105200052	1,096300006	1,12440002
6,00E-06	1,046499968	1,090499997	1,052899957	1,116899967
1,00E-05	1,02760005	1,161800027	1,051499963	1,124199986
3,00E-05	1,351699948	1,506000042	1,432999969	1,534700036
6,00E-05	2,481600046	2,461699963	2,474400043	2,467999935
1,00E-04	2,430599928	2,45810008	2,423099995	2,439800024

690nm

cocentration [M	Replicate 1	Replicate 2	Replicate 3	Replicate 4
1,00E-06	0,524900019	0,564199984	0,495700002	0,535600007
3,00E-06	0,541000009	0,53579998	0,550100029	0,573199987
6,00E-06	0,511200011	0,546400011	0,520399988	0,58160001
1,00E-05	0,521799982	0,606100023	0,561999977	0,608099997
3,00E-05	0,600199997	0,599699974	0,644599974	0,60860002
6,00E-05	0,541299999	0,521200001	0,555199981	0,537999988
1,00E-04	0,468100011	0,498299986	0,514299989	0,540000021

solvent control 690nm

	Replicate 1	Replicate 2	Replicate 3	Replicate 4
	0,595899999	0,614499986	0,58920002	0,620999992

540-690nm

cocentration [M	Replicate 1	Replicate 2	Replicate 3	Replicate 4
1,00E-06	0,577499986	0,604399979	0,555199981	0,557399988
3,00E-06	0,563399971	0,569500029	0,546199977	0,551299989
6,00E-06	0,535399973	0,544099987	0,532599986	0,535300016
1,00E-05	0,505800009	0,555700004	0,489399999	0,516099989
3,00E-05	0,751399994	0,906199992	0,788399994	0,926100016
6,00E-05	1,940199971	1,940500021	1,919199944	1,929999948
1,00E-04	1,962599993	1,959699988	1,908800006	1,899799943

solvent control 540nm-690nm

	Replicate 1	Replicate 2	Replicate 3	Replicate 4
	0,096299998	0,096100003	0,097099997	0,101199999

(540nm-690nm)-solvent control (540nm-690nm)

cocentration [M	Replicate 1	Replicate 2	Replicate 3	Replicate 4
1,00E-06	0,479824986	0,50672498	0,457524981	0,459724989
3,00E-06	0,465724971	0,47182503	0,448524978	0,45362499
6,00E-06	0,437724974	0,446424987	0,434924986	0,437625017
1,00E-05	0,408125009	0,458025005	0,391725	0,41842499
3,00E-05	0,653724995	0,808524992	0,690724995	0,828425016
6,00E-05	1,842524972	1,842825022	1,821524944	1,832324948
1,00E-04	1,864924993	1,862024989	1,811125007	1,802124944

(540nm-690nm)-solvent control E2 reference

cocentration [M	Replicate 1	Replicate 2	Replicate 3	Replicate 4
1,00E-09	0,466699993	0,465999996	0,499100005	0,480500018

Normalization (substance/1 nM E2)

cocentration [M	Replicate 1	Replicate 2	Replicate 3	Replicate 4	Mean
1,00E-06	102,81%	108,74%	91,67%	95,68%	**99,72%**
3,00E-06	99,79%	101,25%	89,87%	94,41%	**96,33%**
6,00E-06	93,79%	95,80%	87,14%	91,08%	**91,95%**
1,00E-05	87,45%	98,29%	78,49%	87,08%	**87,83%**
3,00E-05	140,07%	173,50%	138,39%	172,41%	**156,10%**
6,00E-05	394,80%	395,46%	364,96%	381,34%	**384,14%**
1,00E-04	399,60%	399,58%	362,88%	375,05%	**384,28%**

yeast-growth/solubility 690nm/solvent control 690nm

cocentration [M	Replicate 1	Replicate 2	Replicate 3	Replicate 4	Mean
1,00E-06	88,09%	91,81%	84,13%	86,25%	**87,57%**
3,00E-06	90,79%	87,19%	93,36%	92,30%	**90,91%**
6,00E-06	85,79%	88,92%	88,32%	93,66%	**89,17%**
1,00E-05	87,57%	98,63%	95,38%	97,92%	**94,88%**
3,00E-05	100,72%	97,59%	109,40%	98,00%	**101,43%**
6,00E-05	90,84%	84,82%	94,23%	86,63%	**89,13%**
1,00E-04	78,55%	81,09%	87,29%	86,96%	**83,47%**

YES E2 chlorpyrifos fenarimol EC110

1st experient

540nm

cocentration [M]	Replicate 1	Replicate 2	Replicate 3	Replicate 4
1,00E-06	0,918600023	0,928300023	0,89230001	0,906199992
3,00E-06	0,880800009	0,878700018	0,878799975	0,955799997
6,00E-06	0,86559999	0,867600024	0,944599986	0,843200028
1,00E-05	0,89230001	0,889299989	0,912199974	0,907800019
3,00E-05	1,019199967	1,068400025	1,055199981	1,093700051
6,00E-05	1,737200022	1,603500009	1,753499985	1,841899991
1,00E-04	2,094700098	2,05340004	2,052099943	2,109499931

690nm

cocentration [M]	Replicate 1	Replicate 2	Replicate 3	Replicate 4
1,00E-06	0,491400003	0,497200012	0,465700001	0,465799987
3,00E-06	0,470699996	0,474099994	0,467000008	0,496699989
6,00E-06	0,459699988	0,46540001	0,497799993	0,435200006
1,00E-05	0,479900002	0,478100002	0,486000001	0,475400001
3,00E-05	0,467599988	0,506500006	0,488999993	0,493900001
6,00E-05	0,479200006	0,476599991	0,478100002	0,510900021
1,00E-04	0,468199998	0,469099998	0,466100007	0,463499993

solvent control 690nm

	Replicate 1	Replicate 2	Replicate 3	Replicate 4
	0,544399977	0,614700019	0,305799991	0,601199985

540-690nm

cocentration [M]	Replicate 1	Replicate 2	Replicate 3	Replicate 4
1,00E-06	0,427100003	0,431100011	0,426600009	0,440400004
3,00E-06	0,409999996	0,404599994	0,411799997	0,459100008
6,00E-06	0,405900002	0,402200013	0,446799994	0,407999992
1,00E-05	0,412400007	0,411199987	0,426099986	0,432399988
3,00E-05	0,551699996	0,561800003	0,566200018	0,599799991
6,00E-05	1,2579	1,126899958	1,275400043	1,33099997
1,00E-04	1,626600027	1,584400058	1,585999966	1,646000028

solvent control 540nm-690nm

	Replicate 1	Replicate 2	Replicate 3	Replicate 4
	0,087700002	0,098200001	0,057	0,084200002

(540nm-690nm)-solvent control (540nm-690nm)

cocentration [M]	Replicate 1	Replicate 2	Replicate 3	Replicate 4
1,00E-06	0,345325002	0,34932501	0,344825008	0,358625003
3,00E-06	0,328224995	0,322824993	0,330024996	0,377325007
6,00E-06	0,324125	0,320425012	0,365024992	0,326224991
1,00E-05	0,330625006	0,329424986	0,344324985	0,350624987
3,00E-05	0,469924995	0,480025002	0,484425017	0,518024989
6,00E-05	1,176124998	1,045124956	1,193625041	1,249224969
1,00E-04	1,544825026	1,502625057	1,504224964	1,564225026

(540nm-690nm)-solvent control E2 reference

cocentration [M]	Replicate 1	Replicate 2	Replicate 3	Replicate 4
1,00E-09	0,40610002	0,311099991	0,297400013	0,334099993

Normalization (substance/1 nM E2)

cocentration [M]	Replicate 1	Replicate 2	Replicate 3	Replicate 4	Mean
1,00E-06	85,03%	112,29%	115,95%	107,34%	**105,15%**
3,00E-06	80,82%	103,77%	110,97%	112,94%	**102,13%**
6,00E-06	79,81%	103,00%	122,74%	97,64%	**100,80%**
1,00E-05	81,41%	105,89%	115,78%	104,95%	**102,01%**
3,00E-05	115,72%	154,30%	162,89%	155,05%	**146,99%**
6,00E-05	289,61%	335,95%	401,35%	373,91%	**350,21%**
1,00E-04	380,41%	483,00%	505,79%	468,19%	**459,35%**

yeast-growth/solubility 690nm/solvent control 690nm

cocentration [M]	Replicate 1	Replicate 2	Replicate 3	Replicate 4	Mean
1,00E-06	90,26%	80,88%	152,29%	77,48%	**100,23%**
3,00E-06	86,46%	77,13%	152,71%	82,62%	**99,73%**
6,00E-06	84,44%	75,71%	162,79%	72,39%	**98,83%**
1,00E-05	88,15%	77,78%	158,93%	79,08%	**100,98%**
3,00E-05	88,02%	77,53%	156,34%	84,98%	**101,72%**
6,00E-05	85,89%	82,40%	159,91%	82,15%	**102,59%**
1,00E-04	86,00%	76,31%	152,42%	77,10%	**97,96%**

2nd experient

540nm

cocentration [M]	Replicate 1	Replicate 2	Replicate 3	Replicate 4
1,00E-06	0,980099976	0,930700004	1,056599975	0,985199988
3,00E-06	0,9375	0,988900006	0,999100029	0,9278
6,00E-06	0,966199994	0,940599978	0,980300009	0,967400014
1,00E-05	0,9199	1,027400017	1,027999997	0,972899973
3,00E-05	1,272400022	1,238999963	1,242200017	1,256500006
6,00E-05	2,140500069	2,19020009	2,142199993	2,157399893
1,00E-04	2,373600006	2,352400064	2,348999977	2,419100046

690nm

cocentration [M]	Replicate 1	Replicate 2	Replicate 3	Replicate 4
1,00E-06	0,469399989	0,446500003	0,539300025	0,475899994
3,00E-06	0,450100005	0,513199985	0,522800028	0,4542
6,00E-06	0,493800014	0,469599992	0,511399984	0,478700012
1,00E-05	0,457800001	0,528800011	0,550700009	0,511600018
3,00E-05	0,589999974	0,53670001	0,566999972	0,536899984
6,00E-05	0,56370002	0,514500022	0,514999986	0,483399987
1,00E-04	0,494500011	0,461800009	0,474299997	0,519999981

solvent control 690nm

	Replicate 1	Replicate 2	Replicate 3	Replicate 4
	0,623199999	0,583700001	0,487699986	0,664200008

540-690nm

cocentration [M]	Replicate 1	Replicate 2	Replicate 3	Replicate 4
1,00E-06	0,510699987	0,484200001	0,517199993	0,509299994
3,00E-06	0,487399995	0,475699991	0,476300001	0,473500013
6,00E-06	0,47240001	0,470999986	0,468899995	0,488700002
1,00E-05	0,462099999	0,498699993	0,477400005	0,461299986
3,00E-05	0,682399988	0,702300012	0,675199986	0,719600022
6,00E-05	1,576799989	1,675600052	1,627200007	1,674000025
1,00E-04	1,879099965	1,890599966	1,874600053	1,899199963

solvent control 540nm-690nm

	Replicate 1	Replicate 2	Replicate 3	Replicate 4
	0,108099997	0,111100003	0,100299999	0,116400003

(540nm-690nm)-solvent control (540nm-690nm)

cocentration [M]	Replicate 1	Replicate 2	Replicate 3	Replicate 4
1,00E-06	0,401724987	0,375225	0,408224992	0,400324993
3,00E-06	0,378424995	0,36672499	0,367325	0,364525013
6,00E-06	0,363425009	0,362024985	0,359924994	0,379725002
1,00E-05	0,353124999	0,389724992	0,368425004	0,352324985
3,00E-05	0,573424987	0,593325011	0,566224985	0,610625021
6,00E-05	1,467824988	1,566625051	1,518225007	1,565025024
1,00E-04	1,770124964	1,781624965	1,765625052	1,790224962

(540nm-690nm)-solvent control E2 reference

cocentration [M]	Replicate 1	Replicate 2	Replicate 3	Replicate 4
1,00E-09	0,431299984	0,381599993	0,374599993	0,377200007

Normalization (substance/1 nM E2)

cocentration [M]	Replicate 1	Replicate 2	Replicate 3	Replicate 4	Mean
1,00E-06	93,14%	98,33%	108,98%	106,13%	**101,64%**
3,00E-06	87,74%	96,10%	98,06%	96,64%	**94,64%**
6,00E-06	84,26%	94,87%	96,08%	100,67%	**93,97%**
1,00E-05	81,87%	102,13%	98,35%	93,41%	**93,94%**
3,00E-05	132,95%	155,48%	151,15%	161,88%	**150,37%**
6,00E-05	340,33%	410,54%	405,29%	414,91%	**392,77%**
1,00E-04	410,42%	466,88%	471,34%	474,61%	**455,81%**

yeast-growth/solubility 690nm/solvent control 690nm

cocentration [M]	Replicate 1	Replicate 2	Replicate 3	Replicate 4	Mean
1,00E-06	75,32%	76,49%	110,58%	71,65%	**83,51%**
3,00E-06	72,22%	87,92%	107,20%	68,38%	**83,93%**
6,00E-06	79,24%	80,45%	104,86%	72,07%	**84,15%**
1,00E-05	73,46%	90,59%	112,92%	77,02%	**88,50%**
3,00E-05	94,67%	91,95%	116,26%	80,83%	**95,93%**
6,00E-05	90,45%	88,14%	105,60%	72,78%	**89,24%**
1,00E-04	79,35%	79,12%	97,25%	78,29%	**83,50%**

3rd experiment

540nm

cocentration [M	Replicate 1	Replicate 2	Replicate 3	Replicate 4
1,00E-06	1,029299974	1,07280004	1,115700006	1,117499948
3,00E-06	1,038300037	1,125200033	1,12559998	1,121299982
6,00E-06	1,128399968	1,064700007	1,063199997	1,095600009
1,00E-05	1,083600044	1,086500049	1,132799983	1,173799992
3,00E-05	1,180699944	1,258100033	1,2852	1,353000045
6,00E-05	1,947999954	1,979799986	2,082799911	2,03489995
1,00E-04	2,282099962	2,227999926	2,358200073	2,358599901

690nm

cocentration [M	Replicate 1	Replicate 2	Replicate 3	Replicate 4
1,00E-06	0,4454	0,478700012	0,491299987	0,496300012
3,00E-06	0,457399994	0,527400017	0,536000013	0,506900012
6,00E-06	0,548500001	0,490099996	0,483599996	0,504000008
1,00E-05	0,515100002	0,51639998	0,541599989	0,565100014
3,00E-05	0,509299994	0,548799992	0,527999997	0,534200013
6,00E-05	0,517400026	0,485399991	0,490200013	0,476000011
1,00E-04	0,445899993	0,447299987	0,437299997	0,468100011

solvent control 690nm

	Replicate 1	Replicate 2	Replicate 3	Replicate 4
	0,667299986	0,712599993	0,666199982	0,557500005

540-690nm

cocentration [M	Replicate 1	Replicate 2	Replicate 3	Replicate 4
1,00E-06	0,583899975	0,594099998	0,62440002	0,621100008
3,00E-06	0,58099997	0,5977	0,589600027	0,614499986
6,00E-06	0,579900026	0,574599981	0,57950002	0,591700017
1,00E-05	0,568499982	0,570100009	0,591199994	0,608699977
3,00E-05	0,671299994	0,709299982	0,757200003	0,818799973
6,00E-05	1,430600047	1,494400024	1,592499971	1,559000015
1,00E-04	1,836199999	1,780699968	1,920899987	1,890499949

solvent control 540nm-690nm

	Replicate 1	Replicate 2	Replicate 3	Replicate 4
	0,102899998	0,103600003	0,106399998	0,083099999

(540nm-690nm)-solvent control (540nm-690nm)

cocentration [M	Replicate 1	Replicate 2	Replicate 3	Replicate 4
1,00E-06	0,484899975	0,495099999	0,52540002	0,522100009
3,00E-06	0,481999971	0,4987	0,490600027	0,515499987
6,00E-06	0,480900027	0,475599982	0,48050002	0,492700018
1,00E-05	0,469499983	0,47110001	0,492199995	0,509699978
3,00E-05	0,572299995	0,610299982	0,658200003	0,719799973
6,00E-05	1,331600048	1,395400025	1,493499972	1,460000016
1,00E-04	1,737199999	1,681699969	1,821899988	1,79149995

(540nm-690nm)-solvent control E2 reference

cocentration [M	Replicate 1	Replicate 2	Replicate 3	Replicate 4
1,00E-09	0,526925027	0,468824983	0,49712503	0,507725

Normalization (substance/1 nM E2)

cocentration [M	Replicate 1	Replicate 2	Replicate 3	Replicate 4	Mean
1,00E-06	92,02%	105,60%	105,69%	102,83%	**101,54%**
3,00E-06	91,47%	106,37%	98,69%	101,53%	**99,52%**
6,00E-06	91,27%	101,45%	96,66%	97,04%	**96,60%**
1,00E-05	89,10%	100,49%	99,01%	100,39%	**97,25%**
3,00E-05	108,61%	130,18%	132,40%	141,77%	**128,24%**
6,00E-05	252,71%	297,64%	300,43%	287,56%	**284,58%**
1,00E-04	329,69%	358,71%	366,49%	352,85%	**351,93%**

yeast-growth/solubility 690nm/solvent control 690nm

cocentration [M	Replicate 1	Replicate 2	Replicate 3	Replicate 4	Mean
1,00E-06	66,75%	67,18%	73,75%	89,02%	**74,17%**
3,00E-06	68,54%	74,01%	80,46%	90,92%	**78,48%**
6,00E-06	82,20%	68,78%	72,59%	90,40%	**78,49%**
1,00E-05	77,19%	72,47%	81,30%	101,36%	**83,08%**
3,00E-05	76,32%	77,01%	79,26%	95,82%	**82,10%**
6,00E-05	77,54%	68,12%	73,58%	85,38%	**76,15%**
1,00E-04	66,82%	62,77%	65,64%	83,96%	**69,80%**

4th experiment

540nm

cocentration [M	Replicate 1	Replicate 2	Replicate 3	Replicate 4
1,00E-06	0,902999997	0,920899987	0,914799988	0,944500029
3,00E-06	0,865199983	0,887000024	0,92049999	0,976300001
6,00E-06	0,867399991	0,883000016	0,891700029	0,871599972
1,00E-05	0,853399992	0,875400007	0,921999991	0,907500029
3,00E-05	0,957700014	0,995000005	1,063500047	1,059999943
6,00E-05	1,797000051	1,678799987	1,782199979	1,940799952
1,00E-04	2,012200117	2,016400099	2,041699886	2,168299913

690nm

cocentration [M	Replicate 1	Replicate 2	Replicate 3	Replicate 4
1,00E-06	0,472299993	0,481900007	0,477899998	0,486499995
3,00E-06	0,443800002	0,46360001	0,48269999	0,4921
6,00E-06	0,453999996	0,468199998	0,478500009	0,43810001
1,00E-05	0,450300008	0,47389999	0,49149999	0,466100007
3,00E-05	0,473399997	0,489300013	0,567900002	0,493099988
6,00E-05	0,448199987	0,476200014	0,462399989	0,491100013
1,00E-04	0,401800007	0,41960001	0,43599999	0,491899997

solvent control 690nm

	Replicate 1	Replicate 2	Replicate 3	Replicate 4
	0,581399977	0,580600023	0,595700026	0,596700013

540-690nm

cocentration [M	Replicate 1	Replicate 2	Replicate 3	Replicate 4
1,00E-06	0,430799991	0,43900001	0,436800003	0,458000004
3,00E-06	0,421299994	0,423400015	0,43779999	0,484200001
6,00E-06	0,413399994	0,414799988	0,413100004	0,433499992
1,00E-05	0,402999997	0,401499987	0,430599988	0,441399992
3,00E-05	0,484200001	0,505699992	0,495599985	0,566900015
6,00E-05	1,348799944	1,202600002	1,319800019	1,449699998
1,00E-04				

solvent control 540nm-690nm

	Replicate 1	Replicate 2	Replicate 3	Replicate 4
	0,097800002	0,098899998	0,102399997	0,100500003

(540nm-690nm)-solvent control (540nm-690nm)

cocentration [M	Replicate 1	Replicate 2	Replicate 3	Replicate 4
1,00E-06	0,330899991	0,339100011	0,336900003	0,358100004
3,00E-06	0,321399994	0,323500015	0,33789999	0,384300001
6,00E-06	0,313499995	0,314899988	0,313200004	0,333599992
1,00E-05	0,303099997	0,301599987	0,330699988	0,341499992
3,00E-05	0,384300001	0,405799992	0,395699985	0,467000015
6,00E-05	1,248899944	1,102700002	1,219900019	1,349799998
1,00E-04	1,510399945	1,496999986	1,505800016	1,576499946

(540nm-690nm)-solvent control E2 reference

cocentration [M	Replicate 1	Replicate 2	Replicate 3	Replicate 4
1,00E-09	0,379349984	0,384850003	0,386549987	0,385450013

Normalization (substance/1 nM E2)

cocentration [M	Replicate 1	Replicate 2	Replicate 3	Replicate 4	Mean
1,00E-06	87,23%	88,11%	87,16%	92,90%	**88,85%**
3,00E-06	84,72%	84,06%	87,41%	99,70%	**88,97%**
6,00E-06	82,64%	81,82%	81,02%	86,55%	**83,01%**
1,00E-05	79,90%	78,37%	85,55%	88,60%	**83,10%**
3,00E-05	101,30%	105,44%	102,37%	121,16%	**107,57%**
6,00E-05	329,22%	286,53%	315,59%	350,19%	**320,38%**
1,00E-04	398,15%	388,98%	389,55%	409,00%	**396,42%**

yeast-growth/solubility 690nm/solvent control 690nm

cocentration [M	Replicate 1	Replicate 2	Replicate 3	Replicate 4	Mean
1,00E-06	81,23%	83,00%	80,22%	81,53%	**81,50%**
3,00E-06	76,33%	79,85%	81,03%	82,47%	**79,92%**
6,00E-06	78,09%	80,64%	80,33%	73,42%	**78,12%**
1,00E-05	77,45%	81,62%	82,51%	78,11%	**79,92%**
3,00E-05	81,42%	84,27%	95,33%	82,64%	**85,92%**
6,00E-05	77,09%	82,02%	77,62%	82,30%	**79,76%**
1,00E-04	69,11%	72,27%	73,19%	82,44%	**74,25%**

5th experiment

540nm

cocentration [M	Replicate 1	Replicate 2	Replicate 3	Replicate 4
1,00E-06	1,210999966	1,147199988	1,116400003	1,151100039
3,00E-06	0,941399992	1,065700054	1,131600022	1,127599955
6,00E-06	1,099799991	1,059100032	1,063199997	1,113499999
1,00E-05	1,050699949	1,129899979	1,085299969	1,161200047
3,00E-05	1,241000056	1,354699969	1,327700019	1,504799962
6,00E-05	2,382200003	2,348799944	2,354000092	2,453999996
1,00E-04	2,401000023	2,361900091	2,414599895	2,376300097

690nm

cocentration [M	Replicate 1	Replicate 2	Replicate 3	Replicate 4
1,00E-06	0,555400014	0,566699982	0,561999977	0,550000012
3,00E-06	0,570900023	0,561999977	0,565999985	0,532899976
6,00E-06	0,551999986	0,519599974	0,513599992	0,522199988
1,00E-05	0,547599971	0,597599983	0,566900015	0,609300017
3,00E-05	0,592499971	0,613499999	0,586499989	0,6074
6,00E-05	0,540300012	0,563300014	0,562399983	0,524100006
1,00E-04	0,551299989	0,532599986	0,512499988	0,477200001

solvent control 690nm

	Replicate 1	Replicate 2	Replicate 3	Replicate 4
	0,595899999	0,614499986	0,58920002	0,620999992

540-690nm

cocentration [M	Replicate 1	Replicate 2	Replicate 3	Replicate 4
1,00E-06	0,655600011	0,580500007	0,554400027	0,601100028
3,00E-06	0,370499998	0,503799975	0,565699995	0,594699979
6,00E-06	0,547800004	0,539499998	0,549600005	0,591300011
1,00E-05	0,503199995	0,532299995	0,518299997	0,551900029
3,00E-05	0,648500025	0,741100013	0,74119997	0,897400022
6,00E-05	1,841899991	1,785599947	1,791599989	1,92990005
1,00E-04	1,849799991	1,829200029	1,902099967	1,899099946

solvent control 540nm-690nm

	Replicate 1	Replicate 2	Replicate 3	Replicate 4
	0,096299998	0,096100003	0,097099997	0,101199999

(540nm-690nm)-solvent control (540nm-690nm)

cocentration [M	Replicate 1	Replicate 2	Replicate 3	Replicate 4
1,00E-06	0,557925012	0,482825007	0,456725027	0,503425028
3,00E-06	0,272824999	0,406124976	0,468024995	0,497024979
6,00E-06	0,450125005	0,441824999	0,451925006	0,493625011
1,00E-05	0,405524995	0,434624996	0,420624997	0,45422503
3,00E-05	0,550825026	0,643425014	0,643524971	0,799725022
6,00E-05	1,744224992	1,687924948	1,69392499	1,832225051
1,00E-04	1,752124991	1,73152503	1,804424968	1,801424947

(540nm-690nm)-solvent control E2 reference

cocentration [M	Replicate 1	Replicate 2	Replicate 3	Replicate 4
1,00E-09	0,466699993	0,465999996	0,499100005	0,480500018

Normalization (substance/1 nM E2)

cocentration [M	Replicate 1	Replicate 2	Replicate 3	Replicate 4	Mean
1,00E-06	119,55%	103,61%	91,51%	104,77%	**104,86%**
3,00E-06	58,46%	87,15%	93,77%	103,44%	**85,71%**
6,00E-06	96,45%	94,81%	90,55%	102,73%	**96,14%**
1,00E-05	86,89%	93,27%	84,28%	94,53%	**89,74%**
3,00E-05	118,03%	138,07%	128,94%	166,44%	**137,87%**
6,00E-05	373,74%	362,22%	339,40%	381,32%	**364,17%**
1,00E-04	375,43%	371,57%	361,54%	374,91%	**370,86%**

yeast-growth/solubility 690nm/solvent control 690nm

cocentration [M	Replicate 1	Replicate 2	Replicate 3	Replicate 4	Mean
1,00E-06	93,20%	92,22%	95,38%	88,57%	**92,34%**
3,00E-06	95,80%	91,46%	96,06%	85,81%	**92,28%**
6,00E-06	92,63%	84,56%	87,17%	84,09%	**87,11%**
1,00E-05	91,89%	97,25%	96,22%	98,12%	**95,87%**
3,00E-05	99,43%	99,84%	99,54%	97,81%	**99,15%**
6,00E-05	90,67%	91,67%	95,45%	84,40%	**90,55%**
1,00E-04	92,52%	86,67%	86,98%	76,84%	**85,75%**

YES fenhexamid fludioxonil chlorpyrifos EC01

1st experient

540nm

cocentration [M]	Replicate 1	Replicate 2	Replicate 3	Replicate 4
1,00E-10	0,734799981	0,713500023	0,672299981	0,676400006
1,00E-09	0,713400006	0,76700002	0,722800016	0,726000011
1,00E-08	0,72420001	0,720700026	0,704200029	0,720099986
1,00E-07	0,719399989	0,704100013	0,668600023	0,7148
1,00E-06	0,731700003	0,683200002	0,721000016	0,702000022
1,00E-05	0,678600013	0,66839999	0,700900018	0,719799995
1,00E-04	0,704599977	0,714600027	0,708999991	0,710799992

690nm

cocentration [M]	Replicate 1	Replicate 2	Replicate 3	Replicate 4
1,00E-10	0,635299981	0,615499973	0,575500011	0,581200004
1,00E-09	0,61500001	0,666599989	0,627200007	0,631299973
1,00E-08	0,623199999	0,620899975	0,605499983	0,621800005
1,00E-07	0,619599998	0,606400013	0,572300017	0,615700006
1,00E-06	0,629700005	0,586399972	0,618499994	0,604499996
1,00E-05	0,547299981	0,541800022	0,569400012	0,584800005
1,00E-04	0,566699982	0,578499973	0,57160002	0,569999993

solvent control 690nm

	Replicate 1	Replicate 2	Replicate 3	Replicate 4
	0,604700029	0,583800018	0,592299998	0,565800011

540-690nm

cocentration [M]	Replicate 1	Replicate 2	Replicate 3	Replicate 4
1,00E-10	0,0995	0,097900003	0,096799999	0,095200002
1,00E-09	0,098399997	0,100500003	0,095600002	0,094700001
1,00E-08	0,101099998	0,099799998	0,098700002	0,098300003
1,00E-07	0,0999	0,0977	0,096299998	0,099100001
1,00E-06	0,101999998	0,096799999	0,102499999	0,097499996
1,00E-05	0,131400004	0,126599997	0,131500006	0,135000005
1,00E-04	0,137999997	0,136099994	0,137400001	0,140900001

solvent control 540nm-690nm

	Replicate 1	Replicate 2	Replicate 3	Replicate 4
	0,097099997	0,095399998	0,095200002	0,092799999

(540nm-690nm)-solvent control (540nm-690nm)

cocentration [M]	Replicate 1	Replicate 2	Replicate 3	Replicate 4
1,00E-10	0,004375001	0,002775004	0,001675	7,50031E-05
1,00E-09	0,003274998	0,005375003	0,000475002	-0,000424998
1,00E-08	0,005974999	0,004674999	0,003575003	0,003175003
1,00E-07	0,004775001	0,002575001	0,001174999	0,003975002
1,00E-06	0,006874999	0,001675	0,007375	0,002374997
1,00E-05	0,036275005	0,031474998	0,036375007	0,039875006
1,00E-04	0,042874997	0,040974995	0,042275002	0,045775002

(540nm-690nm)-solvent control E2 reference

cocentration [M]	Replicate 1	Replicate 2	Replicate 3	Replicate 4
1,00E-09	0,371175012	0,327574989	0,319475016	0,345174989

Normalization (substance/1 nM E2)

cocentration [M]	Replicate 1	Replicate 2	Replicate 3	Replicate 4	Mean
1,00E-10	1,18%	0,85%	0,52%	0,02%	**0,64%**
1,00E-09	0,88%	1,64%	0,15%	-0,12%	**0,64%**
1,00E-08	1,61%	1,43%	1,12%	0,92%	**1,27%**
1,00E-07	1,29%	0,79%	0,37%	1,15%	**0,90%**
1,00E-06	1,85%	0,51%	2,31%	0,69%	**1,34%**
1,00E-05	9,77%	9,61%	11,39%	11,55%	**10,58%**
1,00E-04	11,55%	12,51%	13,23%	13,26%	**12,64%**

yeast-growth/solubility 690nm/solvent control 690nm

cocentration [M]	Replicate 1	Replicate 2	Replicate 3	Replicate 4	Mean
1,00E-10	105,06%	105,43%	97,16%	102,72%	**102,59%**
1,00E-09	101,70%	114,18%	105,89%	111,58%	**108,34%**
1,00E-08	103,06%	106,35%	102,23%	109,90%	**105,39%**
1,00E-07	102,46%	103,87%	96,62%	108,82%	**102,94%**
1,00E-06	90,51%	92,81%	96,13%	103,36%	**95,70%**
1,00E-05	104,13%	100,45%	104,42%	106,84%	**103,96%**
1,00E-04	93,72%	99,09%	96,51%	100,74%	**97,51%**

2nd experient

540nm

cocentration [M]	Replicate 1	Replicate 2	Replicate 3	Replicate 4
1,00E-10	0,647599995	0,725499988	0,763000011	0,801599979
1,00E-09	0,716700017	0,755500019	0,720300019	0,712400019
1,00E-08	0,76849997	0,773899972	0,748399973	0,785399973
1,00E-07	0,769800007	0,827400029	0,803200006	0,733500004
1,00E-06	0,691200018	0,788500011	0,735300004	0,654500008
1,00E-05	0,744199991	0,798399985	0,773500025	0,721300006
1,00E-04	0,810699999	0,922100008	0,803600013	0,793799996

690nm

cocentration [M]	Replicate 1	Replicate 2	Replicate 3	Replicate 4
1,00E-10	0,551500022	0,624000013	0,657400012	0,695100009
1,00E-09	0,6153	0,6505	0,617799997	0,609899998
1,00E-08	0,660600007	0,666199982	0,644200027	0,677600026
1,00E-07	0,662	0,720499992	0,695200026	0,633099973
1,00E-06	0,592000008	0,680800021	0,630599976	0,555499971
1,00E-05	0,591799974	0,651199996	0,625999987	0,573400021
1,00E-04	0,644800007	0,752099991	0,638100028	0,629499972

solvent control 690nm

	Replicate 1	Replicate 2	Replicate 3	Replicate 4
	0,618399978	0,516200006	0,551999986	0,467799991

540-690nm

cocentration [M]	Replicate 1	Replicate 2	Replicate 3	Replicate 4
1,00E-10	0,096100003	0,101499997	0,105599999	0,1065
1,00E-09	0,101499997	0,104900002	0,102499999	0,102499999
1,00E-08	0,107799999	0,107699998	0,104199998	0,107699998
1,00E-07	0,107799999	0,106899999	0,108000003	0,100400001
1,00E-06	0,099299997	0,107600003	0,104800001	0,098999999
1,00E-05	0,152400002	0,147200003	0,147499993	0,148000002
1,00E-04	0,165900007	0,170000002	0,1655	0,164299995

solvent control 540nm-690nm

	Replicate 1	Replicate 2	Replicate 3	Replicate 4
	0,101999998	0,093400002	0,094700001	0,082900003

(540nm-690nm)-solvent control (540nm-690nm)

cocentration [M]	Replicate 1	Replicate 2	Replicate 3	Replicate 4
1,00E-10	0,002850002	0,008249996	0,012349999	0,013249999
1,00E-09	0,008249996	0,011650002	0,009249998	0,009249998
1,00E-08	0,014549999	0,014449997	0,010949997	0,014449997
1,00E-07	0,014549999	0,013649998	0,014750002	0,00715
1,00E-06	0,006049996	0,014350003	0,01155	0,005749999
1,00E-05	0,059150001	0,053950002	0,054249993	0,054750001
1,00E-04	0,072650006	0,076750001	0,072249999	0,071049994

(540nm-690nm)-solvent control E2 reference

cocentration [M]	Replicate 1	Replicate 2	Replicate 3	Replicate 4
1,00E-09	0,438549971	0,393449998	0,368649995	0,359449988

Normalization (substance/1 nM E2)

cocentration [M]	Replicate 1	Replicate 2	Replicate 3	Replicate 4	Mean
1,00E-10	0,65%	2,10%	3,35%	3,69%	**2,45%**
1,00E-09	1,88%	2,96%	2,51%	2,57%	**2,48%**
1,00E-08	3,32%	3,67%	2,97%	4,02%	**3,50%**
1,00E-07	3,32%	3,47%	4,00%	1,99%	**3,19%**
1,00E-06	1,38%	3,65%	3,13%	1,60%	**2,44%**
1,00E-05	13,49%	13,71%	14,72%	15,23%	**14,29%**
1,00E-04	16,57%	19,51%	19,60%	19,77%	**18,86%**

yeast-growth/solubility 690nm/solvent control 690nm

cocentration [M]	Replicate 1	Replicate 2	Replicate 3	Replicate 4	Mean
1,00E-10	89,18%	120,88%	119,09%	148,59%	**119,44%**
1,00E-09	99,50%	126,02%	111,92%	130,38%	**116,95%**
1,00E-08	106,82%	129,06%	116,70%	144,85%	**124,36%**
1,00E-07	107,05%	139,58%	125,94%	135,34%	**126,98%**
1,00E-06	95,73%	131,89%	114,24%	118,75%	**115,15%**
1,00E-05	95,70%	126,15%	113,41%	122,57%	**114,46%**
1,00E-04	104,27%	145,70%	115,60%	134,57%	**125,03%**

3rd experient

540nm

cocentration [M	Replicate 1	Replicate 2	Replicate 3	Replicate 4
1,00E-10	0,771700025	0,699199975	0,763199985	0,730300009
1,00E-09	0,743200004	0,685100019	0,752099991	0,774600029
1,00E-08	0,756099999	0,767099977	0,812900007	0,743099988
1,00E-07	0,787299991	0,790199995	0,774100006	0,828400016
1,00E-06	0,848100007	0,714600027	0,78490001	0,731400013
1,00E-05	0,786599994	0,724900007	0,679700017	0,679499984
1,00E-04	0,834999979	0,82130003	0,904200017	0,806100011

690nm

cocentration [M	Replicate 1	Replicate 2	Replicate 3	Replicate 4
1,00E-10	0,670899987	0,601199985	0,664200008	0,630100012
1,00E-09	0,642799973	0,593599975	0,654299974	0,674499989
1,00E-08	0,652800024	0,664799988	0,708599985	0,642099977
1,00E-07	0,684099972	0,68599999	0,666599989	0,72390002
1,00E-06	0,739300013	0,611800015	0,677699983	0,628199995
1,00E-05	0,624899983	0,564400017	0,524900019	0,525099993
1,00E-04	0,649600029	0,634299994	0,718100011	0,628600001

solvent control 690nm

	Replicate 1	Replicate 2	Replicate 3	Replicate 4
	0,679799974	0,571099997	0,556999981	0,310699999

540-690nm

cocentration [M	Replicate 1	Replicate 2	Replicate 3	Replicate 4
1,00E-10	0,1008	0,097999997	0,098999999	0,100100003
1,00E-09	0,100400001	0,091499999	0,097800002	0,100100003
1,00E-08	0,103299998	0,102300003	0,1043	0,101000004
1,00E-07	0,103200004	0,104199998	0,107500002	0,104500003
1,00E-06	0,108800001	0,102799997	0,107199997	0,103200004
1,00E-05	0,161699995	0,160500005	0,154799998	0,154499993
1,00E-04	0,185399994	0,186900005	0,186100006	0,177499995

solvent control 540nm-690nm

	Replicate 1	Replicate 2	Replicate 3	Replicate 4
	0,105700001	0,0933	0,092399999	0,062100001

(540nm-690nm)-solvent control (540nm-690nm)

cocentration [M	Replicate 1	Replicate 2	Replicate 3	Replicate 4
1,00E-10	0,012425	0,009624997	0,010624999	0,011725003
1,00E-09	0,012025001	0,003124999	0,009425001	0,011725003
1,00E-08	0,014924997	0,013925003	0,015924999	0,012625003
1,00E-07	0,014825003	0,015824998	0,019125002	0,016125003
1,00E-06	0,020425001	0,014424996	0,018824996	0,014825003
1,00E-05	0,073324995	0,072125005	0,066424998	0,066124992
1,00E-04	0,097024994	0,098525004	0,097725006	0,089124994

(540nm-690nm)-solvent control E2 reference

cocentration [M	Replicate 1	Replicate 2	Replicate 3	Replicate 4
1,00E-09	0,515624972	0,519425007	0,570425006	0,496224971

Normalization (substance/1 nM E2)

cocentration [M	Replicate 1	Replicate 2	Replicate 3	Replicate 4	Mean
1,00E-10	2,41%	1,85%	1,86%	2,36%	**2,12%**
1,00E-09	2,33%	0,60%	1,65%	2,36%	**1,74%**
1,00E-08	2,89%	2,68%	2,79%	2,54%	**2,73%**
1,00E-07	2,88%	3,05%	3,35%	3,25%	**3,13%**
1,00E-06	3,96%	2,78%	3,30%	2,99%	**3,26%**
1,00E-05	14,22%	13,89%	11,64%	13,33%	**13,27%**
1,00E-04	18,82%	18,97%	17,13%	17,96%	**18,22%**

yeast-growth/solubility 690nm/solvent control 690nm

cocentration [M	Replicate 1	Replicate 2	Replicate 3	Replicate 4	Mean
1,00E-10	98,69%	105,27%	119,25%	202,80%	**131,50%**
1,00E-09	94,56%	103,94%	117,47%	217,09%	**133,26%**
1,00E-08	96,03%	116,41%	127,22%	206,66%	**136,58%**
1,00E-07	100,63%	120,12%	119,68%	232,99%	**143,35%**
1,00E-06	108,75%	107,13%	121,67%	202,19%	**134,93%**
1,00E-05	91,92%	98,83%	94,24%	169,01%	**113,50%**
1,00E-04	95,56%	111,07%	128,92%	202,32%	**134,47%**

4th experient

540nm

cocentration [M	Replicate 1	Replicate 2	Replicate 3	Replicate 4
1,00E-10	0,736599982	0,718800008	0,696500003	0,80309999
1,00E-09	0,663699985	0,692799985	0,664900005	0,716600001
1,00E-08	0,742999971	0,725099981	0,755500019	0,728200018
1,00E-07	0,760100007	0,707799971	0,730400026	0,720300019
1,00E-06	0,685800016	0,692399979	0,689599991	0,72299999
1,00E-05	0,673900008	0,703100026	0,720899999	0,70480001
1,00E-04	0,807699978	0,756299973	0,725600004	0,737200022

690nm

cocentration [M	Replicate 1	Replicate 2	Replicate 3	Replicate 4
1,00E-10	0,634000003	0,616400003	0,598699987	0,696200013
1,00E-09	0,563000023	0,591400027	0,564899981	0,614199996
1,00E-08	0,636300027	0,620700002	0,649200022	0,625400007
1,00E-07	0,651000023	0,602800012	0,623399973	0,616599977
1,00E-06	0,58039999	0,585399985	0,584599972	0,615800023
1,00E-05	0,531000018	0,559499979	0,577099979	0,562699974
1,00E-04	0,644299984	0,597100019	0,571399987	0,581399977

solvent control 690nm

	Replicate 1	Replicate 2	Replicate 3	Replicate 4
	0,614499986	0,658200026	0,676900029	0,618799984

540-690nm

cocentration [M	Replicate 1	Replicate 2	Replicate 3	Replicate 4
1,00E-10	0,102600001	0,102300003	0,0977	0,106899999
1,00E-09	0,100699998	0,101400003	0,100000001	0,102399997
1,00E-08	0,106600001	0,104400001	0,106299996	0,102899998
1,00E-07	0,109200001	0,104999997	0,107000001	0,103799999
1,00E-06	0,105400003	0,107000001	0,105099998	0,107199997
1,00E-05	0,142900005	0,143600002	0,143900007	0,142100006
1,00E-04	0,163499996	0,159199998	0,154200003	0,1558

solvent control 540nm-690nm

	Replicate 1	Replicate 2	Replicate 3	Replicate 4
	0,104900002	0,105599999	0,109899998	0,104999997

(540nm-690nm)-solvent control (540nm-690nm)

cocentration [M	Replicate 1	Replicate 2	Replicate 3	Replicate 4
1,00E-10	-0,003749998	-0,004049996	-0,008649999	0,00055
1,00E-09	-0,005650006	-0,004949996	-0,006349998	-0,003950002
1,00E-08	0,000250002	-0,001949998	-5,00027E-05	-0,003450001
1,00E-07	0,002850002	-0,001350002	0,000650002	-0,00255
1,00E-06	-0,000949996	0,000650002	-0,001250001	0,000849998
1,00E-05	0,036550006	0,037250003	0,037550008	0,035750007
1,00E-04	0,057149997	0,052849999	0,047850003	0,049450001

(540nm-690nm)-solvent control E2 reference

cocentration [M	Replicate 1	Replicate 2	Replicate 3	Replicate 4
1,00E-09	0,433650022	0,424149996	0,431750005	0,432150012

Normalization (substance/1 nM E2)

cocentration [M	Replicate 1	Replicate 2	Replicate 3	Replicate 4	Mean
1,00E-10	-0,86%	-0,95%	-2,00%	0,13%	**-0,92%**
1,00E-09	-1,30%	-1,17%	-1,47%	-0,91%	**-1,21%**
1,00E-08	0,06%	-0,46%	-0,01%	-0,80%	**-0,30%**
1,00E-07	0,66%	-0,32%	0,15%	-0,59%	**-0,03%**
1,00E-06	-0,22%	0,15%	-0,29%	0,20%	**-0,04%**
1,00E-05	8,43%	8,78%	8,70%	8,27%	**8,55%**
1,00E-04	13,18%	12,46%	11,08%	11,44%	**12,04%**

yeast-growth/solubility 690nm/solvent control 690nm

cocentration [M	Replicate 1	Replicate 2	Replicate 3	Replicate 4	Mean
1,00E-10	103,17%	93,65%	88,45%	112,51%	**99,44%**
1,00E-09	91,62%	89,85%	83,45%	99,26%	**91,05%**
1,00E-08	103,55%	94,30%	95,91%	101,07%	**98,71%**
1,00E-07	105,94%	91,58%	92,10%	99,64%	**97,32%**
1,00E-06	94,45%	88,94%	86,36%	99,52%	**92,32%**
1,00E-05	86,41%	85,00%	85,26%	90,93%	**86,90%**
1,00E-04	104,85%	90,72%	84,41%	93,96%	**93,48%**

5th experiment

540nm

cocentration [M	Replicate 1	Replicate 2	Replicate 3	Replicate 4
1,00E-10	0,773800015	0,840300024	0,871800005	0,827799976
1,00E-09	0,825800002	0,827799976	0,872500002	0,815199971
1,00E-08	0,781099975	0,779600024	0,800000012	0,787800014
1,00E-07	0,737699986	0,764999986	0,818899989	0,777700007
1,00E-06	0,740800023	0,784200013	0,776600003	0,743200004
1,00E-05	0,761900008	0,74059999	0,771000028	0,712899983
1,00E-04	0,835399985	0,820500016	0,848200023	0,880800009

690nm

cocentration [M	Replicate 1	Replicate 2	Replicate 3	Replicate 4
1,00E-10	0,677200019	0,737800002	0,768899977	0,725499988
1,00E-09	0,724600017	0,725499988	0,768400013	0,712899983
1,00E-08	0,678600013	0,677699983	0,696799994	0,686800003
1,00E-07	0,640999973	0,665400028	0,715900004	0,676900029
1,00E-06	0,641700029	0,684300005	0,673900008	0,643400013
1,00E-05	0,627099991	0,608200014	0,638599992	0,584999979
1,00E-04	0,684800029	0,668799996	0,69749999	0,7245

solvent control 690nm

	Replicate 1	Replicate 2	Replicate 3	Replicate 4
	0,715300024	0,573099971	0,718100011	0,523899972

540-690nm

cocentration [M	Replicate 1	Replicate 2	Replicate 3	Replicate 4
1,00E-10	0,096600004	0,102499999	0,103	0,102300003
1,00E-09	0,101099998	0,102399997	0,104199998	0,102399997
1,00E-08	0,102600001	0,101899996	0,103200004	0,101000004
1,00E-07	0,096600004	0,099600002	0,103	0,100900002
1,00E-06	0,099100001	0,0999	0,102600001	0,099699996
1,00E-05	0,1347	0,132400006	0,132400006	0,127900004
1,00E-04	0,150600001	0,151600003	0,150700003	0,156299993

solvent control 540nm-690nm

	Replicate 1	Replicate 2	Replicate 3	Replicate 4
	0,097900003	0,092299998	0,101199999	0,087099999

(540nm-690nm)-solvent control (540nm-690nm)

cocentration [M	Replicate 1	Replicate 2	Replicate 3	Replicate 4
1,00E-10	0,001975004	0,007874999	0,008375	0,007675003
1,00E-09	0,006474998	0,007774998	0,009574998	0,007774998
1,00E-08	0,007975001	0,007274996	0,008575004	0,006375004
1,00E-07	0,001975004	0,004975002	0,008375	0,006275002
1,00E-06	0,004475001	0,005275	0,007975001	0,005074997
1,00E-05	0,040075	0,037775006	0,037775006	0,033275004
1,00E-04	0,055975001	0,056975003	0,056075003	0,061674993

(540nm-690nm)-solvent control E2 reference

cocentration [M	Replicate 1	Replicate 2	Replicate 3	Replicate 4
1,00E-09	0,480374988	0,495975014	0,477174994	0,56647497

Normalization (substance/1 nM E2)

cocentration [M	Replicate 1	Replicate 2	Replicate 3	Replicate 4	Mean
1,00E-10	0,41%	1,59%	1,76%	1,35%	**1,28%**
1,00E-09	1,35%	1,57%	2,01%	1,37%	**1,57%**
1,00E-08	1,66%	1,47%	1,80%	1,13%	**1,51%**
1,00E-07	0,41%	1,00%	1,76%	1,11%	**1,07%**
1,00E-06	0,93%	1,06%	1,67%	0,90%	**1,14%**
1,00E-05	8,34%	7,62%	7,92%	5,87%	**7,44%**
1,00E-04	11,65%	11,49%	11,75%	10,89%	**11,44%**

yeast-growth/solubility 690nm/solvent control 690nm

cocentration [M	Replicate 1	Replicate 2	Replicate 3	Replicate 4	Mean
1,00E-10	94,67%	128,74%	107,07%	138,48%	**117,24%**
1,00E-09	101,30%	126,59%	107,00%	136,08%	**117,74%**
1,00E-08	94,87%	118,25%	97,03%	131,09%	**110,31%**
1,00E-07	89,61%	116,11%	99,69%	129,20%	**108,65%**
1,00E-06	89,71%	119,40%	93,84%	122,81%	**106,44%**
1,00E-05	87,67%	106,12%	88,93%	111,66%	**98,60%**
1,00E-04	95,74%	116,70%	97,13%	138,29%	**111,96%**

ERα CALUX fenhexamid fludioxonil EC01

1st experient

luminescence

cocentration [M]	Replicate 1	Replicate 2	Replicate 3	Mean
1,00E-07	1677	1285	1171	**1378**
3,00E-07	1598	1455	1170	**1408**
6,00E-07	1602	1562	1396	**1520**
1,00E-06	2083	1663	1483	**1743**
2,00E-06	3323	2991	3134	**3149**
3,00E-06	4893	4215	4188	**4432**
6,00E-06	9480	9157	9244	**9294**
1,00E-05	13168	13311	15576	**14018**

solvent control

	Replicate 1	Replicate 2	Replicate 3	Replicate 4	Replicate 5	Replicate 6	Mean
	1936	1342	1246	1309	1386	1307	**1421**

substance-solvent control

cocentration [M]	Mean
1,00E-07	**-43**
3,00E-07	**-13**
6,00E-07	**99**
1,00E-06	**322**
2,00E-06	**1728**
3,00E-06	**3011**
6,00E-06	**7873**
1,00E-05	**12597**

E2 reference

cocentration [M]	Replicate 1	Replicate 2	Replicate 3	Mean
1,00E-10	15990	17958	18015	**17321**

E2 reference-solvent control

Mean
15900

Normalization (substance/0.1 nM E2)

cocentration [M]	**Mean**
1,00E-07	**-0,27%**
3,00E-07	**-0,08%**
6,00E-07	**0,62%**
1,00E-06	**2,03%**
2,00E-06	**10,87%**
3,00E-06	**18,94%**
6,00E-06	**49,51%**
1,00E-05	**79,23%**

2nd experient

luminescence

cocentration [M]	Replicate 1	Replicate 2	Replicate 3	Mean
1,00E-07	984	1037	970	**997**
3,00E-07	1075	1034	865	**991**
6,00E-07	1056	1101	1019	**1059**
1,00E-06	1422	1478	1346	**1415**
2,00E-06	2506	2642	2160	**2436**
3,00E-06	4117	4339	4227	**4228**
6,00E-06	8350	8475	8899	**8575**
1,00E-05	12119	12531	13563	**12738**

solvent control

	Replicate 1	Replicate 2	Replicate 3	Replicate 4	Replicate 5	Replicate 6	Mean
	986	1070	882	763	905	1098	**951**

substance-solvent control

cocentration [M]	Mean
1,00E-07	**18**
3,00E-07	**12**
6,00E-07	**79**
1,00E-06	**436**
2,00E-06	**1457**
3,00E-06	**3248**
6,00E-06	**7595**
1,00E-05	**11758**

E2 reference

cocentration [M]	Replicate 1	Replicate 2	Replicate 3	Mean
1,00E-10	14413	16654	15868	**15645**

E2 reference-solvent control

Mean
14694

Normalization (substance/0.1 nM E2)

cocentration [M]	**Mean**
1,00E-07	**0,12%**
3,00E-07	**0,08%**
6,00E-07	**0,54%**
1,00E-06	**2,97%**
2,00E-06	**9,91%**
3,00E-06	**22,11%**
6,00E-06	**51,69%**
1,00E-05	**80,02%**

3rd experient

luminescence

cocentration [M	Replicate 1	Replicate 2	Replicate 3	Mean
1,00E-07	894	691	971	**852**
3,00E-07	865	818	808	**830**
6,00E-07	1086	952	743	**927**
1,00E-06	1440	1151	1183	**1258**
2,00E-06	1980	2115	1720	**1938**
3,00E-06	3264	3278	3476	**3339**
6,00E-06	6461	7005	6671	**6712**
1,00E-05	9373	9718	10527	**9873**

solvent control

	Replicate 1	Replicate 2	Replicate 3	Replicate 4	Replicate 5	Replicate 6	Mean
	841	778	999	839	940	762	**860**

substance-solvent control

cocentration [M	Mean
1,00E-07	**-8**
3,00E-07	**-30**
6,00E-07	**67**
1,00E-06	**398**
2,00E-06	**1079**
3,00E-06	**2480**
6,00E-06	**5853**
1,00E-05	**9013**

E2 reference

cocentration [M	Replicate 1	Replicate 2	Replicate 3	Mean
1,00E-10	12063	11622	11400	**11695**

E2 reference-solvent control

Mean
10835

Normalization (substance/0.1 nM E2)

cocentration [M	**Mean**
1,00E-07	**-0,07%**
3,00E-07	**-0,27%**
6,00E-07	**0,62%**
1,00E-06	**3,67%**
2,00E-06	**9,95%**
3,00E-06	**22,88%**
6,00E-06	**54,01%**
1,00E-05	**83,18%**

ERα CALUX fenhexamid fludioxonil EC01

1st experient

luminescence

cocentration [M]	Replicate 1	Replicate 2	Replicate 3	Mean
1,00E-07	1176	1152	1585	**1304**
3,00E-07	1241	1218	1477	**1312**
6,00E-07	1726	1615	1294	**1545**
1,00E-06	1950	1554	1725	**1743**
2,00E-06	3146	2858	3630	**3211**
3,00E-06	4093	4333	4284	**4237**
6,00E-06	9520	8999	9526	**9348**
1,00E-05	15378	14929	15621	**15309**

solvent control

	Replicate 1	Replicate 2	Replicate 3	Replicate 4	Replicate 5	Replicate 6	Mean
	1936	1342	1246	1309	1386	1307	**1421**

substance-solvent control

cocentration [M]	Mean
1,00E-07	**-117**
3,00E-07	**-109**
6,00E-07	**124**
1,00E-06	**322**
2,00E-06	**1790**
3,00E-06	**2816**
6,00E-06	**7927**
1,00E-05	**13888**

E2 reference

cocentration [M]	Replicate 1	Replicate 2	Replicate 3	Mean
1,00E-10	15990	17958	18015	**17321**

E2 reference-solvent control

Mean
15900

Normalization (substance/0.1 nM E2)

cocentration [M]	**Mean**
1,00E-07	**-0,73%**
3,00E-07	**-0,69%**
6,00E-07	**0,78%**
1,00E-06	**2,03%**
2,00E-06	**11,26%**
3,00E-06	**17,71%**
6,00E-06	**49,86%**
1,00E-05	**87,35%**

2nd experient

luminescence

cocentration [M]	Replicate 1	Replicate 2	Replicate 3	Mean
1,00E-07	886	817	921	**875**
3,00E-07	993	992	1052	**1012**
6,00E-07	1104	1240	1349	**1231**
1,00E-06	1248	1125	1632	**1335**
2,00E-06	2588	2573	2814	**2658**
3,00E-06	4647	4099	4334	**4360**
6,00E-06	8939	9837	9613	**9463**
1,00E-05	13279	13000	13273	**13184**

solvent control

	Replicate 1	Replicate 2	Replicate 3	Replicate 4	Replicate 5	Replicate 6	Mean
	986	1070	882	763	905	1098	**951**

substance-solvent control

cocentration [M]	Mean
1,00E-07	**-105**
3,00E-07	**33**
6,00E-07	**252**
1,00E-06	**356**
2,00E-06	**1679**
3,00E-06	**3381**
6,00E-06	**8484**
1,00E-05	**12205**

E2 reference

cocentration [M]	Replicate 1	Replicate 2	Replicate 3	Mean
1,00E-10	14413	16654	15868	**15645**

E2 reference-solvent control

Mean
14694

Normalization (substance/0.1 nM E2)

cocentration [M]	**Mean**
1,00E-07	**-0,71%**
3,00E-07	**0,22%**
6,00E-07	**1,71%**
1,00E-06	**2,42%**
2,00E-06	**11,43%**
3,00E-06	**23,01%**
6,00E-06	**57,73%**
1,00E-05	**83,06%**

3rd experiment

luminescence

cocentration [M]	Replicate 1	Replicate 2	Replicate 3	Mean
1,00E-07	822	859	822	**834**
3,00E-07	847	776	912	**845**
6,00E-07	845	950	971	**922**
1,00E-06	1135	1205	1305	**1215**
2,00E-06	1970	2023	2281	**2091**
3,00E-06	3543	3286	3016	**3282**
6,00E-06	7189	6320	6808	**6772**
1,00E-05	9421	9917	9593	**9644**

solvent control

	Replicate 1	Replicate 2	Replicate 3	Replicate 4	Replicate 5	Replicate 6	Mean
	841	778	999	839	940	762	**860**

substance-solvent control

cocentration [M]	Mean
1,00E-07	**-26**
3,00E-07	**-15**
6,00E-07	**62**
1,00E-06	**355**
2,00E-06	**1232**
3,00E-06	**2422**
6,00E-06	**5913**
1,00E-05	**8784**

E2 reference

cocentration [M]	Replicate 1	Replicate 2	Replicate 3	Mean
1,00E-10	12063	11622	11400	**11695**

E2 reference-solvent control

Mean
10835

Normalization (substance/0.1 nM E2)

cocentration [M]	**Mean**
1,00E-07	**-0,24%**
3,00E-07	**-0,14%**
6,00E-07	**0,57%**
1,00E-06	**3,28%**
2,00E-06	**11,37%**
3,00E-06	**22,35%**
6,00E-06	**54,57%**
1,00E-05	**81,07%**

ERα CALUX chlorpyrifos fenhexamid fludioxonil EC01

1st experient

luminescence

cocentration [M]	Replicate 1	Replicate 2	Replicate 3	Mean
1,00E-07	1996	1248	1257	**1500**
3,00E-07	1604	1366	1231	**1400**
6,00E-07	1900	1503	1174	**1526**
1,00E-06	2057	1853	1492	**1801**
2,00E-06	3422	2988	2841	**3084**
3,00E-06	4495	4399	4037	**4310**
6,00E-06	8341	7295	7028	**7555**
1,00E-05	10700	11138	12303	**11380**

solvent control

	Replicate 1	Replicate 2	Replicate 3	Replicate 4	Replicate 5	Replicate 6	Mean
	1575	1483	1402	1322	1499	1089	**1395**

substance-solvent control

cocentration [M]	Mean
1,00E-07	**105**
3,00E-07	**5**
6,00E-07	**131**
1,00E-06	**406**
2,00E-06	**1689**
3,00E-06	**2915**
6,00E-06	**6160**
1,00E-05	**9985**

E2 reference

cocentration [M]	Replicate 1	Replicate 2	Replicate 3	Mean
1,00E-10	16682	17528	17866	**17359**

E2 reference-solvent control

Mean
15964

Normalization (substance/0.1 nM E2)

cocentration [M]	Mean
1,00E-07	**0,66%**
3,00E-07	**0,03%**
6,00E-07	**0,82%**
1,00E-06	**2,54%**
2,00E-06	**10,58%**
3,00E-06	**18,26%**
6,00E-06	**38,59%**
1,00E-05	**62,55%**

2nd experient

luminescence

cocentration [M]	Replicate 1	Replicate 2	Replicate 3	Mean
1,00E-07	1298	1302	1059	**1220**
3,00E-07	1313	1309	1144	**1255**
6,00E-07	1516	1383	1284	**1394**
1,00E-06	1743	1675	1545	**1654**
2,00E-06	2987	3288	3143	**3139**
3,00E-06	4528	4613	4191	**4444**
6,00E-06	8171	8184	8418	**8258**
1,00E-05	11303	11057	12376	**11579**

solvent control

	Replicate 1	Replicate 2	Replicate 3	Replicate 4	Replicate 5	Replicate 6	Mean
	1477	1031	1078	1048	1308	1121	**1177**

substance-solvent control

cocentration [M]	Mean
1,00E-07	**43**
3,00E-07	**78**
6,00E-07	**217**
1,00E-06	**477**
2,00E-06	**1962**
3,00E-06	**3267**
6,00E-06	**7081**
1,00E-05	**10402**

E2 reference

cocentration [M]	Replicate 1	Replicate 2	Replicate 3	Mean
1,00E-10	15439	16153	16273	**15955**

E2 reference-solvent control

Mean
14778

Normalization (substance/0.1 nM E2)

cocentration [M]	Mean
1,00E-07	**0,29%**
3,00E-07	**0,53%**
6,00E-07	**1,47%**
1,00E-06	**3,23%**
2,00E-06	**13,28%**
3,00E-06	**22,11%**
6,00E-06	**47,91%**
1,00E-05	**70,39%**

3rd experiment

luminescence

cocentration [M	Replicate 1	Replicate 2	Replicate 3	Mean
1,00E-07	1027	895	952	**958**
3,00E-07	940	904	813	**886**
6,00E-07	1010	971	825	**935**
1,00E-06	1389	956	1007	**1117**
2,00E-06	2181	1880	1897	**1986**
3,00E-06	3140	2773	2863	**2925**
6,00E-06	5461	5121	6015	**5532**
1,00E-05	8409	8024	8347	**8260**

solvent control

	Replicate 1	Replicate 2	Replicate 3	Replicate 4	Replicate 5	Replicate 6	Mean
	1014	935	832	751	813	803	**858**

substance-solvent control

cocentration [M	Mean
1,00E-07	**100**
3,00E-07	**28**
6,00E-07	**77**
1,00E-06	**259**
2,00E-06	**1128**
3,00E-06	**2067**
6,00E-06	**4674**
1,00E-05	**7402**

E2 reference

cocentration [M	Replicate 1	Replicate 2	Replicate 3	Mean
1,00E-10	11117	11081	11911	**11370**

E2 reference-solvent control

Mean
10512

Normalization (substance/0.1 nM E2)

cocentration [M	Mean
1,00E-07	**0,95%**
3,00E-07	**0,26%**
6,00E-07	**0,74%**
1,00E-06	**2,47%**
2,00E-06	**10,73%**
3,00E-06	**19,67%**
6,00E-06	**44,47%**
1,00E-05	**70,42%**

ERα CALUX chlorpyrifos fenhexamid fludioxonil EC10

1st experient

luminescence

cocentration [M]	Replicate 1	Replicate 2	Replicate 3	Mean
1,00E-07	1212	1141	1210	**1188**
3,00E-07	1325	1330	1167	**1274**
6,00E-07	1492	1184	1360	**1345**
1,00E-06	1422	1361	1653	**1479**
2,00E-06	1875	1855	1998	**1909**
3,00E-06	2878	2808	2519	**2735**
6,00E-06	5194	5379	4984	**5186**
1,00E-05	7471	8319	8501	**8097**

solvent control

	Replicate 1	Replicate 2	Replicate 3	Replicate 4	Replicate 5	Replicate 6	Mean
	1575	1483	1402	1322	1499	1089	**1395**

substance-solvent control

cocentration [M]	Mean
1,00E-07	**-207**
3,00E-07	**-121**
6,00E-07	**-50**
1,00E-06	**84**
2,00E-06	**514**
3,00E-06	**1340**
6,00E-06	**3791**
1,00E-05	**6702**

E2 reference

cocentration [M]	Replicate 1	Replicate 2	Replicate 3	Mean
1,00E-10	16682	17528	17866	**17359**

E2 reference-solvent control

Mean
15964

Normalization (substance/0.1 nM E2)

cocentration [M]	**Mean**
1,00E-07	**-1,30%**
3,00E-07	**-0,76%**
6,00E-07	**-0,31%**
1,00E-06	**0,52%**
2,00E-06	**3,22%**
3,00E-06	**8,39%**
6,00E-06	**23,75%**
1,00E-05	**41,98%**

2nd experient

luminescence

cocentration [M]	Replicate 1	Replicate 2	Replicate 3	Mean
1,00E-07	919	928	1157	**1001**
3,00E-07	1167	1074	1170	**1137**
6,00E-07	1114	1237	985	**1112**
1,00E-06	1531	1292	1406	**1410**
2,00E-06	2058	1873	2097	**2009**
3,00E-06	2253	2599	2753	**2535**
6,00E-06	5013	5346	5056	**5138**
1,00E-05	7910	7640	7910	**7820**

solvent control

	Replicate 1	Replicate 2	Replicate 3	Replicate 4	Replicate 5	Replicate 6	Mean
	1477	1031	1078	1048	1308	1121	**1177**

substance-solvent control

cocentration [M]	Mean
1,00E-07	**-176**
3,00E-07	**-40**
6,00E-07	**-65**
1,00E-06	**233**
2,00E-06	**832**
3,00E-06	**1358**
6,00E-06	**3961**
1,00E-05	**6643**

E2 reference

cocentration [M]	Replicate 1	Replicate 2	Replicate 3	Mean
1,00E-10	15439	16153	16273	**15955**

E2 reference-solvent control

Mean
14778

Normalization (substance/0.1 nM E2)

cocentration [M]	**Mean**
1,00E-07	**-1,19%**
3,00E-07	**-0,27%**
6,00E-07	**-0,44%**
1,00E-06	**1,57%**
2,00E-06	**5,63%**
3,00E-06	**9,19%**
6,00E-06	**26,80%**
1,00E-05	**44,95%**

3rd experient

luminescence

cocentration [M]	Replicate 1	Replicate 2	Replicate 3	Mean
1,00E-07	785	707	965	**819**
3,00E-07	798	931	851	**860**
6,00E-07	914	803	833	**850**
1,00E-06	852	1011	967	**943**
2,00E-06	1301	1375	1430	**1369**
3,00E-06	1543	1382	1466	**1464**
6,00E-06	3262	3329	3044	**3212**
1,00E-05	5539	5128	5454	**5374**

solvent control

	Replicate 1	Replicate 2	Replicate 3	Replicate 4	Replicate 5	Replicate 6	Mean
	1014	935	832	751	813	803	**858**

substance-solvent control

cocentration [M]	Mean
1,00E-07	**-39**
3,00E-07	**2**
6,00E-07	**-8**
1,00E-06	**85**
2,00E-06	**511**
3,00E-06	**606**
6,00E-06	**2354**
1,00E-05	**4516**

E2 reference

cocentration [M]	Replicate 1	Replicate 2	Replicate 3	Mean
1,00E-10	11117	11081	11911	**11370**

E2 reference-solvent control

Mean
10512

Normalization (substance/0.1 nM E2)

cocentration [M]	**Mean**
1,00E-07	**-0,37%**
3,00E-07	**0,02%**
6,00E-07	**-0,08%**
1,00E-06	**0,81%**
2,00E-06	**4,86%**
3,00E-06	**5,76%**
6,00E-06	**22,39%**
1,00E-05	**42,96%**

ERα CALUX propamocarb fenhexamid fludioxonil EC01

1st experient

luminescence

cocentration [M]	Replicate 1	Replicate 2	Replicate 3	Mean
1,00E-07	2357	1438	1521	**1772**
3,00E-07	1824	1330	1485	**1546**
6,00E-07	1901	1628	1323	**1617**
1,00E-06	2187	1577	1596	**1787**
2,00E-06	3096	3043	3237	**3125**
3,00E-06	4403	4564	4823	**4597**
6,00E-06	10287	10359	10072	**10239**
1,00E-05	15684	15813	15770	**15756**

solvent control

	Replicate 1	Replicate 2	Replicate 3	Replicate 4	Replicate 5	Replicate 6	Mean
	1787	1493	1457	1705	1554	1498	**1582**

substance-solvent control

cocentration [M]	Mean
1,00E-07	**190**
3,00E-07	**-36**
6,00E-07	**35**
1,00E-06	**204**
2,00E-06	**1543**
3,00E-06	**3014**
6,00E-06	**8657**
1,00E-05	**14173**

E2 reference

cocentration [M]	Replicate 1	Replicate 2	Replicate 3	Mean
1,00E-10	18346	19893	19940	**19393**

E2 reference-solvent control

Mean
17811

Normalization (substance/0.1 nM E2)

cocentration [M]	Mean
1,00E-07	**1,06%**
3,00E-07	**-0,20%**
6,00E-07	**0,20%**
1,00E-06	**1,15%**
2,00E-06	**8,66%**
3,00E-06	**16,92%**
6,00E-06	**48,61%**
1,00E-05	**79,58%**

2nd experient

luminescence

cocentration [M]	Replicate 1	Replicate 2	Replicate 3	Mean
1,00E-07	1678	1323	1111	**1371**
3,00E-07	1636	1208	1350	**1398**
6,00E-07	1572	1629	1132	**1444**
1,00E-06	1543	1677	1427	**1549**
2,00E-06	2591	2732	2248	**2524**
3,00E-06	4432	4182	4161	**4258**
6,00E-06	8916	8455	8857	**8743**
1,00E-05	11978	10820	12531	**11776**

solvent control

	Replicate 1	Replicate 2	Replicate 3	Replicate 4	Replicate 5	Replicate 6	Mean
	1482	1372	1271	1153	1113	1261	**1275**

substance-solvent control

cocentration [M]	Mean
1,00E-07	**95**
3,00E-07	**123**
6,00E-07	**169**
1,00E-06	**274**
2,00E-06	**1248**
3,00E-06	**2983**
6,00E-06	**7467**
1,00E-05	**10501**

E2 reference

cocentration [M]	Replicate 1	Replicate 2	Replicate 3	Mean
1,00E-10	15831	15200	16107	**15713**

E2 reference-solvent control

Mean
14437

Normalization (substance/0.1 nM E2)

cocentration [M]	Mean
1,00E-07	**0,66%**
3,00E-07	**0,85%**
6,00E-07	**1,17%**
1,00E-06	**1,90%**
2,00E-06	**8,65%**
3,00E-06	**20,66%**
6,00E-06	**51,72%**
1,00E-05	**72,74%**

3rd experiment

luminescence

cocentration [M	Replicate 1	Replicate 2	Replicate 3	Mean
1,00E-07	811	854	761	**809**
3,00E-07	824	707	793	**775**
6,00E-07	1040	911	666	**872**
1,00E-06	1399	1005	927	**1110**
2,00E-06	2104	1857	1914	**1958**
3,00E-06	2949	2979	2910	**2946**
6,00E-06	6148	6249	6395	**6264**
1,00E-05	8290	8937	8966	**8731**

solvent control

	Replicate 1	Replicate 2	Replicate 3	Replicate 4	Replicate 5	Replicate 6	Mean
	895	809	778	819	599	724	**771**

substance-solvent control

cocentration [M	Mean
1,00E-07	**38**
3,00E-07	**4**
6,00E-07	**102**
1,00E-06	**340**
2,00E-06	**1188**
3,00E-06	**2175**
6,00E-06	**5493**
1,00E-05	**7960**

E2 reference

cocentration [M	Replicate 1	Replicate 2	Replicate 3	Mean
1,00E-10	11678	11449	11359	**11495**

E2 reference-solvent control

Mean
10725

Normalization (substance/0.1 nM E2)

cocentration [M	Mean
1,00E-07	**0,35%**
3,00E-07	**0,04%**
6,00E-07	**0,95%**
1,00E-06	**3,17%**
2,00E-06	**11,07%**
3,00E-06	**20,28%**
6,00E-06	**51,22%**
1,00E-05	**74,22%**

ERα CALUX propamocarb fenhexamid fludioxonil EC01

1st experient

luminescence

cocentration [M]	Replicate 1	Replicate 2	Replicate 3	Mean
1,00E-07	1469	1583	1624	**1559**
3,00E-07	1474	1532	1463	**1490**
6,00E-07	1556	1328	1479	**1454**
1,00E-06	1681	1752	1744	**1726**
2,00E-06	2667	2335	3184	**2729**
3,00E-06	4076	4098	4305	**4160**
6,00E-06	8521	9130	9618	**9090**
1,00E-05	15197	16306	16662	**16055**

solvent control

	Replicate 1	Replicate 2	Replicate 3	Replicate 4	Replicate 5	Replicate 6	Mean
	1787	1493	1457	1705	1554	1498	**1582**

substance-solvent control

cocentration [M]	Mean
1,00E-07	**-24**
3,00E-07	**-93**
6,00E-07	**-128**
1,00E-06	**143**
2,00E-06	**1146**
3,00E-06	**2577**
6,00E-06	**7507**
1,00E-05	**14473**

E2 reference

cocentration [M]	Replicate 1	Replicate 2	Replicate 3	Mean
1,00E-10	18346	19893	19940	**19393**

E2 reference-solvent control

Mean
17811

Normalization (substance/0.1 nM E2)

cocentration [M]	**Mean**
1,00E-07	**-0,13%**
3,00E-07	**-0,52%**
6,00E-07	**-0,72%**
1,00E-06	**0,80%**
2,00E-06	**6,44%**
3,00E-06	**14,47%**
6,00E-06	**42,15%**
1,00E-05	**81,26%**

2nd experient

luminescence

cocentration [M]	Replicate 1	Replicate 2	Replicate 3	Mean
1,00E-07	973	1055	1134	**1054**
3,00E-07	993	992	1033	**1006**
6,00E-07	1189	1216	1052	**1152**
1,00E-06	1535	1294	1175	**1335**
2,00E-06	2172	1956	2139	**2089**
3,00E-06	2879	3214	3227	**3107**
6,00E-06	8333	8028	7567	**7976**
1,00E-05	12532	12543	12281	**12452**

solvent control

	Replicate 1	Replicate 2	Replicate 3	Replicate 4	Replicate 5	Replicate 6	Mean
	1482	1372	1271	1153	1113	1261	**1275**

substance-solvent control

cocentration [M]	Mean
1,00E-07	**-221**
3,00E-07	**-269**
6,00E-07	**-123**
1,00E-06	**59**
2,00E-06	**814**
3,00E-06	**1831**
6,00E-06	**6701**
1,00E-05	**11177**

E2 reference

cocentration [M]	Replicate 1	Replicate 2	Replicate 3	Mean
1,00E-10	15831	15200	16107	**15713**

E2 reference-solvent control

Mean
14437

Normalization (substance/0.1 nM E2)

cocentration [M]	**Mean**
1,00E-07	**-1,53%**
3,00E-07	**-1,87%**
6,00E-07	**-0,85%**
1,00E-06	**0,41%**
2,00E-06	**5,64%**
3,00E-06	**12,68%**
6,00E-06	**46,41%**
1,00E-05	**77,42%**

3rd experiment

luminescence

cocentration [M]	Replicate 1	Replicate 2	Replicate 3	Mean
1,00E-07	739	751	717	**736**
3,00E-07	726	704	873	**768**
6,00E-07	870	735	850	**818**
1,00E-06	987	836	853	**892**
2,00E-06	1590	1854	1427	**1624**
3,00E-06	2467	2023	1978	**2156**
6,00E-06	5801	5461	5176	**5479**
1,00E-05	8248	8771	8017	**8345**

solvent control

	Replicate 1	Replicate 2	Replicate 3	Replicate 4	Replicate 5	Replicate 6	Mean
	895	809	778	819	599	724	**771**

substance-solvent control

cocentration [M]	Mean
1,00E-07	**-35**
3,00E-07	**-3**
6,00E-07	**48**
1,00E-06	**121**
2,00E-06	**853**
3,00E-06	**1385**
6,00E-06	**4709**
1,00E-05	**7575**

E2 reference

cocentration [M]	Replicate 1	Replicate 2	Replicate 3	Mean
1,00E-10	11678	11449	11359	**11495**

E2 reference-solvent control

Mean
10725

Normalization (substance/0.1 nM E2)

cocentration [M]	**Mean**
1,00E-07	**-0,33%**
3,00E-07	**-0,03%**
6,00E-07	**0,44%**
1,00E-06	**1,13%**
2,00E-06	**7,95%**
3,00E-06	**12,92%**
6,00E-06	**43,91%**
1,00E-05	**70,63%**

ERβ CALUX fenhexamid fludioxonil EC01

1st experient

luminescence

cocentration [M]	Replicate 1	Replicate 2	Replicate 3	Mean
3,00E-07	200	179	173	**184**
6,00E-07	215	170	167	**184**
1,00E-06	226	197	169	**197**
2,00E-06	247	262	211	**240**
3,00E-06	316	375	244	**312**
6,00E-06	457	489	411	**452**
1,00E-05	569	674	588	**610**
3,00E-05	819	822	949	**863**

solvent control

	Replicate 1	Replicate 2	Replicate 3	Replicate 4	Replicate 5	Replicate 6	Mean
	173	166	178	162	159	196	**172**

substance-solvent control

cocentration [M]	Mean
3,00E-07	**12**
6,00E-07	**12**
1,00E-06	**25**
2,00E-06	**68**
3,00E-06	**139**
6,00E-06	**280**
1,00E-05	**438**
3,00E-05	**691**

E2 reference

cocentration [M]	Replicate 1	Replicate 2	Replicate 3	Mean
3,00E-08	1355	1464	1501	**1440**

E2 reference-solvent control

Mean
1268

Normalization (substance/30 nM E2)

cocentration [M]	**Mean**
3,00E-07	**0,92%**
6,00E-07	**0,92%**
1,00E-06	**1,97%**
2,00E-06	**5,34%**
3,00E-06	**10,99%**
6,00E-06	**22,09%**
1,00E-05	**34,55%**
3,00E-05	**54,51%**

2nd experient

luminescence

cocentration [M]	Replicate 1	Replicate 2	Replicate 3	Mean
3,00E-07	318	412	427	**386**
6,00E-07	343	388	353	**361**
1,00E-06	316	366	435	**372**
2,00E-06	403	460	412	**425**
3,00E-06	508	572	476	**519**
6,00E-06	561	642	614	**606**
1,00E-05	855	800	750	**802**
3,00E-05	1190	1099	1187	**1159**

solvent control

	Replicate 1	Replicate 2	Replicate 3	Replicate 4	Replicate 5	Replicate 6	Mean
	334	326	394	360	323	361	**350**

substance-solvent control

cocentration [M]	Mean
3,00E-07	**36**
6,00E-07	**12**
1,00E-06	**23**
2,00E-06	**75**
3,00E-06	**169**
6,00E-06	**256**
1,00E-05	**452**
3,00E-05	**809**

E2 reference

cocentration [M]	Replicate 1	Replicate 2	Replicate 3	Mean
3,00E-08	1480	1600	1386	**1489**

E2 reference-solvent control

Mean
1139

Normalization (substance/30 nM E2)

cocentration [M]	**Mean**
3,00E-07	**3,16%**
6,00E-07	**1,02%**
1,00E-06	**1,99%**
2,00E-06	**6,61%**
3,00E-06	**14,84%**
6,00E-06	**22,48%**
1,00E-05	**39,68%**
3,00E-05	**71,03%**

3rd experient

luminescence

cocentration [M	Replicate 1	Replicate 2	Replicate 3	Mean
3,00E-07	93	72	95	**87**
6,00E-07	100	69	86	**85**
1,00E-06	113	81	106	**100**
2,00E-06	115	145	93	**118**
3,00E-06	124	137	147	**136**
6,00E-06	162	175	169	**169**
1,00E-05	299	243	296	**279**
3,00E-05	439	516	494	**483**

solvent control

	Replicate 1	Replicate 2	Replicate 3	Replicate 4	Replicate 5	Replicate 6	Mean
	103	99	91	93	103	108	**100**

substance-solvent control

cocentration [M	Mean
3,00E-07	**-13**
6,00E-07	**-15**
1,00E-06	**1**
2,00E-06	**18**
3,00E-06	**37**
6,00E-06	**69**
1,00E-05	**180**
3,00E-05	**384**

E2 reference

cocentration [M	Replicate 1	Replicate 2	Replicate 3	Mean
3,00E-08	740	955	828	**841**

E2 reference-solvent control

Mean
742

Normalization (substance/30 nM E2)

cocentration [M	**Mean**
3,00E-07	**-1,73%**
6,00E-07	**-1,96%**
1,00E-06	**0,07%**
2,00E-06	**2,45%**
3,00E-06	**4,92%**
6,00E-06	**9,33%**
1,00E-05	**24,25%**
3,00E-05	**51,72%**

ERβ CALUX fenhexamid fludioxonil EC10

1st experient

luminescence

cocentration [M]	Replicate 1	Replicate 2	Replicate 3	Mean
3,00E-07	186	225	186	**199**
6,00E-07	182	185	189	**185**
1,00E-06	189	220	190	**200**
2,00E-06	233	229	253	**238**
3,00E-06	341	362	332	**345**
6,00E-06	488	413	437	**446**
1,00E-05	622	514	570	**569**
3,00E-05	989	780	642	**804**

solvent control

	Replicate 1	Replicate 2	Replicate 3	Replicate 4	Replicate 5	Replicate 6	Mean
	173	166	178	162	159	196	**172**

substance-solvent control

cocentration [M]	Mean
3,00E-07	**27**
6,00E-07	**13**
1,00E-06	**27**
2,00E-06	**66**
3,00E-06	**173**
6,00E-06	**274**
1,00E-05	**396**
3,00E-05	**631**

E2 reference

cocentration [M]	Replicate 1	Replicate 2	Replicate 3	Mean
3,00E-08	1355	1464	1501	**1440**

E2 reference-solvent control

Mean
1268

Normalization (substance/30 nM E2)

cocentration [M]	Mean
3,00E-07	**2,10%**
6,00E-07	**1,03%**
1,00E-06	**2,16%**
2,00E-06	**5,21%**
3,00E-06	**13,62%**
6,00E-06	**21,59%**
1,00E-05	**31,26%**
3,00E-05	**49,80%**

2nd experient

luminescence

cocentration [M]	Replicate 1	Replicate 2	Replicate 3	Mean
3,00E-07	387	333	373	**364**
6,00E-07	340	317	371	**343**
1,00E-06	390	389	377	**385**
2,00E-06	466	486	415	**456**
3,00E-06	528	461	550	**513**
6,00E-06	533	647	606	**595**
1,00E-05	717	711	647	**692**
3,00E-05	1174	1062	1419	**1218**

solvent control

	Replicate 1	Replicate 2	Replicate 3	Replicate 4	Replicate 5	Replicate 6	Mean
	334	326	394	360	323	361	**350**

substance-solvent control

cocentration [M]	Mean
3,00E-07	**15**
6,00E-07	**-7**
1,00E-06	**36**
2,00E-06	**106**
3,00E-06	**163**
6,00E-06	**246**
1,00E-05	**342**
3,00E-05	**869**

E2 reference

cocentration [M]	Replicate 1	Replicate 2	Replicate 3	Mean
3,00E-08	1480	1600	1386	**1489**

E2 reference-solvent control

Mean
1139

Normalization (substance/30 nM E2)

cocentration [M]	Mean
3,00E-07	**1,29%**
6,00E-07	**-0,61%**
1,00E-06	**3,13%**
2,00E-06	**9,31%**
3,00E-06	**14,34%**
6,00E-06	**21,57%**
1,00E-05	**30,03%**
3,00E-05	**76,27%**

3rd experient

luminescence

cocentration [M	Replicate 1	Replicate 2	Replicate 3	Mean
3,00E-07	106	99	94	**100**
6,00E-07	75	92	85	**84**
1,00E-06	87	97	97	**94**
2,00E-06	114	115	108	**112**
3,00E-06	105	158	125	**129**
6,00E-06	164	197	137	**166**
1,00E-05	321	295	202	**273**
3,00E-05	484	435	422	**447**

solvent control

	Replicate 1	Replicate 2	Replicate 3	Replicate 4	Replicate 5	Replicate 6	Mean
	103	99	91	93	103	108	**100**

substance-solvent control

cocentration [M	Mean
3,00E-07	**0**
6,00E-07	**-16**
1,00E-06	**-6**
2,00E-06	**13**
3,00E-06	**30**
6,00E-06	**67**
1,00E-05	**173**
3,00E-05	**348**

E2 reference

cocentration [M	Replicate 1	Replicate 2	Replicate 3	Mean
3,00E-08	740	955	828	**841**

E2 reference-solvent control

Mean
742

Normalization (substance/30 nM E2)

cocentration [M	Mean
3,00E-07	**0,02%**
6,00E-07	**-2,09%**
1,00E-06	**-0,79%**
2,00E-06	**1,73%**
3,00E-06	**4,02%**
6,00E-06	**8,97%**
1,00E-05	**23,35%**
3,00E-05	**46,86%**

ERβ CALUX propamocarb fenhexamid fludioxonil EC01

1st experient

luminescence

cocentration [M]	Replicate 1	Replicate 2	Replicate 3	Mean
3,00E-07	172	167	199	**179**
6,00E-07	170	160	164	**165**
1,00E-06	173	162	157	**164**
2,00E-06	156	172	178	**169**
3,00E-06	150	153	164	**156**
6,00E-06	196	143	182	**174**
1,00E-05	230	207	280	**239**
3,00E-05	523	490	625	**546**

solvent control

	Replicate 1	Replicate 2	Replicate 3	Replicate 4	Replicate 5	Replicate 6	Mean
	195	153	157	170	181	177	**172**

substance-solvent control

cocentration [M]	Mean
3,00E-07	**-8**
6,00E-07	**10**
1,00E-06	**-3**
2,00E-06	**-3**
3,00E-06	**-4**
6,00E-06	**9**
1,00E-05	**31**
3,00E-05	**222**

E2 reference

cocentration [M]	Replicate 1	Replicate 2	Replicate 3	Mean
3,00E-08	1885	1453	1829	**1722**

E2 reference-solvent control

Mean
1550

Normalization (substance/30 nM E2)

cocentration [M]	**Mean**
3,00E-07	**-0,53%**
6,00E-07	**0,66%**
1,00E-06	**-0,16%**
2,00E-06	**-0,20%**
3,00E-06	**-0,23%**
6,00E-06	**0,55%**
1,00E-05	**2,01%**
3,00E-05	**14,33%**

2nd experient

luminescence

cocentration [M]	Replicate 1	Replicate 2	Replicate 3	Mean
3,00E-07	321	367	352	**347**
6,00E-07	407	390	337	**378**
1,00E-06	416	369	410	**398**
2,00E-06	371	429	337	**379**
3,00E-06	345	333	387	**355**
6,00E-06	385	377	374	**379**
1,00E-05	421	385	420	**409**
3,00E-05	779	665	571	**672**

solvent control

	Replicate 1	Replicate 2	Replicate 3	Replicate 4	Replicate 5	Replicate 6	Mean
	392	318	372	398	376	358	**369**

substance-solvent control

cocentration [M]	Mean
3,00E-07	**-22**
6,00E-07	**9**
1,00E-06	**29**
2,00E-06	**10**
3,00E-06	**-14**
6,00E-06	**10**
1,00E-05	**40**
3,00E-05	**303**

E2 reference

cocentration [M]	Replicate 1	Replicate 2	Replicate 3	Mean
3,00E-08	1331	1795	1603	**1576**

E2 reference-solvent control

Mean
1207

Normalization (substance/30 nM E2)

cocentration [M]	**Mean**
3,00E-07	**-1,85%**
6,00E-07	**0,75%**
1,00E-06	**2,43%**
2,00E-06	**0,83%**
3,00E-06	**-1,16%**
6,00E-06	**0,80%**
1,00E-05	**3,29%**
3,00E-05	**25,07%**

3rd experient

luminescence

cocentration [M	Replicate 1	Replicate 2	Replicate 3	Mean
3,00E-07	76	92	74	**81**
6,00E-07	93	92	80	**88**
1,00E-06	97	80	86	**88**
2,00E-06	103	76	79	**86**
3,00E-06	114	86	97	**99**
6,00E-06	109	88	101	**99**
1,00E-05	137	123	116	**125**
3,00E-05	267	173	214	**218**

solvent control

	Replicate 1	Replicate 2	Replicate 3	Replicate 4	Replicate 5	Replicate 6	Mean
	92	85	77	86	76	90	**84**

substance-solvent control

cocentration [M	Mean
3,00E-07	**-4**
6,00E-07	**4**
1,00E-06	**3**
2,00E-06	**2**
3,00E-06	**15**
6,00E-06	**15**
1,00E-05	**41**
3,00E-05	**134**

E2 reference

cocentration [M	Replicate 1	Replicate 2	Replicate 3	Mean
3,00E-08	914	745	827	**829**

E2 reference-solvent control

Mean
744

Normalization (substance/30 nM E2)

cocentration [M	**Mean**
3,00E-07	**-0,49%**
6,00E-07	**0,54%**
1,00E-06	**0,45%**
2,00E-06	**0,22%**
3,00E-06	**1,97%**
6,00E-06	**2,02%**
1,00E-05	**5,51%**
3,00E-05	**17,96%**

ERβ CALUX propamocarb fenhexamid fludioxonil EC10

1st experient

luminescence

cocentration [M]	Replicate 1	Replicate 2	Replicate 3	Mean
3,00E-07	88	91	122	**100**
6,00E-07	75	79	91	**82**
1,00E-06	99	85	81	**88**
2,00E-06	76	84	76	**79**
3,00E-06	94	90	97	**94**
6,00E-06	88	93	112	**98**
1,00E-05	95	111	120	**109**
3,00E-05	243	196	219	**219**

solvent control

	Replicate 1	Replicate 2	Replicate 3	Replicate 4	Replicate 5	Replicate 6	Mean
	195	153	157	170	181	177	**172**

substance-solvent control

cocentration [M]	Mean
3,00E-07	**7**
6,00E-07	**-8**
1,00E-06	**-8**
2,00E-06	**-4**
3,00E-06	**-17**
6,00E-06	**2**
1,00E-05	**67**
3,00E-05	**374**

E2 reference

cocentration [M]	Replicate 1	Replicate 2	Replicate 3	Mean
3,00E-08	1885	1453	1829	**1722**

E2 reference-solvent control

Mean
1550

Normalization (substance/30 nM E2)

cocentration [M]	**Mean**
3,00E-07	**0,46%**
6,00E-07	**-0,48%**
1,00E-06	**-0,53%**
2,00E-06	**-0,23%**
3,00E-06	**-1,06%**
6,00E-06	**0,10%**
1,00E-05	**4,31%**
3,00E-05	**24,12%**

2nd experient

luminescence

cocentration [M]	Replicate 1	Replicate 2	Replicate 3	Mean
3,00E-07	369	360	314	**348**
6,00E-07	368	390	363	**374**
1,00E-06	310	311	325	**315**
2,00E-06	292	319	336	**316**
3,00E-06	404	357	351	**371**
6,00E-06	341	373	396	**370**
1,00E-05	440	406	379	**408**
3,00E-05	622	636	678	**645**

solvent control

	Replicate 1	Replicate 2	Replicate 3	Replicate 4	Replicate 5	Replicate 6	Mean
	392	318	372	398	376	358	**369**

substance-solvent control

cocentration [M]	Mean
3,00E-07	**-21**
6,00E-07	**5**
1,00E-06	**-54**
2,00E-06	**-53**
3,00E-06	**2**
6,00E-06	**1**
1,00E-05	**39**
3,00E-05	**276**

E2 reference

cocentration [M]	Replicate 1	Replicate 2	Replicate 3	Mean
3,00E-08	1331	1795	1603	**1576**

E2 reference-solvent control

Mean
1207

Normalization (substance/30 nM E2)

cocentration [M]	**Mean**
3,00E-07	**-1,77%**
6,00E-07	**0,39%**
1,00E-06	**-4,45%**
2,00E-06	**-4,42%**
3,00E-06	**0,14%**
6,00E-06	**0,08%**
1,00E-05	**3,26%**
3,00E-05	**22,89%**

3rd experiment

luminescence

cocentration [M	Replicate 1	Replicate 2	Replicate 3	Mean
3,00E-07	88	91	122	**100**
6,00E-07	75	79	91	**82**
1,00E-06	99	85	81	**88**
2,00E-06	76	84	76	**79**
3,00E-06	94	90	97	**94**
6,00E-06	88	93	112	**98**
1,00E-05	95	111	120	**109**
3,00E-05	243	196	219	**219**

solvent control

	Replicate 1	Replicate 2	Replicate 3	Replicate 4	Replicate 5	Replicate 6	Mean
	92	85	77	86	76	90	**84**

substance-solvent control

cocentration [M	Mean
3,00E-07	**16**
6,00E-07	**-3**
1,00E-06	**4**
2,00E-06	**-6**
3,00E-06	**9**
6,00E-06	**13**
1,00E-05	**24**
3,00E-05	**135**

E2 reference

cocentration [M	Replicate 1	Replicate 2	Replicate 3	Mean
3,00E-08	914	745	827	**829**

E2 reference-solvent control

Mean
744

Normalization (substance/30 nM E2)

cocentration [M	**Mean**
3,00E-07	**2,15%**
6,00E-07	**-0,36%**
1,00E-06	**0,54%**
2,00E-06	**-0,76%**
3,00E-06	**1,25%**
6,00E-06	**1,79%**
1,00E-05	**3,27%**
3,00E-05	**18,14%**
