# Supplementary material for: Mixture Effects of Estrogenic Pesticides at the Human Estrogen Receptor α and β
Source: PLoS One. 2016 Jan 26;11(1):e0147490. doi: 10.1371/journal.pone.0147490 (PMC4728068; doi:10.1371/journal.pone.0147490)
Supplement: S1 Table — Iso-effective mixtures based on EC01/EC10 or EC101/EC110 values of the single compounds. (PDF) [file pone.0147490.s007.pdf]

		Mixture ratios (percentages)	
**test system**	**compound**	**EC01**	**EC10**
ERα CALUX	fenhexamid	62.81%	67.61%
	fludioxonil	37.19%	32.39%
YES	fenhexamid	20.84%	44.86%
	fludioxonil	79.16%	55.14%
ERα CALUX	fenhexamid	38.48%	35,73%
	fludioxonil	22.79%	17.12%
	chlorpyrifos	38.73%	47.15%
YES	fenhexamid	19.37%	-
	fludioxonil	73.60%	
	chlorpyrifos	7.02%	
ERα CALUX	fenhexamid	51.73%	57.00%
	fludioxonil	30.63%	27.31%
	propamocarb	17.64%	15.69%
ERα CALUX	fenhexamid	62.81%	67.61%
	fludioxonil	37.19%	32.39%
YES	fenhexamid	20.84%	44.86%
	fludioxonil	79.16%	55.14%
		**EC101**	**EC110**
YES	chlorpyrifos	33.89%	47.14%
	fenarimol	66.11%	52.86%
		**EC01**	**EC10**
ERβ CALUX	fenhexamid	56.77%	70.56%
	fludioxonil	43.23%	29.44%
ERβ CALUX	fenhexamid	8.63%	15%
	fludioxonil	6.57%	6.26%
	propamocarb	84.80%	78.74%
