# Supplementary material for: Mixture Effects of Estrogenic Pesticides at the Human Estrogen Receptor α and β
Source: PLoS One. 2016 Jan 26;11(1):e0147490. doi: 10.1371/journal.pone.0147490 (PMC4728068; doi:10.1371/journal.pone.0147490)
Supplement: S2 Table — RM, the selected regression model; θ^1, θ^2 the estimated model parameters; θ^min, set 0; θ^max, the mean of the highest effect observed in the assay, corresponding to the effect induced by 1 nM E2. (PDF) [file pone.0147490.s008.pdf]

Concentration-response function

substance	**RM**	$\widehat{\boldsymbol{\theta}}_1$	$\widehat{\boldsymbol{\theta}}_2$	$\widehat{\boldsymbol{\theta}}_{\mathbf{min}}$	$\widehat{\boldsymbol{\theta}}_{\mathbf{max}}$
**chlorpyrifos**	Weibull	2.04	0.62	0	0.11
**fenarimol**	Weibull	36.03	8.44	0	2.69
**fludioxonil**	probit	6.73	1.35	0	0.14
**fenhexamid**	Weibull	8.43	1.62	0	0.11
**4,4‘-DDT**	probit	5.40	1.11	0	0.24
**2,4‘-DDT**	probit	4.58	0.92	0	0.93
