# Supplementary material for: Mixture Effects of Estrogenic Pesticides at the Human Estrogen Receptor α and β
Source: PLoS One. 2016 Jan 26;11(1):e0147490. doi: 10.1371/journal.pone.0147490 (PMC4728068; doi:10.1371/journal.pone.0147490)
Supplement: S3 Table — RM, the selected regression model; θ^1, θ^2 the estimated model parameters; θ^min, set 0; θ^max, the mean of the highest effect observed in the assay, corresponding to the effect induced by 0.1 nM E2. (PDF) [file pone.0147490.s009.pdf]

Concentration-response function

substance	**RM**	$\widehat{\boldsymbol{\theta}}_1$	$\widehat{\boldsymbol{\theta}}_2$	$\widehat{\boldsymbol{\theta}}_{\mathbf{min}}$	$\widehat{\boldsymbol{\theta}}_{\mathbf{max}}$
**propamocarb**	probit	10.49	1.92	0	1.01
**chlorpyrifos**	Weibull	16.06	3.20	0	0.47
**fenarimol**	logit	21.64	4.33	0	1.17
**fludioxonil**	Weibull	24.15	4.40	0	0.67
**fenhexamid**	Weibull	18.41	3.76	0	1.37
**4,4‘-DDT**	probit	17.04	3.04	0	0.75
**2,4‘-DDT**	probit	16.25	2.52	0	1.23
