# Supplementary material for: Mixture Effects of Estrogenic Pesticides at the Human Estrogen Receptor α and β
Source: PLoS One. 2016 Jan 26;11(1):e0147490. doi: 10.1371/journal.pone.0147490 (PMC4728068; doi:10.1371/journal.pone.0147490)
Supplement: S4 Table — RM, the selected regression model; θ^1, θ^2 the estimated model parameters; θ^min, set 0; θ^max, the mean of the highest effect observed in the assay, corresponding to the effect induced by 30 nM E2. (PDF) [file pone.0147490.s010.pdf]

Concentration-response function

substance	**RM**	$\hat{\boldsymbol{\theta}}_1$	$\hat{\boldsymbol{\theta}}_2$	$\hat{\boldsymbol{\theta}}_{\mathbf{min}}$	$\hat{\boldsymbol{\theta}}_{\mathbf{max}}$
**propamocarb**	Weibull	30.81	6.93	0	0.31
**fenarimol**	Weibull	21.74	4.54	0	0.20
**fludioxonil**	probit	18.55	3.39	0	0.42
**fenhexamid**	logit	20.06	4.02	0	0.46
**4,4‘-DDT**	probit	11.93	2.18	0	0.08
**2,4‘-DDT**	logit	14.58	2.50	0	0.37
