# Supplementary material for: Mixture Effects of Estrogenic Pesticides at the Human Estrogen Receptor α and β
Source: PLoS One. 2016 Jan 26;11(1):e0147490. doi: 10.1371/journal.pone.0147490 (PMC4728068; doi:10.1371/journal.pone.0147490)
Supplement: S5 Table — RM, the selected regression model; glogitII, generalized logit II; θ^1, θ^2, θ^3 the estimated model parameters; θ^min, set 1 if the effect is enhanced by the test compound in comparison to 1 nM E2 or the smallest mean value if the substance inhibits the effect of E2; θ^max, the mean of the highest effect observed in the assay, corresponding to the effect induced by 1 nM E2 or set to 1 if the substance inhibits the effect of E2; EC101/EC110, the effect concentration needed to elicit a 101% or 110% effect of 1 nM E2; [CI], the approximate 95% confidence interval. (PDF) [file pone.0147490.s011.pdf]

	Concentration-response function						EC101		EC110	
substance	**RM**	$\widehat{\theta}_1$	$\widehat{\theta}_2$	$\widehat{\theta}_3$	$\widehat{\theta}_{min}$	$\widehat{\theta}_{max}$	M	[CI]	M	[CI]
**chlorpyrifos**	logit	26.56	8.28	-	1	1.84	1.82E-04	[3.98E-05-4.68E-04]	3.55E-04	[2.09E-04-5.50E-04]
**fenarimol**	logit	182.96	42.40	-	1	4.36	3.55E-05	[9.55E-06-4.47E-05]	3.98E-05	[1.58E-05-4.68E-05]
**fludioxonil**	logit	-22.55	-3.93	-	0.56	1	-	-	-	-
**fenhexamid**	probit	-6.26	-1.19	-	0.21	1	-	-	-	-
**2,4‘-DDT**	probit	24.21	7.19	-	1	1.22	2.51E-04	[2.51E-05-5.01E-02]	4.17E-04	[9.12E-05-1.45E-01]
