# Supplementary material for: Mixture Effects of Estrogenic Pesticides at the Human Estrogen Receptor α and β
Source: PLoS One. 2016 Jan 26;11(1):e0147490. doi: 10.1371/journal.pone.0147490 (PMC4728068; doi:10.1371/journal.pone.0147490)
Supplement: S6 Table — RM, the selected regression model; θ^1, θ^2, the estimated model parameters; θ^min, set 1; θ^max, the mean of the highest effect observed in the assay, corresponding to the effect induced by 3 pM E2. (PDF) [file pone.0147490.s012.pdf]

Concentration-response function

substance	**RM**	$\widehat{\boldsymbol{\theta}}_1$	$\widehat{\boldsymbol{\theta}}_2$	$\widehat{\boldsymbol{\theta}}_{\min}$	$\widehat{\boldsymbol{\theta}}_{\max}$
**fludioxonil**	Weibull	47.44	8.59	1	4
**fenhexamid**	Weibull	21.33	3.97	1	9.73
