# Supplementary material for: Mixture Effects of Estrogenic Pesticides at the Human Estrogen Receptor α and β
Source: PLoS One. 2016 Jan 26;11(1):e0147490. doi: 10.1371/journal.pone.0147490 (PMC4728068; doi:10.1371/journal.pone.0147490)
Supplement: S7 Table — (PDF) [file pone.0147490.s013.pdf]

Concentration-response function

| substance | **RM** | $\hat{\boldsymbol{\theta}}_1$ | $\hat{\boldsymbol{\theta}}_2$ | $\hat{\boldsymbol{\theta}}_3$ | $\hat{\boldsymbol{\theta}}_{\min}$ | $\hat{\boldsymbol{\theta}}_{\max}$ |
|---|---|---|---|---|---|---|
| **100 µM fenarimol + 1 nM E2 + 4-hydroxytamoxifen** | glogitI | -7.34 | -1.55 | 9.48 | -0.22 | 4.76 |
| **1 mM chlorpyrifos +1 nM E2 + 4-hydroxytamoxifen** | probit | -5.45 | -0.83 | - | -0.2 | 1.26 |
| **100 µM fenarimol + 1 nM E2 + ICI 182,780** | glogitI | -10.24 | -3,68 | 18.76 | 0.73 | 4.35 |
| **1 mM chlorpyrifos + 1 nM E2 + ICI 182,780** | Weibull | -2.04 | -0.34 | - | 0.8 | 1.11 |
| **100 µM fenarimol + 4-hydroxytamoxifen** | Weibull | -8.21 | -0.98 | - | -0.21 | 2.77 |
| **100 µM fenarimol + ICI 182,780** | Weibull | -7.32 | -1.03 | - | 0.12 | 2.09 |
