# Supplementary material for: Mixture Effects of Estrogenic Pesticides at the Human Estrogen Receptor α and β
Source: PLoS One. 2016 Jan 26;11(1):e0147490. doi: 10.1371/journal.pone.0147490 (PMC4728068; doi:10.1371/journal.pone.0147490)
Supplement: S8 Table — (PDF) [file pone.0147490.s014.pdf]

Concentration-response function

substances	**RM**	$\widehat{\boldsymbol{\theta}}_1$	$\widehat{\boldsymbol{\theta}}_2$	$\widehat{\boldsymbol{\theta}}_{\min}$	$\widehat{\boldsymbol{\theta}}_{\max}$
**60 µM fenhexamid + tamoxifen**	Weibull	-83.73	-14.99	-0.02	1.49
