# Supplementary material for: Mixture Effects of Estrogenic Pesticides at the Human Estrogen Receptor α and β
Source: PLoS One. 2016 Jan 26;11(1):e0147490. doi: 10.1371/journal.pone.0147490 (PMC4728068; doi:10.1371/journal.pone.0147490)
Supplement: S9 Table — (PDF) [file pone.0147490.s015.pdf]

	Concentration-response function						EC101		EC110	
mixture	**RM**	$\hat{\boldsymbol{\theta}}_1$	$\hat{\boldsymbol{\theta}}_2$	$\hat{\boldsymbol{\theta}}_3$	$\hat{\boldsymbol{\theta}}_{min}$	$\hat{\boldsymbol{\theta}}_{max}$	predicted M	observed M [CI]	predicted M	observed M [CI]
**chlorpyrifos fenarimol EC101**	glogitI	37.34	7.62	33.22	1	4.39	4.90E-05	2.04E-05 [1.15E-05-2.82E-05]	5.75E-05	2.45E-05 [1.74E-05-2.88E-05]
**chlorpyrifos fenarimol EC110**	logit	48.77	11.21	-	1	4.05	5.62E-05	1.38E-05 [8.91E-06-2.34E-05]	6.92E-05	2.24E-05 [1.86E-05-3.09E-05]
